# Design, Synthesis, and Anti-Fungal Evaluation of Heterocyclic Benzoxazole Derivatives

**DOI:** 10.3390/molecules27238375

**Published:** 2022-11-30

**Authors:** Ruibo Wang, Ruiting Kang, Xuan Yang, Yu Cheng, Hongjin Bai, Zhenting Du

**Affiliations:** 1 Engineering Laboratory of Chemical Resources Utilization in South Xinjiang of Xinjiang Production and Construction Corps, Tarim University, Alar 843300, China; 2 College of Chemistry and Pharmacy, Northwest A&F University, Xianyang 712100, China; 3 Key Laboratory of Botanical Pesticide R&D in Shaanxi Province, Xianyang 712100, China

**Keywords:** benzoxazole, crystal structure, synthesis, anti-fungal activities

## Abstract

In order to discover more promising anti-fungal agents, a series of benzoxazole family was synthesized by PPA-catalyzed condensation and a Raney nickel/hydrazine reduction. Altogether 45 compounds were obtained in good to excellent yields and characterized by FT-IR, NMR, MS, and X-ray crystal diffraction. Moreover, the biological activity against eight phytopathogenic fungi was investigated. All in all, most of these compounds bear moderate antifungal activities. Among them, three candidates show the strongest activities, compound **4ac, 4bc** provided over 50% inhibition rate against five fungi. Especially, the inhibitory rate of compound **4ah** on *Mycosphaerella melonis* reached 76.4%.

## 1. Introduction

**Figure 1:**
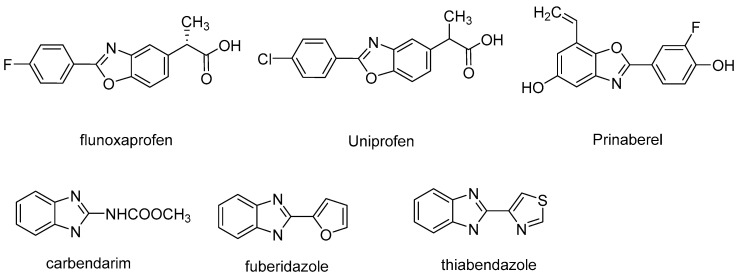
Typical compounds of benzoxazoles.

In recent years, pathogenic bacteria and plant viruses targeting agricultural crops have become increasingly difficult to control, leading to enormous losses in worldwide crop production each year. As such, the development of environmentally friendly pesticides with high selectivity, high efficiency, low toxicity, and suitable degradation are important challenges to both chemists and biologists [1]. Development of drug-like heterocyclic compounds from simple and facile substrates is one of the emerging areas in modern synthetic chemistry. Heterocyclic structures are important key features in natural products or synthetic medicines and pesticides because of their high-efficiency, low toxicity, and diversity of possible substituents [2]. As shown in Figure 1, the benzoxazole is an important heteroaromatic moiety found in many pharmaceuticals and agrichemicals [3,4,5]. Benzoxazole is a structural scaffold which can be found in nonsteroidal anti-inflammatory drugs such as flunoxaprofen [6], uniprofen [7], and Prinaberel [8]. Many benzo heterocycles compounds can be found in agrichemicals, such as carbendarim [9], fubridazole [10], and thiabendazole [11]. There are also other pharmaceutical applications such as antibiotic [12,13], anticancer, [14,15,16] anti-inflammatory applications [17]. Due to their structural characteristics, they can be used in material industry such as fluorescent chemosensor for special ions [18] and mesogenic applications [19].

**Scheme 1:**
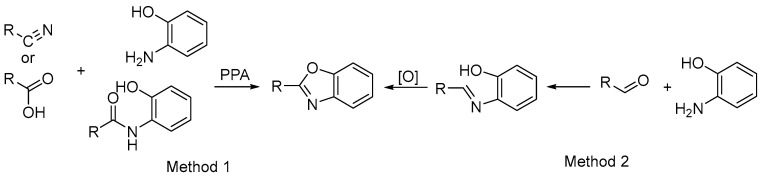
The syntheses of benzoxazoles.

**Figure 2:**
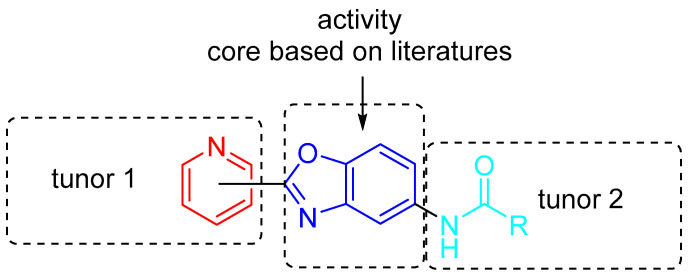
The molecular structural design idea.

Benzoxazoles are important members of the family of fused heterocycles that have attracted much attention because of their diverse biological activity. In order to find new potential pesticide molecules with antifungal activities, we designed and synthesized a series of benzoxazole derivatives and evaluated their potential antifungal activities against eight kinds of plant pathogenic fungus commonly found in agriculture systematically, including *Fusarium solani, Colletotrichum gloeosporioides, Mycosphaerella melonis, Alternaria brassicae, Pyricularia grisea, Curvularia lunata, Alternaria solani,* and *Fusarium graminearum*. As Scheme 1 shows, namely the synthesis of benzoxazoles can be classified into two kinds: [20,21] one is using PPA as solvent and catalyst at the presence of acid and aniline in one step [15,17,22], the other is using condensation of aldehyde and aniline then an oxidation step using some oxidizer such as DDQ, Manganese oxide, air, etc. [23]. Our research group has been intensively studying the synthesis of heterocycles and their agricultural applications [24,25,26]. Pyridine, also known as azabenzene, has a certain aromatic structure and is widely used in sulfonamides and pesticides. Inspired by three molecules used as agrichemicals in Figure 1, we envisioned a combination of pyridine, benzoxazole, and amide might have good antifungal activities against plant pathogen (Figure 2). Prompted by the above thoughts, we wish to report the synthesis, spectroscopic, single-crystal diffraction, antifungal activity, of a series of benzoxazoles bearing a pyridine ring and amide moiety. In our devise, the two heterocyclic moiety contributes the biological activities, and the amide would tune the activities.

## 2. Results and Discussion

### 2.1. Chemistry

**Scheme 2:**
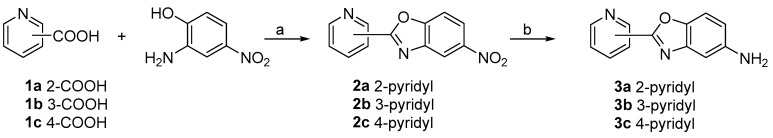
The preparation of benzoxazoles. (a: PPA, 140–150 °C, 6–8 h; b: W-2 Raney Ni, NH_2_NH_2_, 60 °C, 5 h).

**Figure 3:**
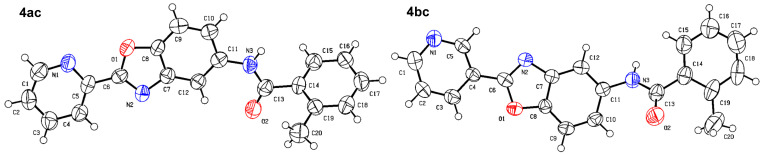
X-ray crystallographic structures of **4ac** and **4bc**.

As shown in Scheme 2, the isomers (2-, 3-, 4-, respectively) of picolinic acid condensed with 4-nitro-2-aminophenol at the presence of PPA to provide the three types of pyridyl-benzoxazole in 75–85% yield. Compared with the reported method, the PPA was preheated to 80 °C before use in order to be measured easily. Next, a mild reduction in the nitro group to amino group was adopted using hydrazine and Raney nickel in 75–80% yield. The other reduction methods proved unsuccessful, such as palladium/carbon catalytic hydrogenation, stannous chloride in common solvent (ethyl acetate and tetrahydro furan), and iron powder in acetic acid. A lot of black tar which can be solved in acidic condition was produced under these conditions. We suspected that the oxales ring was opened upon these conditions. As to the preparation of the nitro-benzoxazoles, there is another nitration method of benzoxazole, but the nitro group position is limited to the *para*- to the OH group. Next, we condensed three pyridyl-benzoxazole compounds with 15 different types of acyl chlorides to obtain 45 amide derivatives (**4aa–4co**) in fairly good to excellent yield (Table 1) and were characterized by ^1^H NMR and ^13^C NMR. The stereochemistry of two compounds **4ac** and **4bc** with the best antifungal effect was further confirmed by the X-ray crystallographic analysis (Figure 3).

### 2.2. X-ray Crystallographic Studies of **4ac**, **4bc**

Compound **4ac** crystallizes in the monoclinic space group P2_1_. There are two crystallographic independent molecules in the asymmetric unit (only one of them is shown in Figure 3). In the molecule of **4ac**, bond lengths and angles are very similar to those provided in the literature for benzoxazole derivative. The benzoxazole ring was approximately planar. Compared with the **4bc**, the dihedral angle between the methylphenyl plane and the oxazole ring was 55.65°. Compound **4bc** crystallizes in the monoclinic space group C2/c. There are eight crystallographic independent molecules in the asymmetric unit (only one of them is shown in Figure 3). In the molecule of **4bc**, bond lengths and angles are very similar to those provided in the literature for benzoxazole derivative. The whole molecule was approximately planar. The dihedral angle of the methylphenyl plane, the oxazole ring, was 8.61°. X-ray diffraction reflection data of 4ac and 4bc are shown in the Supplementary Materials.

### 2.3. In Vitro Anti-Fungal Activity

**Figure 4:**
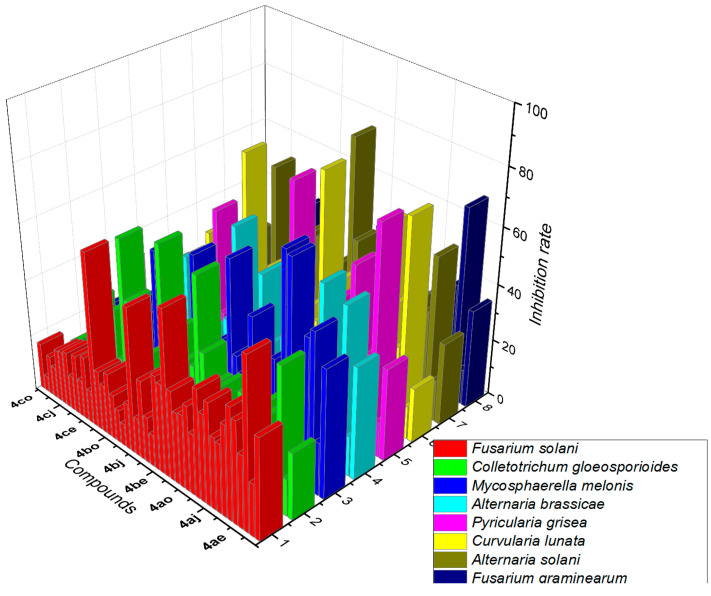
Antifungal activities of compounds **4aa**–**4co** at 100 μg/mL.

Meanwhile, the anti-fungal activities of these compounds were evaluated against eight common phytopathogenic fungi, (*Fusarium solani*, *Colletotrichum gloeosporioides*, *Mycosphaerella melonis*, *Alternaria brassicae*, *Pyricularia grisea*, *Curvularia lunata*, *Alternaria solani*, and *Fusarium graminearum*) using the inhibition zone method at the concentration of 100 μg/mL by the poisoned food technique. While thiophanate-methyl, which is a fungicide bearing similar heterocyclic structures and a commercially available agricultural fungicide, was used as a positive control at 100 μg/mL. As shown in Figure 4, the experiments revealed that most of these compounds bears moderate result to *Colletotrichum gloeosporioides* and *Mycosphaerella melonis*. Through the 3D -Bar of inhibition rate against eight fungi, we could see, compound **4ac, 4bc** showed best results, providing over 50% inhibition rate against five fungi. Herein, we believe the methyl makes a rotation barrier, and to some extent enhances the combination of compound to the enzyme. Interestingly, some compounds could selectively inhibit some fungi. For example, the inhibitory rate of compound **4ah** on *Mycosphaerella melonis* could reach 76.4%, but it had little inhibitory activity on other fungi. Another example was that compound 4bh could highly selectively inhibit the growth of *Alternaria brassicae*. However, **4be** against *Fusarium solani*, **4cj** and **4af** against *Colletotrichum gloeosporioides* provided unsatisfactory results, even positive results. From the whole table, the effect of acyl ligand on antifungal activity was greater than that of pyridyl-benzoxazole skeleton. Typical examples are compounds **4ac** and **4bc**, and even compound **4cc**, which have very good antifungal activity and all of them have the same acyl ligand.

## 3. Materials and Methods

### 3.1. Chemistry

All chemicals were purchased from Energy chemicals and Merck (used without further purification). The melting point was determined using a digital melting point meter WRS-1B. The ^1^H NMR and ^13^C NMR spectra were carried out by AV 500 MHz spectrometer. X-ray analysis of the samples were recorded on a Bruker APEX II area detector diffractometer at 296(2) K with a graphite-monochromatic MoK radiation (λ = 0.71073A°).

#### 3.1.1. General Synthesis of **3a**–**3c**

5-Nitro-2-(pyridin-2-yl)benzo[*d*]oxazole (**3a**). To a solution of 2-picolinic acid (2.46 g 0.01 mol) condensed with 4-nitro-2-aminophenol (3.08 g, 0.01 mol) in polyphosphoric acid (30 mL) in a 100mL three-necked flask, the middle neck was installed a mechanical stirring, and the side neck was set for thermometer. The reaction mixture was heated 140 °C and the external temperature was kept at 150 °C. After 6 h, the reaction was completed through TLC. Then the reaction mixture was poured into water slowly and was adjusted to pH 6 using potassium carbonate. A quantity of solid precipitated and was collected by suction. This compound was used without further purification and drying. Because the next reduction was carried out in ethyl alcohol, the crude product **2a** was used directly. The crude **2a** was dissolved in 95% ethyl alcohol (40 mL), and then was added W-2 Raney nickel (ca. 0.20 g), and then hydrazine (1.96 g, 3 equiv.) was introduced drop wise. Moreover, the mixture was heated to 60 °C and monitored by TLC. After completion, the reaction mixture was filtered through a Celite pad. The filtration was condensed and purified by flash column chromatography (petroleum ether: ethyl acetate) to obtain **3a** (1.94 g, 72% for two steps) as a brown solid: ^1^H NMR (500 MHz, DMSO) δ 8.78 (d, *J* = 4.1 Hz, 1H), 8.29 (d, *J* = 7.9 Hz, 1H), 8.05 (dd, *J* = 10.9, 4.5 Hz, 1H), 7.61 (dd, *J* = 6.9, 5.1 Hz, 1H), 7.49 (d, *J* = 8.7 Hz, 1H), 6.95 (d, *J* = 1.6 Hz, 1H), 6.77 (dd, *J* = 8.7, 1.9 Hz, 1H), 5.19 (s, 2H); ^13^C NMR (126 MHz, DMSO) δ 161.53, 150.55, 147.35, 146.12, 143.46, 142.78, 138.02, 126.21, 123.63, 114.66, 111.38, 103.16.

2-(Pyridin-3-yl)benzo[*d*]oxazol-5-amine (**3b**) and 2-(pyridin-4-yl)benzo[*d*]oxazol-5-amine (**3c**) could be obtained in same procedure as a brown solid in a similar yield, respectively.

Compound **3b**: ^1^H NMR (500 MHz, DMSO) δ 9.31 (s, 1H), 8.79 (s, 1H), 8.48 (d, *J* = 6.7 Hz, 1H), 7.64 (s, 1H), 7.48 (d, *J* = 8.6 Hz, 1H), 6.93 (s, 1H), 6.74 (d, *J* = 8.6 Hz, 1H), 5.21 (s, 2H); ^13^C NMR (126 MHz, DMSO) δ 160.40, 152.31, 148.16, 147.35, 143.20, 142.75, 134.80, 124.72, 123.69, 114.18, 111.18, 103.03.

Compund **3c:** ^1^H NMR (500 MHz, DMSO) δ 8.83 (d, *J* = 5.6 Hz, 2H), 8.05 (d, *J* = 5.7 Hz, 2H), 7.51 (d, *J* = 8.7 Hz, 1H), 6.95 (d, *J* = 1.7 Hz, 1H), 6.79 (dd, *J* = 8.7, 1.9 Hz, 1H), 5.27 (s, 2H); ^13^C NMR (126 MHz, DMSO) δ 160.31, 151.26, 147.53, 143.37, 142.76, 134.33, 120.97, 115.06, 111.41, 103.07.

#### 3.1.2. General Procedures for the Synthesis of **4aa**–**4co**

Typical synthesis procedure of amide, taking **4aa** as an example: to a vial (20 mL) was charged THF (5 mL), and then **3a** (0.02 g, 1 mmol) and benzoyl chloride (0.17 mL) and catalytical amount of DMAP. Thus, the reaction mixture was stirred for 6 h. Moreover, the reaction mixture was poured into water (50 mL) and the precipitate was collected by suction. After recrystallization in ethyl acetate, the target molecules could be obtained in 75–92% yield. The detailed characterization of NMR and spectrum are attached as follows:

##### *N*-(2-(Pyridin-2-yl)benzo[*d*]oxazol-5-yl)benzamide (**4aa**)

Yield 65.3%; Pink solid; mp 138.1–138.5 °C; ^1^H NMR (500 MHz, DMSO) δ 10.49 (s, 1H), 8.84 (d, *J* = 4.2 Hz, 1H), 8.39 (d, *J* = 11.0 Hz, 2H), 8.10 (td, *J* = 7.8, 1.4 Hz, 1H), 8.01 (dd, *J* = 27.5, 7.2 Hz, 2H), 7.86 (s, 2H), 7.68–7.64 (m, 2H), 7.61–7.51 (m, 2H).^13^C NMR (126 MHz, DMSO) δ 166.17, 150.71, 147.41, 145.73, 141.85, 138.18, 137.08, 135.37, 132.13, 129.74, 128.92, 128.18, 126.70, 124.10, 120.13, 112.10, 111.49.

##### 2-Phenyl-*N*-(2-(pyridin-2-yl)benzo[*d*]oxazol-5-yl)acetamide (**4ab**)

Yield 37.4%; Pink solid; mp 120.6–120.8 °C; ^1^H NMR (500 MHz, DMSO) δ 10.46 (s, 1H), 8.82 (d, *J* = 4.1 Hz, 1H), 8.35 (d, *J* = 7.9 Hz, 1H), 8.26 (s, 1H), 8.08 (dd, *J* = 11.0, 4.4 Hz, 1H), 7.80 (d, *J* = 8.8 Hz, 1H), 7.67–7.62 (m, 2H), 7.38 (dd, *J* = 13.4, 6.0 Hz, 4H), 7.29 (t, *J* = 7.0 Hz, 1H), 3.72 (s, 2H).^13^C NMR (126 MHz, DMSO) δ 169.70, 162.46, 150.68, 147.05, 145.70, 141.90, 138.15, 137.19, 136.42, 129.65, 128.81, 127.05, 126.67, 124.07, 118.84, 111.62, 110.70, 43.80.

##### 2-Methyl-*N*-(2-(pyridin-2-yl)benzo[*d*]oxazol-5-yl)benzamide (**4ac**)

Yield 56.8%; Pink solid; mp 168.1–168.9 °C; ^1^H NMR (500 MHz, DMSO) δ 10.46 (s, 1H), 8.83 (d, *J* = 4.1 Hz, 1H), 8.38 (d, *J* = 10.0 Hz, 2H), 8.10 (t, *J* = 7.7 Hz, 1H), 7.87–7.82 (m, 4H), 7.69–7.65 (m, 1H), 7.47 (d, *J* = 6.6 Hz, 2H), 2.45 (s, 3H).^13^C NMR (126 MHz, DMSO) δ 166.27, 162.47, 150.71, 147.37, 145.73, 141.83, 138.24, 138.19, 137.13, 135.36, 132.70, 128.82, 128.66, 126.70, 125.34, 124.09, 120.10, 112.05, 111.47, 21.47.

##### 3-Methyl-*N*-(2-(pyridin-2-yl)benzo[*d*]oxazol-5-yl)benzamide (**4ad**)

Yield 26.8%; Pink solid; mp 179.2–179.7 °C; ^1^H NMR (500 MHz, DMSO) δ 10.55 (s, 1H), 8.83 (d, *J* = 4.1 Hz, 1H), 8.44–8.31 (m, 2H), 8.09 (td, *J* = 7.8, 1.5 Hz, 1H), 7.83 (q, *J* = 8.8 Hz, 2H), 7.67 (dd, *J* = 6.6, 4.9 Hz, 1H), 7.54 (d, *J* = 7.2 Hz, 1H), 7.44 (t, *J* = 7.1 Hz, 1H), 7.35 (t, *J* = 7.5 Hz, 2H), 2.45 (s, 3H).^13^C NMR (126 MHz, DMSO) δ 168.41, 162.49, 150.71, 147.29, 145.72, 141.89, 138.18, 137.64, 137.24, 135.78, 131.05, 130.18, 127.75, 126.69, 126.16, 124.09, 119.34, 111.57, 111.26, 19.82.

##### 4-Methyl-*N*-(2-(pyridin-2-yl)benzo[*d*]oxazol-5-yl)benzamide (**4ae**)

Yield 43.4%; Pink solid; mp 212.2–212.8 °C; ^1^H NMR (500 MHz, DMSO) δ 10.45 (s, 1H), 8.83 (d, *J* = 4.1 Hz, 1H), 8.41–8.35 (m, 2H), 8.09 (t, *J* = 7.6 Hz, 1H), 7.96 (d, *J* = 7.9 Hz, 2H), 7.90–7.81 (m, 2H), 7.66 (dd, *J* = 6.9, 5.0 Hz, 1H), 7.38 (d, *J* = 7.9 Hz, 2H), 2.42 (s, 3H).^13^C NMR (126 MHz, DMSO) δ 165.96, 162.43, 150.69, 147.32, 145.73, 142.13, 141.82, 138.16, 137.18, 132.45, 129.42, 128.24, 126.67, 124.07, 120.14, 112.07, 111.41, 21.51.

##### *N*-(2-(Pyridin-2-yl)benzo[*d*]oxazol-5-yl)-2-naphthamide (**4af**)

Yield 37.8%; Pink solid; mp 182.4–182.9 °C; ^1^H NMR (500 MHz, DMSO) δ 10.83 (s, 1H), 8.84 (d, *J* = 4.2 Hz, 1H), 8.71 (s, 1H), 8.47 (d, *J* = 10.5 Hz, 1H), 8.39 (d, *J* = 7.8 Hz, 1H), 8.16–8.08 (m, 4H), 8.05 (d, *J* = 7.7 Hz, 1H), 7.95 (dd, *J* = 8.8, 1.5 Hz, 1H), 7.88 (d, *J* = 8.8 Hz, 1H), 7.72–7.61 (m, 3H).^13^C NMR (126 MHz, DMSO) δ 166.22, 162.48, 150.71, 147.41, 145.74, 141.87, 138.18, 137.24, 134.81, 132.62, 129.49, 128.60, 128.51, 128.17, 127.81, 127.33, 126.69, 126.01, 124.99, 124.09, 120.19, 112.14, 111.50.

##### 4-Methoxy-*N*-(2-(pyridin-2-yl)benzo[*d*]oxazol-5-yl)benzamide (**4ag**)

Yield 70.1%; Pink solid; mp 199.1–199.8 °C; ^1^H NMR (500 MHz, DMSO) δ 10.32 (s, 1H), 8.83 (d, *J* = 4.0 Hz, 1H), 8.38 (d, *J* = 3.7 Hz, 2H), 8.10 (t, *J* = 7.6 Hz, 1H), 8.04 (d, *J* = 8.6 Hz, 2H), 7.84 (s, 2H), 7.67 (dd, *J* = 6.9, 5.1 Hz, 1H), 7.12 (d, *J* = 8.7 Hz, 2H), 3.89 (s, 3H).^13^C NMR (126 MHz, DMSO) δ 165.52, 162.47, 162.43, 150.71, 147.28, 145.75, 141.84, 138.18, 137.26, 130.12, 127.36, 126.68, 124.08, 120.13, 114.15, 112.03, 111.41, 55.95.

##### *N*-(2-(Pyridin-2-yl)benzo[*d*]oxazol-5-yl)-3-(trifluoromethyl)benzamide (**4ah**)

Yield 52.3%; Pink solid; mp 137.9–138.5 °C; ^1^H NMR (500 MHz, DMSO) δ 10.82 (s, 1H), 8.83 (s, 1H), 8.39 (d, *J* = 9.9 Hz, 3H), 8.19 (s, 1H), 8.10 (t, *J* = 6.9 Hz, 1H), 8.01 (d, *J* = 6.9 Hz, 1H), 7.89–7.77 (m, 3H), 7.67–7.62 (m, 1H).^13^C NMR (126 MHz, DMSO) δ 164.66, 162.56, 150.71, 147.60, 145.69, 141.85, 138.18, 136.71, 136.22, 133.34, 132.37, 130.23, 129.22, 128.68, 126.72, 124.80, 124.11, 120.33, 112.43, 111.56.

##### *N*-(2-(Pyridin-2-yl)benzo[*d*]oxazol-5-yl)-4-(trifluoromethyl)benzamide (**4ai**)

Yield 42.7%; Pink solid; mp 146.0–146.6 °C; ^1^H NMR (500 MHz, DMSO) δ 10.70 (s, 1H), 8.83 (d, *J* = 4.2 Hz, 1H), 8.43–8.35 (m, 2H), 8.22 (d, *J* = 8.0 Hz, 2H), 8.08 (tt, *J* = 12.1, 6.0 Hz, 1H), 7.96 (d, *J* = 8.1 Hz, 2H), 7.90–7.85 (m, 2H), 7.66 (dd, *J* = 6.9, 5.1 Hz, 1H).^13^C NMR (126 MHz, DMSO) δ 166.68, 164.97, 162.56, 150.69, 147.59, 145.69, 141.86, 139.15, 138.15, 136.69, 130.58, 129.12, 126.70, 125.88, 124.10, 120.16, 112.26, 111.57.

##### 2-Fluoro-*N*-(2-(pyridin-2-yl)benzo[*d*]oxazol-5-yl)benzamide (**4aj**)

Yield 22.6%; Pink solid; mp 231.2–231.9 °C; ^1^H NMR (500 MHz, DMSO) δ 10.70 (s, 1H), 8.83 (s, 1H), 8.38 (s, 2H), 8.09 (s, 1H), 7.88–7.68 (m, 5H), 7.39 (d, *J* = 7.4 Hz, 2H).^13^C NMR (126 MHz, DMSO) δ 163.37, 162.57, 150.71, 147.44, 145.68, 141.90, 138.19, 136.82, 133.12, 133.06, 130.44, 126.73, 125.08, 124.11, 119.44, 116.78, 116.61, 111.70, 111.42.

##### 3-Fluoro-*N*-(2-(pyridin-2-yl)benzo[*d*]oxazol-5-yl)benzamide (**4ak**)

Yield 62.8%; Pink solid; mp 174.5–175.4 °C; ^1^H NMR (500 MHz, DMSO) δ 8.83 (s, 1H), 8.38 (d, *J* = 11.4 Hz, 2H), 8.09 (t, *J* = 7.1 Hz, 1H), 7.92–7.79 (m, 4H), 7.65 (d, *J* = 6.2 Hz, 2H), 7.52–7.35 (m, 2H).^13^C NMR (126 MHz, DMSO) δ 164.74, 163.42, 162.52, 161.48, 150.70, 147.52, 145.69, 141.84, 138.17, 136.79, 131.06, 126.70, 124.10, 120.20, 118.93, 116.09, 114.94, 112.25, 111.53.

##### 4-Fluoro-*N*-(2-(pyridin-2-yl)benzo[*d*]oxazol-5-yl)benzamide (**4al**)

Yield 23.7%; Pink solid; mp 168.0–168.7 °C; ^1^H NMR (500 MHz, DMSO) δ 10.56 (s, 1H), 8.83 (d, *J* = 4.2 Hz, 1H), 8.37 (d, *J* = 7.5 Hz, 2H), 8.10 (ddd, *J* = 9.1, 8.2, 3.5 Hz, 3H), 7.87–7.80 (m, 2H), 7.66 (dd, *J* = 7.1, 5.0 Hz, 1H), 7.41 (t, *J* = 8.8 Hz, 2H).^13^C NMR (126 MHz, DMSO) δ 165.60, 165.05, 163.62, 162.49, 150.70, 147.43, 145.71, 141.84, 138.17, 136.98, 130.90, 126.69, 124.09, 120.18, 115.76, 112.18, 111.49.

##### 4-Chloro-*N*-(2-(pyridin-2-yl)benzo[*d*]oxazol-5-yl)benzamide (**4am**)

Yield 32.1%; Pink solid; mp 201.9–202.3 °C; ^1^H NMR (500 MHz, DMSO) δ 10.63 (s, 1H), 8.83 (d, *J* = 4.2 Hz, 1H), 8.37 (d, *J* = 9.4 Hz, 2H), 8.11–8.06 (m, 3H), 7.88–7.84 (m, 2H), 7.66 (t, *J* = 7.9 Hz, 3H).^13^C NMR (126 MHz, DMSO) δ 165.04, 162.50, 150.70, 147.48, 145.70, 141.83, 138.17, 136.96, 136.89, 134.03, 130.17, 128.97, 126.70, 124.09, 120.18, 112.21, 111.50.

##### 4-Bromo-*N*-(2-(pyridin-2-yl)benzo[*d*]oxazol-5-yl)benzamide (**4an**)

Yield 43.2%; Pink solid; mp 284.5–285.1 °C; ^1^H NMR (500 MHz, DMSO) δ 10.69 (s, 1H), 8.83 (d, *J* = 4.1 Hz, 1H), 8.39–8.36 (m, 1H), 8.09 (dd, *J* = 10.9, 4.5 Hz, 1H), 8.01 (d, *J* = 8.4 Hz, 2H), 7.86 (s, 2H), 7.78 (d, *J* = 8.4 Hz, 2H), 7.66 (dd, *J* = 7.1, 5.1 Hz, 1H), 7.47 (d, *J* = 8.2 Hz, 1H).^13^C NMR (126 MHz, DMSO) δ 165.17, 162.49, 150.70, 147.47, 145.70, 141.83, 138.17, 136.91, 134.40, 131.90, 130.37, 126.69, 125.88, 124.09, 120.20, 112.22, 111.49.

##### *N*-(2-(Pyridin-2-yl)benzo[*d*]oxazol-5-yl)thiophene-2-carboxamide (**4ao**)

Yield 72.3%; Pink solid; mp 140.3–140.7 °C; ^1^H NMR (500 MHz, DMSO) δ 10.46 (s, 1H), 8.83 (s, 1H), 8.40–8.32 (m, 2H), 8.10 (s, 2H), 7.92–7.79 (m, 3H), 7.67 (s, 1H), 7.28 (s, 1H).^13^C NMR (126 MHz, DMSO) δ 162.53, 160.52, 150.70, 147.47, 145.70, 141.89, 140.37, 138.18, 136.59, 132.44, 129.73, 128.57, 126.71, 124.11, 120.15, 112.21, 111.57.

##### *N*-(2-(Pyridin-3-yl)benzo[*d*]oxazol-5-yl)benzamide (**4ba**)

Yield 36.9%; Pink solid; mp 152.1–152.7 °C; ^1^H NMR (500 MHz, CDCl_3_) δ 10.48 (s, 1H), 9.34 (s, 1H), 8.79 (s, 1H), 8.52 (d, *J* = 6.9 Hz, 1H), 8.32 (s, 1H), 7.97 (d, *J* = 6.3 Hz, 2H), 7.79 (s, 2H), 7.65–7.52 (m, 4H).^13^C NMR (126 MHz, CDCl_3_) δ 165.72, 161.05, 152.39, 147.99, 146.70, 141.37, 136.61, 134.88, 131.69, 128.45, 127.71, 124.31, 122.85, 119.28, 111.43, 111.29, 110.82.

##### 2-Phenyl-*N*-(2-(pyridin-3-yl)benzo[*d*]oxazol-5-yl)acetamide (**4bb**)

Yield 47.2%; Pink solid; mp 110.9–111.6 °C; ^1^H NMR (500 MHz, DMSO) δ 10.48 (s, 1H), 9.36 (s, 1H), 8.83 (s, 1H), 8.55 (d, *J* = 6.8 Hz, 1H), 8.23 (s, 1H), 7.79 (d, *J* = 8.6 Hz, 1H), 7.70–7.58 (m, 2H), 7.41–7.23 (m, 5H), 3.72 (s, 2H).^13^C NMR (126 MHz, DMSO) δ 169.71, 161.50, 152.81, 148.46, 146.82, 141.90, 137.20, 136.40, 135.23, 129.67, 128.83, 127.07, 124.82, 123.30, 118.43, 111.44, 110.41, 43.79.

##### 2-Methyl-*N*-(2-(pyridin-3-yl)benzo[*d*]oxazol-5-yl)benzamide (**4bc**)

Yield 29.8%; Pink solid; mp 158.1–158.8 °C; ^1^H NMR (500 MHz, DMSO) δ 10.56 (s, 1H), 9.41 (s, 1H), 8.87 (s, 1H), 8.63 (d, *J* = 6.9 Hz, 1H), 8.37 (s, 1H), 7.85–7.73 (m, 3H), 7.54 (d, *J* = 6.8 Hz, 1H), 7.44 (d, *J* = 7.0 Hz, 1H), 7.36 (d, *J* = 7.3 Hz, 2H), 2.45 (s, 3H).^13^C NMR (126 MHz, DMSO) δ 168.41, 161.29, 152.17, 147.92, 147.05, 141.86, 137.60, 137.28, 135.92, 135.76, 131.05, 130.21, 127.75, 126.17, 125.12, 123.57, 119.01, 111.42, 110.98, 19.82.

##### 3-Methyl-*N*-(2-(pyridin-3-yl)benzo[*d*]oxazol-5-yl)benzamide (**4bd**)

Yield 36.8%; Pink solid; mp 179.1–179.7 °C; ^1^H NMR (500 MHz, DMSO) δ 10.55 (s, 1H), 9.39 (s, 1H), 8.84 (s, 1H), 8.57 (d, *J* = 7.2 Hz, 1H), 8.38 (s, 1H), 7.85 (s, 3H), 7.70 (d, *J* = 14.2 Hz, 2H), 7.45 (s, 1H), 7.17 (d, *J* = 20.4 Hz, 1H), 2.44 (s, 3H).^13^C NMR (126 MHz, DMSO) δ 166.28, 161.48, 152.81, 148.47, 147.11, 141.83, 138.22, 137.17, 135.22, 132.68, 130.21, 128.80, 128.67, 125.35, 124.82, 123.33, 119.72, 111.79, 111.25, 21.47.

##### 4-Methyl-*N*-(2-(pyridin-3-yl)benzo[*d*]oxazol-5-yl)benzamide (**4be**)

Yield 73.5%; Pink solid; mp 186.4–186.9 °C; ^1^H NMR (500 MHz, DMSO) δ 10.45 (s, 1H), 9.39 (s, 1H), 8.84 (s, 1H), 8.57 (d, *J* = 7.2 Hz, 1H), 8.37 (s, 1H), 7.95 (d, *J* = 7.3 Hz, 2H), 7.84 (s, 2H), 7.69 (s, 1H), 7.39 (d, *J* = 7.4 Hz, 2H), 3.37 (s, 3H).^13^C NMR (126 MHz, DMSO) δ 165.96, 161.47, 152.81, 148.47, 147.09, 142.15, 141.83, 137.17, 135.22, 132.44, 129.43, 128.23, 124.82, 123.33, 119.75, 111.80, 111.23, 21.52.

##### *N*-(2-(Pyridin-3-yl)benzo[*d*]oxazol-5-yl)-2-naphthamide (**4bf**)

Yield 37.8%; Pink solid; mp 182.4–182.9 °C; ^1^H NMR (500 MHz, DMSO) δ 10.69 (s, 1H), 9.40 (s, 1H), 8.84 (s, 1H), 8.66 (s, 1H), 8.58 (d, *J* = 7.6 Hz, 1H), 8.43 (s, 1H), 8.10 (s, 4H), 7.88 (s, 2H), 7.68 (s, 3H).^13^C NMR (126 MHz, DMSO) δ 166.20, 161.53, 152.82, 148.49, 147.18, 141.88, 137.15, 135.23, 134.80, 132.66, 132.58, 129.46, 128.55, 128.36, 128.18, 127.38, 124.94, 124.82, 123.33, 119.74, 119.64, 111.84, 111.33.

##### 4-Methoxy-*N*-(2-(pyridin-3-yl)benzo[*d*]oxazol-5-yl)benzamide (**4bg**)

Yield 55.3%; Pink solid; mp 204.9–205.3 °C; ^1^H NMR (500 MHz, DMSO) δ 10.34 (s, 1H), 9.38 (s, 1H), 8.83 (s, 1H), 8.56 (d, *J* = 6.8 Hz, 1H), 8.35 (s, 1H), 8.02 (s, 2H), 7.82 (s, 2H), 7.68 (s, 1H), 7.10 (s, 2H), 3.88 (s, 3H).^13^C NMR (126 MHz, DMSO) δ 165.50, 162.43, 161.43, 152.78, 148.46, 147.02, 141.82, 137.26, 135.19, 130.12, 127.33, 124.80, 123.33, 119.72, 114.12, 111.76, 111.18, 55.93.

##### *N*-(2-(Pyridin-3-yl)benzo[*d*]oxazol-5-yl)-3-(trifluoromethyl)benzamide (**4bh**)

Yield 72.6%; Pink solid; mp 127.3–127.8 °C; ^1^H NMR (500 MHz, DMSO) δ 10.73 (s, 1H), 9.39 (s, 1H), 8.85 (s, 1H), 8.59 (s, 1H), 8.36 (s, 2H), 8.02 (d, *J* = 7.4 Hz, 1H), 7.90–7.81 (m, 4H), 7.70 (s, 1H).^13^C NMR (126 MHz, DMSO) δ 164.65, 161.61, 152.86, 148.50, 147.36, 141.87, 138.72, 136.72, 136.24, 135.26, 132.37, 130.26, 129.82, 129.56, 128.68, 124.83, 123.29, 119.95, 112.15, 111.38.

##### *N*-(2-(Pyridin-3-yl)benzo[*d*]oxazol-5-yl)-4-(trifluoromethyl)benzamide (**4bi**)

Yield 42.7%; Pink solid; mp 179.8–180.4 °C; ^1^H NMR (500 MHz, DMSO) δ 10.72 (s, 1H), 9.39 (s, 1H), 8.84 (s, 1H), 8.56 (s, 1H), 8.37 (s, 1H), 8.22 (d, *J* = 7.6 Hz, 2H), 7.97 (d, *J* = 7.6 Hz, 2H), 7.85 (d, *J* = 8.8 Hz, 2H), 7.69 (s, 1H).^13^C NMR (126 MHz, DMSO) δ 164.99, 161.61, 152.85, 148.49, 147.35, 141.86, 139.15, 136.70, 135.24, 132.03, 131.78, 129.12, 125.90, 124.82, 123.28, 119.78, 111.98, 111.39.

##### 2-Fluoro-*N*-(2-(pyridin-3-yl)benzo[*d*]oxazol-5-yl)benzamide (**4bj**)

Yield 32.6%; Pink solid; mp 208.6–209.1 °C; ^1^H NMR (500 MHz, DMSO) δ 10.68 (s, 1H), 9.39 (s, 1H), 8.84 (s, 1H), 8.57 (d, *J* = 6.9 Hz, 1H), 8.34 (s, 1H), 7.85 (d, *J* = 8.5 Hz, 1H), 7.77–7.63 (m, 4H), 7.40 (dd, *J* = 19.0, 8.9 Hz, 2H).^13^C NMR (126 MHz, DMSO) δ 163.35, 161.62, 160.37, 152.86, 148.50, 147.20, 141.91, 136.81, 135.25, 133.07, 130.42, 125.12, 124.82, 123.28, 119.02, 116.79, 116.62, 111.51, 111.12.

##### 3-Fluoro-*N*-(2-(pyridin-3-yl)benzo[*d*]oxazol-5-yl)benzamide (**4bk**)

Yield 42.8%; Pink solid; mp 164.4–164.9 °C; ^1^H NMR (500 MHz, DMSO) δ 10.60 (s, 1H), 9.38 (s, 1H), 8.83 (s, 1H), 8.56 (d, *J* = 7.1 Hz, 1H), 8.36 (s, 1H), 7.90–7.82 (m, 4H), 7.65 (d, *J* = 24.3 Hz, 2H), 7.49 (s, 1H).^13^C NMR (126 MHz, DMSO) δ 164.73, 163.39, 161.54, 152.81, 148.47, 147.27, 141.83, 137.65, 136.78, 135.21, 131.12, 124.79, 124.42, 123.28, 119.78, 119.10, 115.11, 111.95, 111.31.

##### 4-Fluoro-*N*-(2-(pyridin-3-yl)benzo[*d*]oxazol-5-yl)benzamide (**4bl**)

Yield 23.7%; Pink solid; mp 174.5–175.1 °C; ^1^H NMR (500 MHz, DMSO) δ 10.54 (s, 1H), 9.38 (s, 1H), 8.83 (s, 1H), 8.56 (d, *J* = 7.0 Hz, 1H), 8.35 (s, 1H), 8.12 (s, 2H), 7.83 (s, 2H), 7.68 (s, 1H), 7.42 (s, 2H).^13^C NMR (126 MHz, DMSO) δ 165.02, 163.60, 161.50, 152.78, 148.45, 147.18, 141.82, 136.97, 135.23, 131.72, 130.91, 124.81, 123.31, 119.78, 115.93, 111.91, 111.26.

##### 4-Chloro-*N*-(2-(pyridin-3-yl)benzo[*d*]oxazol-5-yl)benzamide (**4bm**)

Yield 62.5%; Pink solid; mp 211.9–211.3 °C; ^1^H NMR (500 MHz, DMSO) δ 10.62 (s, 1H), 9.39 (d, *J* = 1.4 Hz, 1H), 8.86–8.83 (m, 1H), 8.58–8.56 (m, 1H), 8.36 (s, 1H), 8.07 (d, *J* = 8.5 Hz, 2H), 7.91 (d, *J* = 8.4 Hz, 1H), 7.84 (s, 1H), 7.70–7.66 (m, 2H), 7.41 (d, *J* = 8.3 Hz, 1H).^13^C NMR (126 MHz, DMSO) δ 165.07, 161.57, 152.84, 148.50, 147.28, 141.87, 135.24, 134.05, 131.39, 130.18, 128.99, 128.05, 124.82, 123.33, 119.84, 111.99, 111.31.

##### 4-Bromo-*N*-(2-(pyridin-3-yl)benzo[*d*]oxazol-5-yl)benzamide (**4bn**)

Yield 27.6%; Pink solid; mp 263.0–263.8 °C; ^1^H NMR (500 MHz, DMSO) δ 10.57 (s, 1H), 9.38 (s, 1H), 8.84 (s, 1H), 8.56 (d, *J* = 7.0 Hz, 1H), 8.35 (s, 1H), 7.98 (d, *J* = 7.3 Hz, 2H), 7.82 (d, *J* = 13.6 Hz, 4H), 7.68 (s, 1H).^13^C NMR (126 MHz, DMSO) δ 165.13, 161.54, 152.82, 148.48, 147.23, 141.84, 136.86, 135.22, 134.38, 131.92, 130.32, 125.90, 124.80, 123.29, 119.76, 111.91, 111.31.

##### *N*-(2-(Pyridin-3-yl)benzo[*d*]oxazol-5-yl)thiophene-2-carboxamide (**4bo**)

Yield 42.3%; Pink solid; mp 161.2–161.8 °C; ^1^H NMR (500 MHz, DMSO) δ 10.49 (s, 1H), 9.39 (s, 1H), 8.84 (s, 1H), 8.56 (s, 1H), 8.30 (s, 1H), 8.10 (s, 1H), 7.92 (s, 1H), 7.85 (d, *J* = 8.6 Hz, 1H), 7.78 (d, *J* = 8.7 Hz, 1H), 7.69 (s, 1H), 7.28 (s, 1H); ^13^C NMR (126 MHz, DMSO) δ 161.56, 160.50, 152.83, 148.49, 147.22, 141.88, 140.34, 136.59, 135.24, 132.46, 129.73, 128.58, 124.81, 123.30, 119.77, 111.94, 111.36.

##### *N*-(2-(Pyridin-4-yl)benzo[*d*]oxazol-5-yl)benzamide (**4ca**)

Yield 56.2%; Pink solid; mp 132.1–132.6 °C; ^1^H NMR (500 MHz, DMSO) δ 10.50 (s, 1H), 8.88 (d, *J* = 5.8 Hz, 2H), 8.40 (s, 1H), 8.17–8.10 (m, 2H), 8.03 (d, *J* = 7.2 Hz, 2H), 7.87 (s, 2H), 7.61 (dt, *J* = 12.2, 5.9 Hz, 3H).^13^C NMR (126 MHz, DMSO) δ 166.21, 161.42, 151.37, 147.29, 141.80, 137.26, 135.33, 133.97, 132.16, 128.93, 128.18, 121.28, 120.44, 112.05, 111.51.

##### 2-Phenyl-*N*-(2-(pyridin-4-yl)benzo[*d*]oxazol-5-yl)acetamide (**4cb**)

Yield 67.7%; Pink solid; mp 120.5–120.9 °C; ^1^H NMR (500 MHz, DMSO) δ 10.52 (s, 1H), 8.86 (d, *J* = 6.1 Hz, 2H), 8.27 (s, 1H), 8.11 (d, *J* = 6.1 Hz, 2H), 7.82 (d, *J* = 8.9 Hz, 1H), 7.65 (d, *J* = 10.9 Hz, 1H), 7.42–7.34 (m, 4H), 7.29 (d, *J* = 7.0 Hz, 1H), 3.72 (s, 2H).^13^C NMR (126 MHz, DMSO) δ 169.76, 161.39, 151.37, 146.93, 141.85, 137.39, 136.38, 133.94, 129.67, 128.82, 127.06, 121.26, 119.15, 111.68, 110.62, 43.78.

##### 2-Methyl-*N*-(2-(pyridin-4-yl)benzo[*d*]oxazol-5-yl)benzamide (**4cc**)

Yield 22.3%; Pink solid; mp 151.6–152.0 °C; ^1^H NMR (500 MHz, DMSO) δ 10.57 (s, 1H), 8.87 (s, 2H), 8.40 (s, 1H), 8.13 (d, *J* = 5.9 Hz, 2H), 7.84 (dd, *J* = 20.2, 8.8 Hz, 2H), 7.54 (d, *J* = 7.4 Hz, 1H), 7.44 (t, *J* = 7.2 Hz, 1H), 7.36 (t, *J* = 7.7 Hz, 2H), 1.94 (s, 3H).^13^C NMR (126 MHz, DMSO) δ 172.49, 168.44, 161.43, 151.40, 147.16, 141.84, 137.41, 135.78, 133.95, 131.07, 130.23, 127.75, 126.18, 121.27, 119.63, 111.64, 111.18, 21.55.

##### 3-Methyl-*N*-(2-(pyridin-4-yl)benzo[*d*]oxazol-5-yl)benzamide (**4cd**)

Yield 26.9%; Pink solid; mp 189.1–189.7 °C; ^1^H NMR (500 MHz, DMSO) δ 10.47 (s, 1H), 8.88 (d, *J* = 6.0 Hz, 2H), 8.40 (s, 1H), 8.13 (d, *J* = 6.0 Hz, 2H), 7.87–7.79 (m, 4H), 7.50–7.41 (m, 2H), 2.45 (s, 3H).^13^C NMR (126 MHz, DMSO) δ 166.31, 161.41, 151.39, 147.24, 141.79, 138.26, 137.30, 135.34, 133.96, 132.74, 128.84, 128.66, 125.34, 121.27, 120.38, 111.98, 111.52, 21.47.

##### 4-Methyl-*N*-(2-(pyridin-4-yl)benzo[*d*]oxazol-5-yl)benzamide (**4ce**)

Yield 73.2%; Pink solid; mp 193.6–194.0 °C; ^1^H NMR (500 MHz, DMSO) δ 10.44 (s, 1H), 8.87 (d, *J* = 6.0 Hz, 2H), 8.40 (s, 1H), 8.13 (d, *J* = 6.0 Hz, 2H), 7.95 (d, *J* = 8.1 Hz, 2H), 7.86 (s, 2H), 7.39 (d, *J* = 7.9 Hz, 2H), 2.43 (s, 3H).^13^C NMR (126 MHz, DMSO) δ 166.00, 161.38, 151.38, 147.21, 142.19, 141.78, 137.34, 133.97, 132.41, 129.45, 128.23, 121.27, 120.42, 111.99, 111.48, 21.52.

##### *N*-(2-(Pyridin-4-yl)benzo[*d*]oxazol-5-yl)-2-naphthamide (**4cf**)

Yield 37.8%; Pink solid; mp 178.6–178.9 °C; ^1^H NMR (500 MHz, DMSO) δ 10.69 (s, 1H), 8.88 (d, *J* = 5.9 Hz, 2H), 8.65 (s, 1H), 8.46 (d, *J* = 1.3 Hz, 1H), 8.16–8.03 (m, 6H), 7.91 (dt, *J* = 18.0, 5.3 Hz, 2H), 7.71–7.64 (m, 2H).^13^C NMR (126 MHz, DMSO) δ 166.24, 161.43, 151.35, 147.32, 141.84, 137.33, 134.83, 134.00, 132.63, 132.60, 129.46, 128.80, 128.56, 128.37, 128.19, 127.38, 124.93, 121.29, 120.46, 112.08, 111.55.

##### 4-Methoxy-*N*-(2-(pyridin-4-yl)benzo[*d*]oxazol-5-yl)benzamide (**4cg**)

Yield 35.3%; Pink solid; mp 194.9–195.4 °C; ^1^H NMR (500 MHz, DMSO) δ 10.34 (s, 1H), 8.88 (d, *J* = 5.7 Hz, 2H), 8.39 (s, 1H), 8.17–8.11 (m, 2H), 8.03 (d, *J* = 8.8 Hz, 2H), 7.86 (s, 2H), 7.12 (d, *J* = 8.8 Hz, 2H), 3.88 (s, 3H).^13^C NMR (126 MHz, DMSO) δ 165.55, 162.49, 161.37, 151.38, 147.17, 141.79, 137.45, 133.99, 130.13, 127.31, 121.27, 120.44, 114.15, 111.99, 111.44, 55.95.

##### *N*-(2-(Pyridin-4-yl)benzo[*d*]oxazol-5-yl)-3-(trifluoromethyl)benzamide (**4ch**)

Yield 65.6%; Pink solid; mp 138.3–138.9 °C; ^1^H NMR (500 MHz, DMSO) δ 10.75 (s, 1H), 8.88 (d, *J* = 5.9 Hz, 2H), 8.36 (dd, *J* = 21.3, 13.1 Hz, 3H), 8.14 (d, *J* = 5.9 Hz, 2H), 8.03 (d, *J* = 7.7 Hz, 1H), 7.86 (dd, *J* = 20.6, 12.0 Hz, 3H).^13^C NMR (126 MHz, DMSO) δ 164.70, 161.53, 154.77, 151.40, 147.49, 141.81, 136.84, 136.15, 133.93, 132.38, 130.29, 129.84, 128.80, 124.81, 121.30, 120.63, 112.37, 111.65.

##### *N*-(2-(Pyridin-4-yl)benzo[*d*]oxazol-5-yl)-4-(trifluoromethyl)benzamide (**4ci**)

Yield 42.7%; Pink solid; mp 189.8–190.4 °C; ^1^H NMR (500 MHz, DMSO) δ 10.71 (s, 1H), 8.86 (d, *J* = 6.0 Hz, 2H), 8.39 (s, 1H), 8.21 (d, *J* = 8.1 Hz, 2H), 8.11 (d, *J* = 6.0 Hz, 2H), 7.95 (d, *J* = 8.3 Hz, 2H), 7.86 (s, 2H).^13^C NMR (126 MHz, DMSO) δ 164.99, 161.48, 151.35, 147.44, 141.78, 139.09, 136.86, 133.89, 132.06, 129.12, 125.89, 123.32, 121.25, 120.43, 112.17, 111.60.

##### 2-Fluoro-*N*-(2-(pyridin-4-yl)benzo[*d*]oxazol-5-yl)benzamide (**4cj**)

Yield 47.8%; Pink solid; mp 221.2–221.5 °C; ^1^H NMR (500 MHz, DMSO) δ 10.69 (s, 1H), 8.88 (d, *J* = 5.5 Hz, 2H), 8.38 (s, 1H), 8.14 (d, *J* = 5.6 Hz, 2H), 7.88 (d, *J* = 8.8 Hz, 1H), 7.82–7.73 (m, 2H), 7.64 (d, *J* = 6.6 Hz, 1H), 7.40 (dd, *J* = 19.6, 8.8 Hz, 2H).^13^C NMR (126 MHz, DMSO) δ 163.39, 161.54, 151.41, 147.33, 141.86, 136.98, 133.92, 133.18, 130.45, 125.42, 125.11, 121.29, 119.72, 116.81, 116.63, 111.77, 111.35.

##### 3-Fluoro-*N*-(2-(pyridin-4-yl)benzo[*d*]oxazol-5-yl)benzamide (**4ck**)

Yield 32.8%; Pink solid; mp 163.3–163.8 °C; ^1^H NMR (500 MHz, DMSO) δ 10.67 (s, 1H), 8.88 (d, *J* = 4.1 Hz, 2H), 8.38 (s, 1H), 8.14 (d, *J* = 4.2 Hz, 2H), 7.88 (d, *J* = 8.7 Hz, 1H), 7.81–7.73 (m, 2H), 7.64 (d, *J* = 5.8 Hz, 1H), 7.40 (dd, *J* = 17.4, 9.2 Hz, 2H).^13^C NMR (126 MHz, DMSO) δ 163.39, 161.54, 151.40, 147.35, 141.87, 136.98, 133.93, 133.17, 133.10, 130.43, 125.12, 121.29, 119.75, 116.80, 116.62, 111.75, 111.39.

##### 4-Fluoro-*N*-(2-(pyridin-4-yl)benzo[*d*]oxazol-5-yl)benzamide (**4cl**)

Yield 23.7%; Pink solid; mp 154.6–155.1 °C; ^1^H NMR (500 MHz, DMSO) δ 10.54 (s, 1H), 8.88 (d, *J* = 5.9 Hz, 2H), 8.39 (s, 1H), 8.15–8.09 (m, 4H), 7.87 (d, *J* = 7.4 Hz, 2H), 7.43 (t, *J* = 8.8 Hz, 2H).^13^C NMR (126 MHz, DMSO) δ 165.08, 161.44, 151.39, 147.31, 141.79, 137.13, 133.94, 131.73, 130.90, 121.27, 120.47, 115.97, 115.79, 112.11, 111.55.

##### 4-Chloro-*N*-(2-(pyridin-4-yl)benzo[*d*]oxazol-5-yl)benzamide (**4cm**)

Yield 34.5%; Pink solid; mp 216.5–217.0 °C; ^1^H NMR (500 MHz, DMSO) δ 10.60 (s, 1H), 8.87 (d, *J* = 4.5 Hz, 2H), 8.38 (s, 1H), 8.12 (d, *J* = 5.8 Hz, 2H), 8.05 (d, *J* = 8.5 Hz, 2H), 7.86 (s, 2H), 7.66 (d, *J* = 8.5 Hz, 2H).^13^C NMR (126 MHz, DMSO) δ 165.07, 161.45, 151.37, 147.37, 141.80, 137.08, 136.99, 134.02, 133.94, 130.16, 128.99, 121.27, 120.50, 112.18, 111.53.

##### 4-Bromo-*N*-(2-(pyridin-4-yl)benzo[*d*]oxazol-5-yl)benzamide (**4cn**)

Yield 17.6%; Pink solid; mp 268.1–268.7 °C; ^1^H NMR (500 MHz, DMSO) δ 10.55 (s, 1H), 8.86 (s, 2H), 8.37 (s, 1H), 8.11 (d, *J* = 5.7 Hz, 2H), 7.97 (d, *J* = 8.4 Hz, 2H), 7.86–7.77 (m, 4H).^13^C NMR (126 MHz, DMSO) δ 165.17, 161.43, 151.36, 147.34, 141.78, 137.01, 134.33, 133.91, 131.93, 130.32, 125.95, 121.26, 120.42, 112.11, 111.54.

##### *N*-(2-(Pyridin-4-yl)benzo[*d*]oxazol-5-yl)thiophene-2-carboxamide (**4co**)

Yield 62.8%; Pink solid; mp 162.4–162.8 °C; ^1^H NMR (500 MHz, DMSO) δ 10.48 (s, 1H), 8.88 (d, *J* = 6.0 Hz, 2H), 8.34 (s, 1H), 8.14 (d, *J* = 4.5 Hz, 2H), 8.09 (d, *J* = 3.6 Hz, 1H), 7.90 (dd, *J* = 18.6, 7.3 Hz, 2H), 7.81 (d, *J* = 8.9 Hz, 1H), 7.31–7.27 (m, 1H).^13^C NMR (126 MHz, DMSO) δ 161.49, 160.54, 151.40, 147.36, 141.84, 140.29, 136.76, 133.95, 132.53, 129.77, 128.60, 121.30, 120.47, 112.17, 111.63.

#### 3.1.3. Structure Determination

The crystal structures of **4ac**–**4bc** were determined by single-crystal X-ray diffraction [27]. A total of 100 mg of the compound **4ac**–**4bc** was dissolved in 3 mL of ethyl acetate, and the solution was placed in a 25 mL micro-reaction bottle. The reaction bottle was placed in a dryer containing petroleum ether and the dryer was sealed. After 3 days, the reaction bottle was taken out, and the single-crystal culture was completed. Reflection data were collected at room temperature on a Bruker APEX II area detector diffractometer equipped with graphite-monochromatic MoK radiation (λ = 0.71073A°) at 296(2)K with ω–2θ scan mode. Empirical adsorption corrections were applied to all data using SADABS. The structures were solved by direct methods and refined by full matrix least squares on *F_2_* using *SHELXTL 97* software. All non-hydrogen atoms were located by direct methods and subsequent difference Fourier syntheses. The hydrogen atoms bound to carbon were located by geometrical calculations, and their positions and thermal parameters were fixed during the structure refinement in **4ac**–**4bc**. Crystallographic data and pertinent information are provided in Supplementary Materials.

### 3.2. Anti-Fungal Activity

Herein, the antifungal activities of these compounds were evaluated against 8 common phytopathogenic fungi, (*Fusarium solani*, *Colletotrichum gloeosporioides*, *Mycosphaerella melonis melonis*, *Alternaria brassicae*, *Pyricularia grisea*, *Curvularia lunata*, *Alternaria solani*, and *Fusarium graminearum*) using inhibition zone method [28] at the concentration of 100 μg/mL by poisoned food technique. Inoculate the potato dextrose agar (PDA) plate: the potato dextrose agar (PDA) culture solution without compounds to be tested (blank controls) was poured in the petri dish evenly, until it became cold and solidified. In the same way, the potato dextrose agar (PDA) culture solution of different concentrations with the compound to be tested was poured into the petri dish evenly until it became cold and solidified. Then colonies from 24-h-old potato dextrose agar (PDA) petri dishes were inoculated on each cold petri dish by sterile drill to drill holes (5 mm). Among them, the concentration of the stock solutions for in vitro studies was 400 μg/mL and the amount of DMSO must be less than 2% of the overall solution volume. As a positive control, thiophanate-methyl (a commercially available agricultural fungicide) was used, the solution without the test compound was used as a blank control. Moreover, then petri dishes were incubated for 5–7 days under suitable cultivation conditions in the light incubator. All experiments were independently repeated three times.

## 4. Conclusions

In conclusion, a series of benzoxazoles were synthesized efficiently using a combination of PPA and Raney nickel reduction. On the basis of syntheses, their anti-phytopathogenic activities were investigated as well. All the synthesized coumarin derivatives were characterized by NMR and MS. The single crystal of **4ac**, **4bc** were obtained and characterized by X-ray. These compounds showed moderate to good excellent inhibition rate using linear growth rate method. These results will not only be helpful in the synthesis of similar heterocycles, but also will enlighten the discovery of a new kind of agrochemicals in the future.

## Figures and Tables

**Table 1:** The syntheses of amide derivatives of benzoxazoles ^a^.

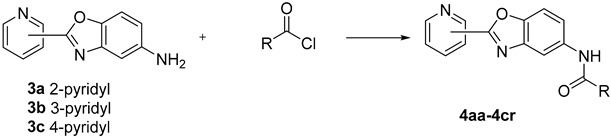				
Entry	2-Pyridyl	3-Pyridyl	4-Pyridyl	R*^a^*
1	**4aa**	**4ba**	**4ca**	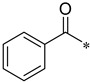
2	**4ab**	**4bb**	**4cb**	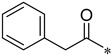
3	**4ac**	**4bc**	**4cc**	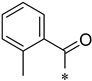
4	**4ad**	**4bd**	**4cd**	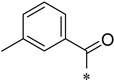
5	**4ae**	**4be**	**4ce**	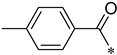
6	**4af**	**4bf**	**4cf**	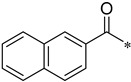
7	**4ag**	**4bg**	**4cg**	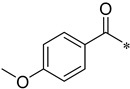
8	**4ah**	**4bh**	**4ch**	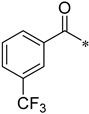
9	**4ai**	**4bi**	**4ci**	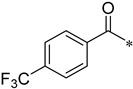
10	**4aj**	**4bj**	**4cj**	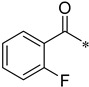
11	**4ak**	**4bk**	**4ck**	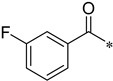
12	**4al**	**4bl**	**4cl**	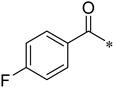
13	**4am**	**4bm**	**4cm**	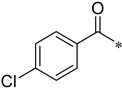
14	**4an**	**4bn**	**4cn**	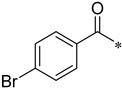
15	**4ao**	**4bo**	**4co**	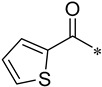

^a^ Reaction condition: each entry was performed at 3 mmol scale, in the presence of DMAP, in THF, r.t., 6 h.

## Data Availability

Not applicable.

## Supplementary Materials

The following supporting information can be downloaded at: https://www.mdpi.com/article/10.3390/molecules27238375/s1, supplementary data associated with this article can be found in the online version at doi.

## Sample Availability

Samples of the all the tested compounds **4aa**–**4co** are available from the authors.
