# Supplementary material for: Design, Synthesis, and Anti-Fungal Evaluation of Heterocyclic Benzoxazole Derivatives
Source: Molecules. 2022 Nov 30;27(23):8375. doi: 10.3390/molecules27238375 (PMC9739801; doi:10.3390/molecules27238375)
Supplement: Supplementary file 1 [file molecules-27-08375-s001.zip › molecules-2022407-supplementary.pdf]

SI of SYNTHESIS AND PHARMACOLOGICAL ACTION OF HETEROCYCLIC BENZOXAZOLE DERIVATIVES

_N_-(2-(pyridin-2-yl)benzo[_d_]oxazol-5-yl)benzamide(4aa)

^{1}H NMR (500 MHz, DMSO) δ 10.49 (s, 1H), 8.84 (d, *J* = 4.2 Hz, 1H), 8.39 (d, *J* = 11.0 Hz, 2H), 8.10 (td, *J* = 7.8, 1.4 Hz, 1H), 8.01 (dd, *J* = 27.5, 7.2 Hz, 2H), 7.86 (s, 2H), 7.68 – 7.64 (m, 2H), 7.61 – 7.51 (m, 2H).^{13}C NMR (126 MHz, DMSO) δ 166.17, 150.71, 147.41, 145.73, 141.85, 138.18, 137.08, 135.37, 132.13, 129.74, 128.92, 128.18, 126.70, 124.10, 120.13, 112.10, 111.49.

2-phenyl-_N_-(2-(pyridin-2-yl)benzo[_d_]oxazol-5-yl)acetamide(4ab)

^{1}H NMR (500 MHz, DMSO) δ 10.46 (s, 1H), 8.82 (d, *J* = 4.1 Hz, 1H), 8.35 (d, *J* = 7.9 Hz, 1H), 8.26 (s, 1H), 8.08 (dd, *J* = 11.0, 4.4 Hz, 1H), 7.80 (d, *J* = 8.8 Hz, 1H), 7.67 – 7.62 (m, 2H), 7.38 (dd, *J* = 13.4, 6.0 Hz, 4H), 7.29 (t, *J* = 7.0 Hz, 1H), 3.72 (s, 2H).^{13}C NMR (126 MHz, DMSO) δ 169.70, 162.46, 150.68, 147.05, 145.70, 141.90, 138.15, 137.19, 136.42, 129.65, 128.81, 127.05, 126.67, 124.07, 118.84, 111.62, 110.70, 43.80.

2-methyl-_N_-(2-(pyridin-2-yl)benzo[_d_]oxazol-5-yl)benzamide(4ac)

^{1}H NMR (500 MHz, DMSO) δ 10.46 (s, 1H), 8.83 (d, *J* = 4.1 Hz, 1H), 8.38 (d, *J* = 10.0 Hz, 2H), 8.10 (t, *J* = 7.7 Hz, 1H), 7.87 – 7.82 (m, 4H), 7.69 – 7.65 (m, 1H), 7.47 (d, *J* = 6.6 Hz, 2H), 2.45 (s, 3H).^{13}C NMR (126 MHz, DMSO) δ 166.27, 162.47, 150.71, 147.37, 145.73, 141.83, 138.24, 138.19, 137.13, 135.36, 132.70, 128.82, 128.66, 126.70, 125.34, 124.09, 120.10, 112.05, 111.47, 21.47.

3-methyl-_N_-(2-(pyridin-2-yl)benzo[_d_]oxazol-5-yl)benzamide(4ad)

^{1}H NMR (500 MHz, DMSO) δ 10.55 (s, 1H), 8.83 (d, *J* = 4.1 Hz, 1H), 8.44 – 8.31 (m, 2H), 8.09 (td, *J* = 7.8, 1.5 Hz, 1H), 7.83 (q, *J* = 8.8 Hz, 2H), 7.67 (dd, *J* = 6.6, 4.9 Hz, 1H), 7.54 (d, *J* = 7.2 Hz, 1H), 7.44 (t, *J* = 7.1 Hz, 1H), 7.35 (t, *J* = 7.5 Hz, 2H), 2.45 (s, 3H).^{13}C NMR (126 MHz, DMSO) δ 168.41, 162.49, 150.71, 147.29, 145.72, 141.89, 138.18, 137.64, 137.24, 135.78, 131.05, 130.18, 127.75, 126.69, 126.16, 124.09, 119.34, 111.57, 111.26, 19.82.

4-methyl-_N_-(2-(pyridin-2-yl)benzo[_d_]oxazol-5-yl)benzamide(4ae)

^{1}H NMR (500 MHz, DMSO) δ 10.45 (s, 1H), 8.83 (d, *J* = 4.1 Hz, 1H), 8.41 – 8.35 (m, 2H), 8.09 (t, *J* = 7.6 Hz, 1H), 7.96 (d, *J* = 7.9 Hz, 2H), 7.90 – 7.81 (m, 2H), 7.66 (dd, *J* = 6.9, 5.0 Hz, 1H), 7.38 (d, *J* = 7.9 Hz, 2H), 2.42 (s, 3H).^{13}C NMR (126 MHz, DMSO) δ 165.96, 162.43, 150.69, 147.32, 145.73, 142.13, 141.82, 138.16, 137.18, 132.45, 129.42, 128.24, 126.67, 124.07, 120.14, 112.07, 111.41, 21.51.

***N*-(2-(pyridin-2-yl)benzo[*d*]oxazol-5-yl)-2-naphthamide(4af)**

^{1}H NMR (500 MHz, DMSO) δ 10.83 (s, 1H), 8.84 (d, *J* = 4.2 Hz, 1H), 8.71 (s, 1H), 8.47 (d, *J* = 10.5 Hz, 1H), 8.39 (d, *J* = 7.8 Hz, 1H), 8.16 – 8.08 (m, 4H), 8.05 (d, *J* = 7.7 Hz, 1H), 7.95 (dd, *J* = 8.8, 1.5 Hz, 1H), 7.88 (d, *J* = 8.8 Hz, 1H), 7.72 – 7.61 (m, 3H).^{13}C NMR (126 MHz, DMSO) δ 166.22, 162.48, 150.71, 147.41, 145.74, 141.87, 138.18, 137.24, 134.81, 132.62, 129.49, 128.60, 128.51, 128.17, 127.81, 127.33, 126.69, 126.01, 124.99, 124.09, 120.19, 112.14, 111.50.

4-methoxy-*N*-(2-(pyridin-2-yl)benzo[*d*]oxazol-5-yl)benzamide(4ag)

^{1}H NMR (500 MHz, DMSO) δ 10.32 (s, 1H), 8.83 (d, *J* = 4.0 Hz, 1H), 8.38 (d, *J* = 3.7 Hz, 2H), 8.10 (t, *J* = 7.6 Hz, 1H), 8.04 (d, *J* = 8.6 Hz, 2H), 7.84 (s, 2H), 7.67 (dd, *J* = 6.9, 5.1 Hz, 1H), 7.12 (d, *J* = 8.7 Hz, 2H), 3.89 (s, 3H).^{13}C NMR (126 MHz, DMSO) δ 165.52, 162.47, 162.43, 150.71, 147.28, 145.75, 141.84, 138.18, 137.26, 130.12, 127.36, 126.68, 124.08, 120.13, 114.15, 112.03, 111.41, 55.95.

***N*-(2-(pyridin-2-yl)benzo[*d*]oxazol-5-yl)-3-(trifluoromethyl)benzamide(4ah)**

^{1}H NMR (500 MHz, DMSO) δ 10.82 (s, 1H), 8.83 (s, 1H), 8.39 (d, *J* = 9.9 Hz, 3H), 8.19 (s, 1H), 8.10 (t, *J* = 6.9 Hz, 1H), 8.01 (d, *J* = 6.9 Hz, 1H), 7.89 – 7.77 (m, 3H), 7.67 – 7.62 (m, 1H).^{13}C NMR (126 MHz, DMSO) δ 164.66, 162.56, 150.71, 147.60, 145.69, 141.85, 138.18, 136.71, 136.22, 133.34, 132.37, 130.23, 129.22, 128.68, 126.72, 124.80, 124.11, 120.33, 112.43, 111.56.

***N*-(2-(pyridin-2-yl)benzo[*d*]oxazol-5-yl)-4-(trifluoromethyl)benzamide(4ai)**

^{1}H NMR (500 MHz, DMSO) δ 10.70 (s, 1H), 8.83 (d, *J* = 4.2 Hz, 1H), 8.43 – 8.35 (m, 2H), 8.22 (d, *J* = 8.0 Hz, 2H), 8.08 (tt, *J* = 12.1, 6.0 Hz, 1H), 7.96 (d, *J* = 8.1 Hz, 2H), 7.90 – 7.85 (m, 2H), 7.66 (dd, *J* = 6.9, 5.1 Hz, 1H).^{13}C NMR (126 MHz, DMSO) δ 166.68, 164.97, 162.56, 150.69, 147.59, 145.69, 141.86, 139.15, 138.15, 136.69, 130.58, 129.12, 126.70, 125.88, 124.10, 120.16, 112.26, 111.57.

2-fluoro-*N*-(2-(pyridin-2-yl)benzo[*d*]oxazol-5-yl)benzamide(4aj)

^{1}H NMR (500 MHz, DMSO) δ 10.70 (s, 1H), 8.83 (s, 1H), 8.38 (s, 2H), 8.09 (s, 1H), 7.88 – 7.68 (m, 5H), 7.39 (d, *J* = 7.4 Hz, 2H).^{13}C NMR (126 MHz, DMSO) δ 163.37, 162.57, 150.71, 147.44, 145.68, 141.90, 138.19, 136.82, 133.12, 133.06, 130.44, 126.73, 125.08, 124.11, 119.44, 116.78, 116.61, 111.70, 111.42.

3-fluoro-*N*-(2-(pyridin-2-yl)benzo[*d*]oxazol-5-yl)benzamide(4ak)

^{1}H NMR (500 MHz, DMSO) δ 8.83 (s, 1H), 8.38 (d, *J* = 11.4 Hz, 2H), 8.09 (t, *J* = 7.1 Hz, 1H), 7.92 – 7.79 (m, 4H), 7.65 (d, *J* = 6.2 Hz, 2H), 7.52 – 7.35 (m, 2H).^{13}C NMR (126 MHz, DMSO) δ 164.74, 163.42, 162.52, 161.48, 150.70, 147.52, 145.69, 141.84, 138.17, 136.79, 131.06, 126.70, 124.10, 120.20, 118.93, 116.09, 114.94, 112.25, 111.53.

4-fluoro-*N*-(2-(pyridin-2-yl)benzo[*d*]oxazol-5-yl)benzamide(4al)

^{1}H NMR (500 MHz, DMSO) δ 10.56 (s, 1H), 8.83 (d, *J* = 4.2 Hz, 1H), 8.37 (d, *J* = 7.5 Hz, 2H), 8.10 (ddd, *J* = 9.1, 8.2, 3.5 Hz, 3H), 7.87 – 7.80 (m, 2H), 7.66 (dd, *J* = 7.1, 5.0 Hz, 1H), 7.41 (t, *J* = 8.8 Hz, 2H).^{13}C NMR (126 MHz, DMSO) δ 165.60, 165.05, 163.62, 162.49, 150.70, 147.43, 145.71, 141.84, 138.17, 136.98, 130.90, 126.69, 124.09, 120.18, 115.76, 112.18, 111.49.

4-chloro-*N*-(2-(pyridin-2-yl)benzo[*d*]oxazol-5-yl)benzamide(4am)

^{1}H NMR (500 MHz, DMSO) δ 10.63 (s, 1H), 8.83 (d, *J* = 4.2 Hz, 1H), 8.37 (d, *J* = 9.4 Hz, 2H), 8.11 – 8.06 (m, 3H), 7.88 – 7.84 (m, 2H), 7.66 (t, *J* = 7.9 Hz, 3H).^{13}C NMR (126 MHz, DMSO) δ 165.04, 162.50, 150.70, 147.48, 145.70, 141.83, 138.17, 136.96, 136.89, 134.03, 130.17, 128.97, 126.70, 124.09, 120.18, 112.21, 111.50.

4-bromo-*N*-(2-(pyridin-2-yl)benzo[*d*]oxazol-5-yl)benzamide(4an)

^{1}H NMR (500 MHz, DMSO) δ 10.69 (s, 1H), 8.83 (d, *J* = 4.1 Hz, 1H), 8.39 – 8.36 (m, 1H), 8.09 (dd, *J* = 10.9, 4.5 Hz, 1H), 8.01 (d, *J* = 8.4 Hz, 2H), 7.86 (s, 2H), 7.78 (d, *J* = 8.4 Hz, 2H), 7.66 (dd, *J* = 7.1, 5.1 Hz, 1H), 7.47 (d, *J* = 8.2 Hz, 1H).^{13}C NMR (126 MHz, DMSO) δ 165.17, 162.49, 150.70, 147.47, 145.70, 141.83, 138.17, 136.91, 134.40, 131.90, 130.37, 126.69, 125.88, 124.09, 120.20, 112.22, 111.49.

***N*-(2-(pyridin-2-yl)benzo[*d*]oxazol-5-yl)thiophene-2-carboxamide(4ao)**

^{1}H NMR (500 MHz, DMSO) δ 10.46 (s, 1H), 8.83 (s, 1H), 8.40 – 8.32 (m, 2H), 8.10 (s, 2H), 7.92 – 7.79 (m, 3H), 7.67 (s, 1H), 7.28 (s, 1H).^{13}C NMR (126 MHz, DMSO) δ 162.53, 160.52, 150.70, 147.47, 145.70, 141.89, 140.37, 138.18, 136.59, 132.44, 129.73, 128.57, 126.71, 124.11, 120.15, 112.21, 111.57.

***N*-(2-(pyridin-3-yl)benzo[*d*]oxazol-5-yl)benzamide(4ba)**

^{1}H NMR (500 MHz, $CDCl_3$) δ 10.48 (s, 1H), 9.34 (s, 1H), 8.79 (s, 1H), 8.52 (d, *J* = 6.9 Hz, 1H), 8.32 (s, 1H), 7.97 (d, *J* = 6.3 Hz, 2H), 7.79 (s, 2H), 7.65 – 7.52 (m, 4H).^{13}C NMR (126 MHz, $CDCl_3$) δ 165.72, 161.05, 152.39, 147.99, 146.70, 141.37, 136.61, 134.88, 131.69, 128.45, 127.71, 124.31, 122.85, 119.28, 111.43, 111.29, 110.82.

2-phenyl-*N*-(2-(pyridin-3-yl)benzo[*d*]oxazol-5-yl)acetamide(4bb)

^{1}H NMR (500 MHz, DMSO) δ 10.48 (s, 1H), 9.36 (s, 1H), 8.83 (s, 1H), 8.55 (d, *J* = 6.8 Hz, 1H), 8.23 (s, 1H), 7.79 (d, *J* = 8.6 Hz, 1H), 7.70 – 7.58 (m, 2H), 7.41 – 7.23 (m, 5H), 3.72 (s, 2H).^{13}C NMR (126 MHz, DMSO) δ 169.71, 161.50, 152.81, 148.46, 146.82, 141.90, 137.20, 136.40, 135.23, 129.67, 128.83, 127.07, 124.82, 123.30, 118.43, 111.44, 110.41, 43.79.

2-methyl-*N*-(2-(pyridin-3-yl)benzo[*d*]oxazol-5-yl)benzamide(4bc)

^{1}H NMR (500 MHz, DMSO) δ 10.56 (s, 1H), 9.41 (s, 1H), 8.87 (s, 1H), 8.63 (d, *J* = 6.9 Hz, 1H), 8.37 (s, 1H), 7.85 – 7.73 (m, 3H), 7.54 (d, *J* = 6.8 Hz, 1H), 7.44 (d, *J* = 7.0 Hz, 1H), 7.36 (d, *J* = 7.3 Hz, 2H), 2.45 (s, 3H).^{13}C NMR (126 MHz, DMSO) δ 168.41, 161.29, 152.17, 147.92, 147.05, 141.86, 137.60, 137.28, 135.92, 135.76, 131.05, 130.21, 127.75, 126.17, 125.12, 123.57, 119.01, 111.42, 110.98, 19.82.

3-methyl-*N*-(2-(pyridin-3-yl)benzo[*d*]oxazol-5-yl)benzamide(4bd)

^{1}H NMR (500 MHz, DMSO) δ 10.55 (s, 1H), 9.39 (s, 1H), 8.84 (s, 1H), 8.57 (d, *J* = 7.2 Hz, 1H), 8.38 (s, 1H), 7.85 (s, 3H), 7.70 (d, *J* = 14.2 Hz, 2H), 7.45 (s, 1H), 7.17 (d, *J* = 20.4 Hz, 1H), 2.44 (s, 3H).^{13}C NMR (126 MHz, DMSO) δ 166.28, 161.48, 152.81, 148.47, 147.11, 141.83, 138.22, 137.17, 135.22, 132.68, 130.21, 128.80, 128.67, 125.35, 124.82, 123.33, 119.72, 111.79, 111.25, 21.47.

4-methyl-*N*-(2-(pyridin-3-yl)benzo[*d*]oxazol-5-yl)benzamide(4be)

^{1}H NMR (500 MHz, DMSO) δ 10.45 (s, 1H), 9.39 (s, 1H), 8.84 (s, 1H), 8.57 (d, *J* = 7.2 Hz, 1H), 8.37 (s, 1H), 7.95 (d, *J* = 7.3 Hz, 2H), 7.84 (s, 2H), 7.69 (s, 1H), 7.39 (d, *J* = 7.4 Hz, 2H), 3.37 (s, 3H).^{13}C NMR (126 MHz, DMSO) δ 165.96, 161.47, 152.81, 148.47, 147.09, 142.15, 141.83, 137.17, 135.22, 132.44, 129.43, 128.23, 124.82, 123.33, 119.75, 111.80, 111.23, 21.52.

***N*-(2-(pyridin-3-yl)benzo[*d*]oxazol-5-yl)-2-naphthamide(4bf)**

^{1}H NMR (500 MHz, DMSO) δ 10.69 (s, 1H), 9.40 (s, 1H), 8.84 (s, 1H), 8.66 (s, 1H), 8.58 (d, *J* = 7.6 Hz, 1H), 8.43 (s, 1H), 8.10 (s, 4H), 7.88 (s, 2H), 7.68 (s, 3H).^{13}C NMR (126 MHz, DMSO) δ 166.20, 161.53, 152.82, 148.49, 147.18, 141.88, 137.15, 135.23, 134.80, 132.66, 132.58, 129.46, 128.55, 128.36, 128.18, 127.38, 124.94, 124.82, 123.33, 119.74, 119.64, 111.84, 111.33.

4-methoxy-*N*-(2-(pyridin-3-yl)benzo[*d*]oxazol-5-yl)benzamide(4bg)

^{1}H NMR (500 MHz, DMSO) δ 10.34 (s, 1H), 9.38 (s, 1H), 8.83 (s, 1H), 8.56 (d, *J* = 6.8 Hz, 1H), 8.35 (s, 1H), 8.02 (s, 2H), 7.82 (s, 2H), 7.68 (s, 1H), 7.10 (s, 2H), 3.88 (s, 3H).^{13}C NMR (126 MHz, DMSO) δ 165.50, 162.43, 161.43, 152.78, 148.46, 147.02, 141.82, 137.26, 135.19, 130.12, 127.33, 124.80, 123.33, 119.72, 114.12, 111.76, 111.18, 55.93.

***N*-(2-(pyridin-3-yl)benzo[*d*]oxazol-5-yl)-3-(trifluoromethyl)benzamide(4bh)**

^{1}H NMR (500 MHz, DMSO) δ 10.73 (s, 1H), 9.39 (s, 1H), 8.85 (s, 1H), 8.59 (s, 1H), 8.36 (s, 2H), 8.02 (d, *J* = 7.4 Hz, 1H), 7.90 – 7.81 (m, 4H), 7.70 (s, 1H).^{13}C NMR (126 MHz, DMSO) δ 164.65, 161.61, 152.86, 148.50, 147.36, 141.87, 138.72, 136.72, 136.24, 135.26, 132.37, 130.26, 129.82, 129.56, 128.68, 124.83, 123.29, 119.95, 112.15, 111.38.

***N*-(2-(pyridin-3-yl)benzo[*d*]oxazol-5-yl)-4-(trifluoromethyl)benzamide(4bi)**

^{1}H NMR (500 MHz, DMSO) δ 10.72 (s, 1H), 9.39 (s, 1H), 8.84 (s, 1H), 8.56 (s, 1H), 8.37 (s, 1H), 8.22 (d, *J* = 7.6 Hz, 2H), 7.97 (d, *J* = 7.6 Hz, 2H), 7.85 (d, *J* = 8.8 Hz, 2H), 7.69 (s, 1H).^{13}C NMR (126 MHz, DMSO) δ 164.99, 161.61, 152.85, 148.49, 147.35, 141.86, 139.15, 136.70, 135.24, 132.03, 131.78, 129.12, 125.90, 124.82, 123.28, 119.78, 111.98, 111.39.

2-fluoro-*N*-(2-(pyridin-3-yl)benzo[*d*]oxazol-5-yl)benzamide(4bj)

^{1}H NMR (500 MHz, DMSO) δ 10.68 (s, 1H), 9.39 (s, 1H), 8.84 (s, 1H), 8.57 (d, *J* = 6.9 Hz, 1H), 8.34 (s, 1H), 7.85 (d, *J* = 8.5 Hz, 1H), 7.77 – 7.63 (m, 4H), 7.40 (dd, *J* = 19.0, 8.9 Hz, 2H).^{13}C NMR (126 MHz, DMSO) δ 163.35, 161.62, 160.37, 152.86, 148.50, 147.20, 141.91, 136.81, 135.25, 133.07, 130.42, 125.12, 124.82, 123.28, 119.02, 116.79, 116.62, 111.51, 111.12.

3-fluoro-*N*-(2-(pyridin-3-yl)benzo[*d*]oxazol-5-yl)benzamide(4bk)

^{1}H NMR (500 MHz, DMSO) δ 10.60 (s, 1H), 9.38 (s, 1H), 8.83 (s, 1H), 8.56 (d, *J* = 7.1 Hz, 1H), 8.36 (s, 1H), 7.90 – 7.82 (m, 4H), 7.65 (d, *J* = 24.3 Hz, 2H), 7.49 (s, 1H).^{13}C NMR (126 MHz, DMSO) δ 164.73, 163.39, 161.54, 152.81, 148.47, 147.27, 141.83, 137.65, 136.78, 135.21, 131.12, 124.79, 124.42, 123.28, 119.78, 119.10, 115.11, 111.95, 111.31.

4-fluoro-*N*-(2-(pyridin-3-yl)benzo[*d*]oxazol-5-yl)benzamide(4bl)

^{1}H NMR (500 MHz, DMSO) δ 10.54 (s, 1H), 9.38 (s, 1H), 8.83 (s, 1H), 8.56 (d, *J* = 7.0 Hz, 1H), 8.35 (s, 1H), 8.12 (s, 2H), 7.83 (s, 2H), 7.68 (s, 1H), 7.42 (s, 2H).^{13}C NMR (126 MHz, DMSO) δ 165.02, 163.60, 161.50, 152.78, 148.45, 147.18, 141.82, 136.97, 135.23, 131.72, 130.91, 124.81, 123.31, 119.78, 115.93, 111.91, 111.26.

4-chloro-*N*-(2-(pyridin-3-yl)benzo[*d*]oxazol-5-yl)benzamide(4bm)

^{1}H NMR (500 MHz, DMSO) δ 10.62 (s, 1H), 9.39 (d, *J* = 1.4 Hz, 1H), 8.86 – 8.83 (m, 1H), 8.58 – 8.56 (m, 1H), 8.36 (s, 1H), 8.07 (d, *J* = 8.5 Hz, 2H), 7.91 (d, *J* = 8.4 Hz, 1H), 7.84 (s, 1H), 7.70 – 7.66 (m, 2H), 7.41 (d, *J* = 8.3 Hz, 1H).^{13}C NMR (126 MHz, DMSO) δ 165.07, 161.57, 152.84, 148.50, 147.28, 141.87, 135.24, 134.05, 131.39, 130.18, 128.99, 128.05, 124.82, 123.33, 119.84, 111.99, 111.31.

4-bromo-*N*-(2-(pyridin-3-yl)benzo[*d*]oxazol-5-yl)benzamide(4bn)

^{1}H NMR (500 MHz, DMSO) δ 10.57 (s, 1H), 9.38 (s, 1H), 8.84 (s, 1H), 8.56 (d, *J* = 7.0 Hz, 1H), 8.35 (s, 1H), 7.98 (d, *J* = 7.3 Hz, 2H), 7.82 (d, *J* = 13.6 Hz, 4H), 7.68 (s, 1H).^{13}C NMR (126 MHz, DMSO) δ 165.13, 161.54, 152.82, 148.48, 147.23, 141.84, 136.86, 135.22, 134.38, 131.92, 130.32, 125.90, 124.80, 123.29, 119.76, 111.91, 111.31.

***N*-(2-(pyridin-3-yl)benzo[*d*]oxazol-5-yl)thiophene-2-carboxamide(4bo)**

^{1}H NMR (500 MHz, DMSO) δ 10.49 (s, 1H), 9.39 (s, 1H), 8.84 (s, 1H), 8.56 (s, 1H), 8.30 (s, 1H), 8.10 (s, 1H), 7.92 (s, 1H), 7.85 (d, *J* = 8.6 Hz, 1H), 7.78 (d, *J* = 8.7 Hz, 1H), 7.69 (s, 1H), 7.28 (s, 1H). ^{13}C NMR (126 MHz, DMSO) δ 161.56, 160.50, 152.83, 148.49, 147.22, 141.88, 140.34, 136.59, 135.24, 132.46, 129.73, 128.58, 124.81, 123.30, 119.77, 111.94, 111.36.

***N*-(2-(pyridin-4-yl)benzo[*d*]oxazol-5-yl)benzamide(4ca)**

^{1}H NMR (500 MHz, DMSO) δ 10.50 (s, 1H), 8.88 (d, *J* = 5.8 Hz, 2H), 8.40 (s, 1H), 8.17 – 8.10 (m, 2H), 8.03 (d, *J* = 7.2 Hz, 2H), 7.87 (s, 2H), 7.61 (dt, *J* = 12.2, 5.9 Hz, 3H).^{13}C NMR (126 MHz, DMSO) δ 166.21, 161.42, 151.37, 147.29, 141.80, 137.26, 135.33, 133.97, 132.16, 128.93, 128.18, 121.28, 120.44, 112.05, 111.51.

2-phenyl-*N*-(2-(pyridin-4-yl)benzo[*d*]oxazol-5-yl)acetamide(4cb)

^{1}H NMR (500 MHz, DMSO) δ 10.52 (s, 1H), 8.86 (d, *J* = 6.1 Hz, 2H), 8.27 (s, 1H), 8.11 (d, *J* = 6.1 Hz, 2H), 7.82 (d, *J* = 8.9 Hz, 1H), 7.65 (d, *J* = 10.9 Hz, 1H), 7.42 – 7.34 (m, 4H), 7.29 (d, *J* = 7.0 Hz, 1H), 3.72 (s, 2H).^{13}C NMR (126 MHz, DMSO) δ 169.76, 161.39, 151.37, 146.93, 141.85, 137.39, 136.38, 133.94, 129.67, 128.82, 127.06, 121.26, 119.15, 111.68, 110.62, 43.78.

2-methyl-*N*-(2-(pyridin-4-yl)benzo[*d*]oxazol-5-yl)benzamide(4cc)

^{1}H NMR (500 MHz, DMSO) δ 10.57 (s, 1H), 8.87 (s, 2H), 8.40 (s, 1H), 8.13 (d, *J* = 5.9 Hz, 2H), 7.84 (dd, *J* = 20.2, 8.8 Hz, 2H), 7.54 (d, *J* = 7.4 Hz, 1H), 7.44 (t, *J* = 7.2 Hz, 1H), 7.36 (t, *J* = 7.7 Hz, 2H), 1.94 (s, 3H).^{13}C NMR (126 MHz, DMSO) δ 172.49, 168.44, 161.43, 151.40, 147.16, 141.84, 137.41, 135.78, 133.95, 131.07, 130.23, 127.75, 126.18, 121.27, 119.63, 111.64, 111.18, 21.55.

3-methyl-*N*-(2-(pyridin-4-yl)benzo[*d*]oxazol-5-yl)benzamide(4cd)

^{1}H NMR (500 MHz, DMSO) δ 10.47 (s, 1H), 8.88 (d, *J* = 6.0 Hz, 2H), 8.40 (s, 1H), 8.13 (d, *J* = 6.0 Hz, 2H), 7.87 – 7.79 (m, 4H), 7.50 – 7.41 (m, 2H), 2.45 (s, 3H).^{13}C NMR (126 MHz, DMSO) δ 166.31, 161.41, 151.39, 147.24, 141.79, 138.26, 137.30, 135.34, 133.96, 132.74, 128.84, 128.66, 125.34, 121.27, 120.38, 111.98, 111.52, 21.47.

4-methyl-*N*-(2-(pyridin-4-yl)benzo[*d*]oxazol-5-yl)benzamide(4ce)

^{1}H NMR (500 MHz, DMSO) δ 10.44 (s, 1H), 8.87 (d, *J* = 6.0 Hz, 2H), 8.40 (s, 1H), 8.13 (d, *J* = 6.0 Hz, 2H), 7.95 (d, *J* = 8.1 Hz, 2H), 7.86 (s, 2H), 7.39 (d, *J* = 7.9 Hz, 2H), 2.43 (s, 3H).^{13}C NMR (126 MHz, DMSO) δ 166.00, 161.38, 151.38, 147.21, 142.19, 141.78, 137.34, 133.97, 132.41, 129.45, 128.23, 121.27, 120.42, 111.99, 111.48, 21.52.

***N*-(2-(pyridin-4-yl)benzo[*d*]oxazol-5-yl)-2-naphthamide(4cf)**

^{1}H NMR (500 MHz, DMSO) δ 10.69 (s, 1H), 8.88 (d, *J* = 5.9 Hz, 2H), 8.65 (s, 1H), 8.46 (d, *J* = 1.3 Hz, 1H), 8.16 – 8.03 (m, 6H), 7.91 (dt, *J* = 18.0, 5.3 Hz, 2H), 7.71 – 7.64 (m, 2H).^{13}C NMR (126 MHz, DMSO) δ 166.24, 161.43, 151.35, 147.32, 141.84, 137.33, 134.83, 134.00, 132.63, 132.60, 129.46, 128.80, 128.56, 128.37, 128.19, 127.38, 124.93, 121.29, 120.46, 112.08, 111.55.

4-methoxy-*N*-(2-(pyridin-4-yl)benzo[*d*]oxazol-5-yl)benzamide(4cg)

^{1}H NMR (500 MHz, DMSO) δ 10.34 (s, 1H), 8.88 (d, *J* = 5.7 Hz, 2H), 8.39 (s, 1H), 8.17 – 8.11 (m, 2H), 8.03 (d, *J* = 8.8 Hz, 2H), 7.86 (s, 2H), 7.12 (d, *J* = 8.8 Hz, 2H), 3.88 (s, 3H).^{13}C NMR (126 MHz, DMSO) δ 165.55, 162.49, 161.37, 151.38, 147.17, 141.79, 137.45, 133.99, 130.13, 127.31, 121.27, 120.44, 114.15, 111.99, 111.44, 55.95.

***N*-(2-(pyridin-4-yl)benzo[*d*]oxazol-5-yl)-3-(trifluoromethyl)benzamide(4ch)**

^{1}H NMR (500 MHz, DMSO) δ 10.75 (s, 1H), 8.88 (d, *J* = 5.9 Hz, 2H), 8.36 (dd, *J* = 21.3, 13.1 Hz, 3H), 8.14 (d, *J* = 5.9 Hz, 2H), 8.03 (d, *J* = 7.7 Hz, 1H), 7.86 (dd, *J* = 20.6, 12.0 Hz, 3H).^{13}C NMR (126 MHz, DMSO) δ 164.70, 161.53, 154.77, 151.40, 147.49, 141.81, 136.84, 136.15, 133.93, 132.38, 130.29, 129.84, 128.80, 124.81, 121.30, 120.63, 112.37, 111.65.

***N*-(2-(pyridin-4-yl)benzo[*d*]oxazol-5-yl)-4-(trifluoromethyl)benzamide(4ci)**

^{1}H NMR (500 MHz, DMSO) δ 10.71 (s, 1H), 8.86 (d, *J* = 6.0 Hz, 2H), 8.39 (s, 1H), 8.21 (d, *J* = 8.1 Hz, 2H), 8.11 (d, *J* = 6.0 Hz, 2H), 7.95 (d, *J* = 8.3 Hz, 2H), 7.86 (s, 2H).^{13}C NMR (126 MHz, DMSO) δ 164.99, 161.48, 151.35, 147.44, 141.78, 139.09, 136.86, 133.89, 132.06, 129.12, 125.89, 123.32, 121.25, 120.43, 112.17, 111.60.

2-fluoro-*N*-(2-(pyridin-4-yl)benzo[*d*]oxazol-5-yl)benzamide(4cj)

^{1}H NMR (500 MHz, DMSO) δ 10.69 (s, 1H), 8.88 (d, *J* = 5.5 Hz, 2H), 8.38 (s, 1H), 8.14 (d, *J* = 5.6 Hz, 2H), 7.88 (d, *J* = 8.8 Hz, 1H), 7.82 – 7.73 (m, 2H), 7.64 (d, *J* = 6.6 Hz, 1H), 7.40 (dd, *J* = 19.6, 8.8 Hz, 2H).^{13}C NMR (126 MHz, DMSO) δ 163.39, 161.54, 151.41, 147.33, 141.86, 136.98, 133.92, 133.18, 130.45, 125.42, 125.11, 121.29, 119.72, 116.81, 116.63, 111.77, 111.35.

3-fluoro-*N*-(2-(pyridin-4-yl)benzo[*d*]oxazol-5-yl)benzamide(4ck)

^{1}H NMR (500 MHz, DMSO) δ 10.67 (s, 1H), 8.88 (d, *J* = 4.1 Hz, 2H), 8.38 (s, 1H), 8.14 (d, *J* = 4.2 Hz, 2H), 7.88 (d, *J* = 8.7 Hz, 1H), 7.81 – 7.73 (m, 2H), 7.64 (d, *J* = 5.8 Hz, 1H), 7.40 (dd, *J* = 17.4, 9.2 Hz, 2H).^{13}C NMR (126 MHz, DMSO) δ 163.39, 161.54, 151.40, 147.35, 141.87, 136.98, 133.93, 133.17, 133.10, 130.43, 125.12, 121.29, 119.75, 116.80, 116.62, 111.75, 111.39.

4-fluoro-*N*-(2-(pyridin-4-yl)benzo[*d*]oxazol-5-yl)benzamide(4cl)

^{1}H NMR (500 MHz, DMSO) δ 10.54 (s, 1H), 8.88 (d, *J* = 5.9 Hz, 2H), 8.39 (s, 1H), 8.15 – 8.09 (m, 4H), 7.87 (d, *J* = 7.4 Hz, 2H), 7.43 (t, *J* = 8.8 Hz, 2H).^{13}C NMR (126 MHz, DMSO) δ 165.08, 161.44, 151.39, 147.31, 141.79, 137.13, 133.94, 131.73, 130.90, 121.27, 120.47, 115.97, 115.79, 112.11, 111.55.

4-chloro-*N*-(2-(pyridin-4-yl)benzo[*d*]oxazol-5-yl)benzamide(4cm)

^{1}H NMR (500 MHz, DMSO) δ 10.60 (s, 1H), 8.87 (d, *J* = 4.5 Hz, 2H), 8.38 (s, 1H), 8.12 (d, *J* = 5.8 Hz, 2H), 8.05 (d, *J* = 8.5 Hz, 2H), 7.86 (s, 2H), 7.66 (d, *J* = 8.5 Hz, 2H).^{13}C NMR (126 MHz, DMSO) δ 165.07, 161.45, 151.37, 147.37, 141.80, 137.08, 136.99, 134.02, 133.94, 130.16, 128.99, 121.27, 120.50, 112.18, 111.53.

4-bromo-*N*-(2-(pyridin-4-yl)benzo[*d*]oxazol-5-yl)benzamide(4cn)

^{1}H NMR (500 MHz, DMSO) δ 10.55 (s, 1H), 8.86 (s, 2H), 8.37 (s, 1H), 8.11 (d, *J* = 5.7 Hz, 2H), 7.97 (d, *J* = 8.4 Hz, 2H), 7.86 – 7.77 (m, 4H).^{13}C NMR (126 MHz, DMSO) δ 165.17, 161.43, 151.36, 147.34, 141.78, 137.01, 134.33, 133.91, 131.93, 130.32, 125.95, 121.26, 120.42, 112.11, 111.54.

***N*-(2-(pyridin-4-yl)benzo[*d*]oxazol-5-yl)thiophene-2-carboxamide(4co)**

^{1}H NMR (500 MHz, DMSO) δ 10.48 (s, 1H), 8.88 (d, *J* = 6.0 Hz, 2H), 8.34 (s, 1H), 8.14 (d, *J* = 4.5 Hz, 2H), 8.09 (d, *J* = 3.6 Hz, 1H), 7.90 (dd, *J* = 18.6, 7.3 Hz, 2H), 7.81 (d, *J* = 8.9 Hz, 1H), 7.31 – 7.27 (m, 1H).^{13}C NMR (126 MHz, DMSO) δ 161.49,

160.54, 151.40, 147.36, 141.84, 140.29, 136.76, 133.95, 132.53, 129.77, 128.60, 121.30, 120.47, 112.17, 111.63.

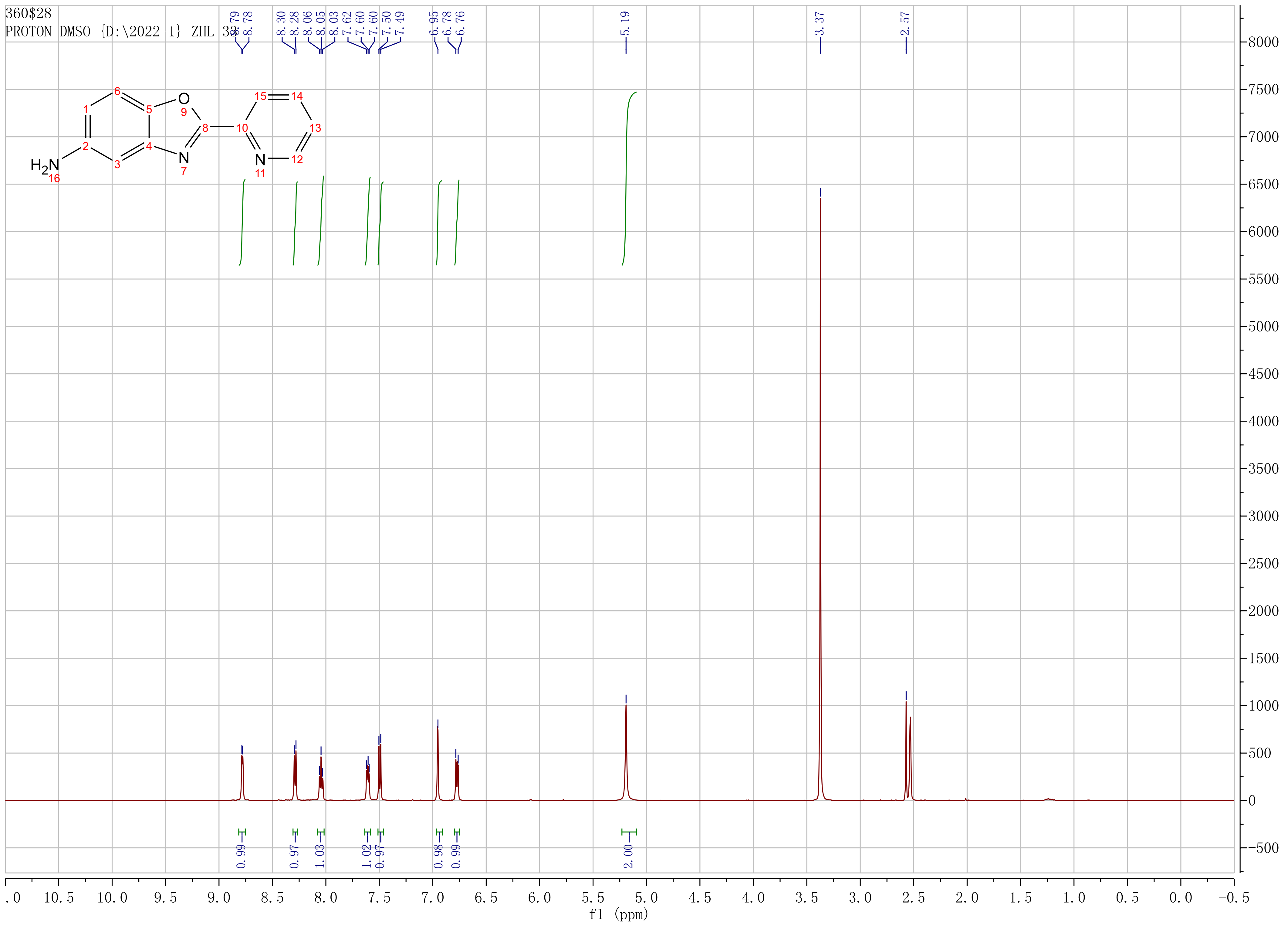

360$28
PROTON DMSO {D:\2022-1} ZHL 33
8.79
8.78
8.30
8.28
8.06
8.05
8.03
7.62
7.60
7.60
7.50
7.49
6.95
6.78
6.76
5.19
3.37
2.57
0.99
0.97
1.03
1.02
0.97
0.98
0.99
2.00
H2N
O
N
N
f1 (ppm)

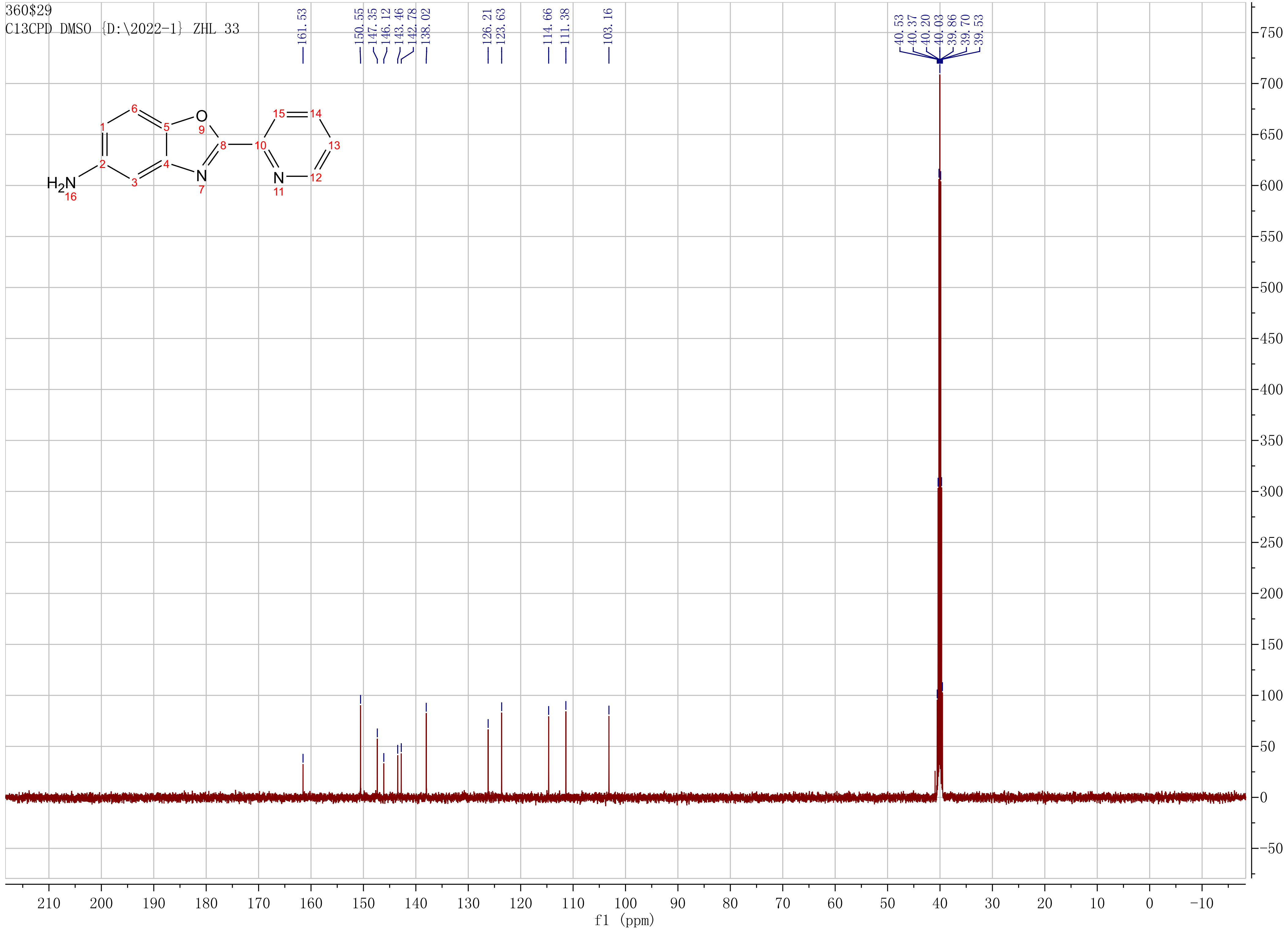

360$29
C13CPD DMSO {D:\2022-1} ZHL 33
161.53
150.55
147.35
146.12
143.46
142.78
138.02
126.21
123.63
114.66
111.38
103.16
40.53
40.37
40.20
40.03
39.86
39.70
39.53
H2N
O
N
N
f1 (ppm)

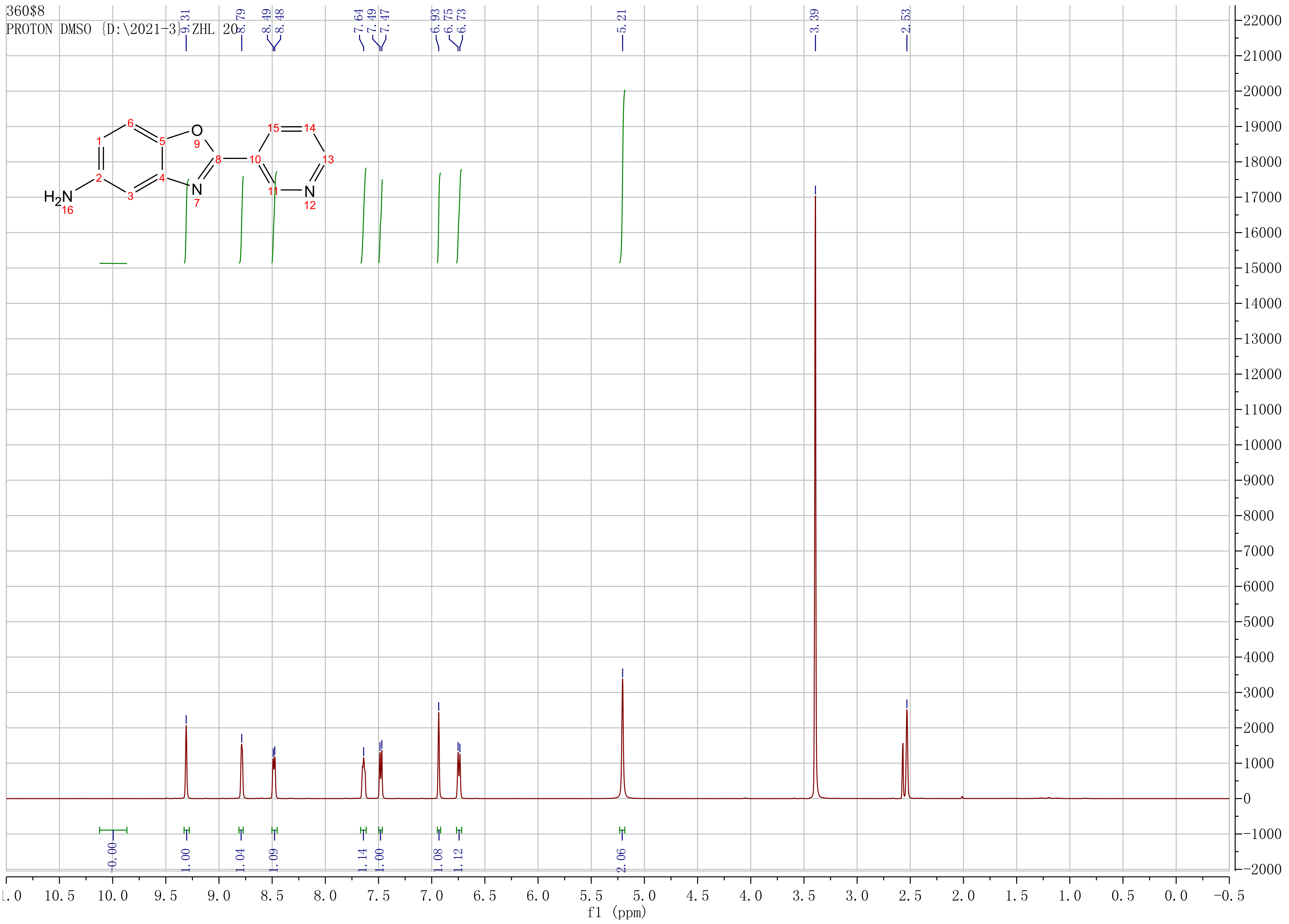

360$8
PROTON DMSO {D:\2021-3} ZHL 20
9.31
8.79
8.49
8.48
7.64
7.49
7.47
6.93
6.75
6.73
5.21
3.39
2.53
H2N
O
N
N
-0.00
1.00
1.04
1.09
1.14
1.00
1.08
1.12
2.06
f1 (ppm)

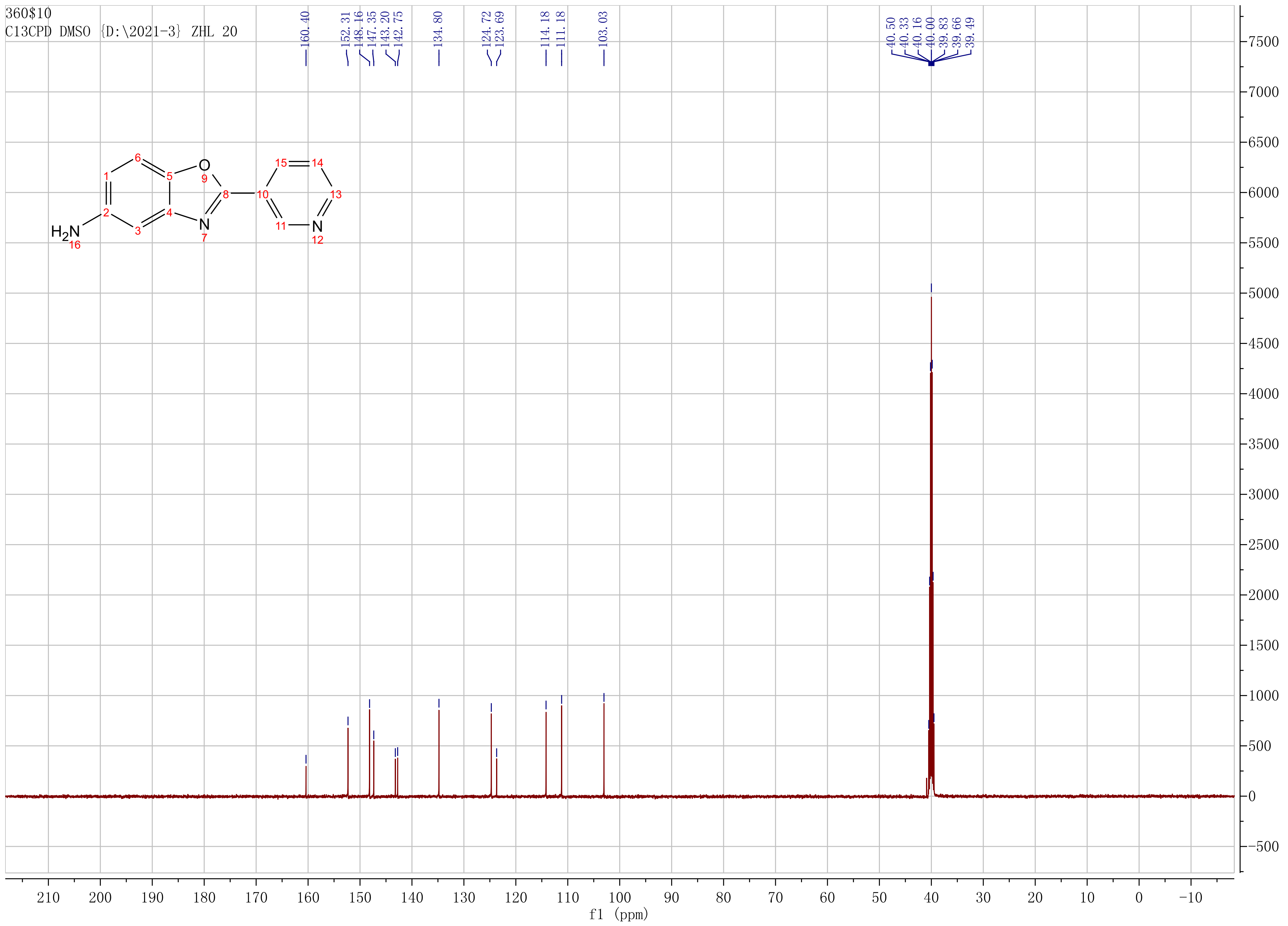

360$10
C13CPD DMSO {D:\2021-3} ZHL 20
160.40
152.31
148.16
147.35
143.20
142.75
134.80
124.72
123.69
114.18
111.18
103.03
40.50
40.33
40.16
40.00
39.83
39.66
39.49
f1 (ppm)

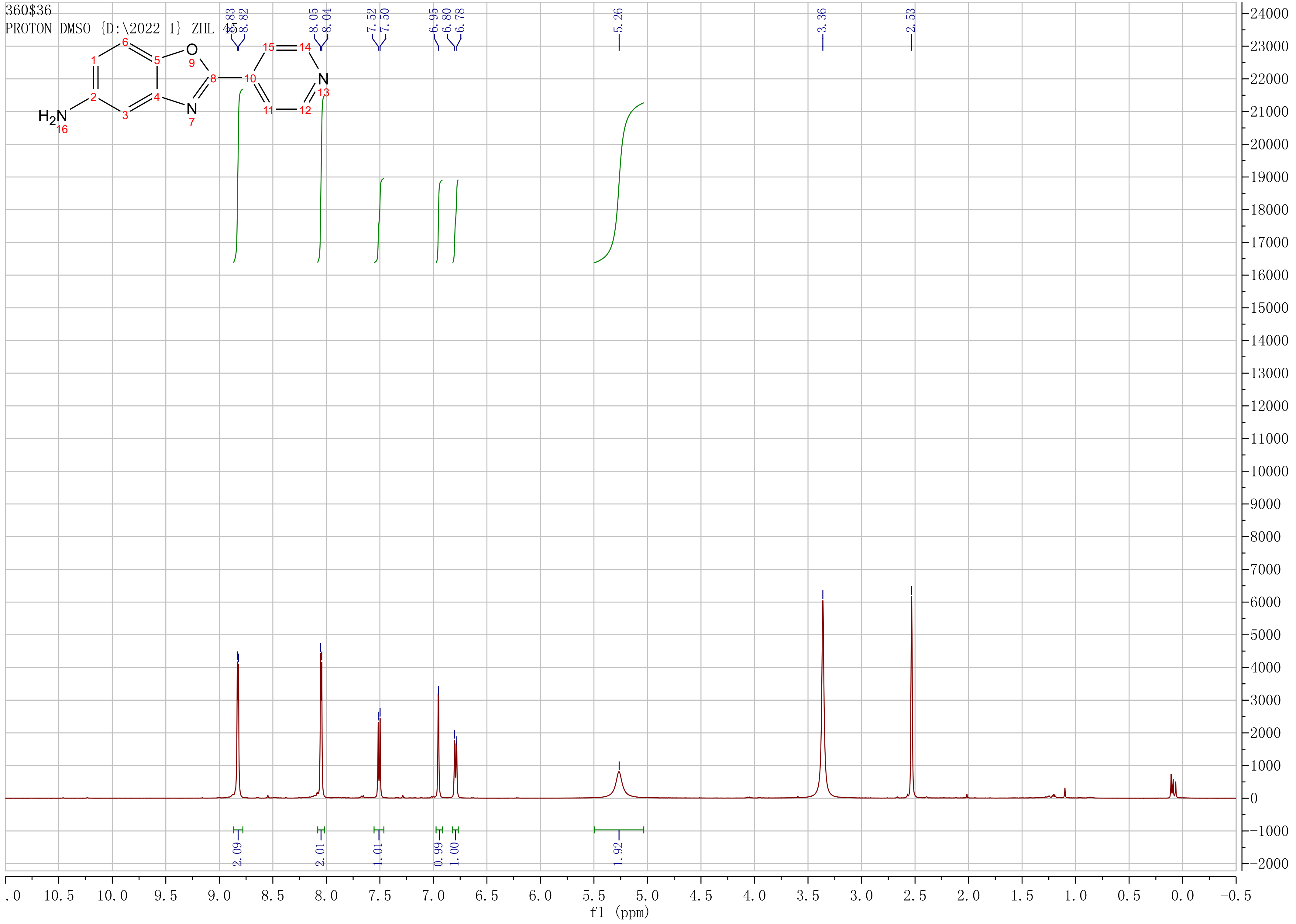
360$36
PROTON DMSO {D:\2022-1} ZHL 45
H2N
O
N
N
8.83
8.82
8.05
8.04
7.52
7.50
6.95
6.80
6.78
5.26
3.36
2.53
2.09
2.01
1.01
0.99
1.00
1.92
f1 (ppm)

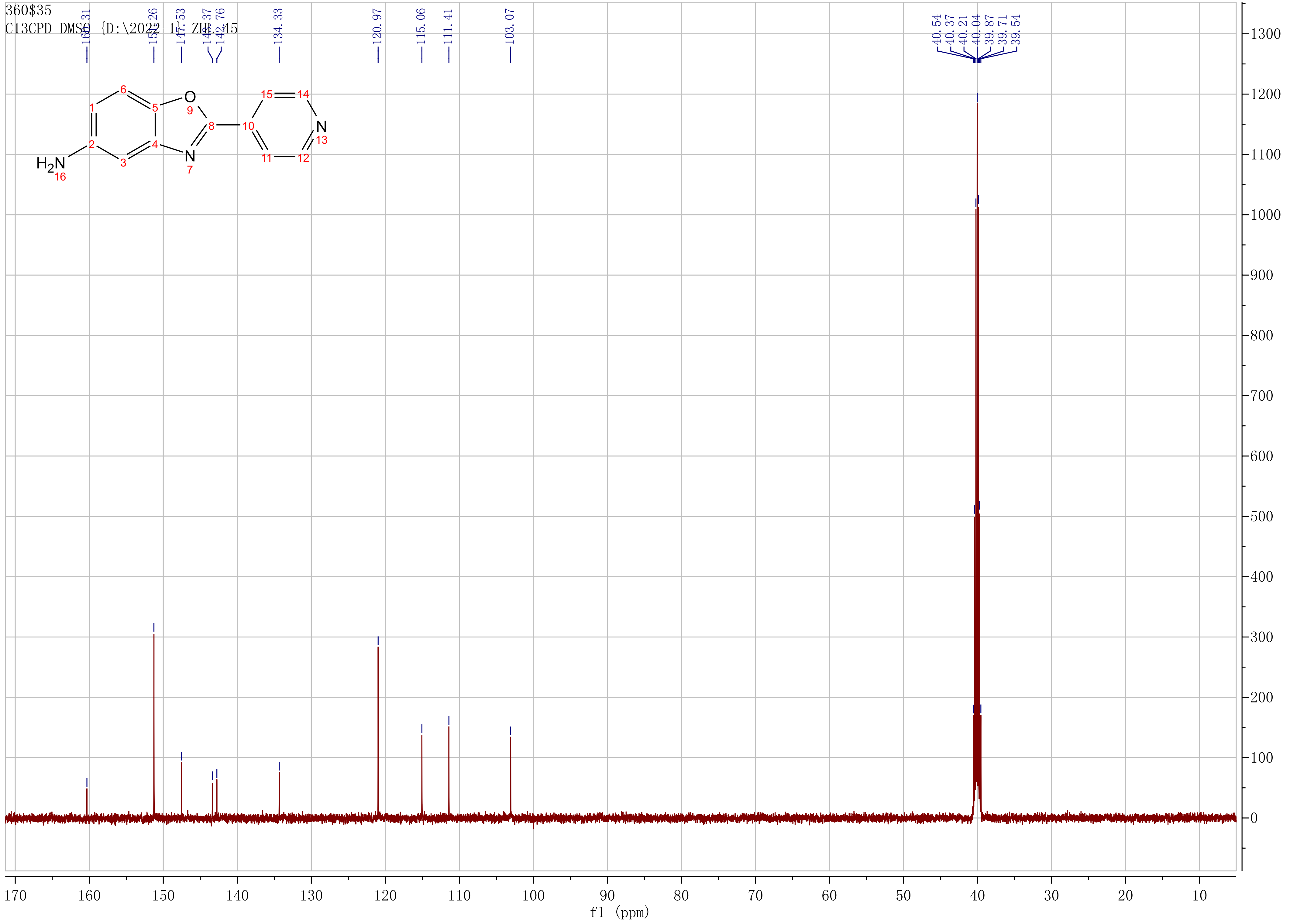

360$35
C13CPD DMSO {D:\2022-1 ZHL 45
147.53
142.76
134.33
120.97
115.06
111.41
103.07
40.54
40.37
40.21
40.04
39.87
39.71
39.54
O
N
H2N
1
2
3
4
5
6
7
8
9
10
11
12
13
14
15
16
f1 (ppm)

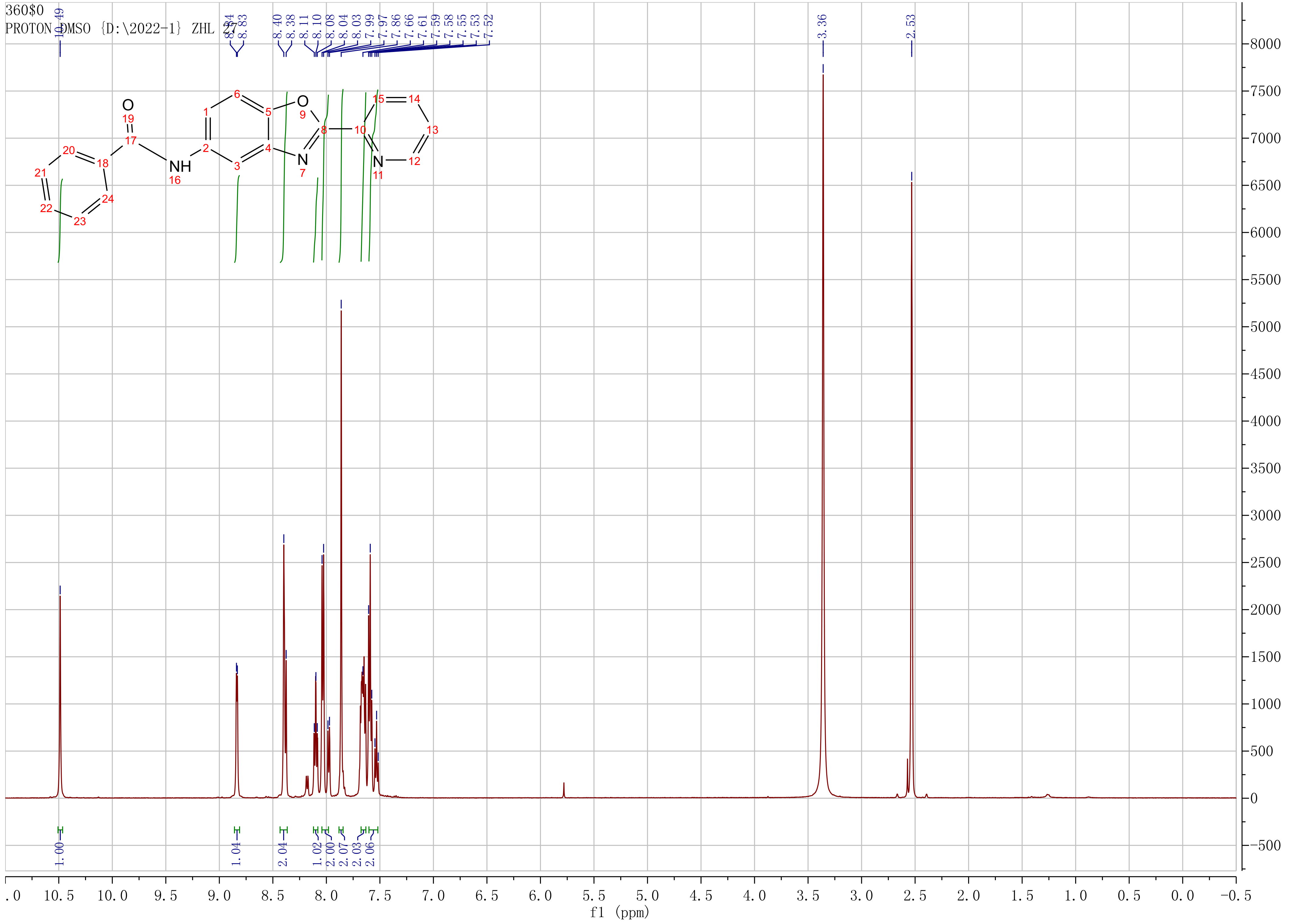
360$0
PROTON DMSO {D:\2022-1} ZHL
10.49
8.84
8.83
8.40
8.38
8.11
8.10
8.08
8.04
8.03
7.99
7.97
7.86
7.66
7.61
7.59
7.58
7.55
7.53
7.52
3.36
2.53
1.00
1.04
2.04
1.02
2.00
2.07
2.03
2.06
f1 (ppm)

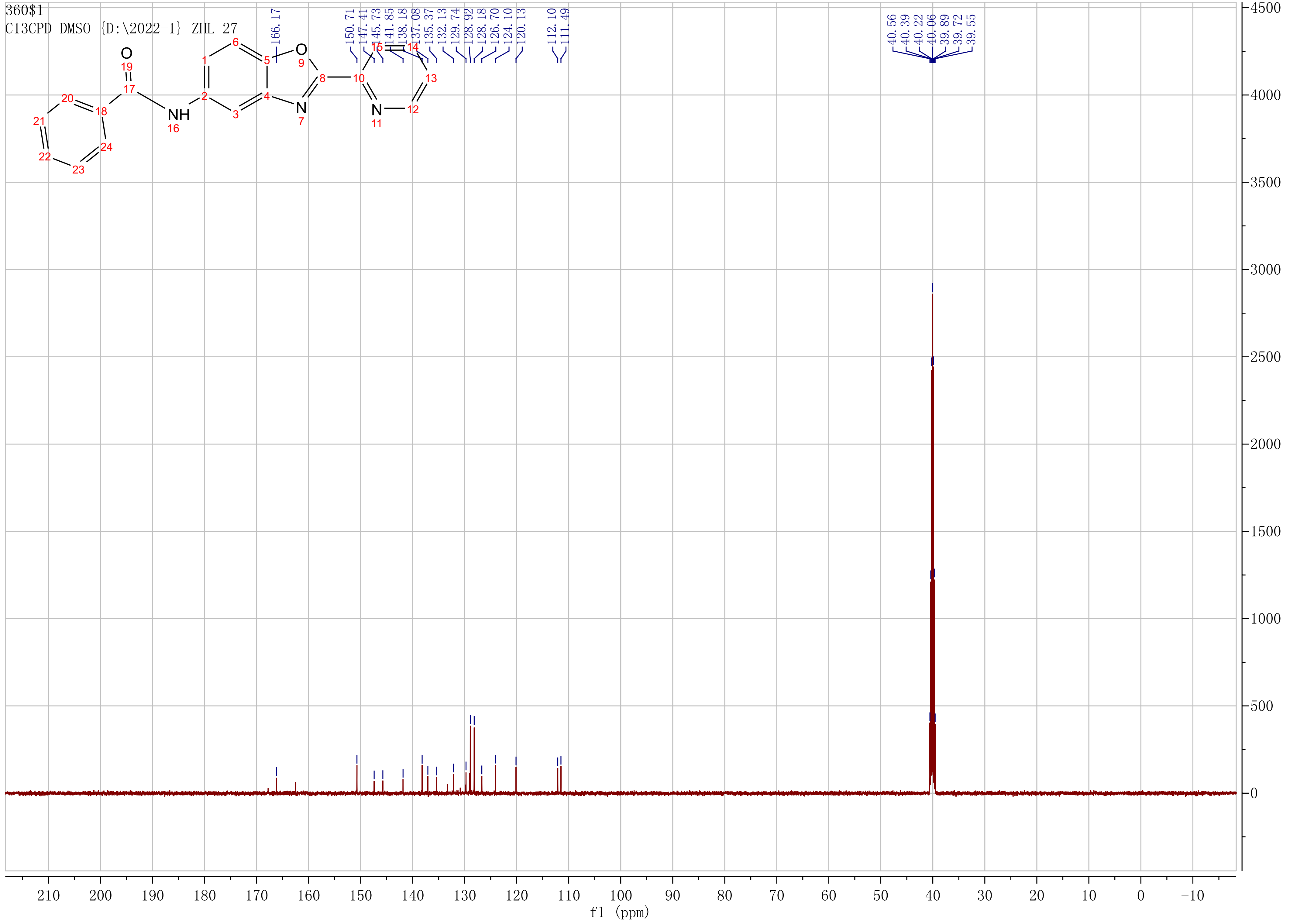

360$1
C13CPD DMSO {D:\2022-1} ZHL 27
166.17
150.71
147.41
145.73
141.85
138.18
137.08
135.37
132.13
129.74
128.92
128.18
126.70
124.10
120.13
112.10
111.49
40.56
40.39
40.22
40.06
39.89
39.72
39.55
f1 (ppm)

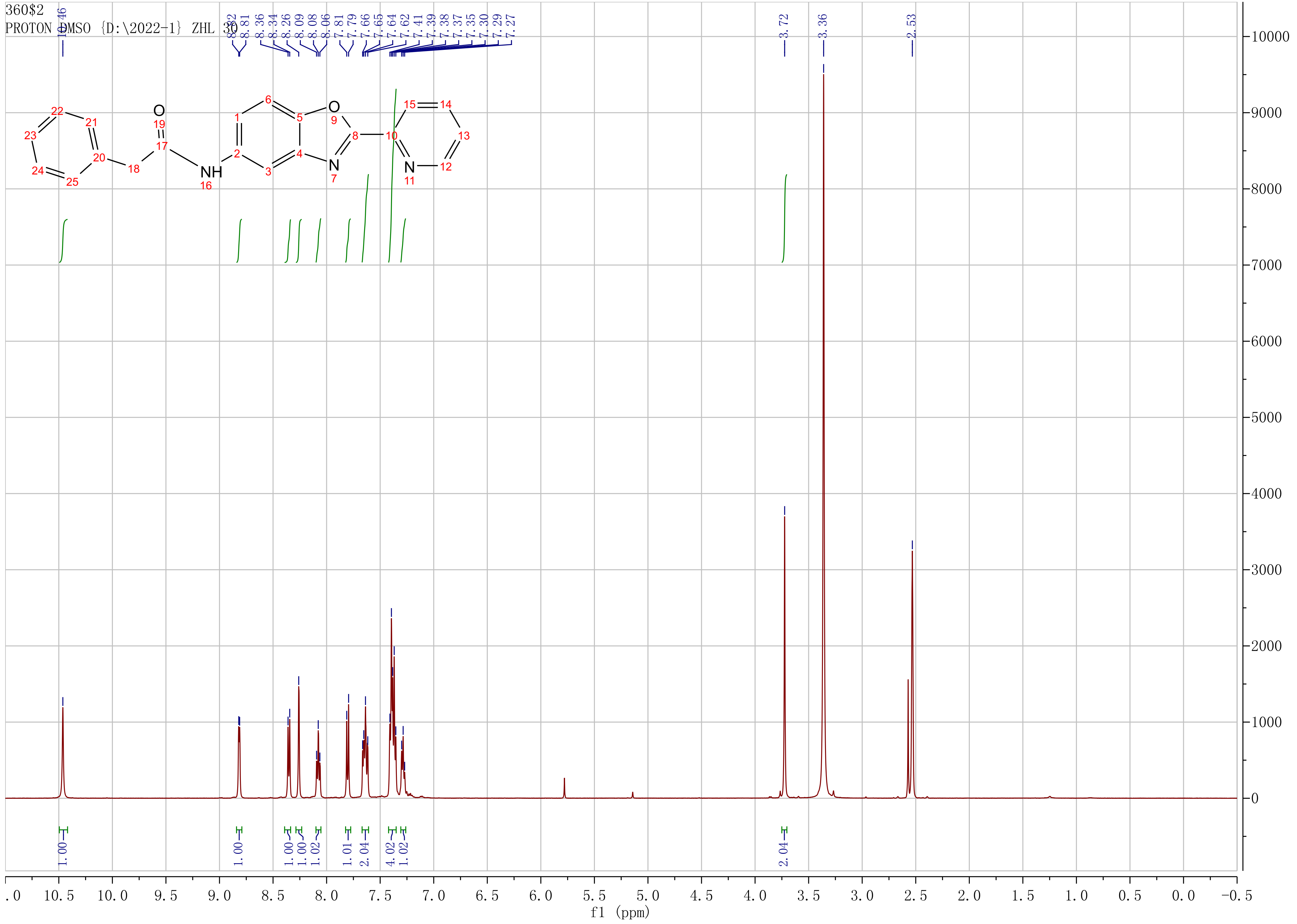
360$2
PROTON DMSO {D:\2022-1} ZHL 30
10.46
8.82
8.81
8.36
8.34
8.26
8.09
8.08
8.06
7.81
7.79
7.66
7.65
7.64
7.62
7.41
7.39
7.38
7.37
7.35
7.30
7.29
7.27
3.72
3.36
2.53
O
19
17
NH
16
18
20
21
22
23
24
25
1
2
3
4
5
6
O
9
8
N
7
10
N
11
12
13
14
15
1.00
1.00
1.00
1.00
1.02
1.01
2.04
4.02
1.02
2.04
f1 (ppm)

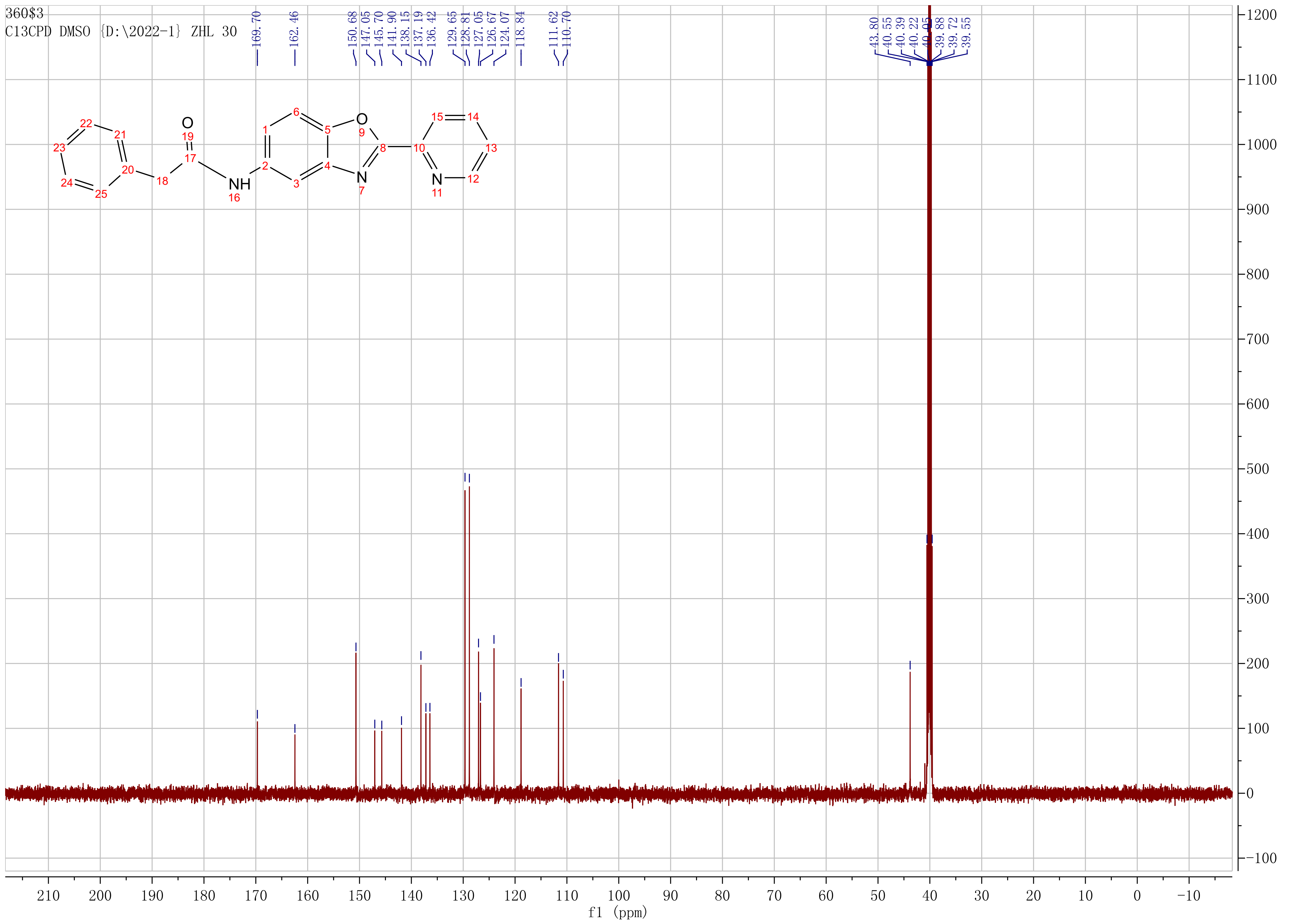

360$3
C13CPD DMSO {D:\2022-1} ZHL 30
169.70
162.46
150.68
147.05
145.70
141.90
138.15
137.19
136.42
129.65
128.81
127.05
126.67
124.07
118.84
111.62
110.70
43.80
40.55
40.39
40.22
40.05
39.88
39.72
39.55
f1 (ppm)

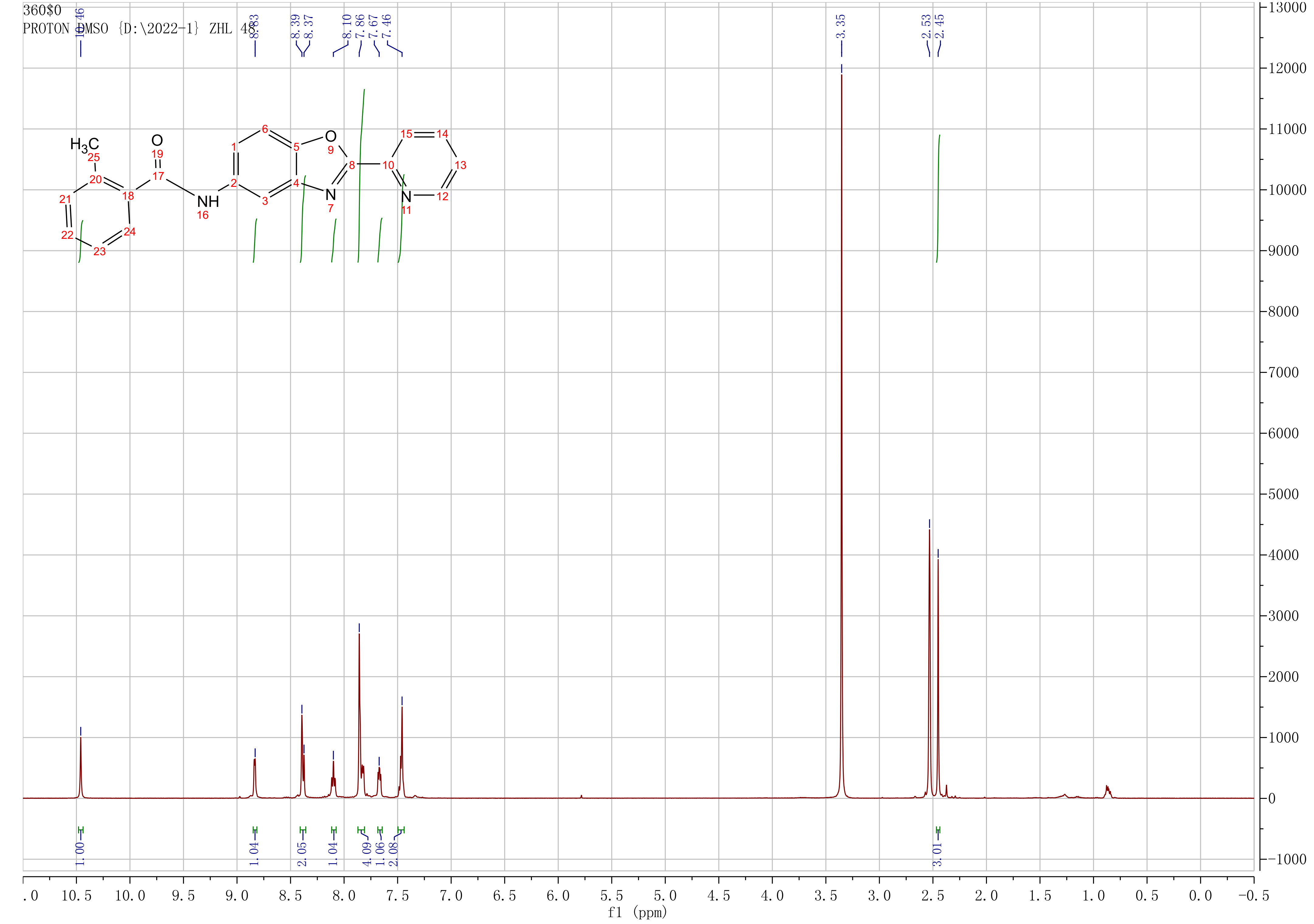
360$0
PROTON DMSO {D:\2022-1} ZHL 48
10.46
8.83
8.39
8.37
8.10
7.86
7.67
7.46
3.35
2.53
2.45
H3C
O
NH
N
O
N
1.00
1.04
2.05
1.04
4.09
1.06
2.08
3.01
f1 (ppm)

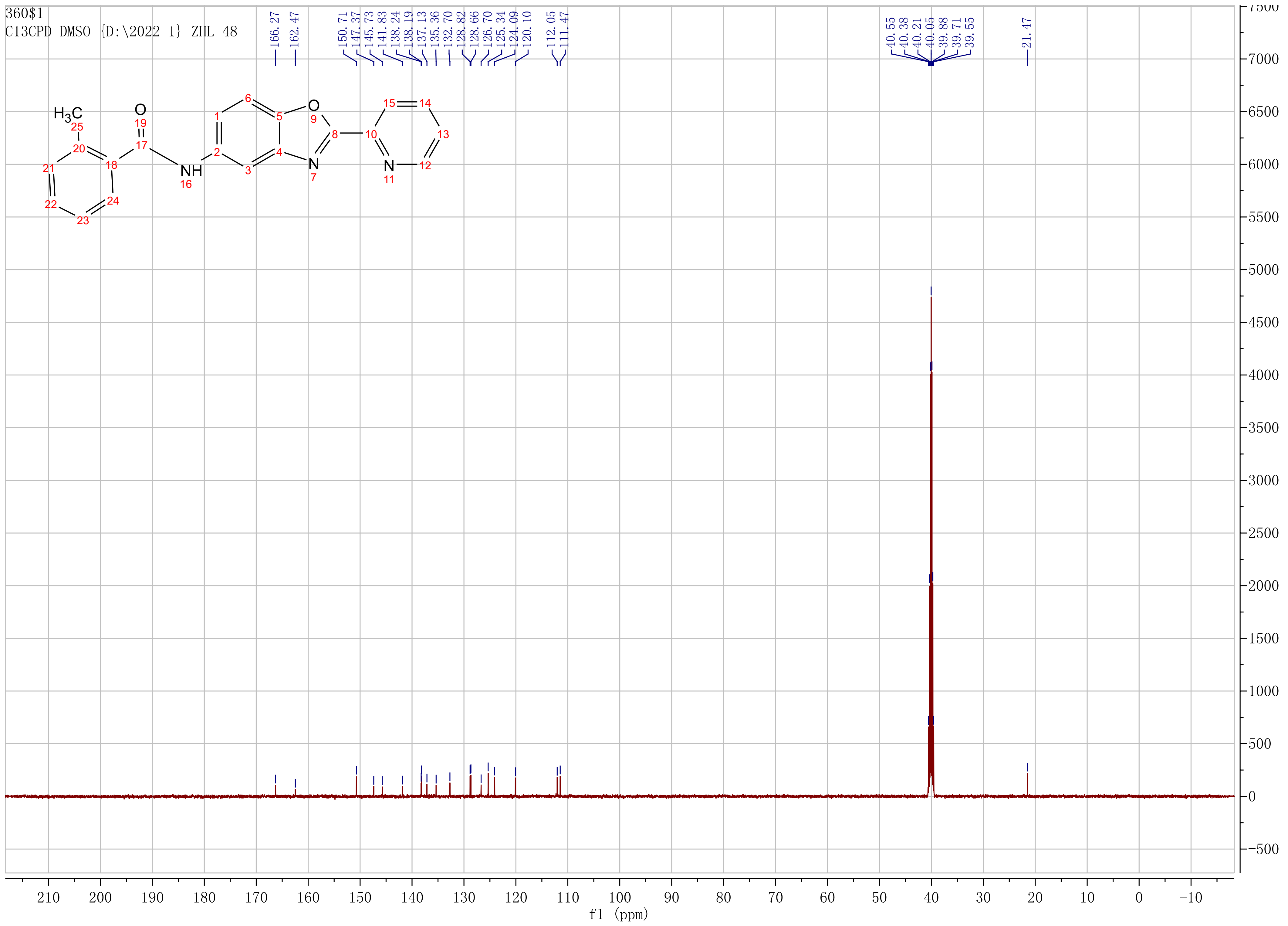

360$1
C13CPD DMSO {D:\2022-1} ZHL 48
166.27
162.47
150.71
147.37
145.73
141.83
138.24
138.19
137.13
135.36
132.70
128.82
128.66
126.70
125.34
124.09
120.10
112.05
111.47
40.55
40.38
40.21
40.05
39.88
39.71
39.55
21.47
f1 (ppm)

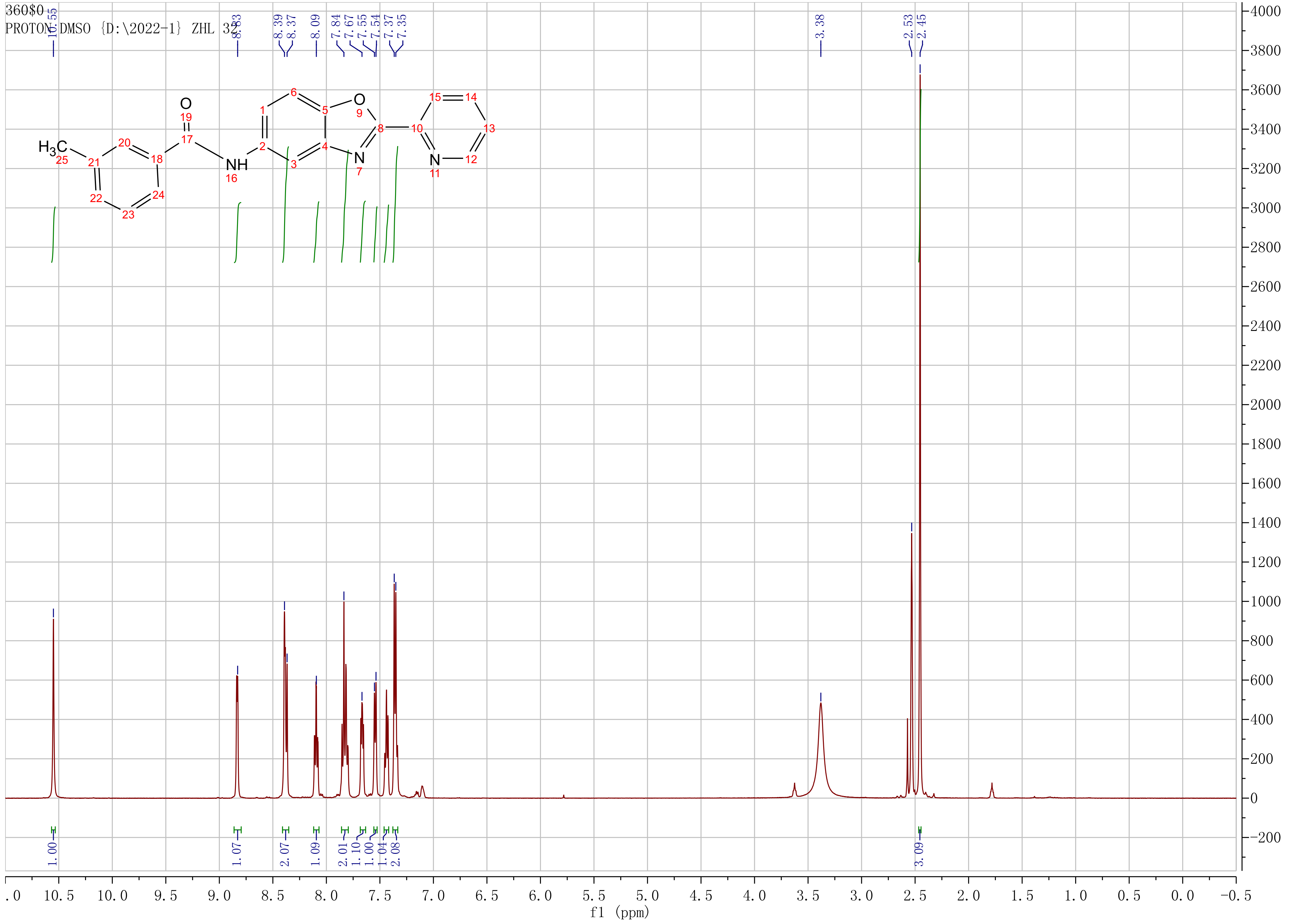
360$0
PROTON DMSO {D:\2022-1} ZHL 33
10.55
8.83
8.39
8.37
8.09
7.84
7.67
7.55
7.54
7.37
7.35
3.38
2.53
2.45
1.00
1.07
2.07
1.09
2.01
1.10
1.00
1.04
2.08
3.09
f1 (ppm)

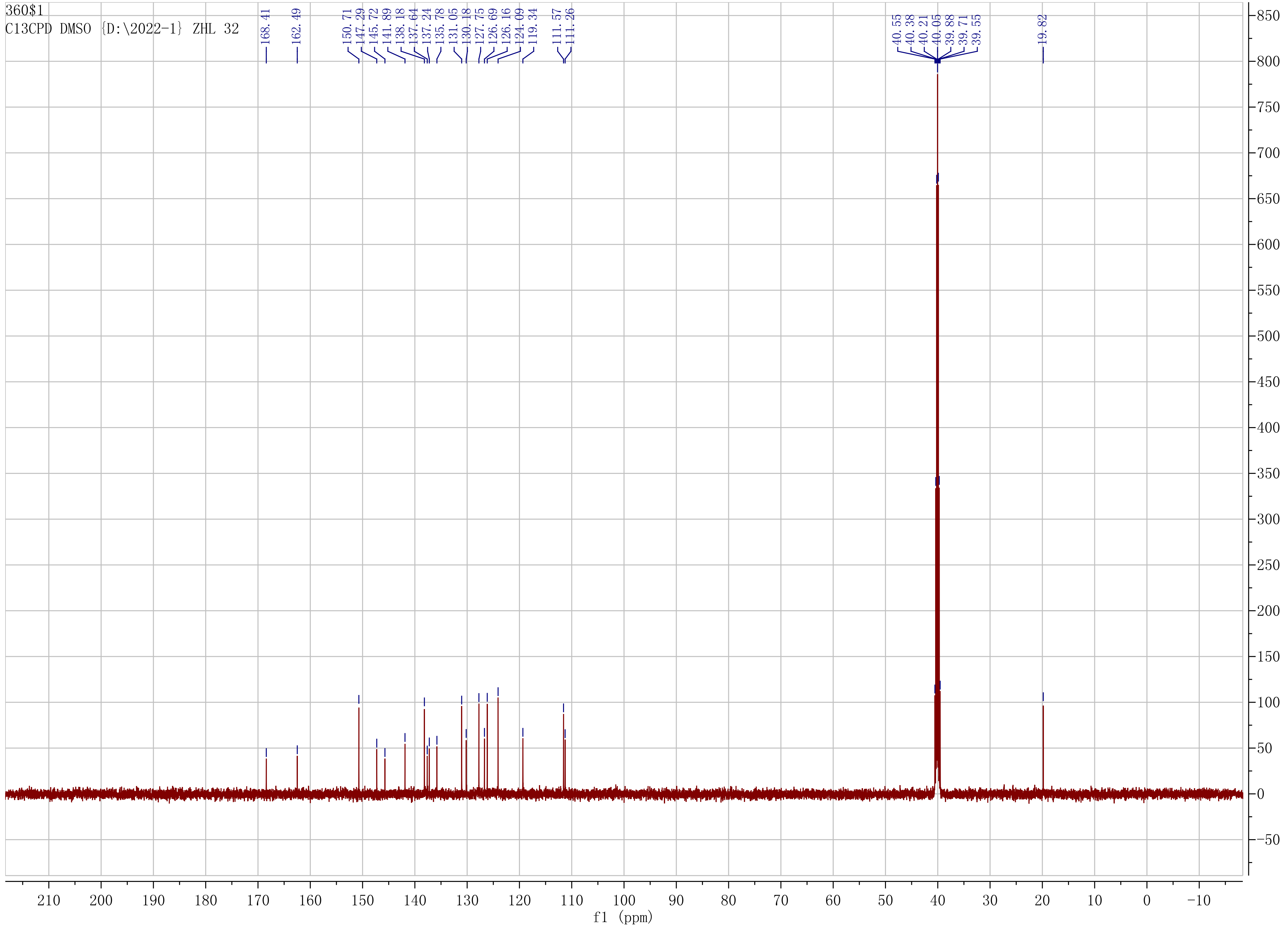
360$1
C13CPD DMSO {D:\2022-1} ZHL 32
168.41
162.49
150.71
147.29
145.72
141.89
138.18
137.64
137.24
135.78
131.05
130.18
127.75
126.69
126.16
124.09
119.34
111.57
111.26
40.55
40.38
40.21
40.05
39.88
39.71
39.55
19.82
f1 (ppm)

360$2
PROTON DMSO {D:\2022-1} ZHL 46

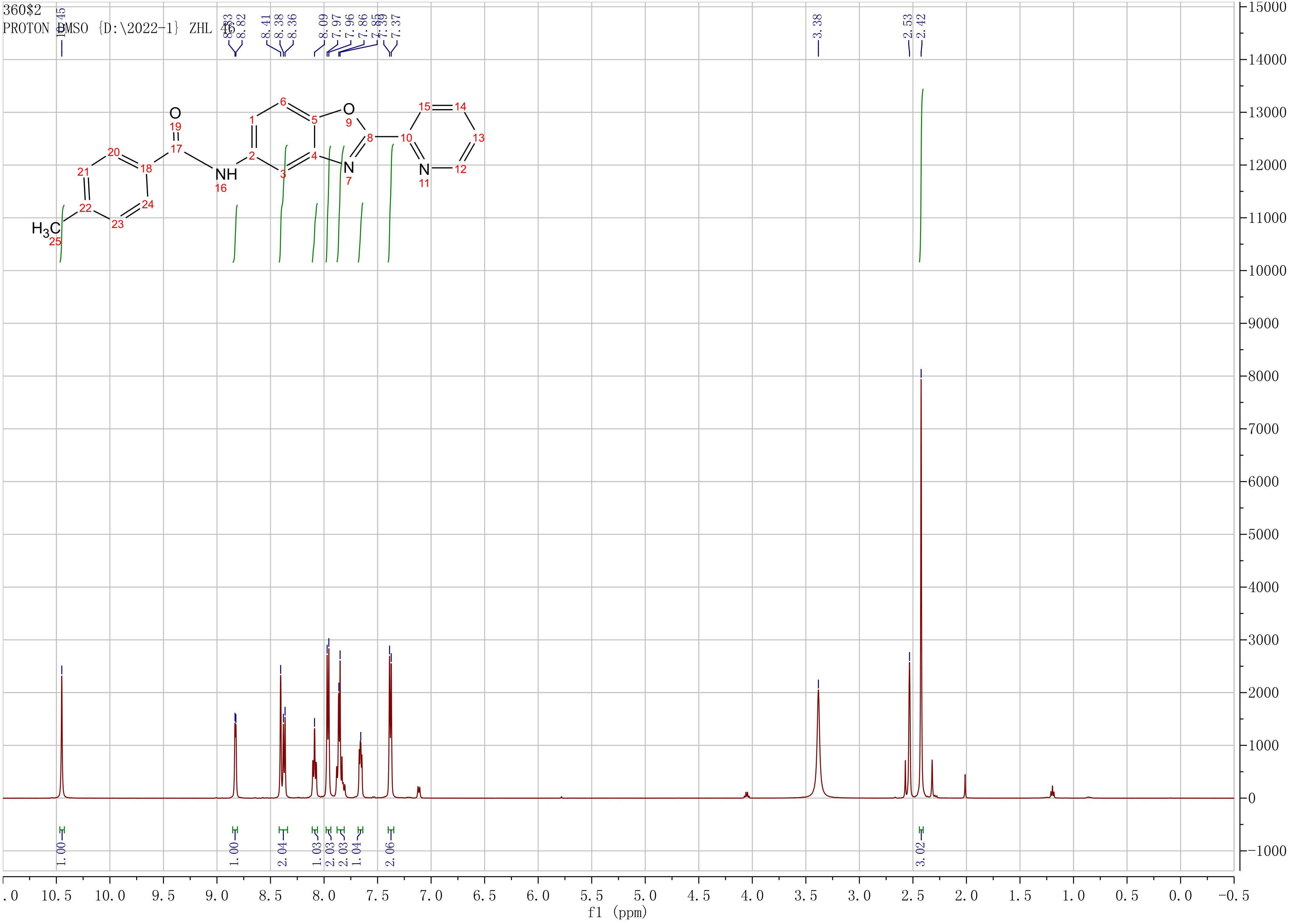

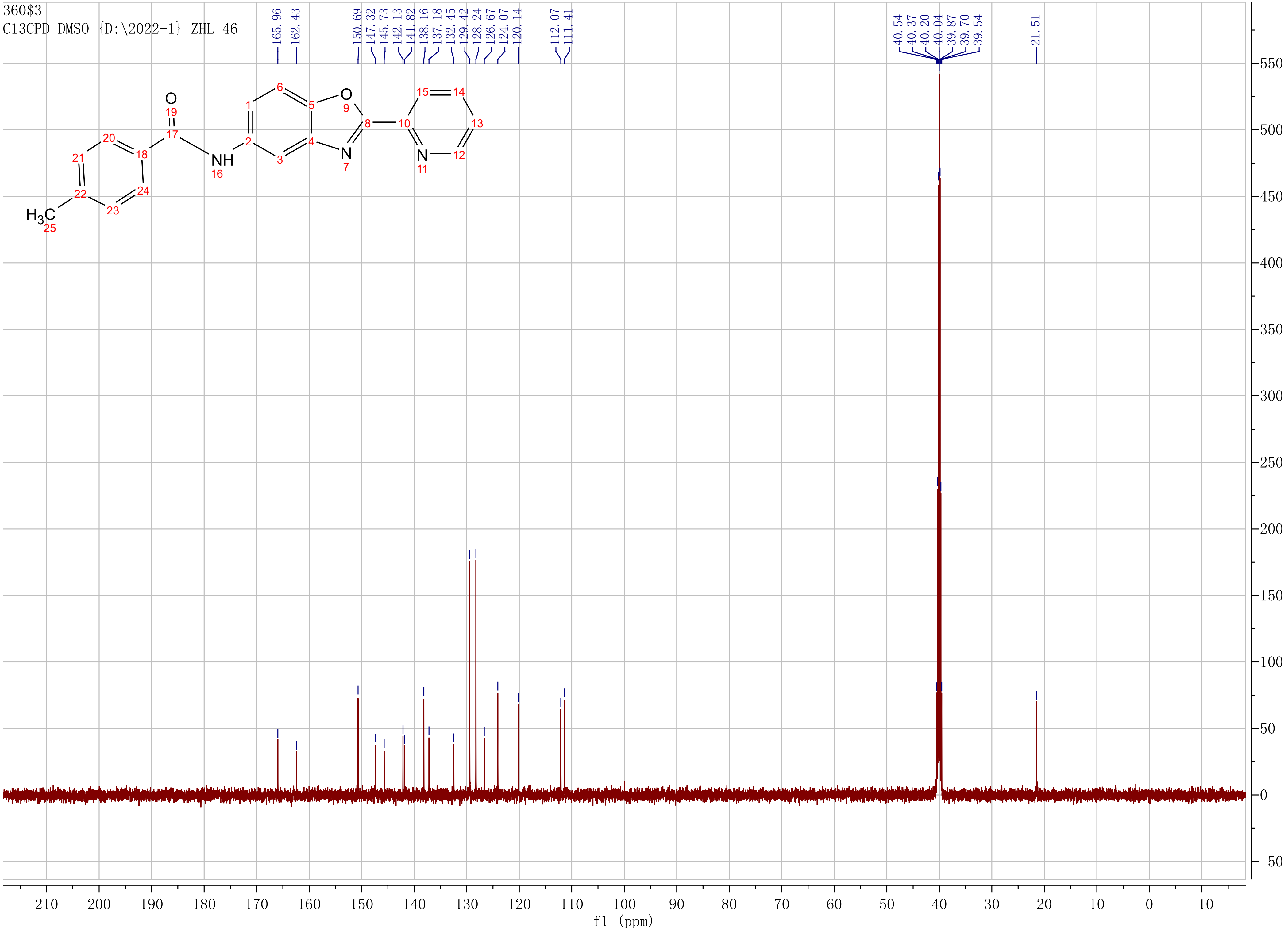
360$3
C13CPD DMSO {D:\2022-1} ZHL 46
165.96
162.43
150.69
147.32
145.73
142.13
141.82
138.16
137.18
132.45
129.42
128.24
126.67
124.07
120.14
112.07
111.41
40.54
40.37
40.20
40.04
39.87
39.70
39.54
21.51
f1 (ppm)

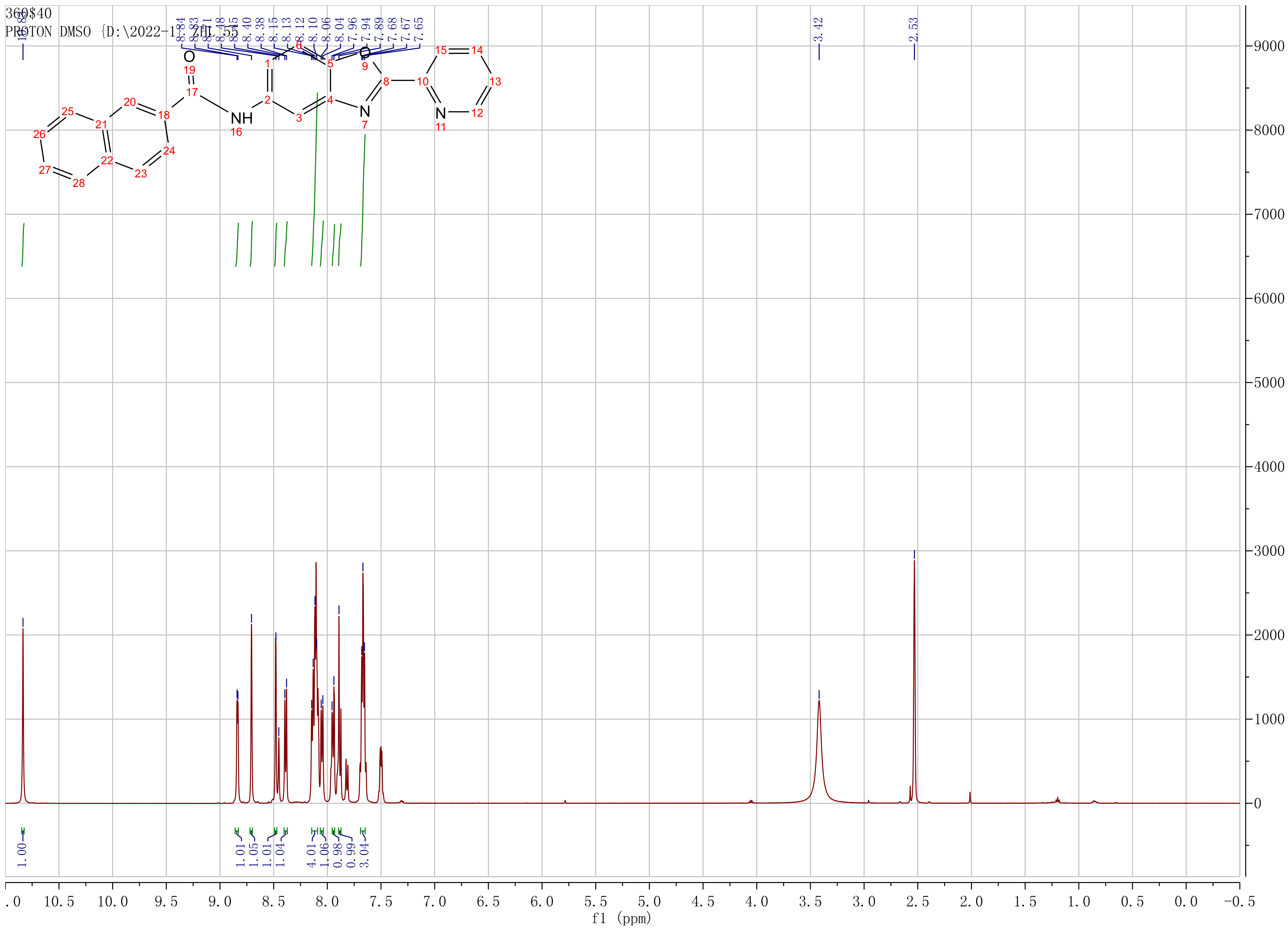
360$40
PROTON DMSO {D:\2022-1
8.84
8.83
8.71
8.48
8.45
8.40
8.38
8.15
8.13
8.12
8.10
8.06
8.04
7.96
7.94
7.89
7.68
7.67
7.65
3.42
2.53
1.00
1.01
1.05
1.01
1.04
4.01
1.06
0.98
0.99
3.04
f1 (ppm)

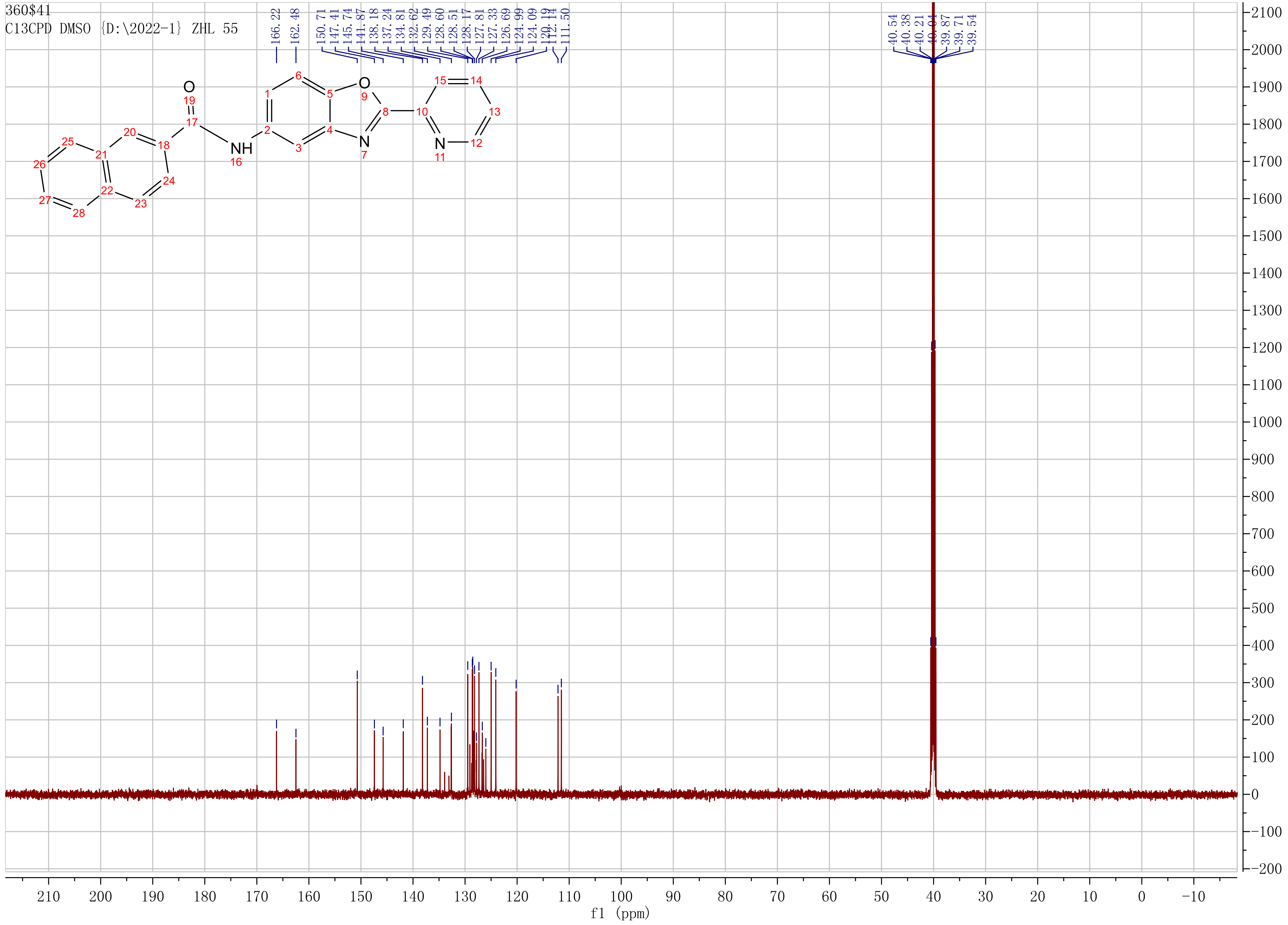

360$41
C13CPD DMSO {D:\2022-1} ZHL 55
166.22
162.48
150.71
147.41
145.74
141.87
138.18
137.24
134.81
132.62
129.49
128.60
128.51
128.17
127.81
127.33
126.69
124.99
124.09
120.19
112.14
111.50
40.54
40.38
40.21
40.04
39.87
39.71
39.54
NH
f1 (ppm)

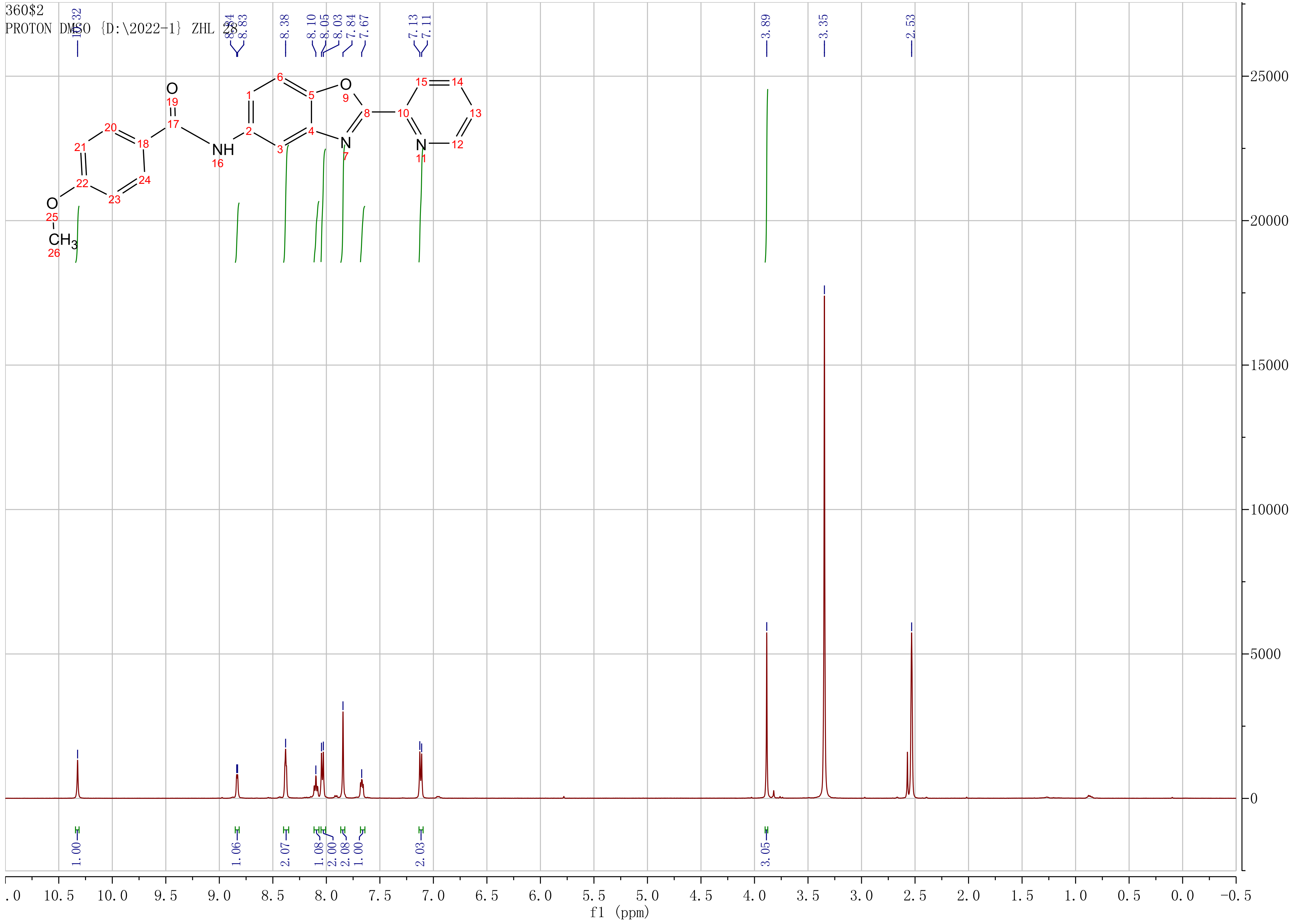
360$2
PROTON DMSO {D:\2022-1} ZHL 28
10.32
8.84
8.83
8.38
8.10
8.05
8.03
7.84
7.67
7.13
7.11
3.89
3.35
2.53
1.00
1.06
2.07
1.08
2.00
2.08
1.00
2.03
3.05
25000
20000
15000
10000
5000
0
f1 (ppm)
O
NH
N
CH3

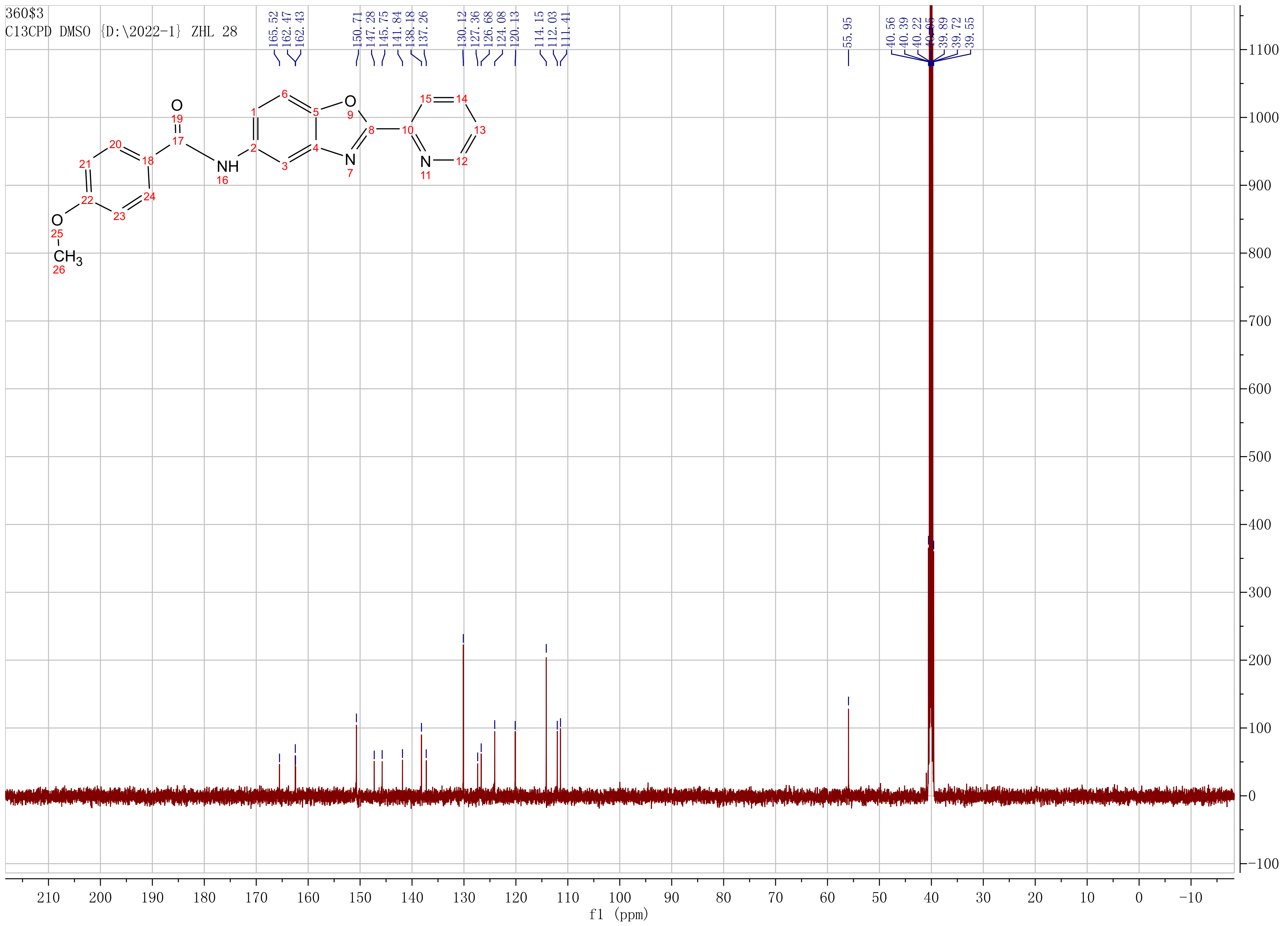

360$3
C13CPD DMSO {D:\2022-1} ZHL 28
165.52
162.47
162.43
150.71
147.28
145.75
141.84
138.18
137.26
130.12
127.36
126.68
124.08
120.13
114.15
112.03
111.41
55.95
40.56
40.39
40.22
39.89
39.72
39.55
f1 (ppm)

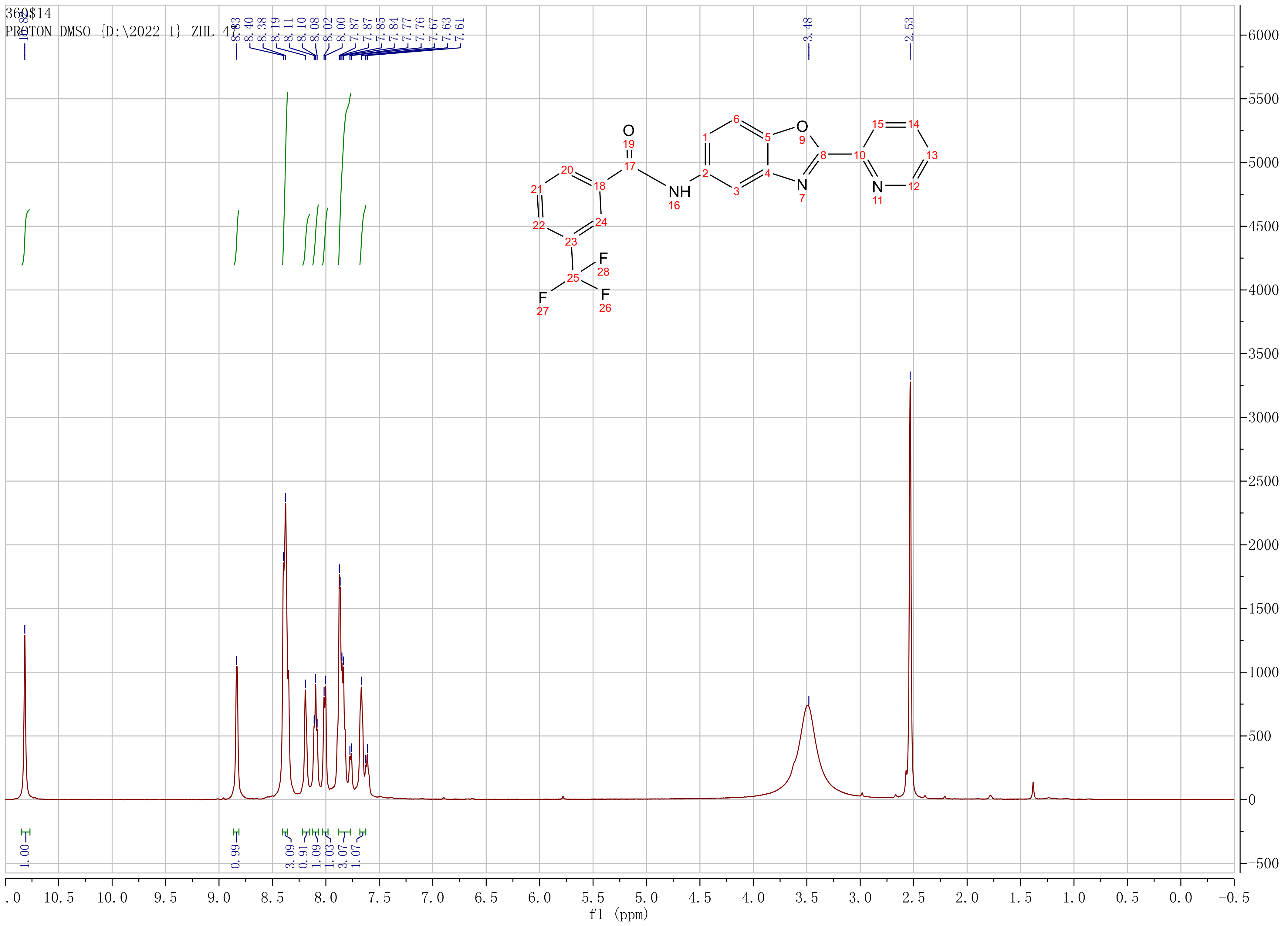

360$14
PROTON DMSO {D:\2022-1} ZHL 47
10.83
8.83
8.40
8.38
8.19
8.11
8.10
8.08
8.02
8.00
7.87
7.87
7.85
7.84
7.77
7.76
7.67
7.63
7.61
3.48
2.53
1.00
0.99
3.09
0.91
1.09
1.03
3.07
1.07
f1 (ppm)

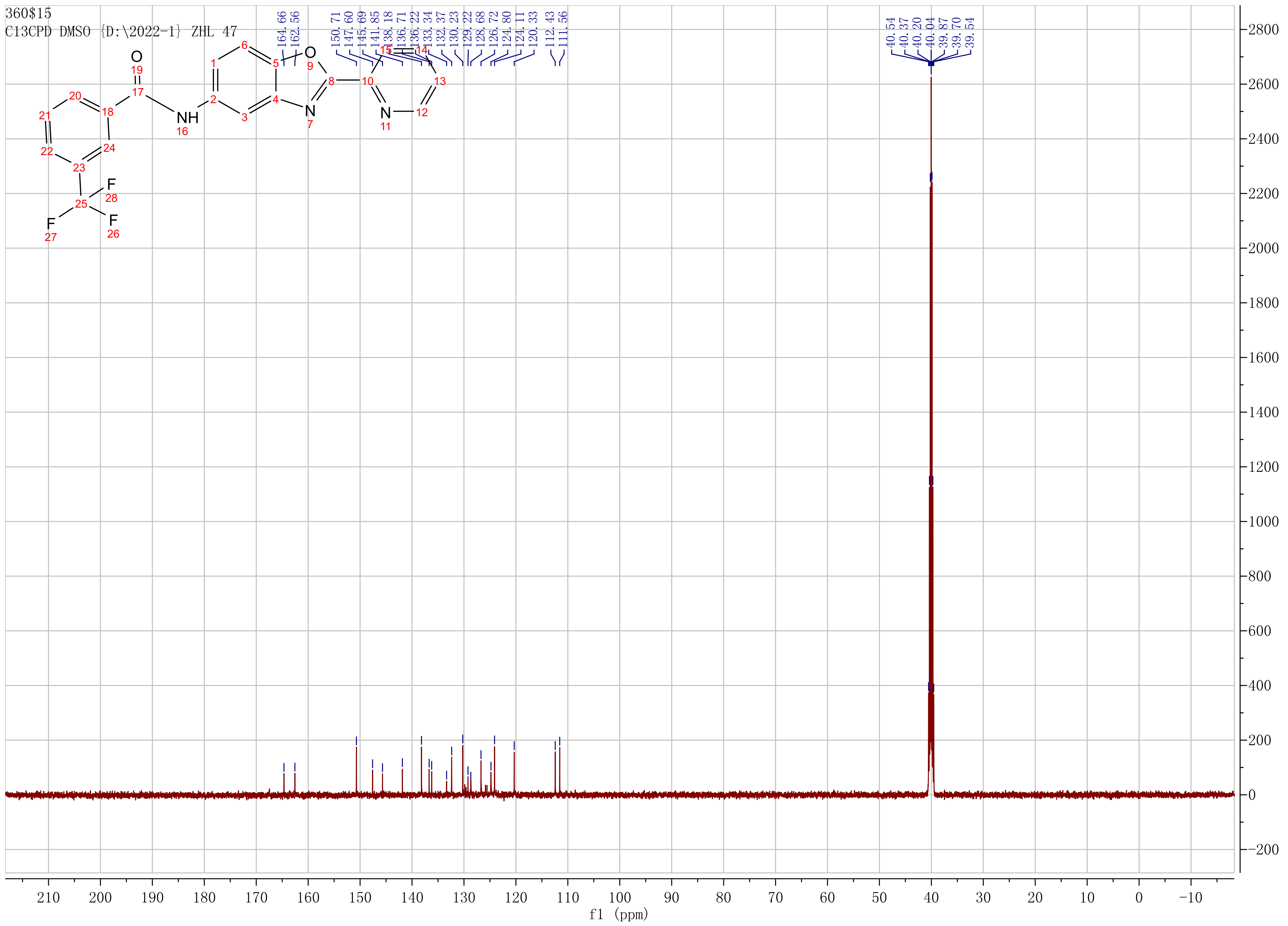

360$15
C13CPD DMSO {D:\2022-1} ZHL 47
164.66
162.56
150.71
147.60
145.69
141.85
138.18
136.71
136.22
133.34
132.37
130.23
129.22
128.68
126.72
124.80
124.11
120.33
112.43
111.56
40.54
40.37
40.20
40.04
39.87
39.70
39.54
f1 (ppm)

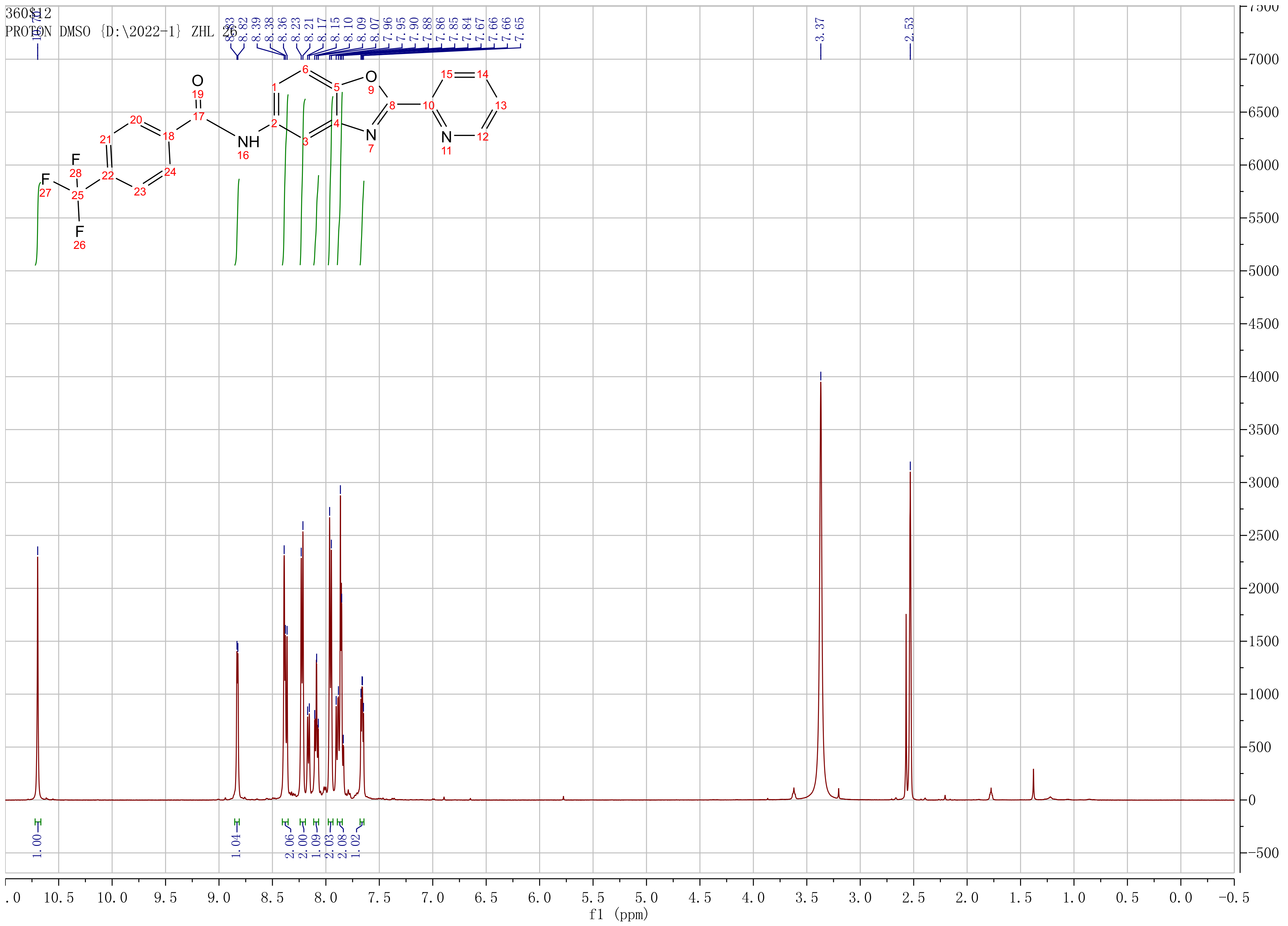

360812
PROTON DMSO {D:\2022-1} ZHL 26
10.71
8.83 8.82 8.39 8.38 8.36 8.23 8.21 8.17 8.15 8.10 8.09 8.07 7.96 7.95 7.90 7.88 7.86 7.85 7.84 7.67 7.66 7.66 7.65
3.37
2.53
1.00 1.04 2.06 2.00 1.09 2.03 2.08 1.02
f1 (ppm)

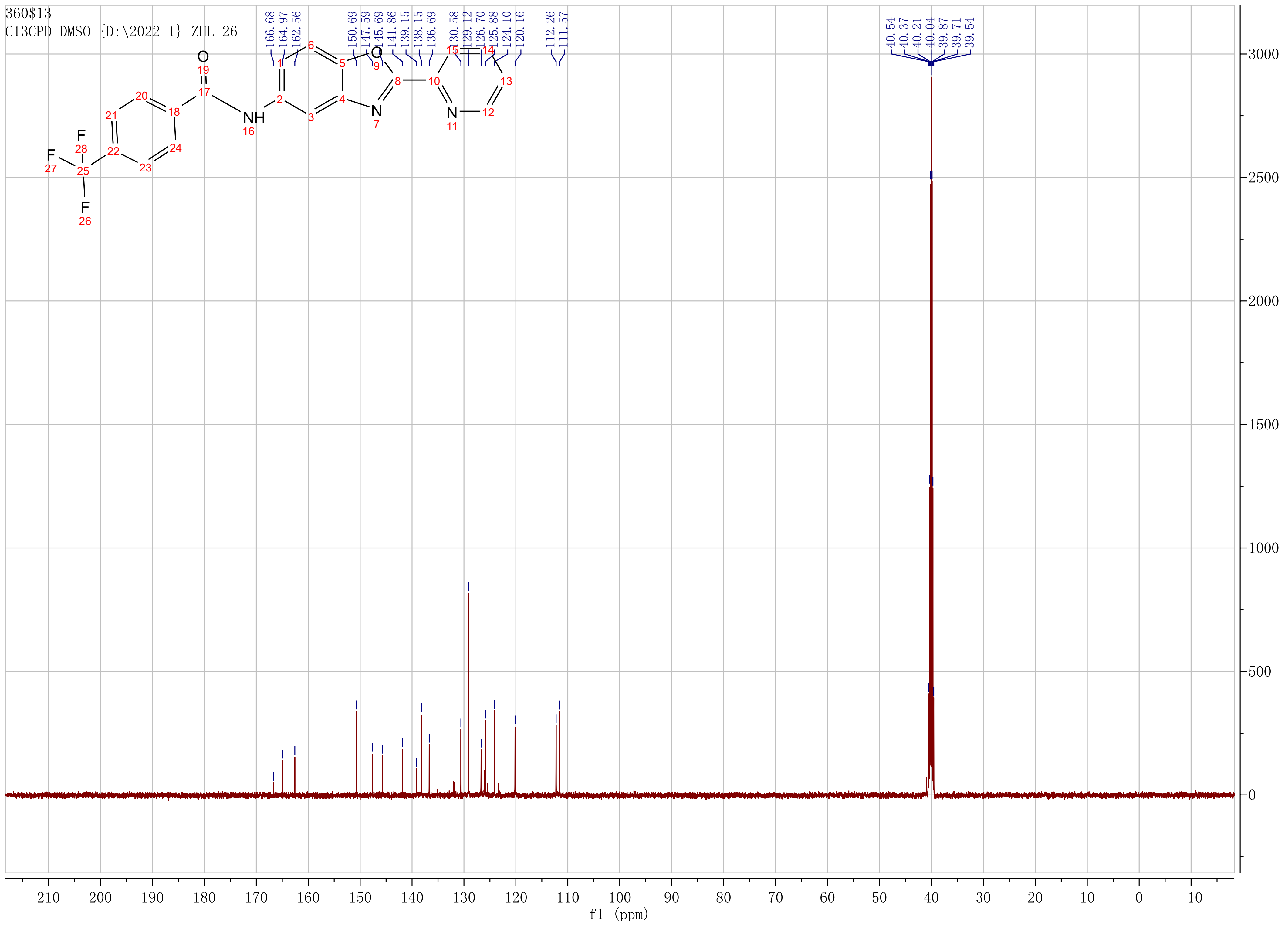
360$13
C13CPD DMSO {D:\2022-1} ZHL 26
166.68
164.97
162.56
150.69
147.59
145.69
141.86
139.15
138.15
136.69
130.58
129.12
126.70
125.88
124.10
120.16
112.26
111.57
40.54
40.37
40.21
40.04
39.87
39.71
39.54
f1 (ppm)

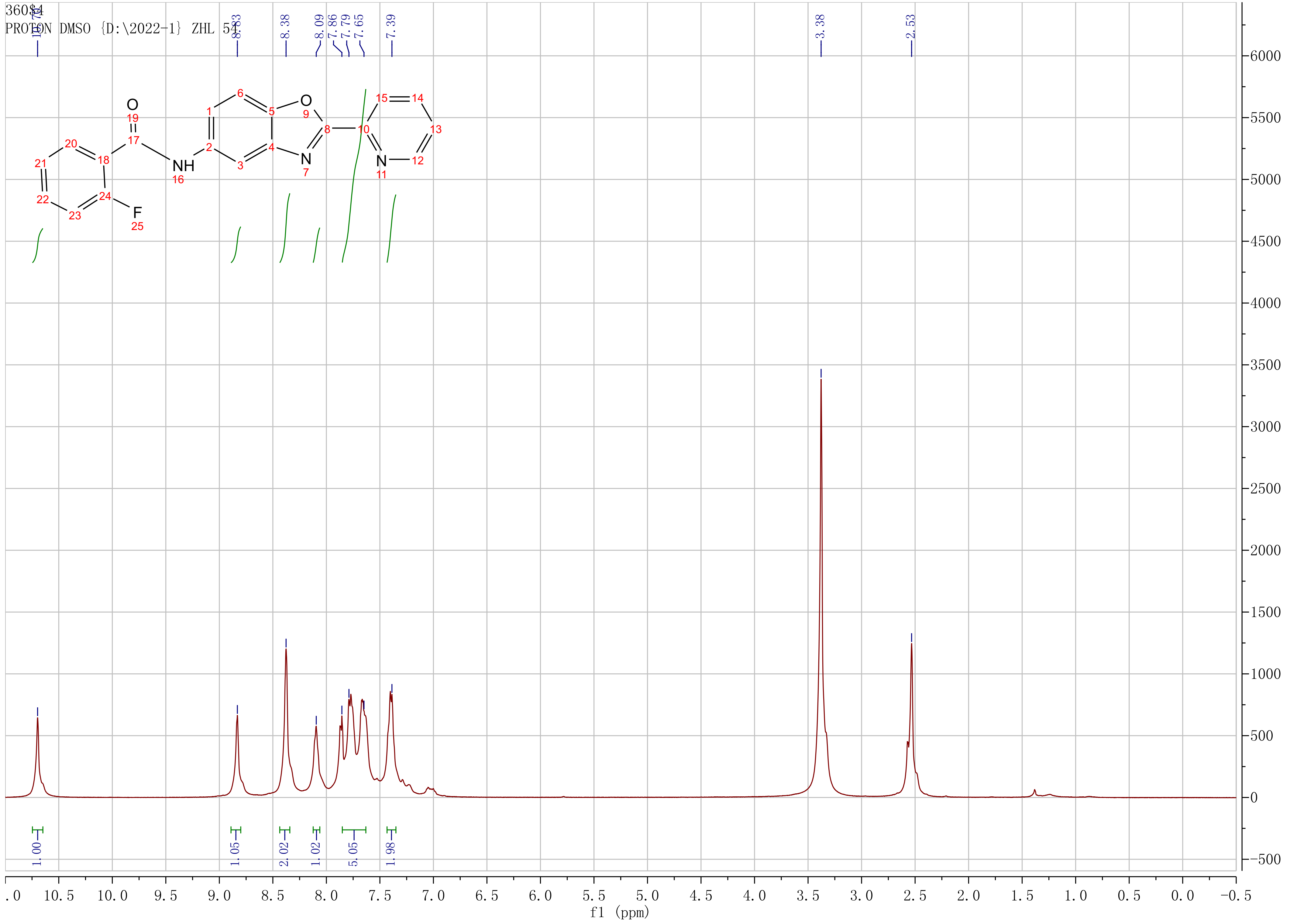
36034
PROTON DMSO {D:\2022-1} ZHL 54
10.70
8.83
8.38
8.09
7.86
7.79
7.65
7.39
3.38
2.53
1.00
1.05
2.02
1.02
5.05
1.98
f1 (ppm)

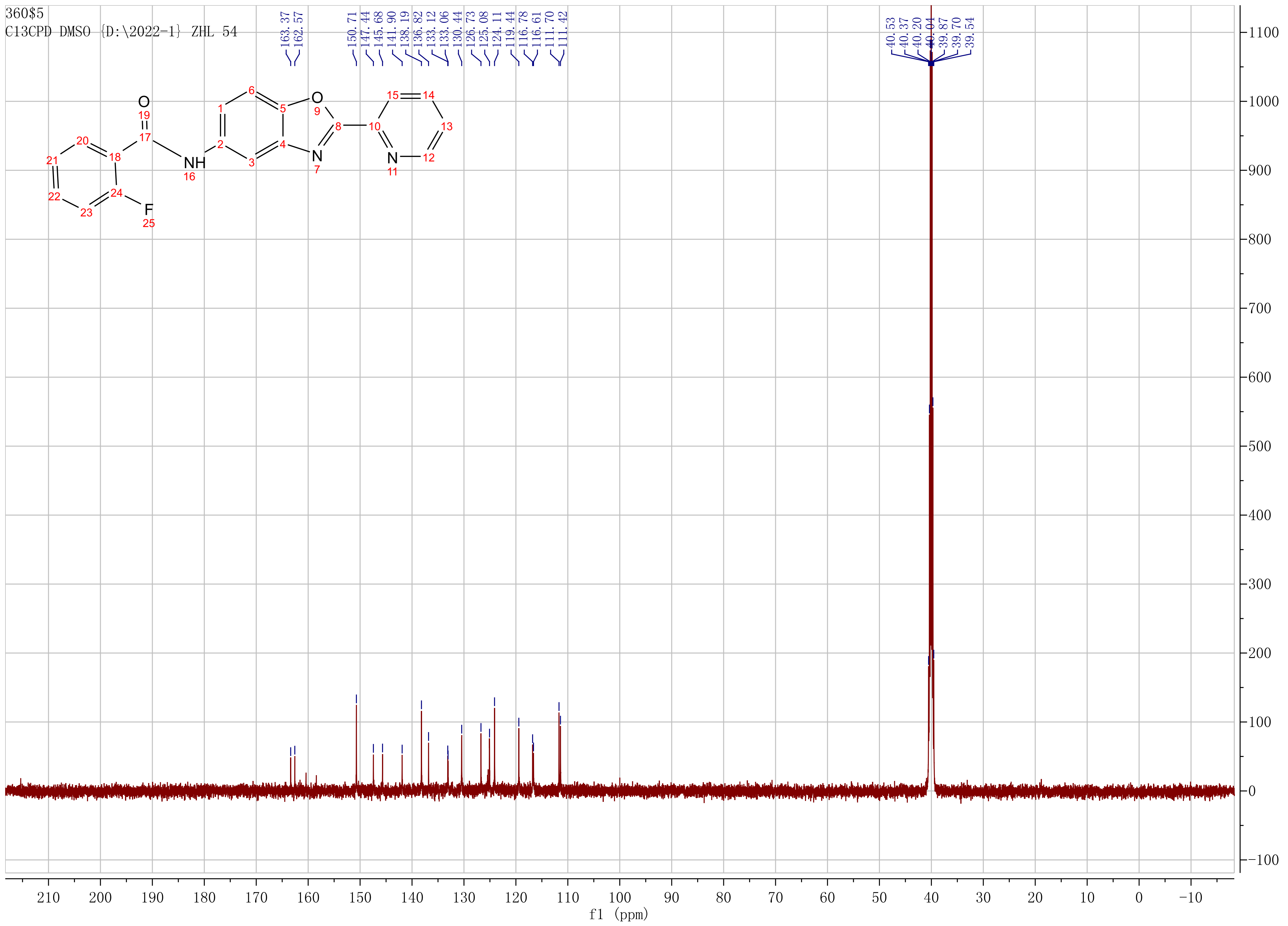

360$5
C13CPD DMSO {D:\2022-1} ZHL 54
163.37
162.57
150.71
147.44
145.68
141.90
138.19
136.82
133.12
133.06
130.44
126.73
125.08
124.11
119.44
116.78
116.61
111.70
111.42
40.53
40.37
40.20
40.04
39.87
39.70
39.54
f1 (ppm)

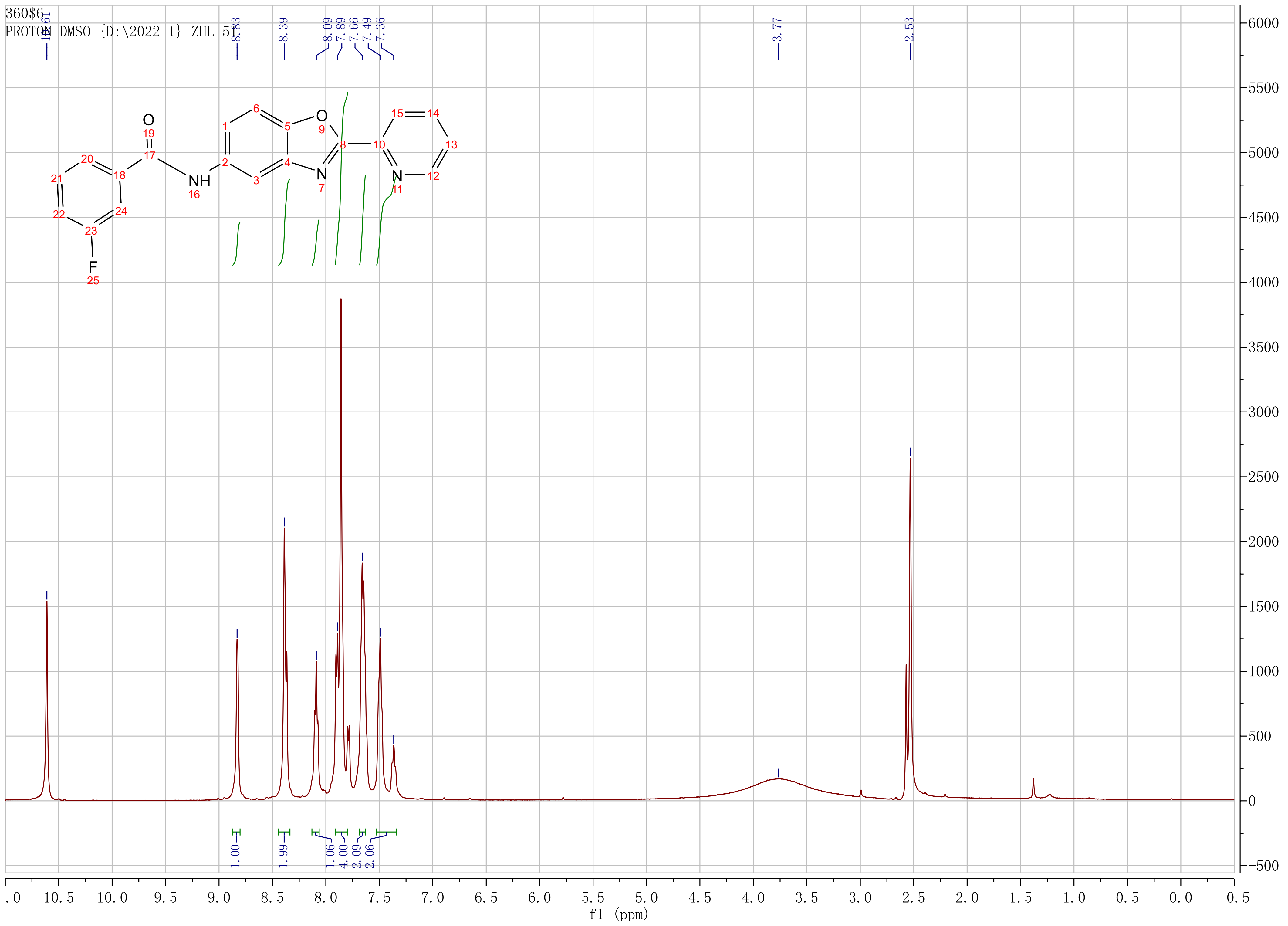

360$6
PROTON DMSO {D:\2022-1} ZHL 51
10.61
8.83
8.39
8.09
7.89
7.66
7.49
7.36
3.77
2.53
1.00
1.99
1.06
4.00
2.09
2.06
f1 (ppm)

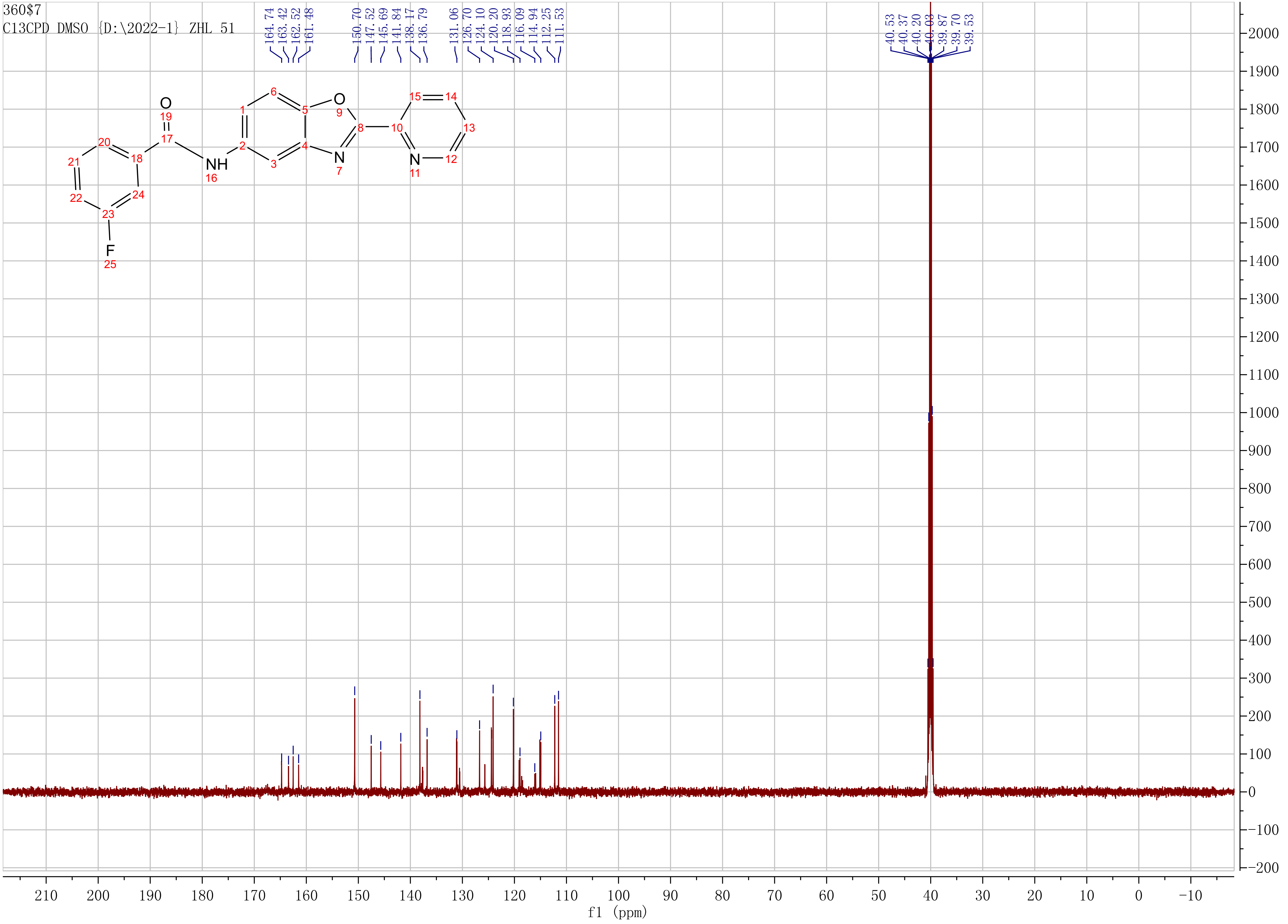

360$7
C13CPD DMSO {D:\2022-1} ZHL 51
164.74
163.42
162.52
161.48
150.70
147.52
145.69
141.84
138.17
136.79
131.06
126.70
124.10
120.20
118.93
116.09
114.94
112.25
111.53
40.53
40.37
40.20
40.03
39.87
39.70
39.53
f1 (ppm)

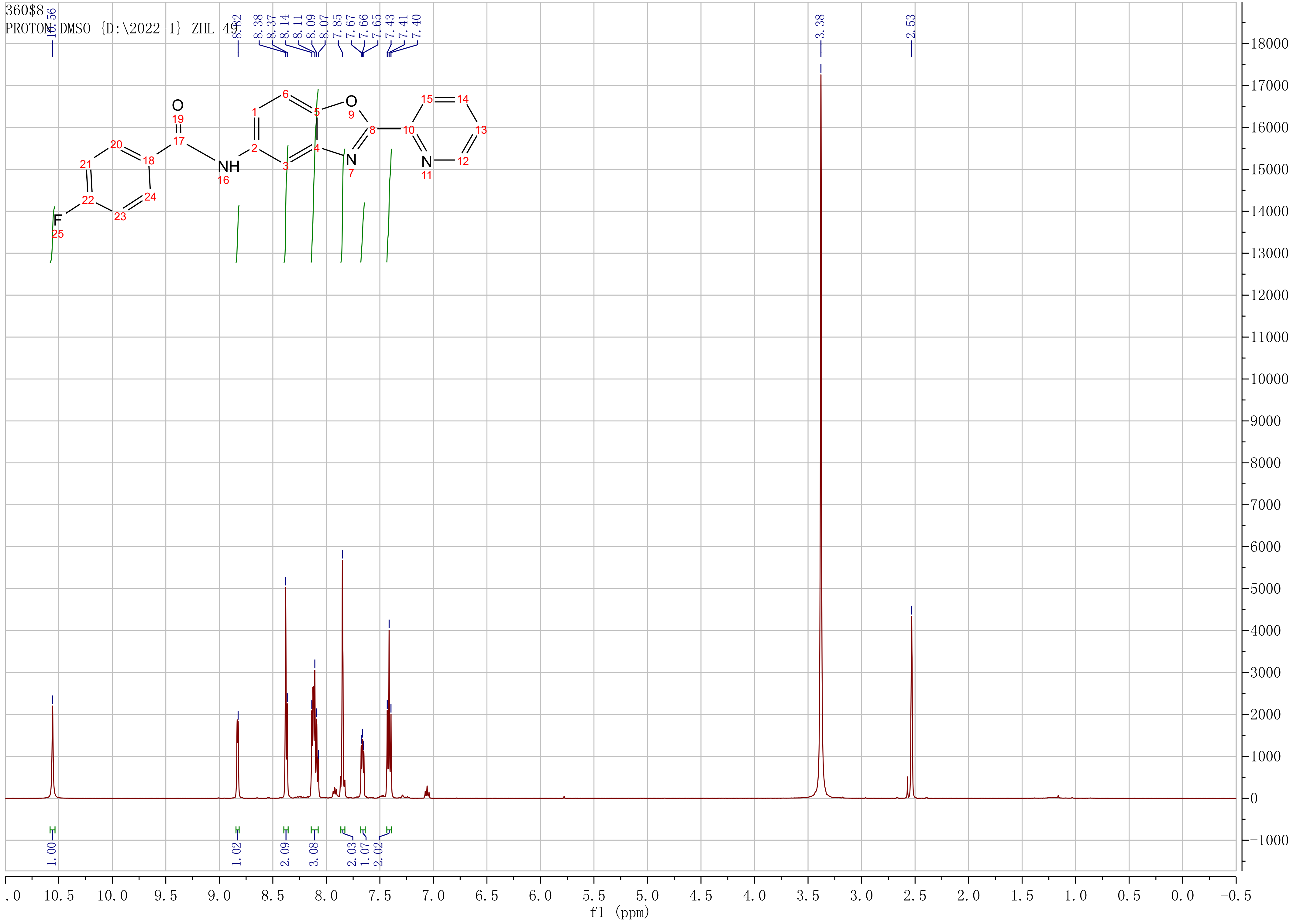
360$8
PROTON DMSO {D:\2022-1} ZHL 49
10.56
8.82
8.38
8.37
8.14
8.11
8.09
8.07
7.85
7.67
7.66
7.65
7.43
7.41
7.40
3.38
2.53
1.00
1.02
2.09
3.08
2.03
1.07
2.02
f1 (ppm)

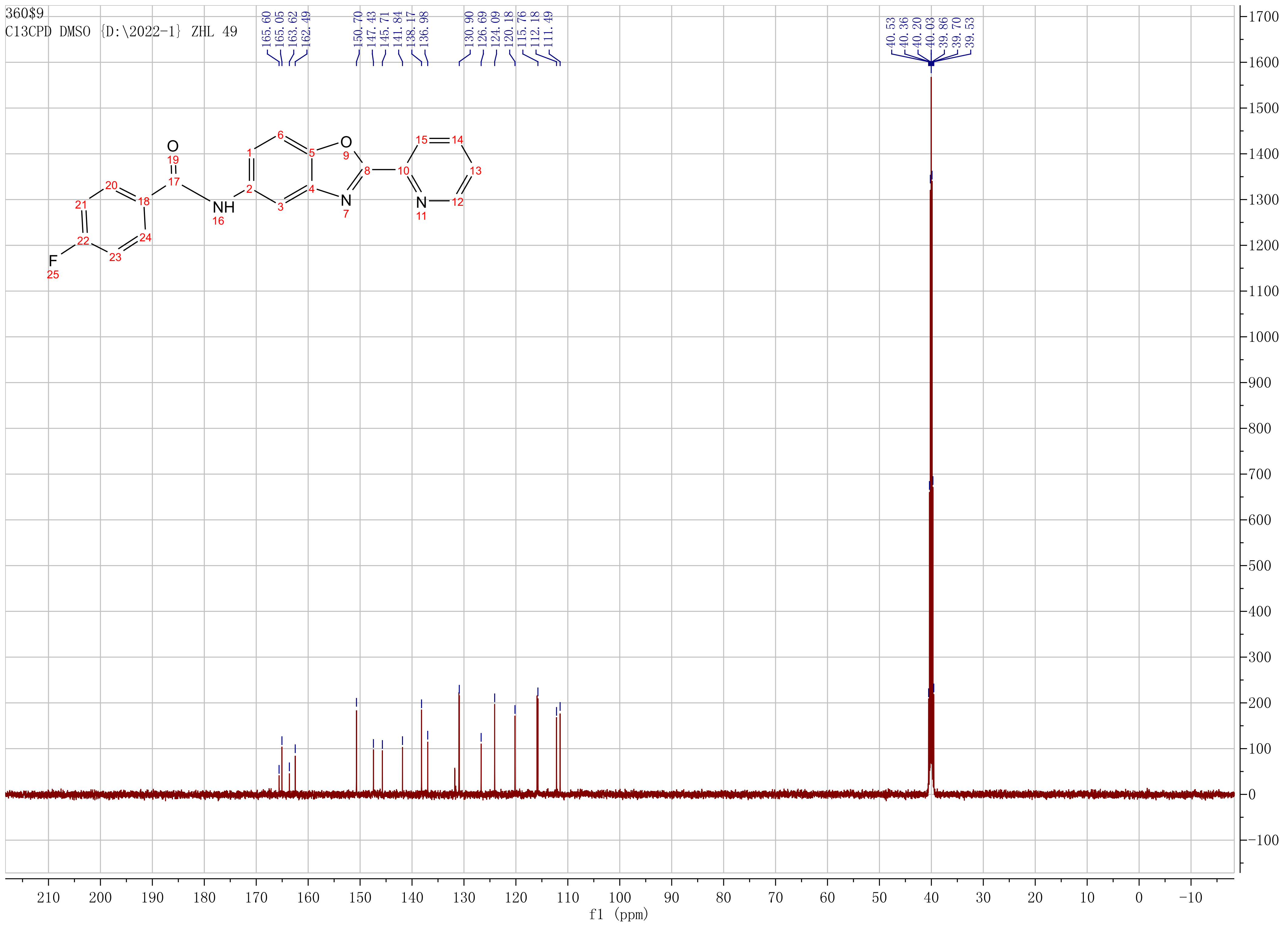
360$9
C13CPD DMSO {D:\2022-1} ZHL 49
165.60
165.05
163.62
162.49
150.70
147.43
145.71
141.84
138.17
136.98
130.90
126.69
124.09
120.18
115.76
112.18
111.49
40.53
40.36
40.20
40.03
39.86
39.70
39.53
f1 (ppm)

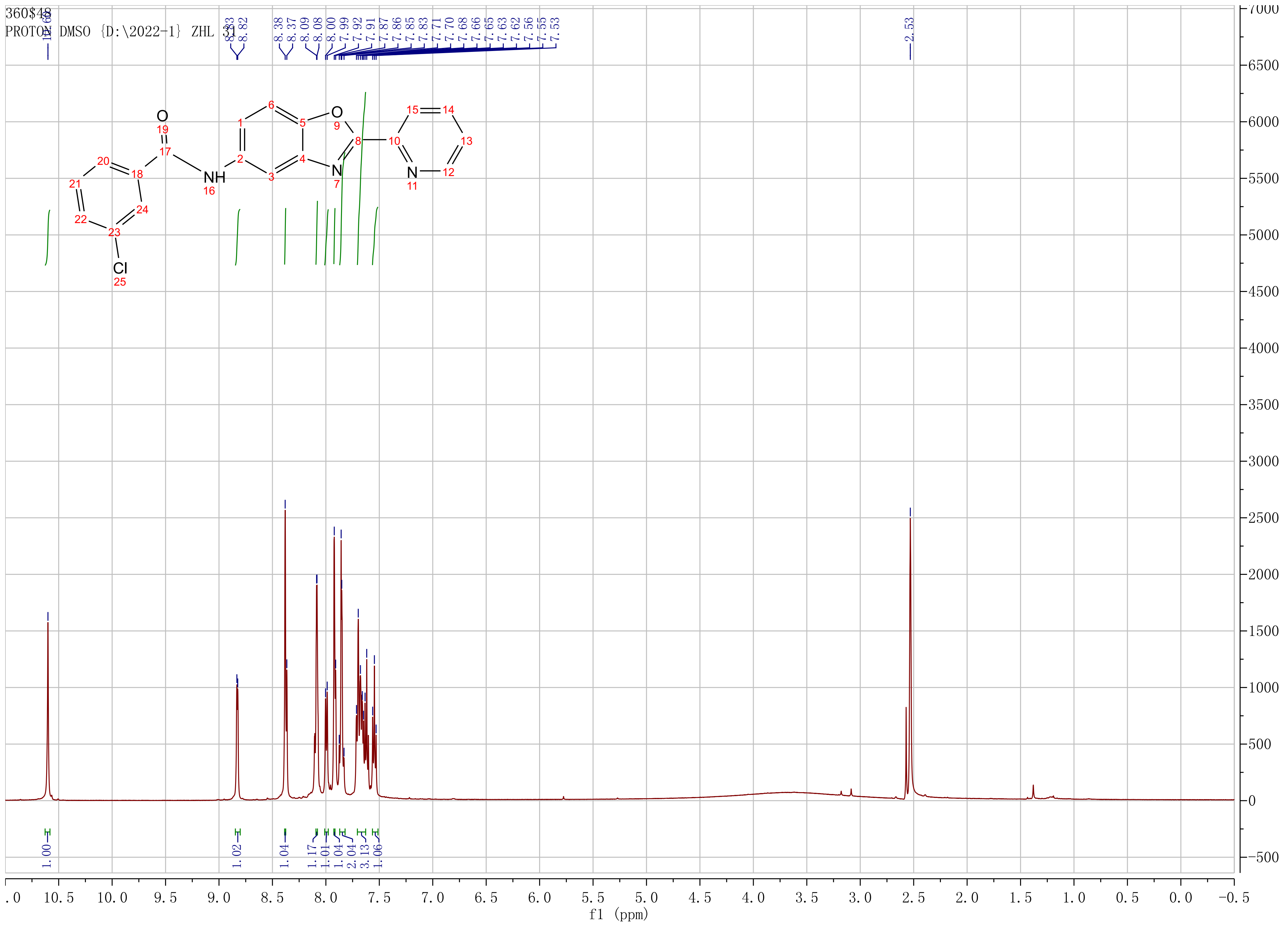
360$48
PROTON DMSO {D:\2022-1} ZHL 31
10.60
8.83
8.82
8.38
8.37
8.09
8.08
8.00
7.99
7.92
7.91
7.87
7.86
7.85
7.83
7.71
7.70
7.68
7.66
7.65
7.63
7.62
7.56
7.55
7.53
2.53
1.00
1.02
1.04
1.17
1.01
1.04
2.04
3.13
1.06
f1 (ppm)

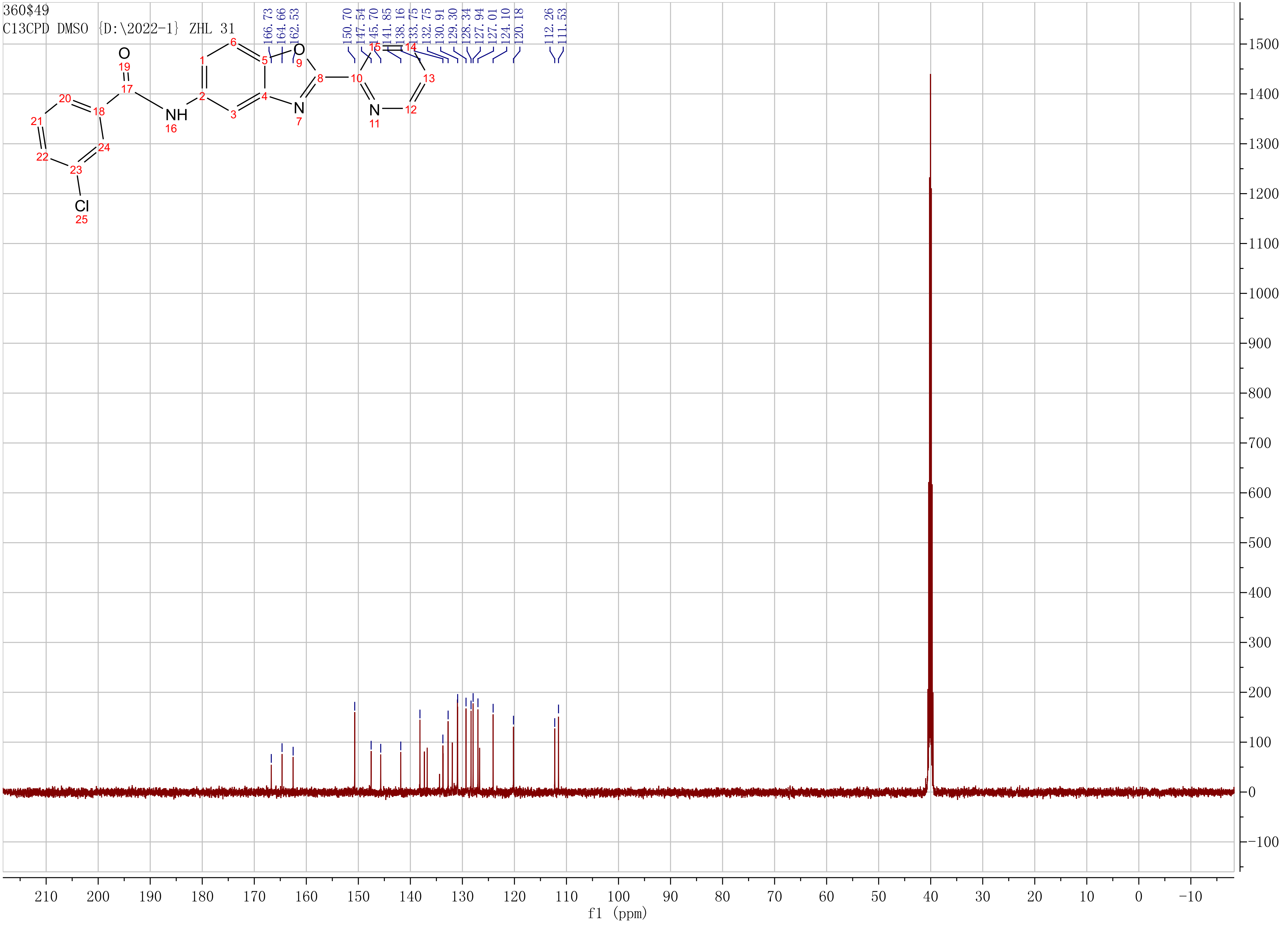
360$49
C13CPD DMSO {D:\2022-1} ZHL 31
166.73
164.66
162.53
150.70
147.54
145.70
141.85
138.16
133.75
132.75
130.91
129.30
128.34
127.94
127.01
124.10
120.18
112.26
111.53
O
NH
N
Cl
f1 (ppm)

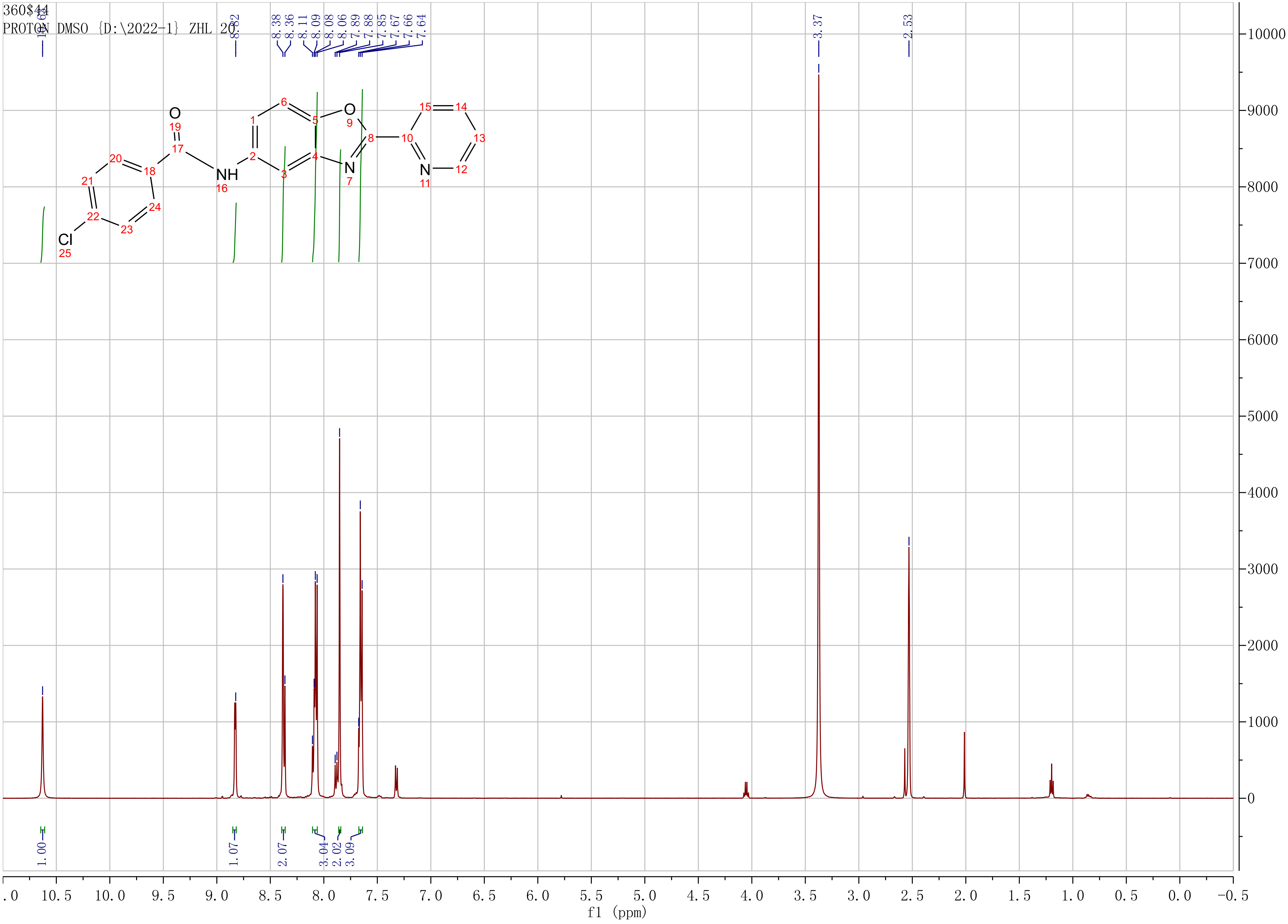

360$44
PROTON DMSO {D:\2022-1} ZHL 20
10.63
8.82
8.38
8.36
8.11
8.09
8.08
8.06
7.89
7.88
7.85
7.67
7.66
7.64
3.37
2.53
1.00
1.07
2.07
3.04
2.02
3.09
f1 (ppm)

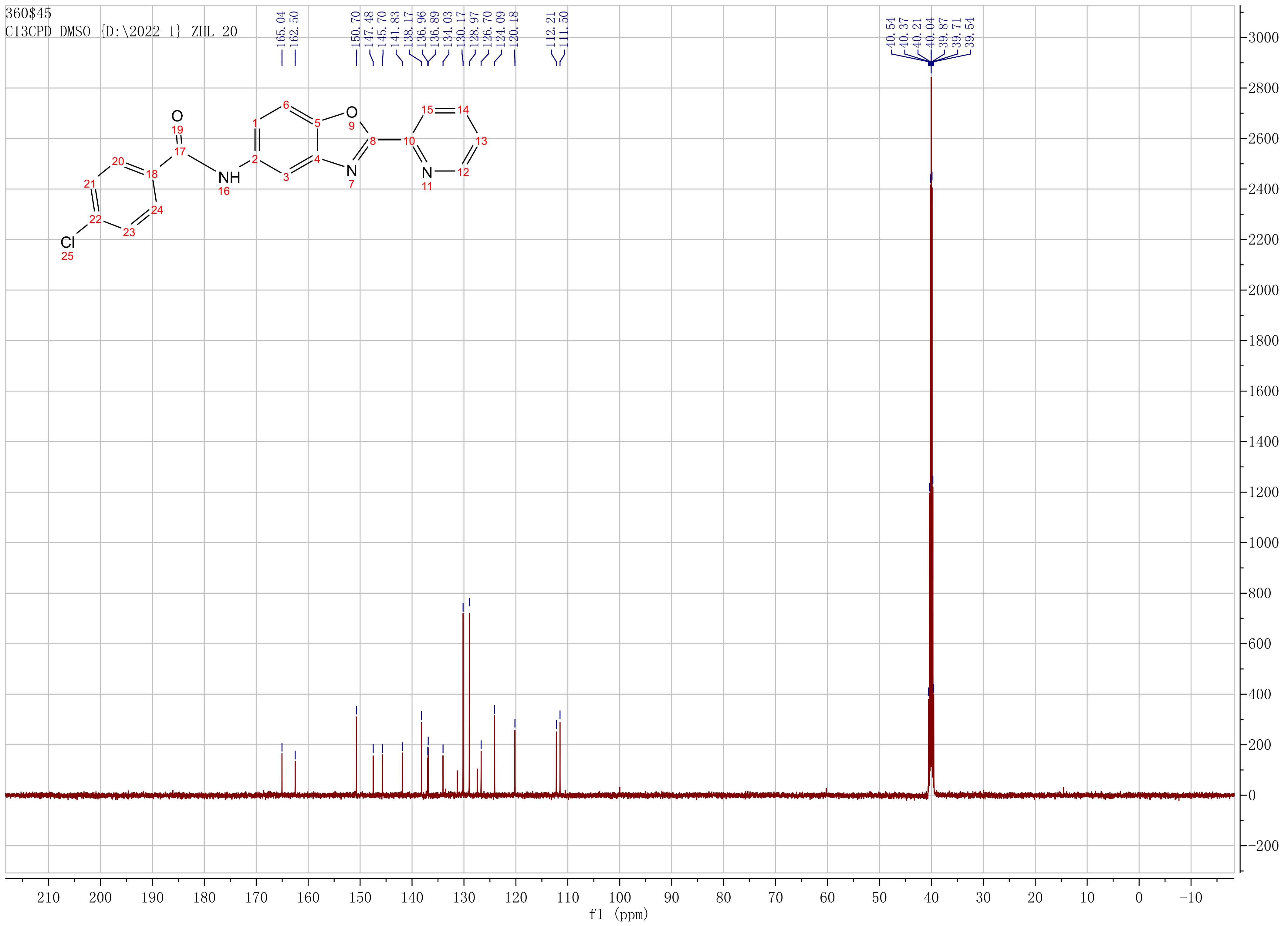
360$45
C13CPD DMSO {D:\2022-1} ZHL 20
165.04
162.50
150.70
147.48
145.70
141.83
138.17
136.96
136.89
134.03
130.17
128.97
126.70
124.09
120.18
112.21
111.50
40.54
40.37
40.21
40.04
39.87
39.71
39.54
f1 (ppm)

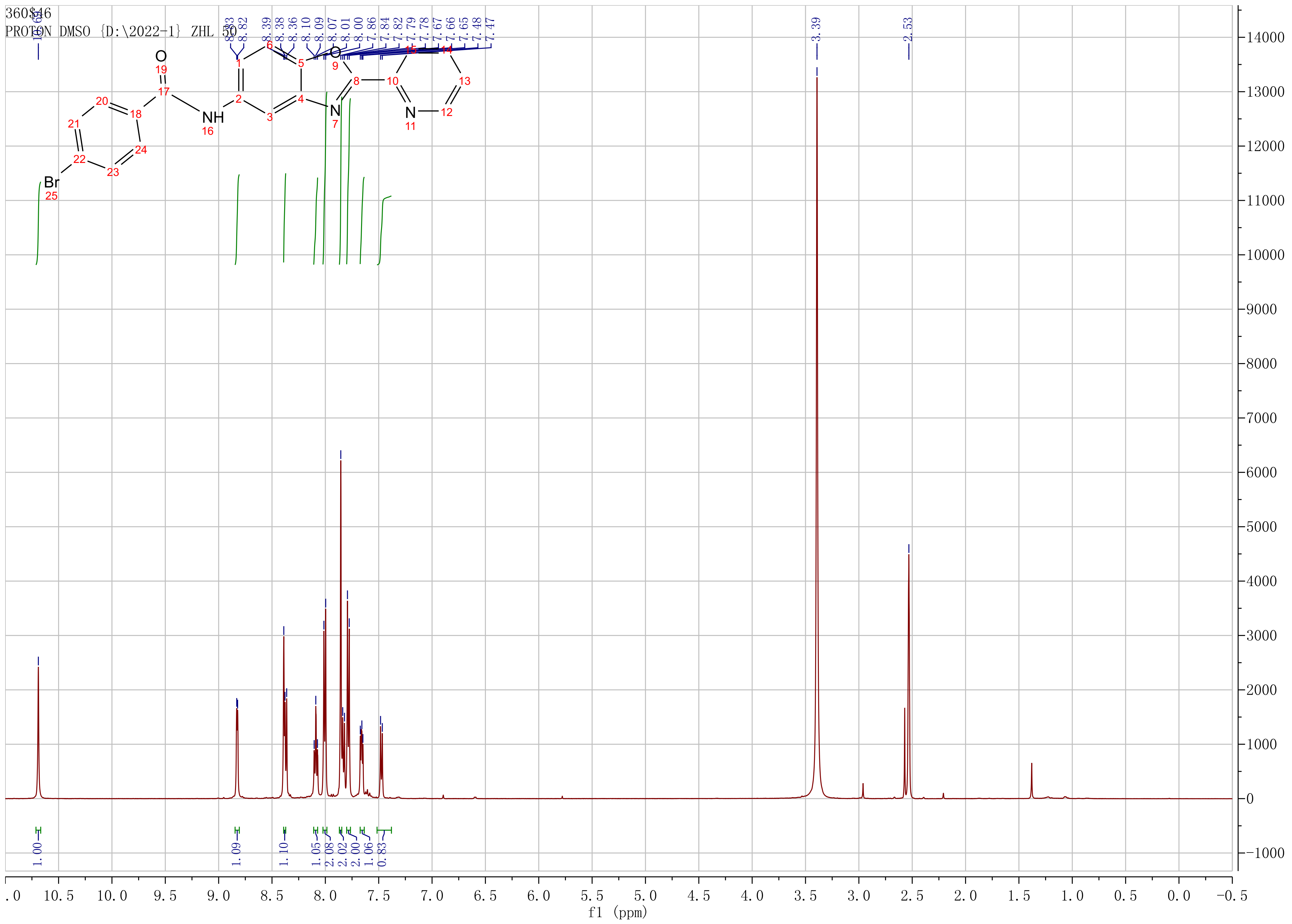
360$46
PROTON DMSO {D:\2022-1} ZHL 50
8.83 8.82 8.39 8.38 8.36 8.10 8.09 8.07 8.01 8.00 7.86 7.84 7.82 7.79 7.78 7.67 7.66 7.65 7.48 7.47
3.39
2.53
1.00 1.09 1.10 1.05 2.08 2.02 2.00 1.06 0.83
f1 (ppm)

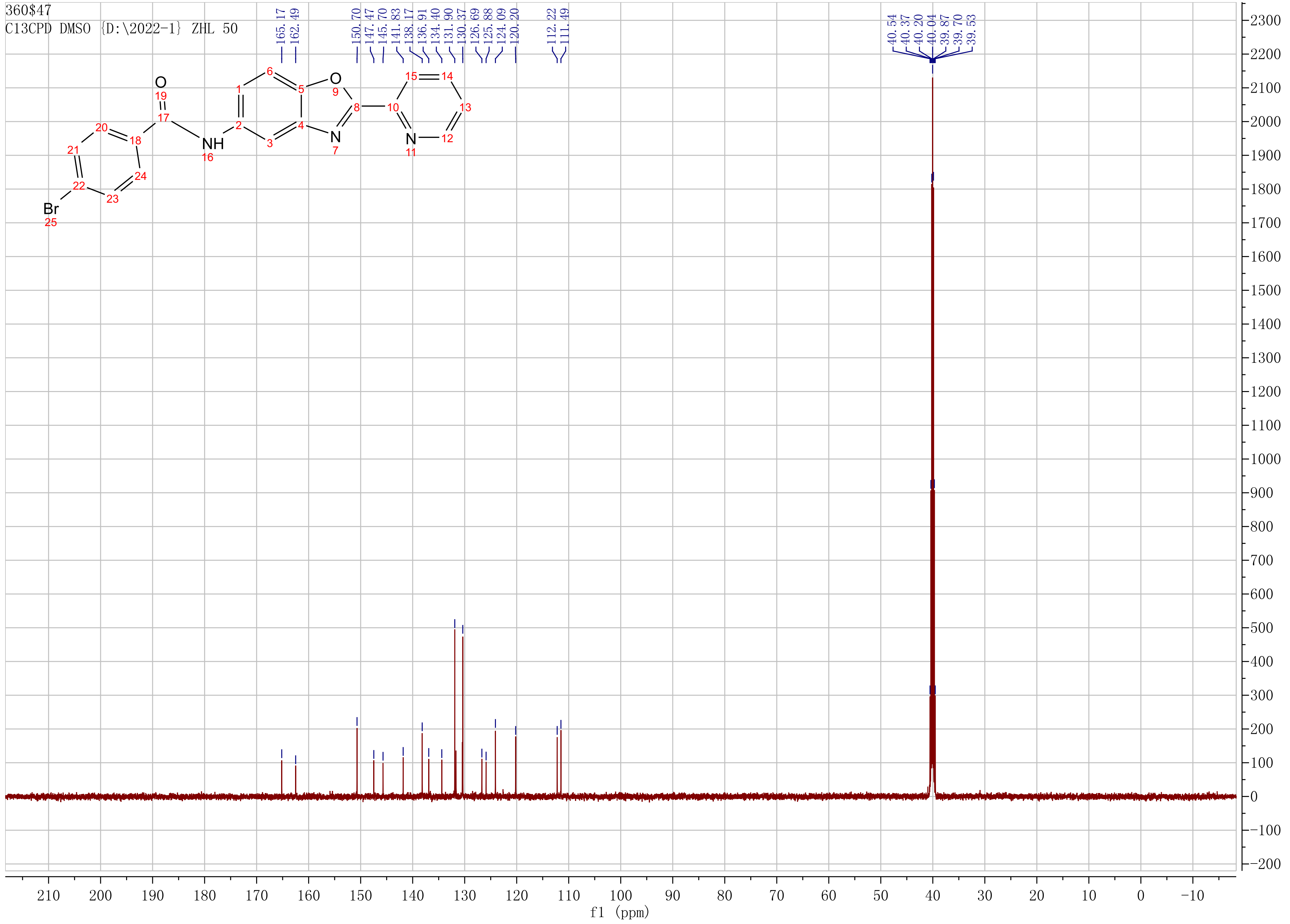
360$47
C13CPD DMSO {D:\2022-1} ZHL 50
165.17
162.49
150.70
147.47
145.70
141.83
138.17
136.91
134.40
131.90
130.37
126.69
125.88
124.09
120.20
112.22
111.49
40.54
40.37
40.20
40.04
39.87
39.70
39.53
Br
NH
O
N
f1 (ppm)

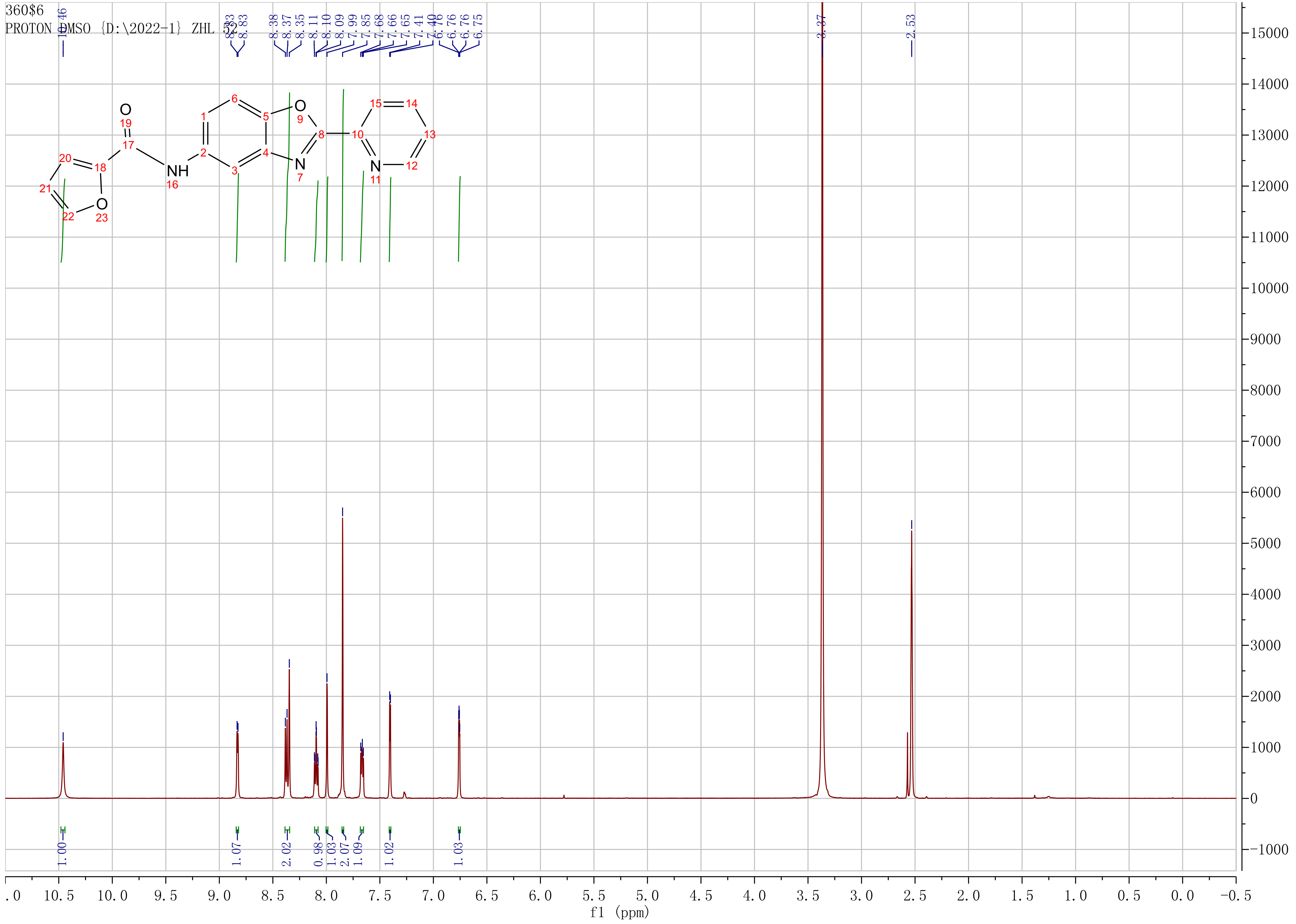

360$6
PROTON DMSO {D:\2022-1} ZHL 52
10.46
8.83
8.83
8.38
8.37
8.35
8.11
8.10
8.09
7.99
7.85
7.68
7.66
7.65
7.41
7.40
6.76
6.76
6.76
6.75
3.37
2.53
O
19
17
NH
16
1
2
3
4
5
6
O
9
8
N
7
10
15
14
13
12
N
11
20
18
21
22
O
23
1.00
1.07
2.02
0.98
1.03
2.07
1.09
1.02
1.03
f1 (ppm)

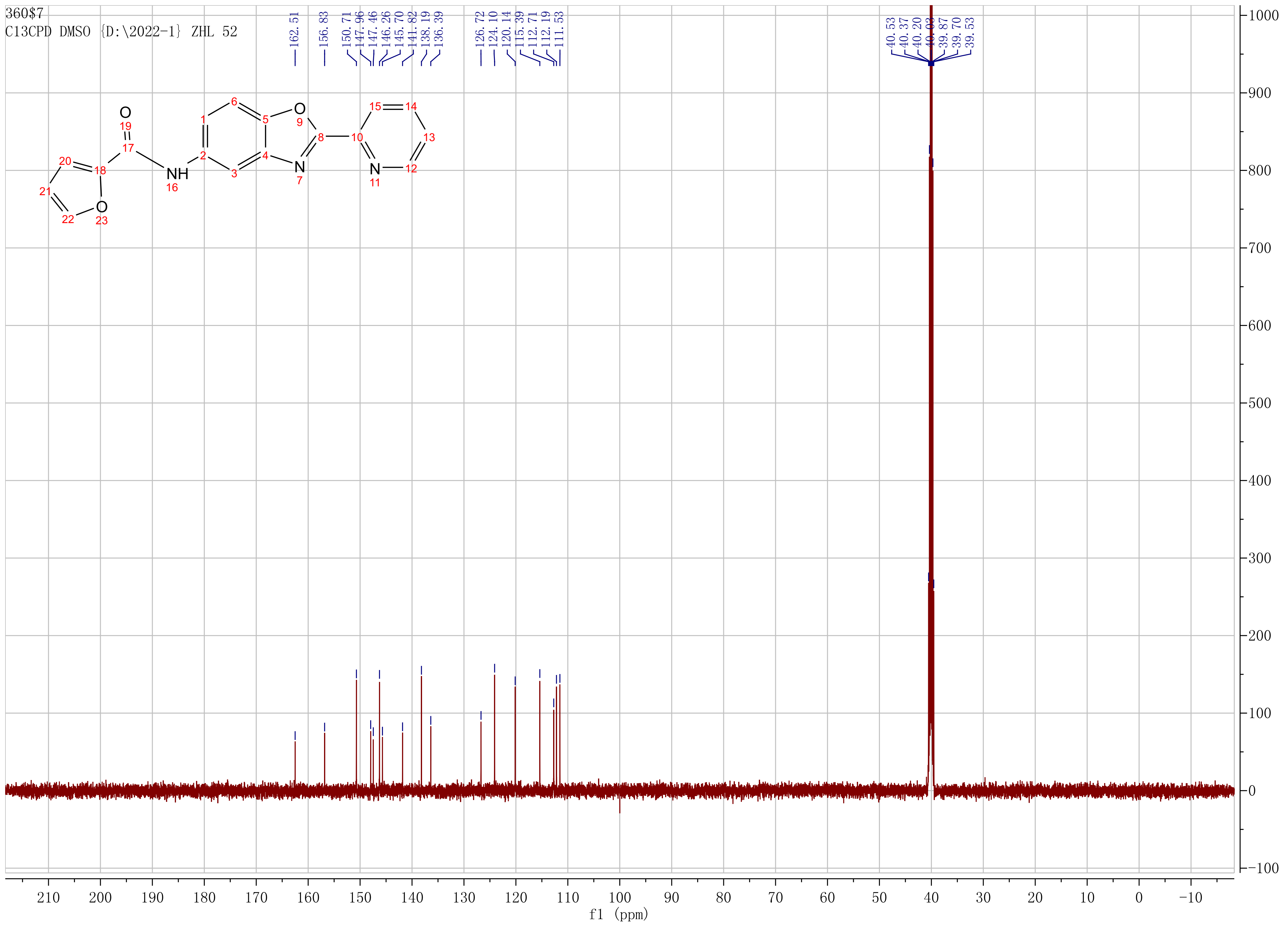

360$7
C13CPD DMSO {D:\2022-1} ZHL 52
162.51
156.83
150.71
147.96
147.46
146.26
145.70
141.82
138.19
136.39
126.72
124.10
120.14
115.39
112.71
112.19
111.53
40.53
40.37
40.20
40.03
39.87
39.70
39.53
f1 (ppm)

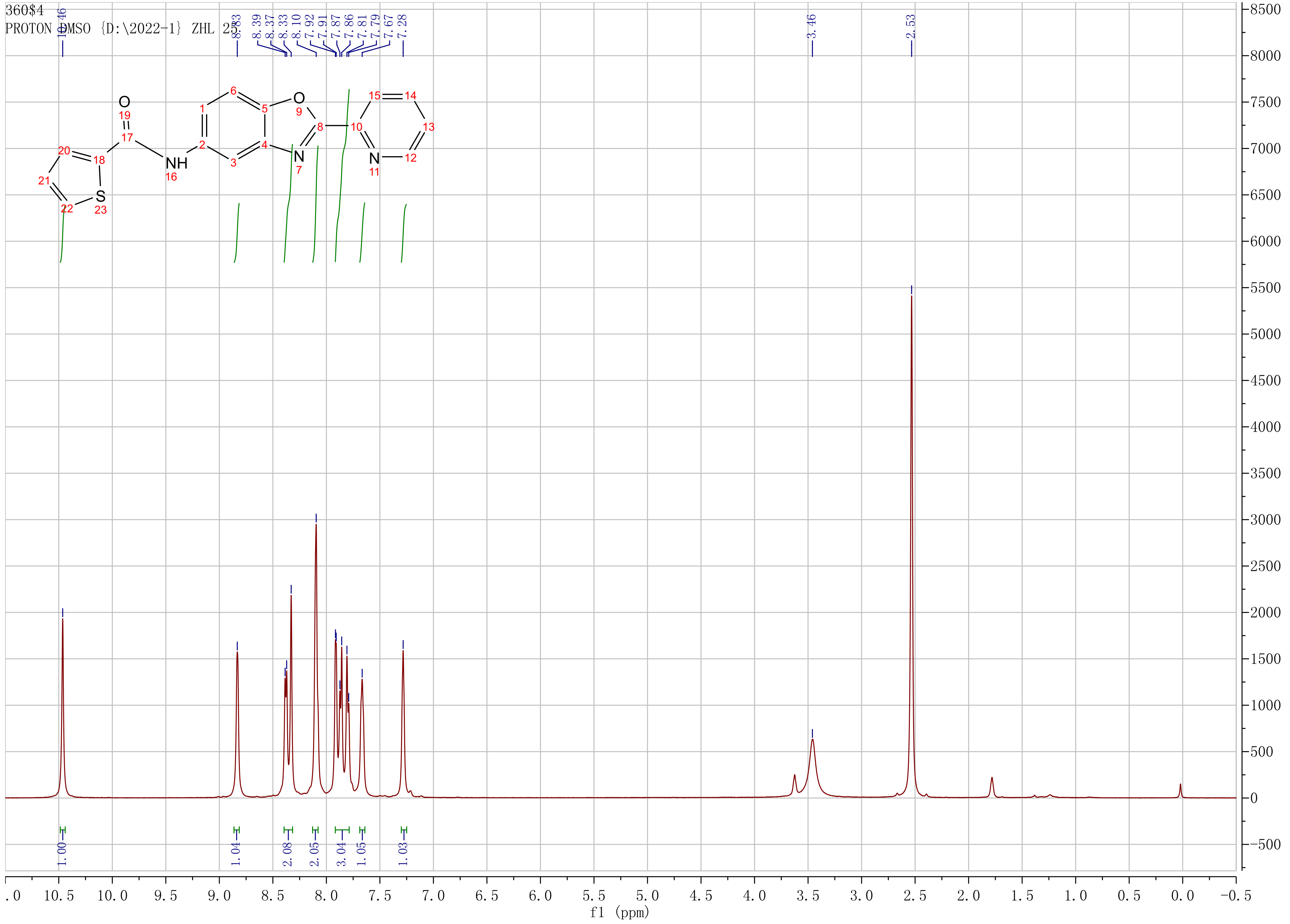

360$4
PROTON DMSO {D:\2022-1} ZHL 25
10.46
8.83
8.39
8.37
8.33
8.10
7.92
7.91
7.87
7.86
7.81
7.79
7.67
7.28
3.46
2.53
1.00
1.04
2.08
2.05
3.04
1.05
1.03
f1 (ppm)

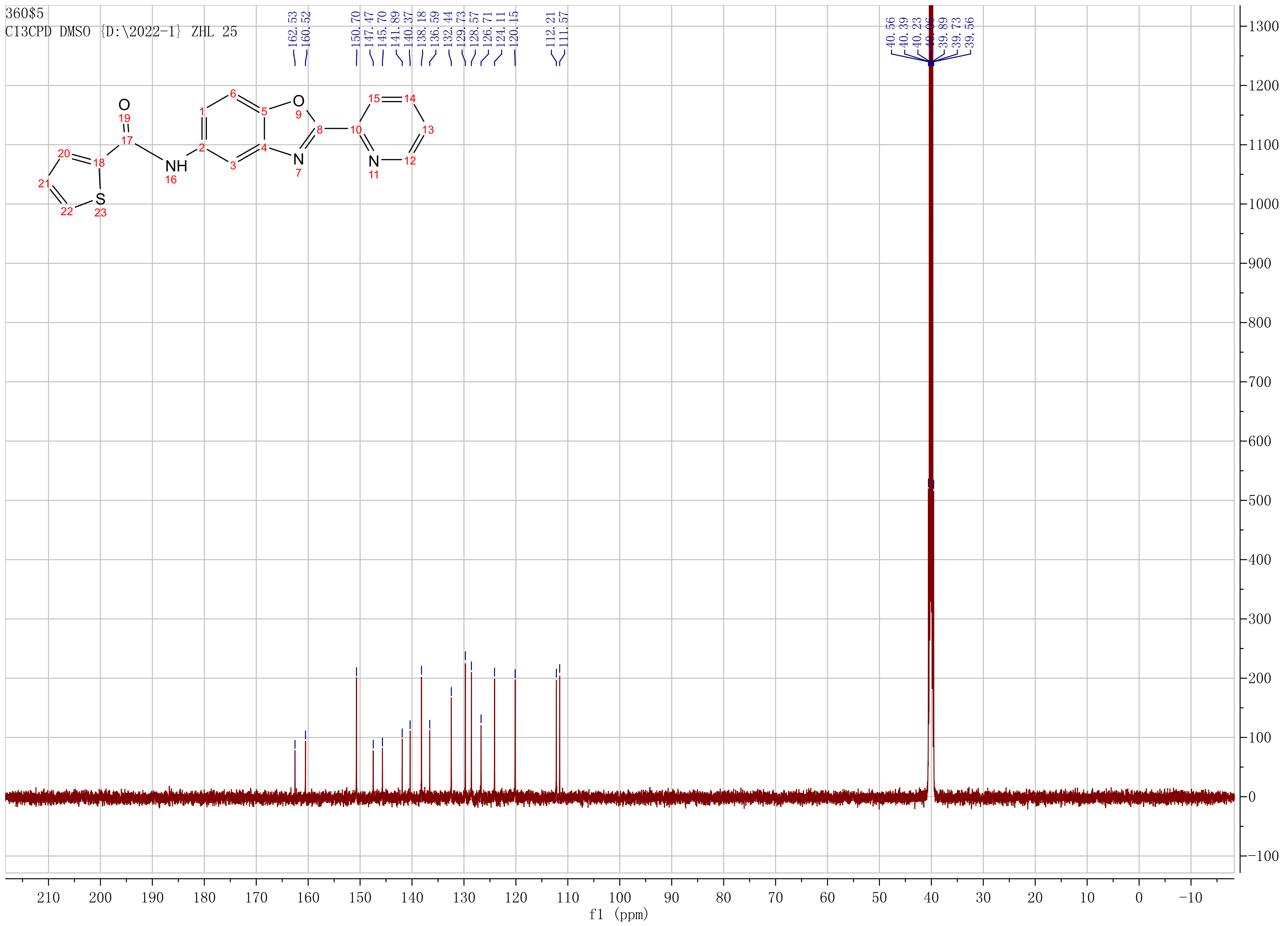
360$5
C13CPD DMSO {D:\2022-1} ZHL 25
162.53
160.52
150.70
147.47
145.70
141.89
140.37
138.18
136.59
132.44
129.73
128.57
126.71
124.11
120.15
112.21
111.57
40.56
40.39
40.23
39.89
39.73
39.56
f1 (ppm)

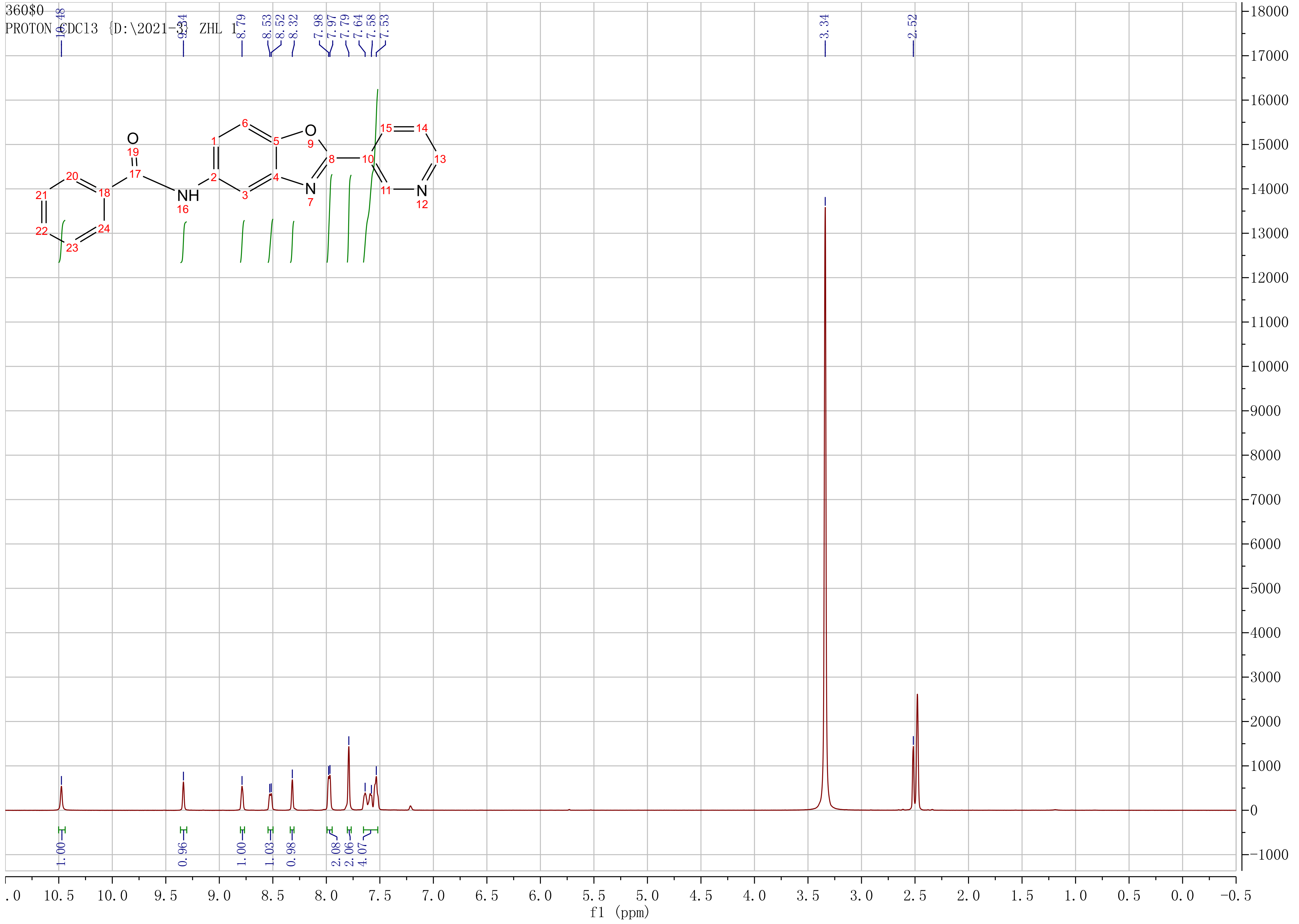
360$0
PROTON CDCl3 {D:\2021-3} ZHL 1
10.48
9.34
8.79
8.53
8.52
8.32
7.98
7.97
7.79
7.64
7.58
7.53
3.34
2.52
1.00
0.96
1.00
1.03
0.98
2.08
2.06
4.07
O
NH
N
f1 (ppm)

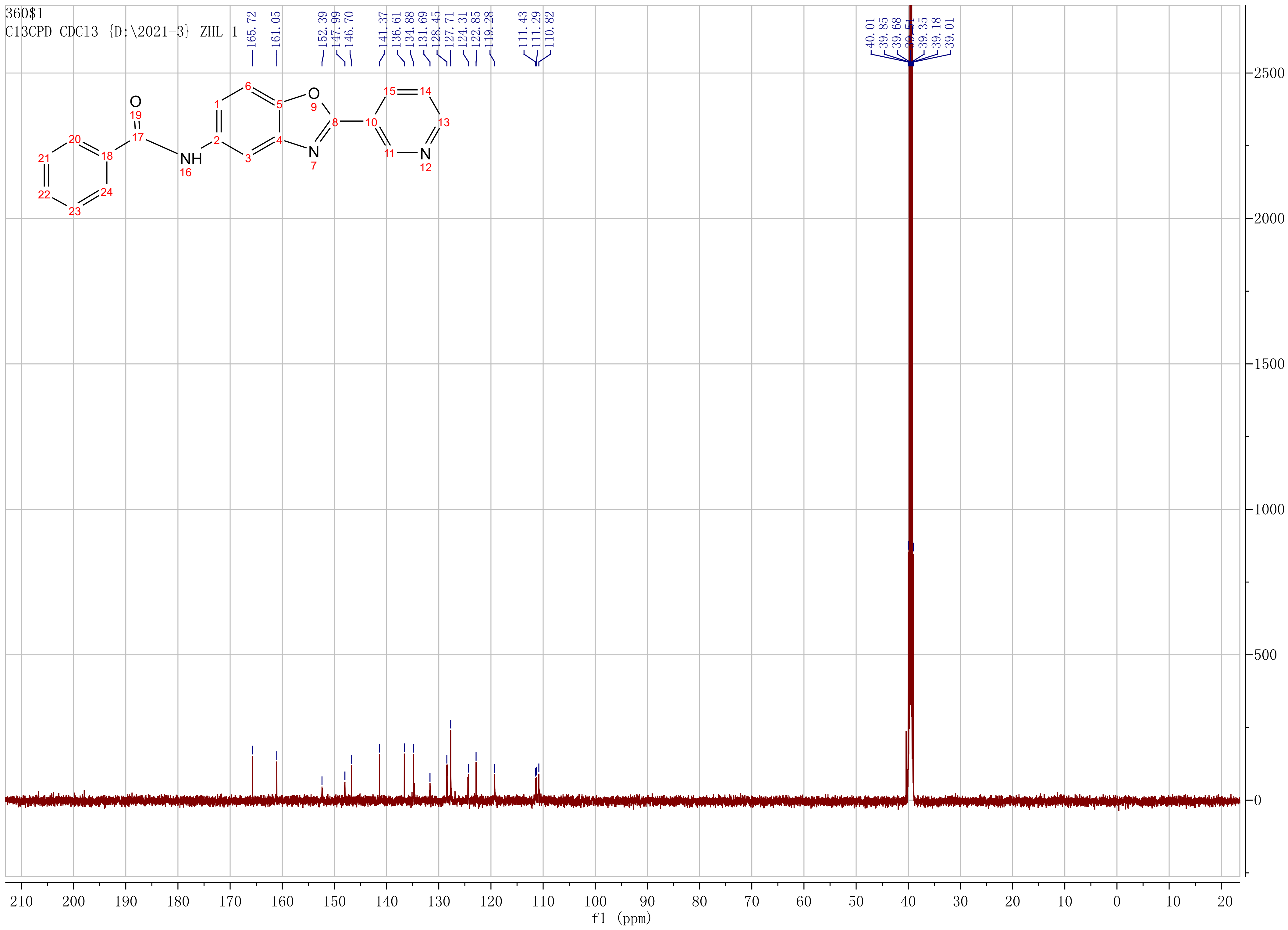

360$1
C13CPD CDCl3 {D:\2021-3} ZHL 1
165.72
161.05
152.39
147.99
146.70
141.37
136.61
134.88
131.69
128.45
127.71
124.31
122.85
119.28
111.43
111.29
110.82
40.01
39.85
39.68
39.51
39.35
39.18
39.01
f1 (ppm)

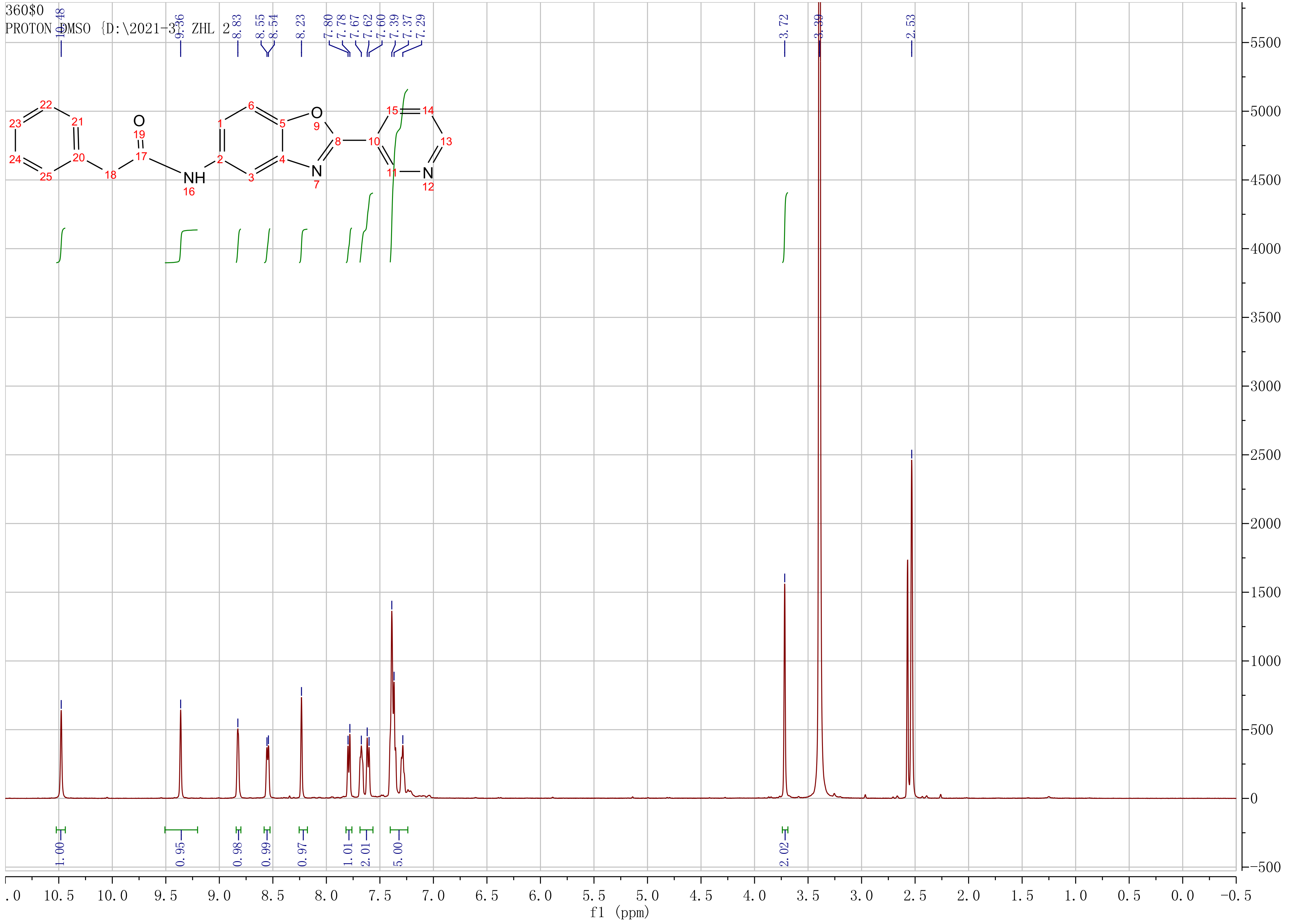
360$0
PROTON DMSO {D:\2021-3} ZHL 2
10.48
9.36
8.83
8.55
8.54
8.23
7.80
7.78
7.67
7.62
7.60
7.39
7.37
7.29
3.72
3.39
2.53
1.00
0.95
0.98
0.99
0.97
1.01
2.01
5.00
2.02
f1 (ppm)

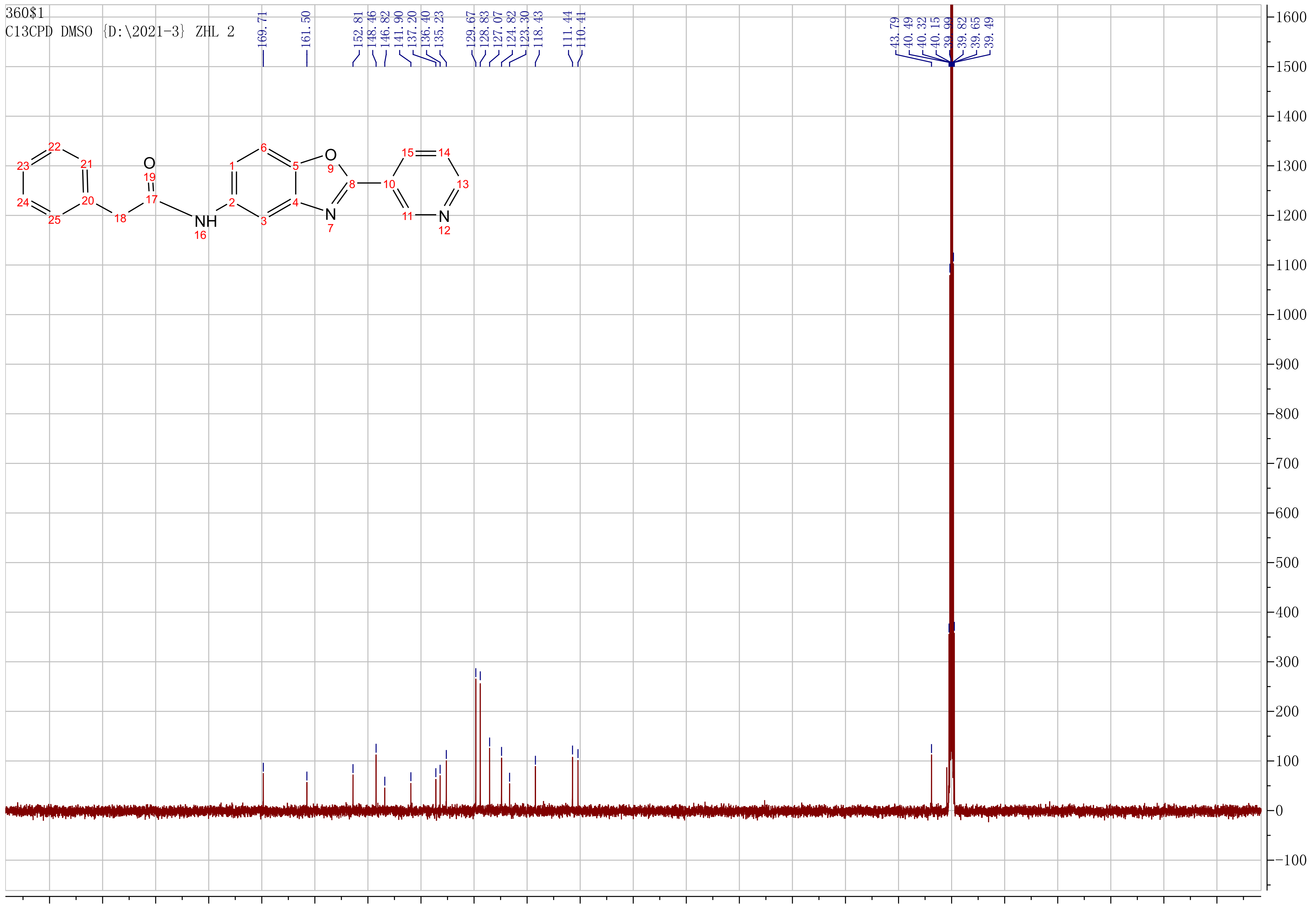

360$1
C13CPD DMSO {D:\2021-3} ZHL 2
169.71
161.50
152.81
148.46
146.82
141.90
137.20
136.40
135.23
129.67
128.83
127.07
124.82
123.30
118.43
111.44
110.41
43.79
40.49
40.32
40.15
39.99
39.82
39.65
39.49
O
NH
O
N
N

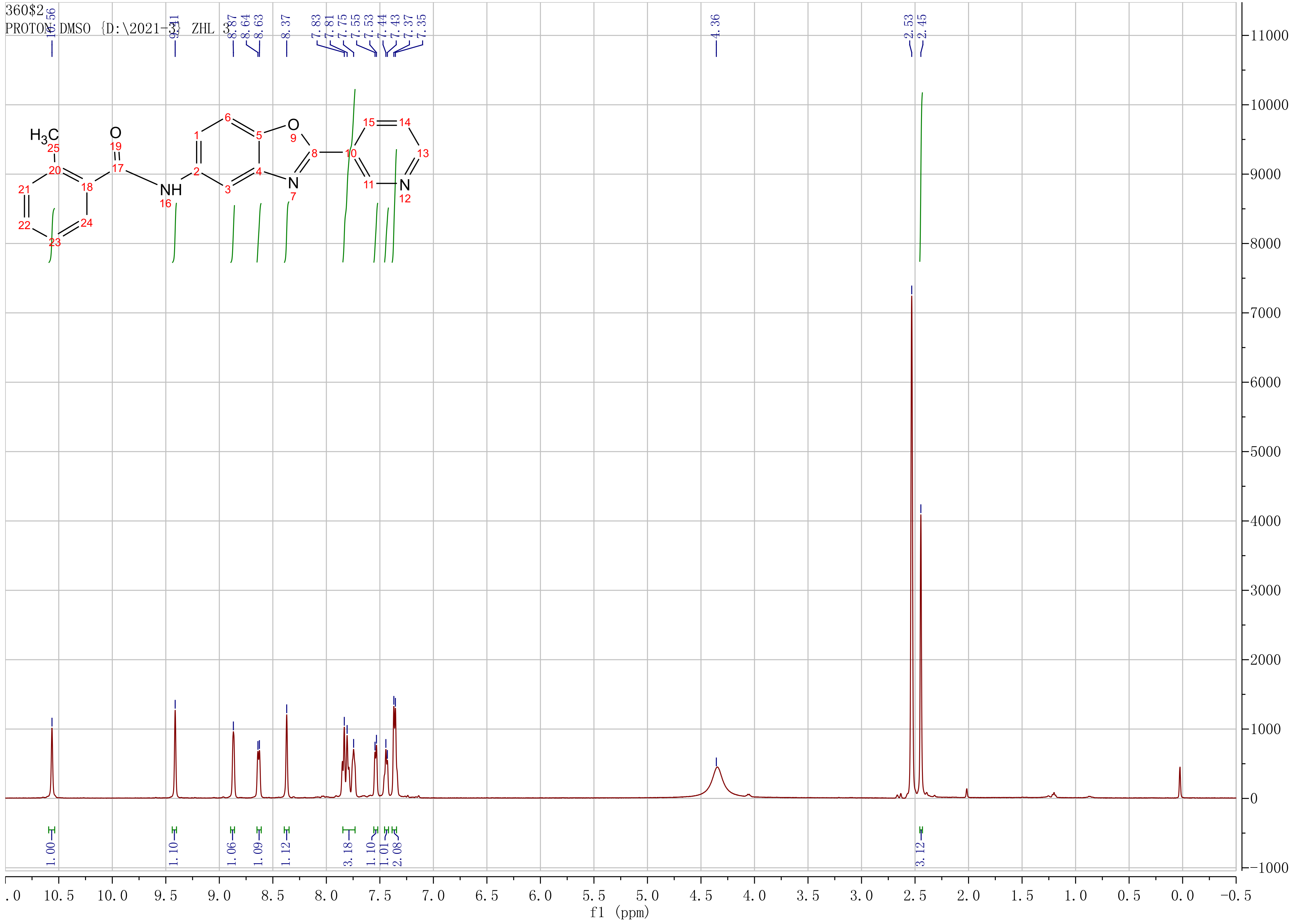

360$2
PROTON DMSO {D:\2021-3} ZHL 3
10.56
9.41
8.87
8.64
8.63
8.37
7.83
7.81
7.75
7.55
7.53
7.44
7.43
7.37
7.35
4.36
2.53
2.45
H3C
O
NH
O
N
N
1.00
1.10
1.06
1.09
1.12
3.18
1.10
1.01
2.08
3.12
f1 (ppm)

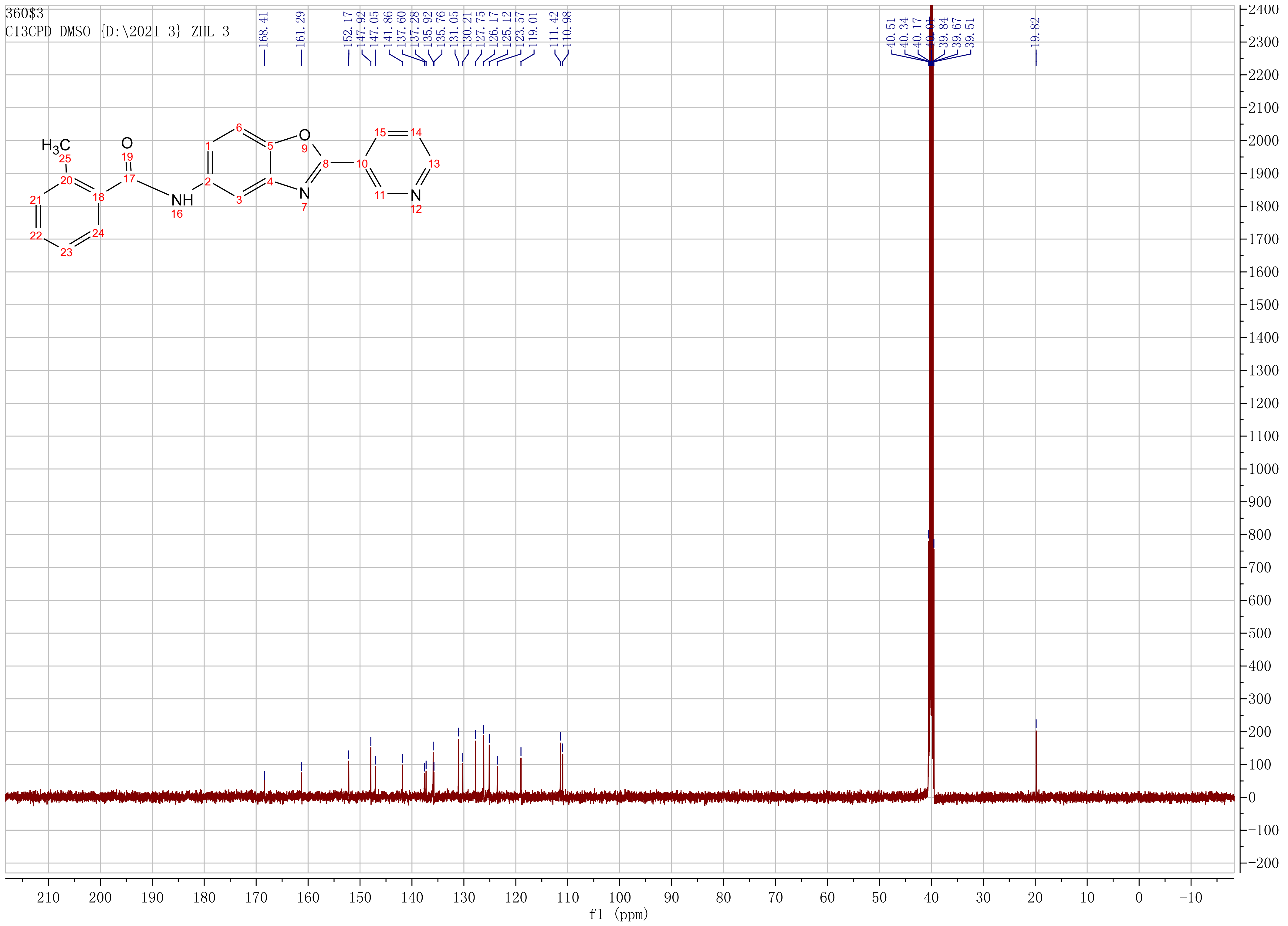

360$3
C13CPD DMSO {D:\2021-3} ZHL 3
168.41
161.29
152.17
147.92
147.05
141.86
137.60
137.28
135.92
135.76
131.05
130.21
127.75
126.17
125.12
123.57
119.01
111.42
110.98
40.51
40.34
40.17
40.01
39.84
39.67
39.51
19.82
f1 (ppm)

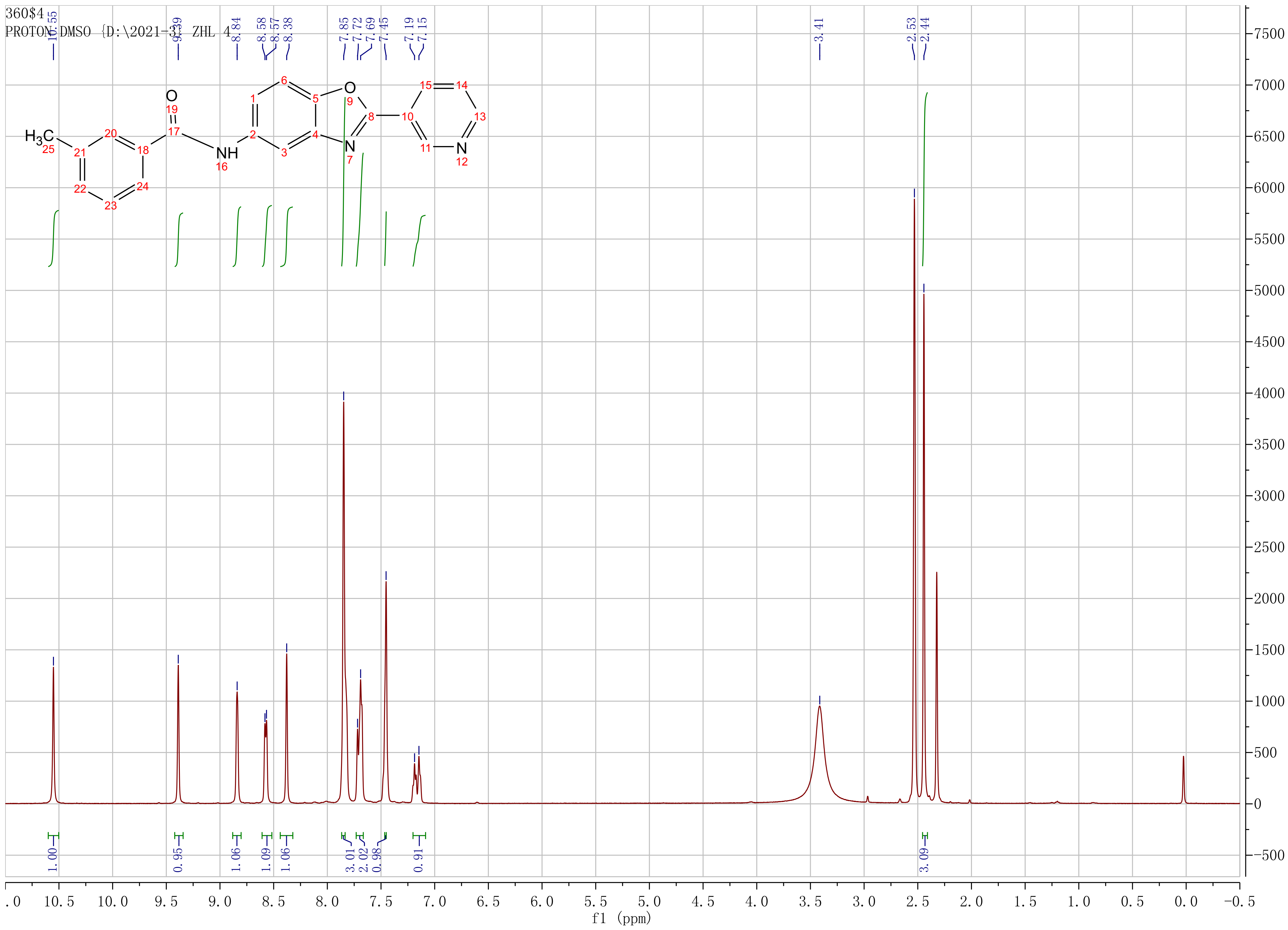
360$4
PROTON DMSO {D:\2021-3} ZHL 4
10.55
9.39
8.84
8.58
8.57
8.38
7.85
7.72
7.69
7.45
7.19
7.15
3.41
2.53
2.44
1.00
0.95
1.06
1.09
1.06
3.01
2.02
0.98
0.91
3.09
f1 (ppm)

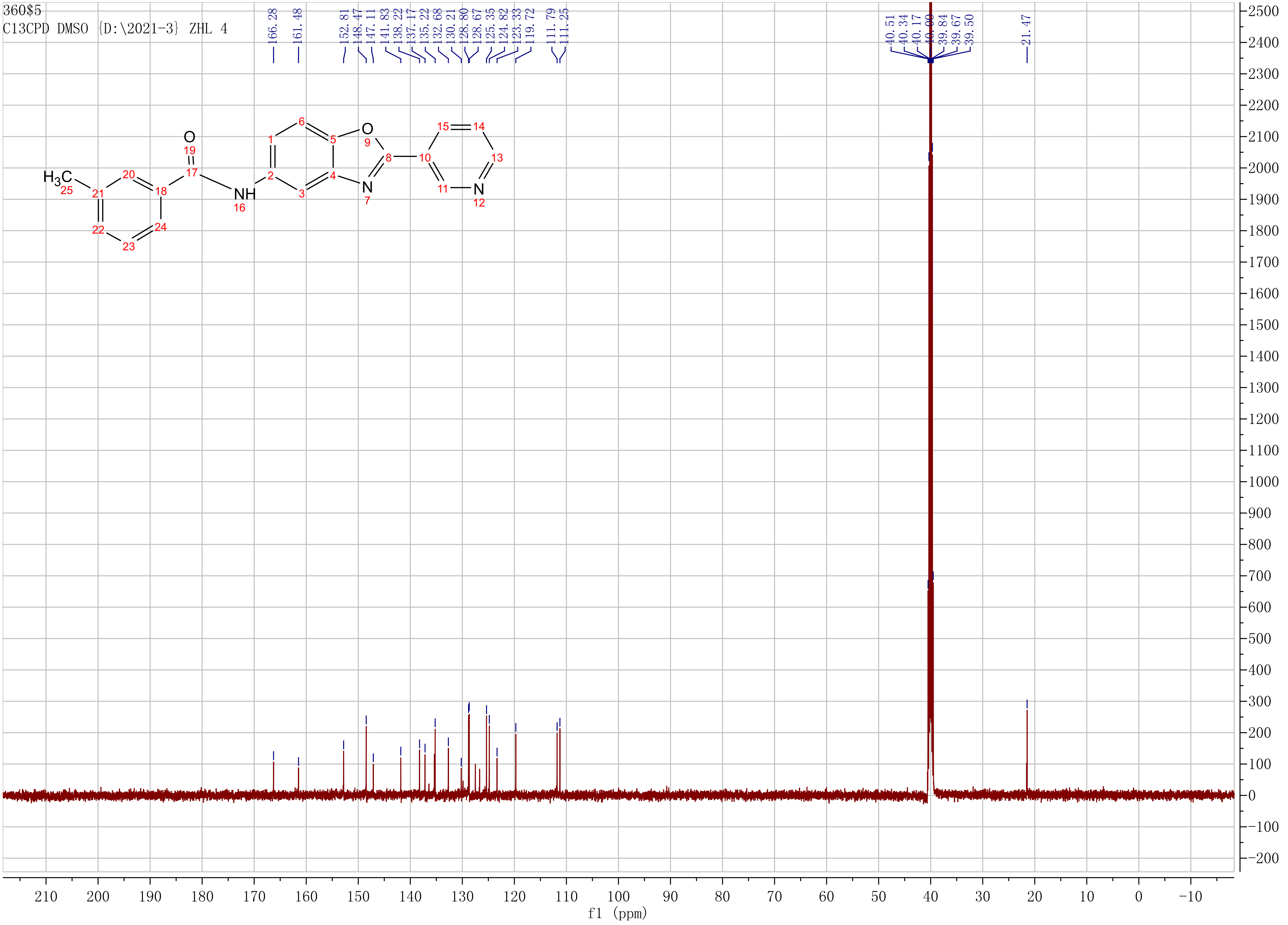

360$5
C13CPD DMSO {D:\2021-3} ZHL 4
166.28
161.48
152.81
148.47
147.11
141.83
138.22
137.17
135.22
132.68
130.21
128.80
128.67
125.35
124.82
123.33
119.72
111.79
111.25
40.51
40.34
40.17
40.00
39.84
39.67
39.50
21.47
f1 (ppm)

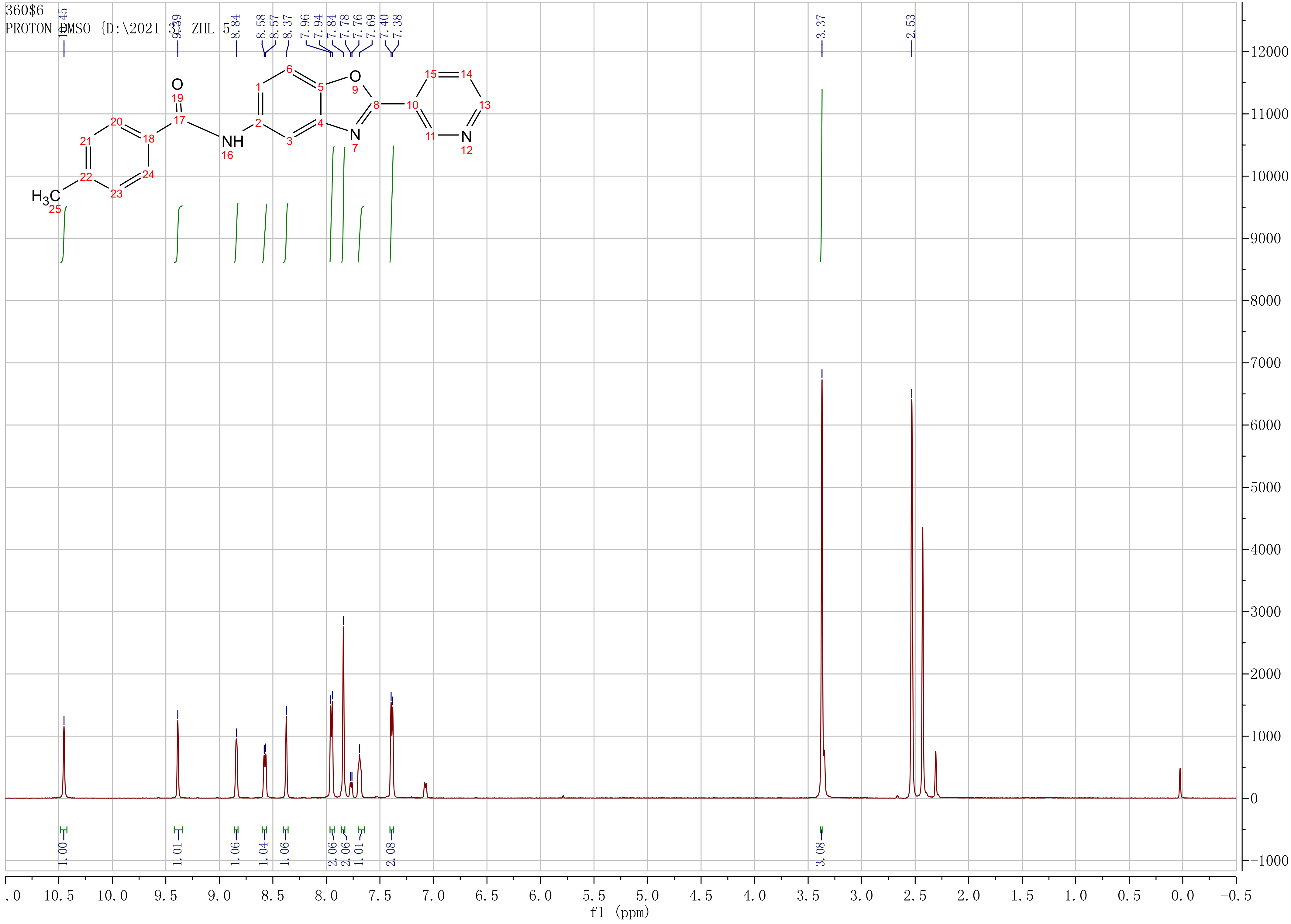

360$6
PROTON DMSO {D:\2021-3} ZHL 5
10.45
9.39
8.84
8.58
8.57
8.37
7.96
7.94
7.84
7.78
7.76
7.69
7.40
7.38
3.37
2.53
O
NH
N
N
H3C
1.00
1.01
1.06
1.04
1.06
2.06
2.06
1.01
2.08
3.08
f1 (ppm)

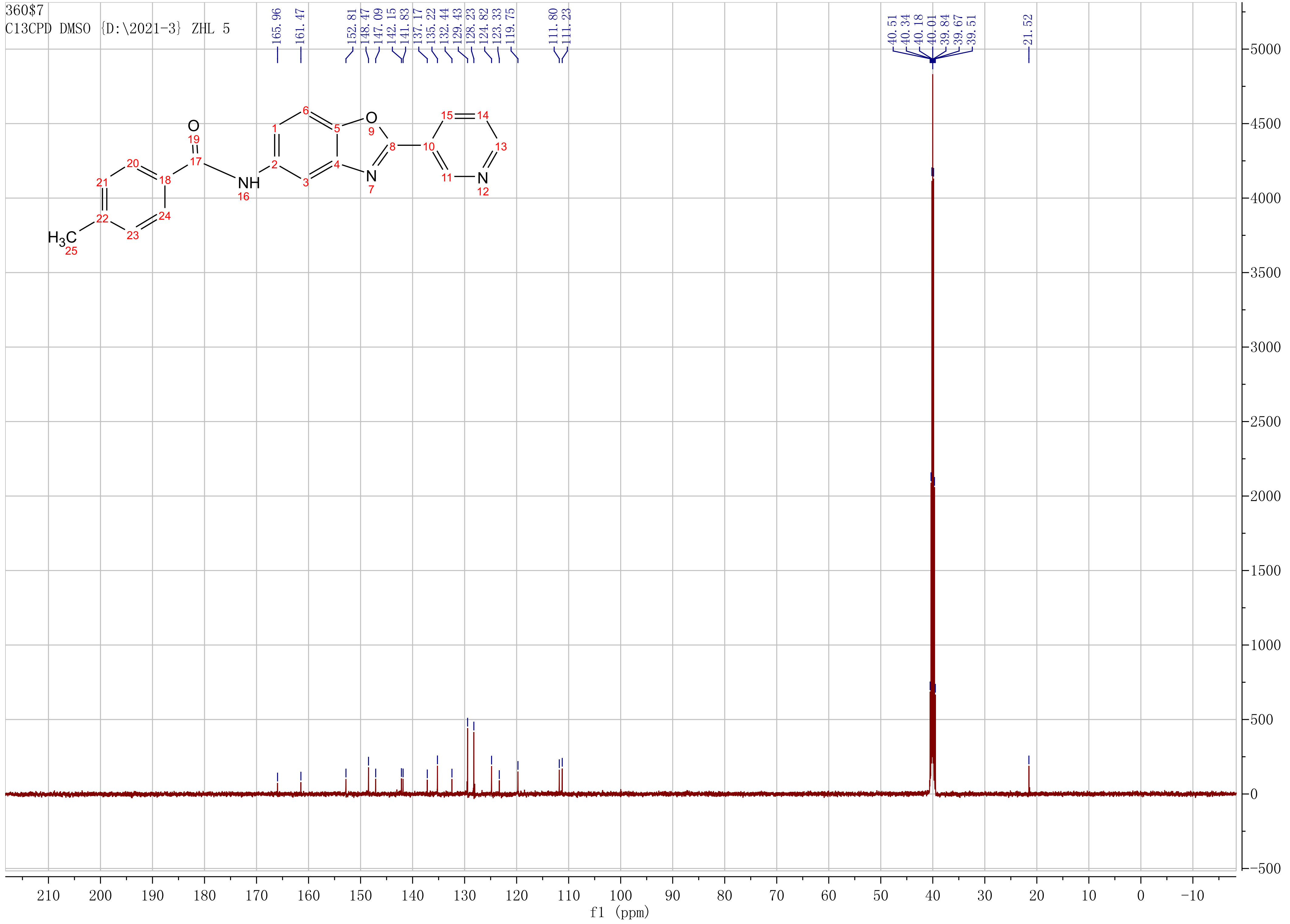

360$7
C13CPD DMSO {D:\2021-3} ZHL 5
165.96
161.47
152.81
148.47
147.09
142.15
141.83
137.17
135.22
132.44
129.43
128.23
124.82
123.33
119.75
111.80
111.23
40.51
40.34
40.18
40.01
39.84
39.67
39.51
21.52
f1 (ppm)

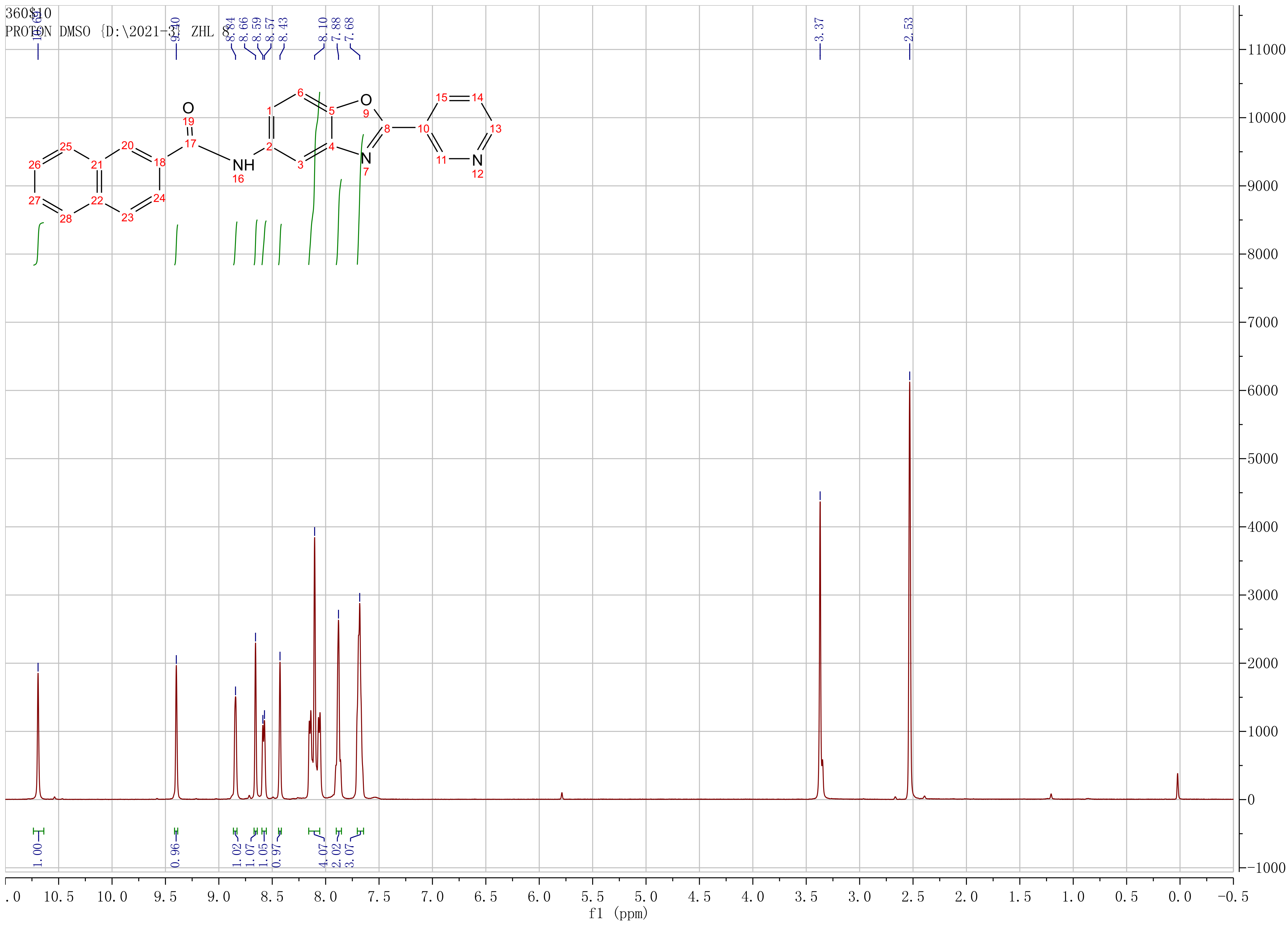
360$10
PROTON DMSO {D:\2021-3} ZHL 8
10.69
9.40
8.84
8.66
8.59
8.57
8.43
8.10
7.88
7.68
3.37
2.53
1.00
0.96
1.02
1.07
1.05
0.97
4.07
2.02
3.07
f1 (ppm)

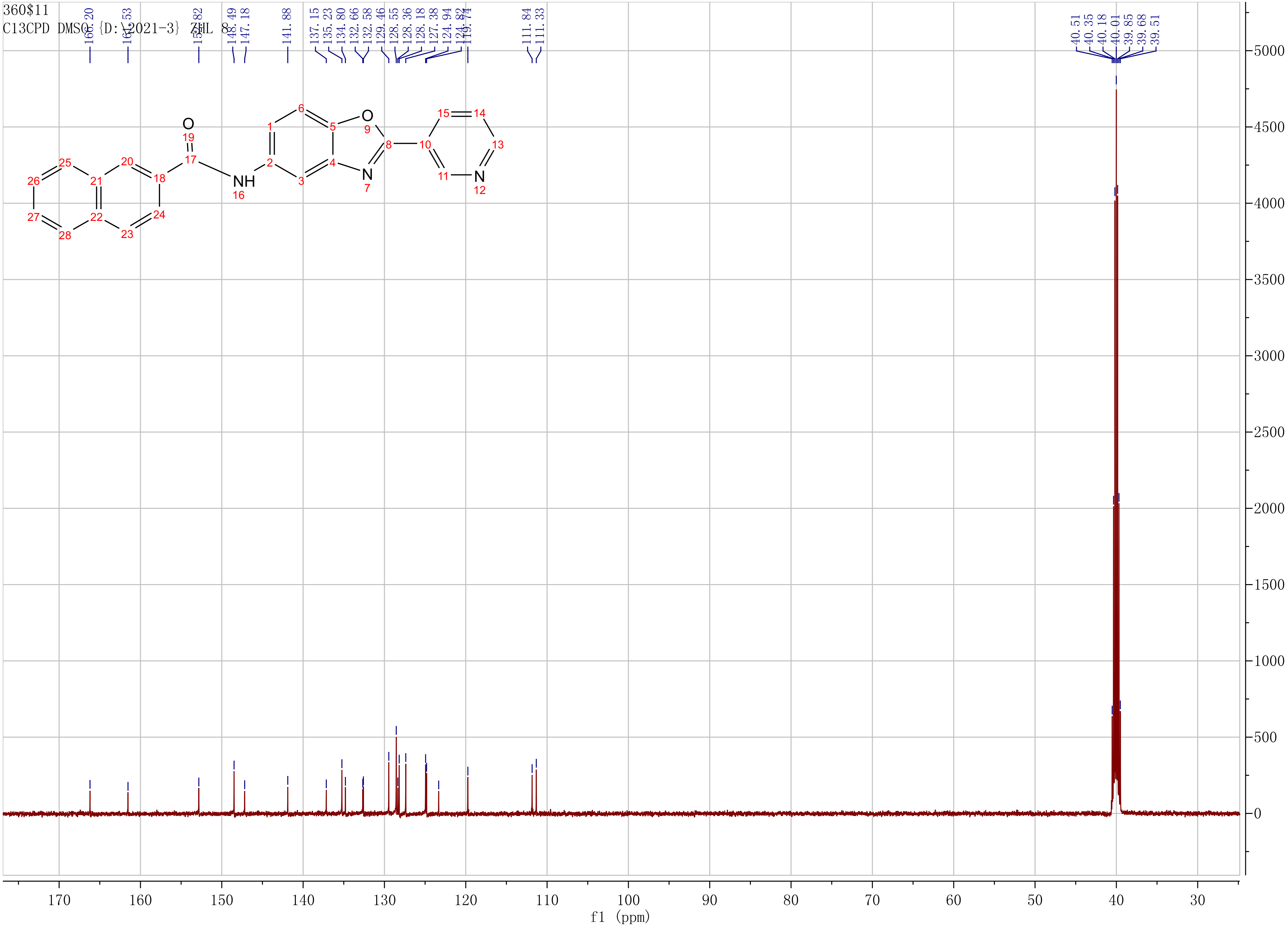
360$11
C13CPD DMSO {D:\2021-3} ZHL 8
166.20
161.53
152.82
148.49
147.18
141.88
137.15
135.23
134.80
132.66
132.58
129.46
128.55
128.36
128.18
127.38
124.94
124.82
119.74
111.84
111.33
40.51
40.35
40.18
40.01
39.85
39.68
39.51
f1 (ppm)

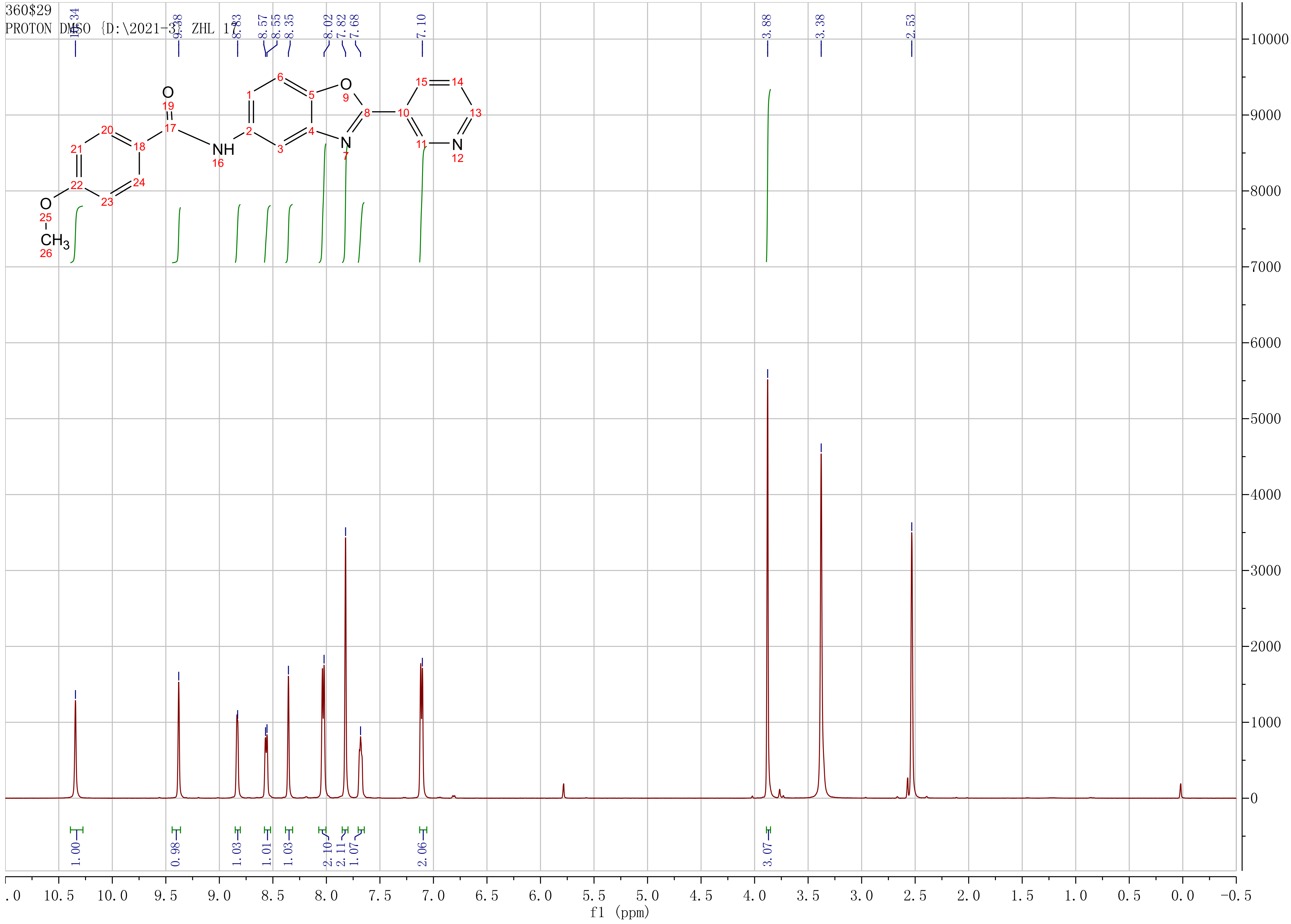
360$29
PROTON DMSO {D:\2021-3} ZHL 17}
10.34
9.38
8.83
8.57
8.55
8.35
8.02
7.82
7.68
7.10
3.88
3.38
2.53
1.00
0.98
1.03
1.01
1.03
2.10
2.11
1.07
2.06
3.07
f1 (ppm)

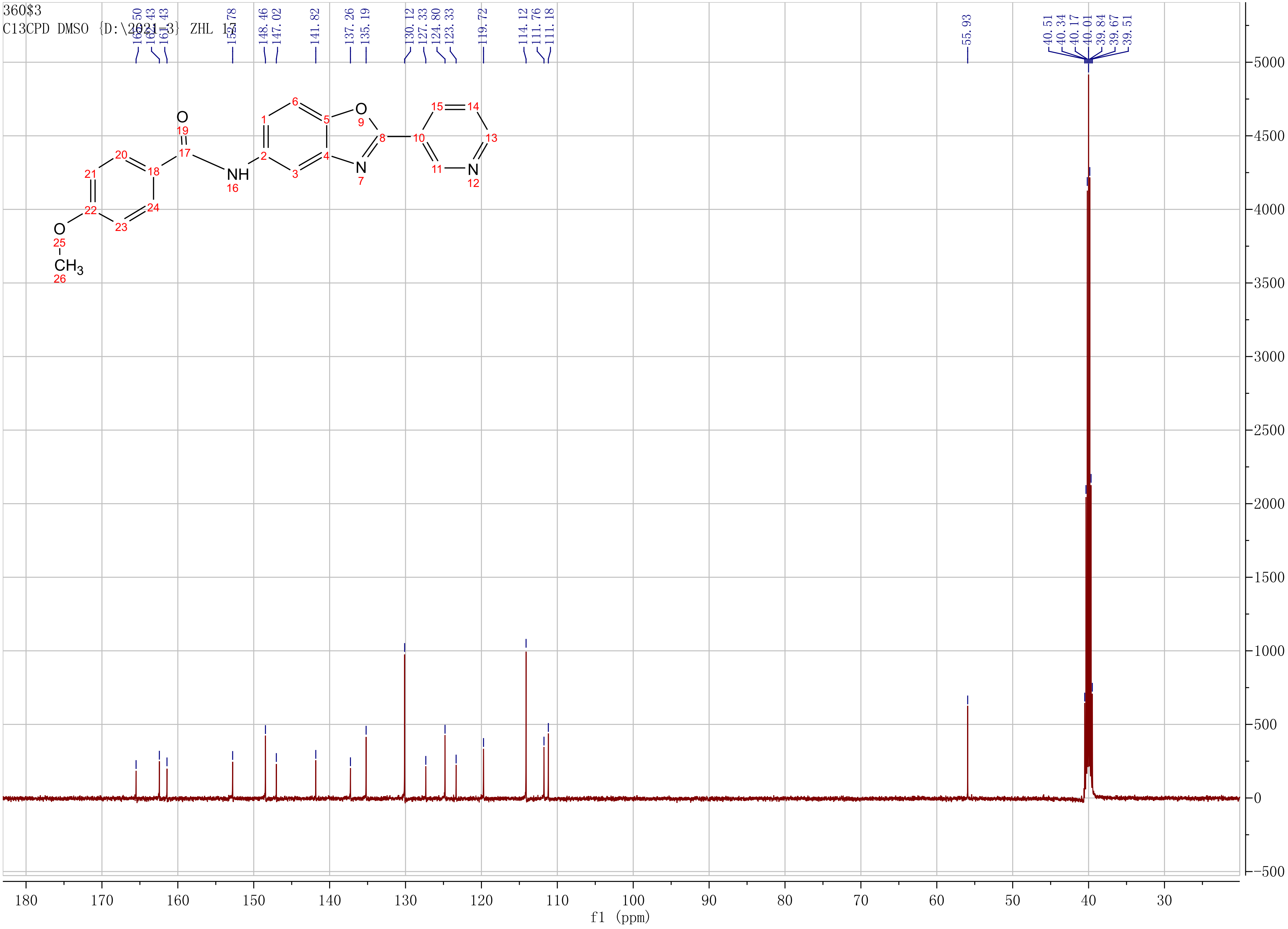

360$3
148.46
147.02
141.82
137.26
135.19
130.12
127.33
124.80
123.33
119.72
114.12
111.76
111.18
55.93
40.51
40.34
40.17
40.01
39.84
39.67
39.51
f1 (ppm)

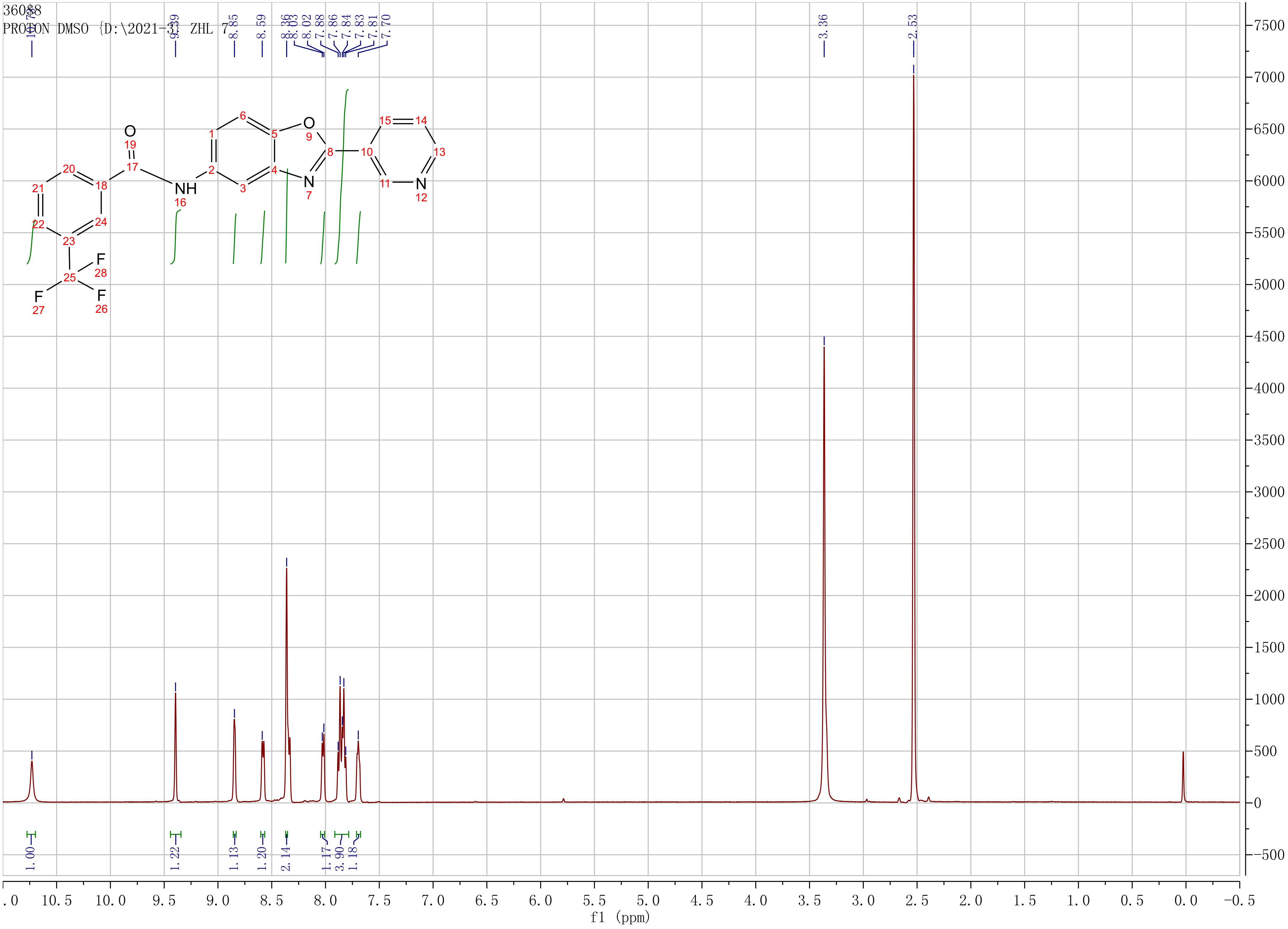

PROTON DMSO {D:\2021-3} ZHL 7
9.39
8.85
8.59
8.36
8.03
8.02
7.88
7.86
7.84
7.83
7.81
7.70
3.36
2.53
1.00
1.22
1.13
1.20
2.14
1.17
3.90
1.18
f1 (ppm)

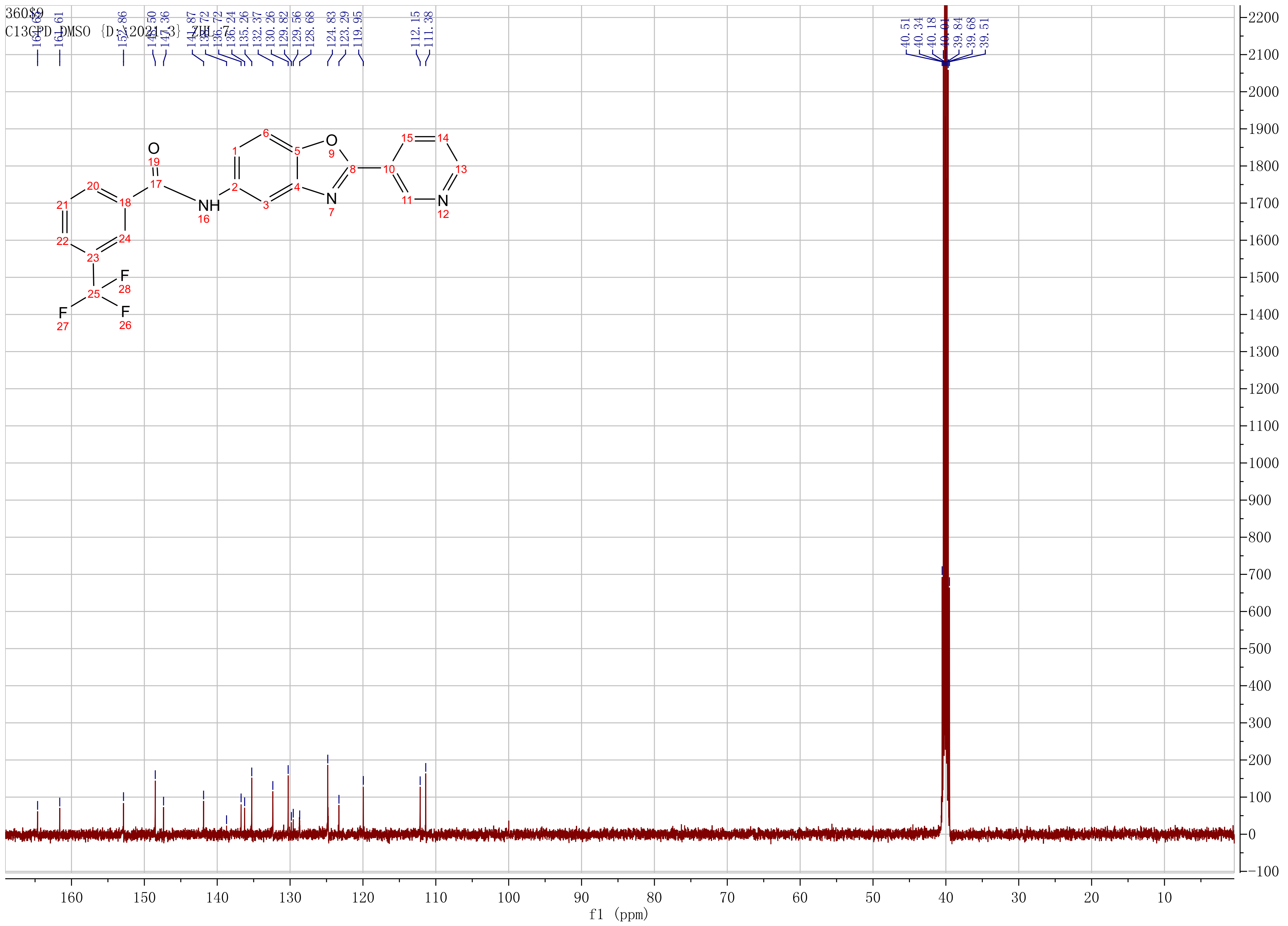
360$9
C13CPD DMSO {D:\2021-3} ZHL-7
161.61
152.86
148.50
147.36
141.87
138.72
136.72
136.24
135.26
132.37
130.26
129.82
129.56
128.68
124.83
123.29
119.95
112.15
111.38
40.51
40.34
40.18
39.84
39.68
39.51
f1 (ppm)

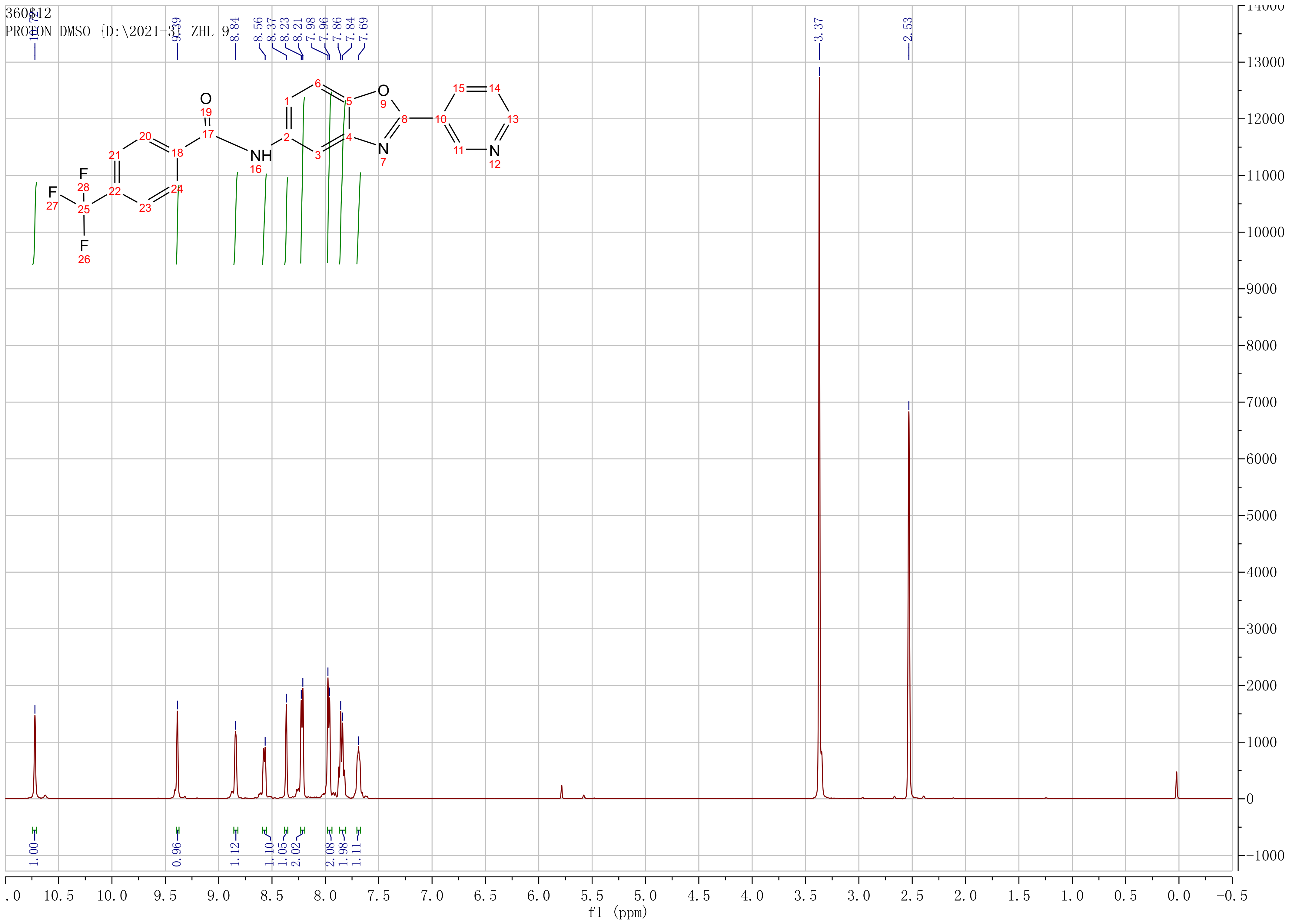
360812
PROTON DMSO {D:\2021-3} ZHL 9
10.72
9.39
8.84
8.56
8.37
8.23
8.21
7.98
7.96
7.86
7.84
7.69
3.37
2.53
1.00
0.96
1.12
1.10
1.05
2.02
2.08
1.98
1.11
f1 (ppm)

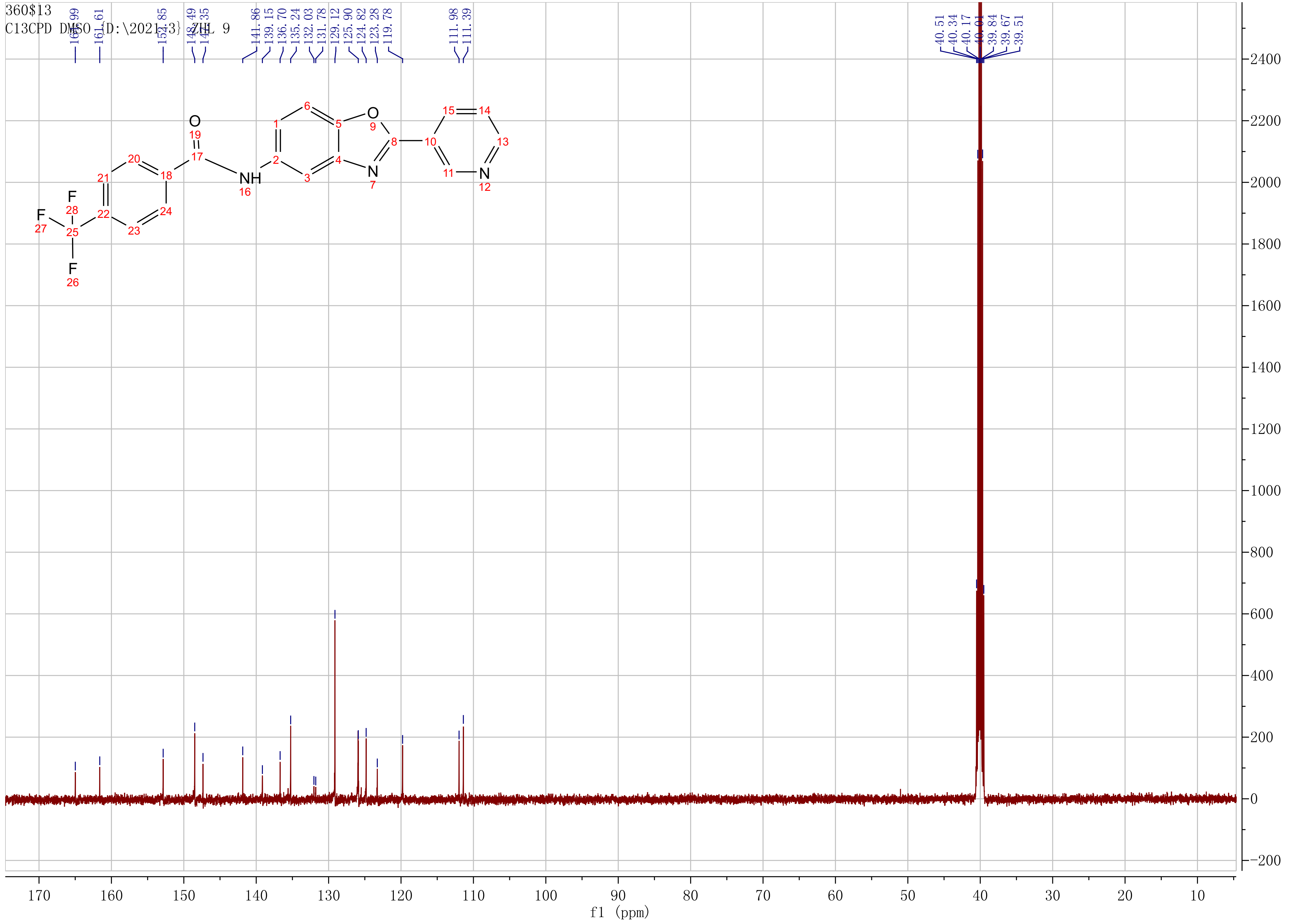

360$13
C13CPD DMSO {D:\2021-3} ZHL 9
164.99
161.61
152.85
148.49
147.35
141.86
139.15
136.70
135.24
132.03
131.78
129.12
125.90
124.82
123.28
119.78
111.98
111.39
40.51
40.34
40.17
40.01
39.84
39.67
39.51
f1 (ppm)

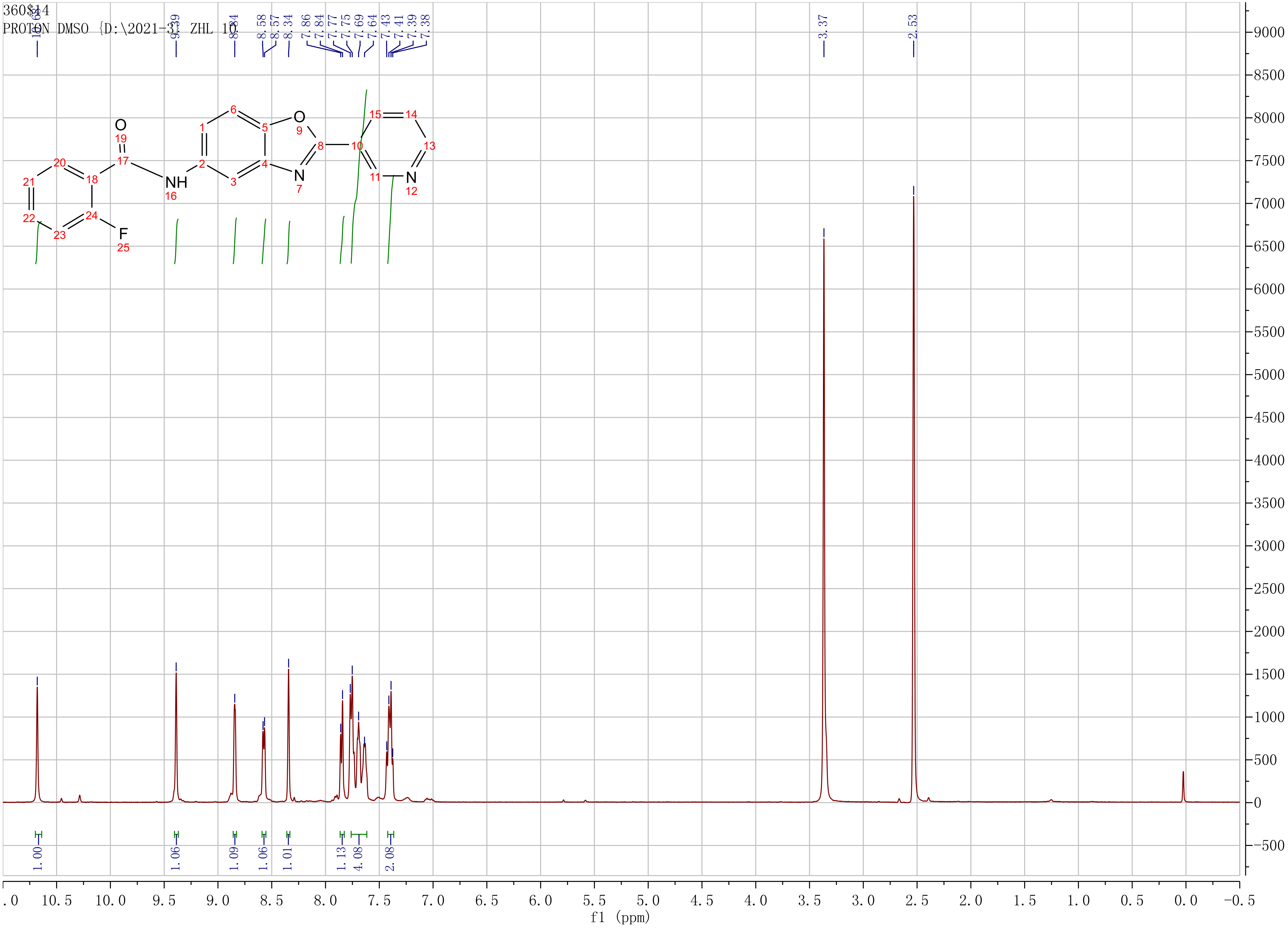

360$14
PROTON DMSO {D:\2021-3} ZHL 10
10.69
9.39
8.84
8.58
8.57
8.34
7.86
7.84
7.77
7.75
7.69
7.64
7.43
7.41
7.39
7.38
3.37
2.53
1.00
1.06
1.09
1.06
1.01
1.13
4.08
2.08
O
19
17
20
21
18
22
23
24
F
25
NH
16
1
2
3
4
5
6
O
9
8
N
7
10
11
N
12
13
14
15
f1 (ppm)

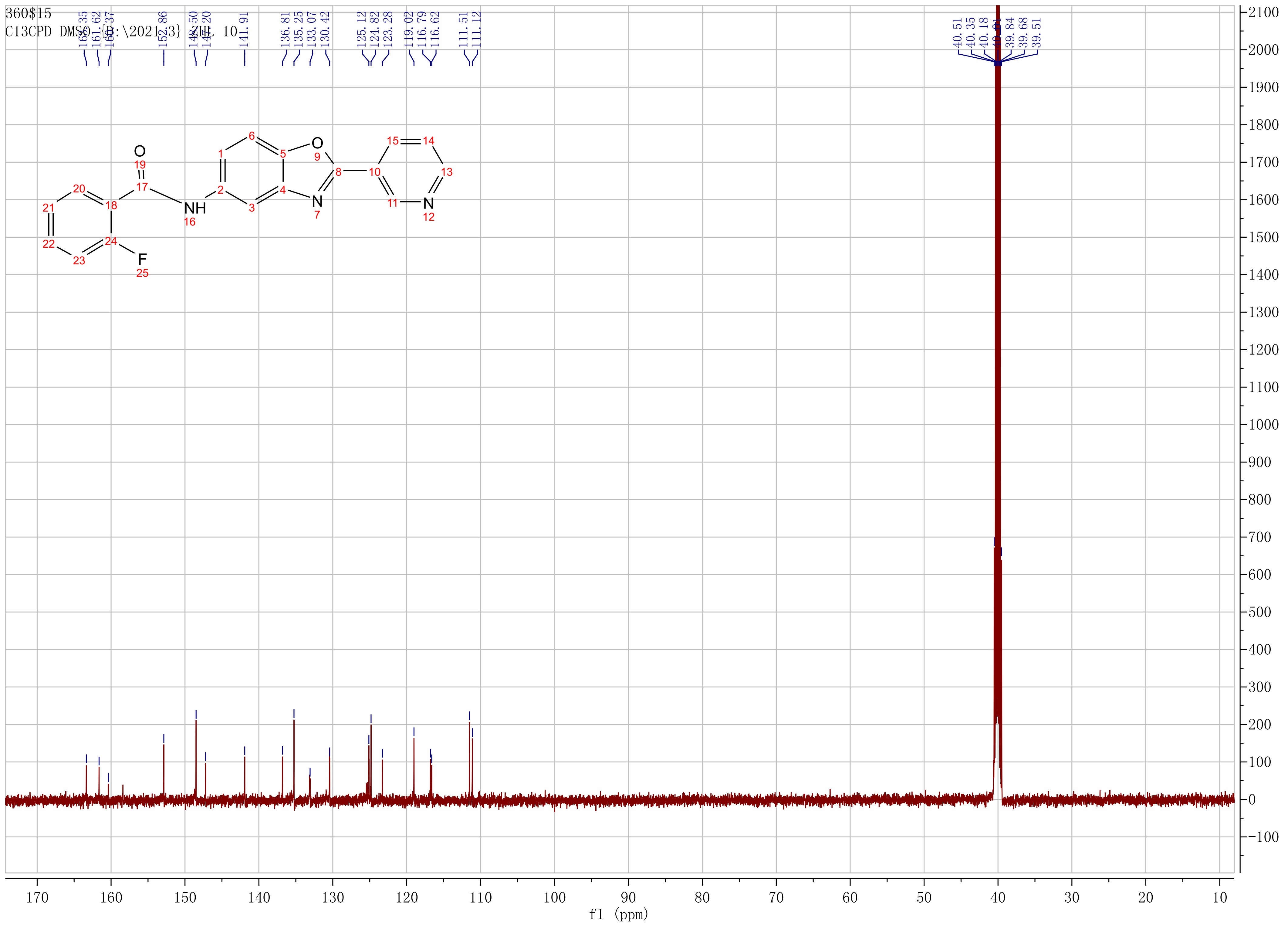

360$15
161.62
141.91
136.81
135.25
133.07
130.42
125.12
124.82
123.28
119.02
116.79
116.62
111.51
111.12
40.51
40.35
40.18
39.84
39.68
39.51
f1 (ppm)

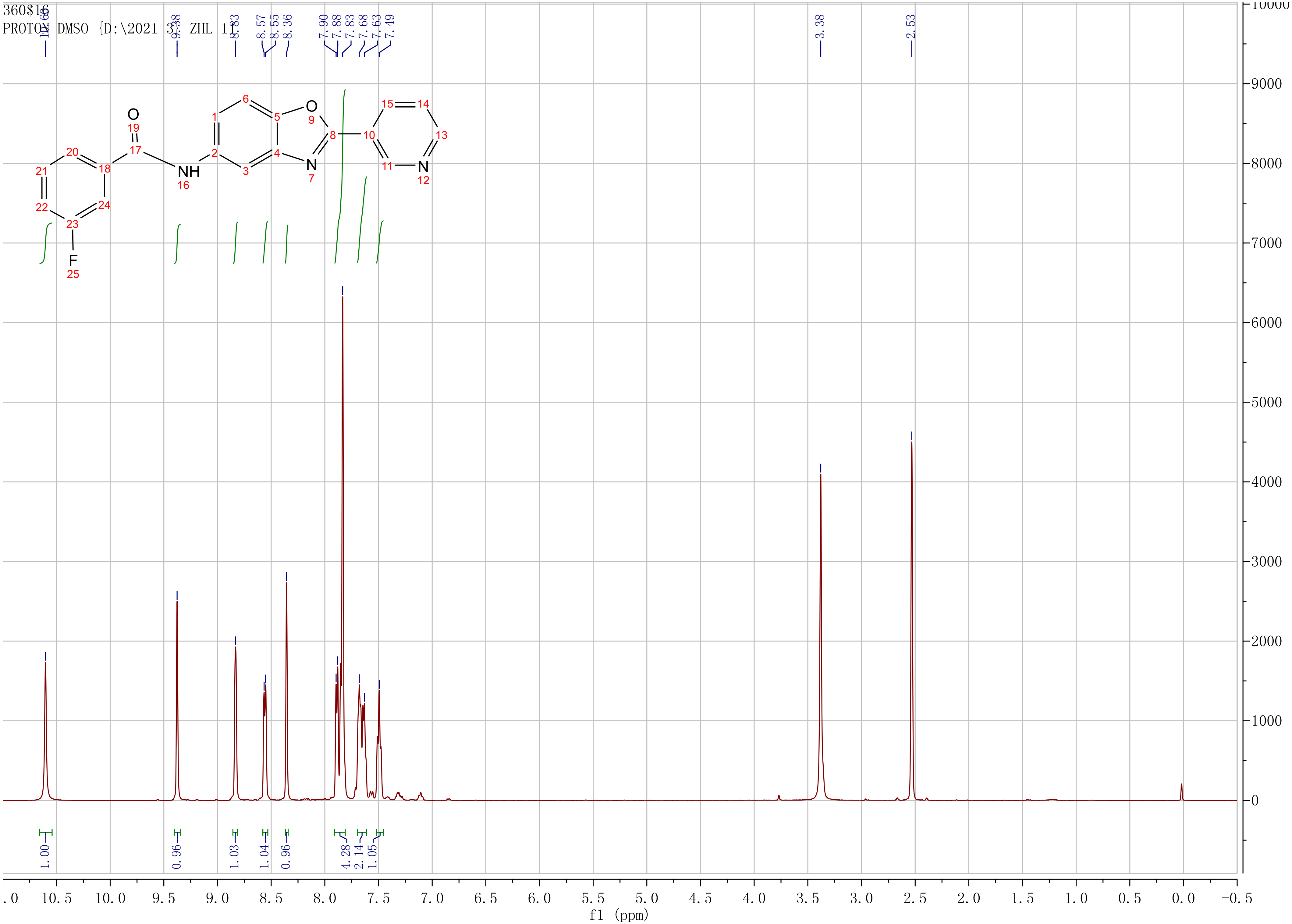

360$16
PROTON DMSO {D:\2021-3 ZHL 1
10.60
9.38
8.83
8.57
8.55
8.36
7.90
7.88
7.83
7.68
7.63
7.49
3.38
2.53
1.00
0.96
1.03
1.04
0.96
4.28
2.14
1.05
f1 (ppm)

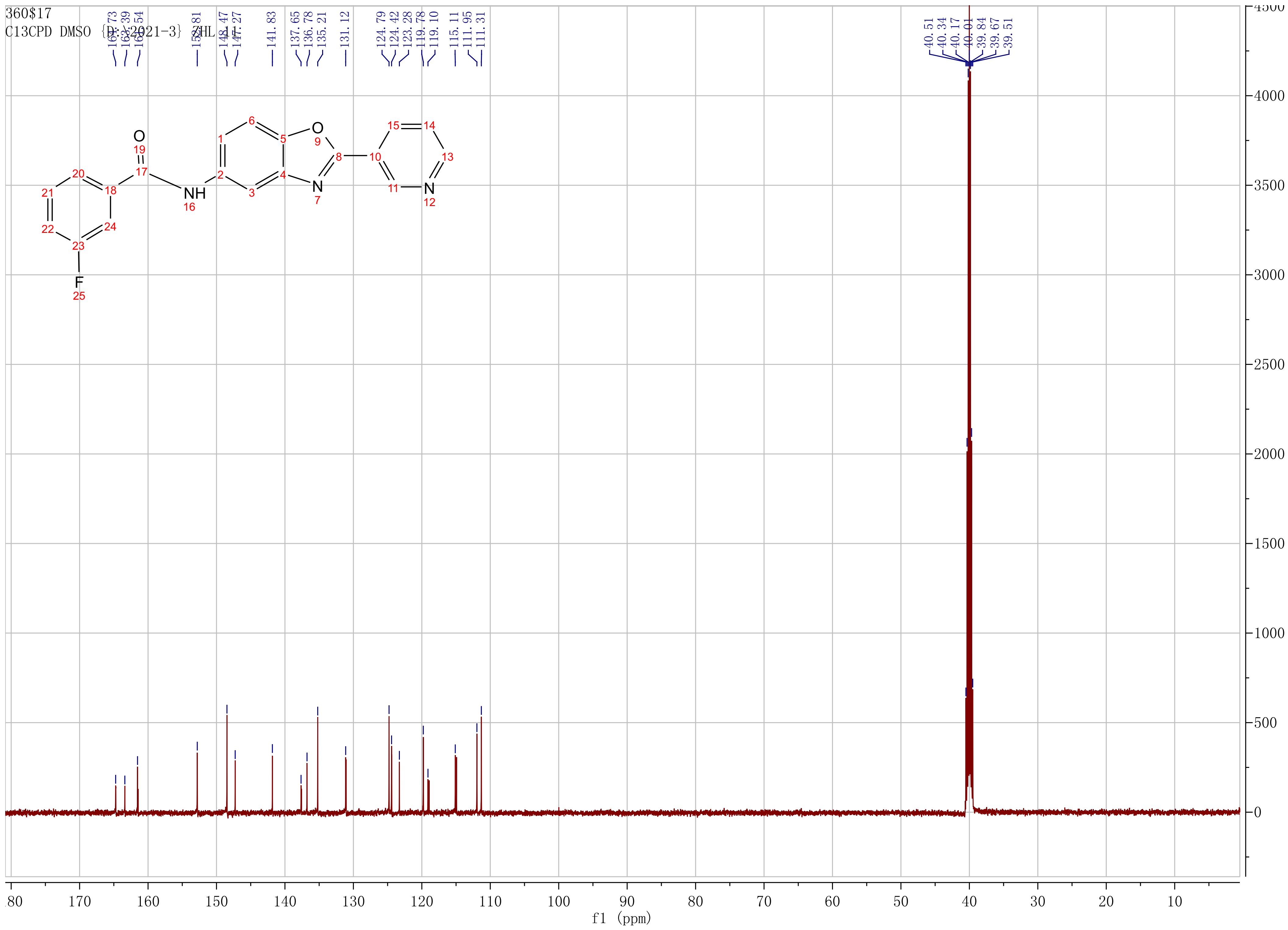

360$17
C13CPD DMSO {D:\2021-3} ZHL 11
153.81
148.47
147.27
141.83
137.65
136.78
135.21
131.12
124.79
124.42
123.28
119.78
119.10
115.11
111.95
111.31
40.51
40.34
40.17
40.01
39.84
39.67
39.51
NH
O
N
F
f1 (ppm)

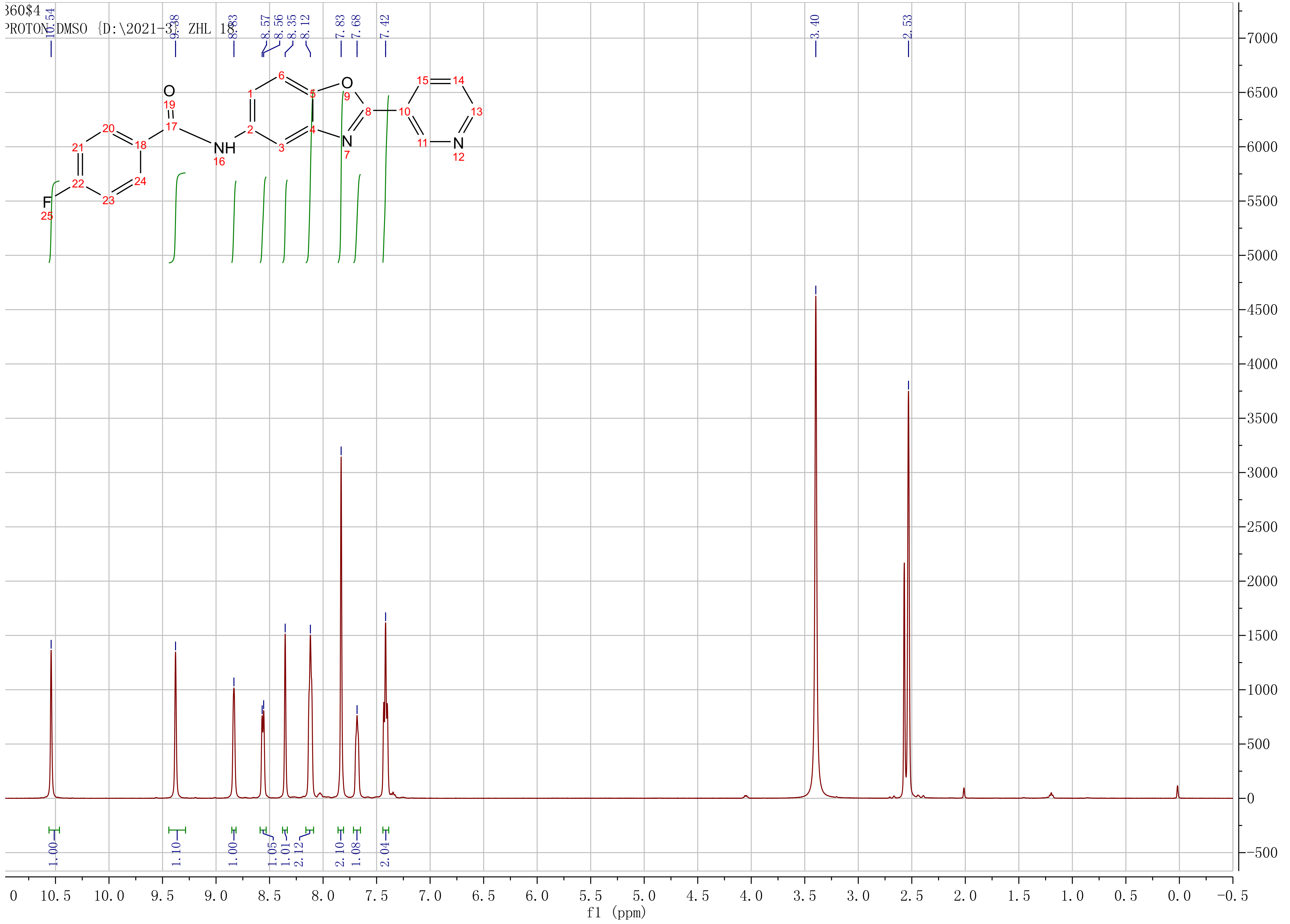

360$4
PROTON DMSO {D:\2021-3} ZHL 18
10.54
9.38
8.83
8.57
8.56
8.35
8.12
7.83
7.68
7.42
3.40
2.53
1.00
1.10
1.00
1.05
1.01
2.12
2.10
1.08
2.04
f1 (ppm)

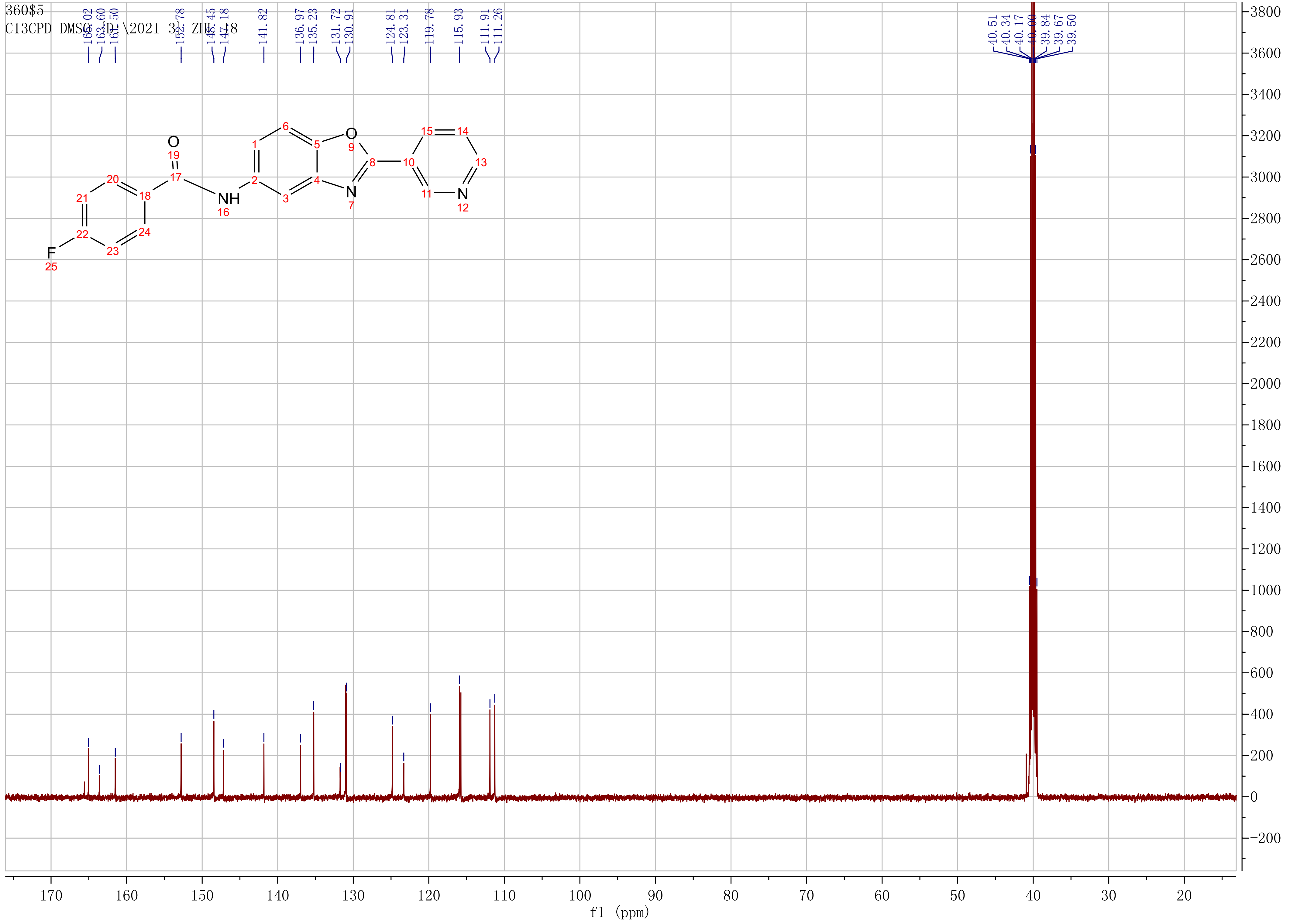

360$5
C13CPD DMSO
163.60
141.82
136.97
135.23
131.72
130.91
124.81
123.31
119.78
115.93
111.91
111.26
40.51
40.34
40.17
40.00
39.84
39.67
39.50
f1 (ppm)

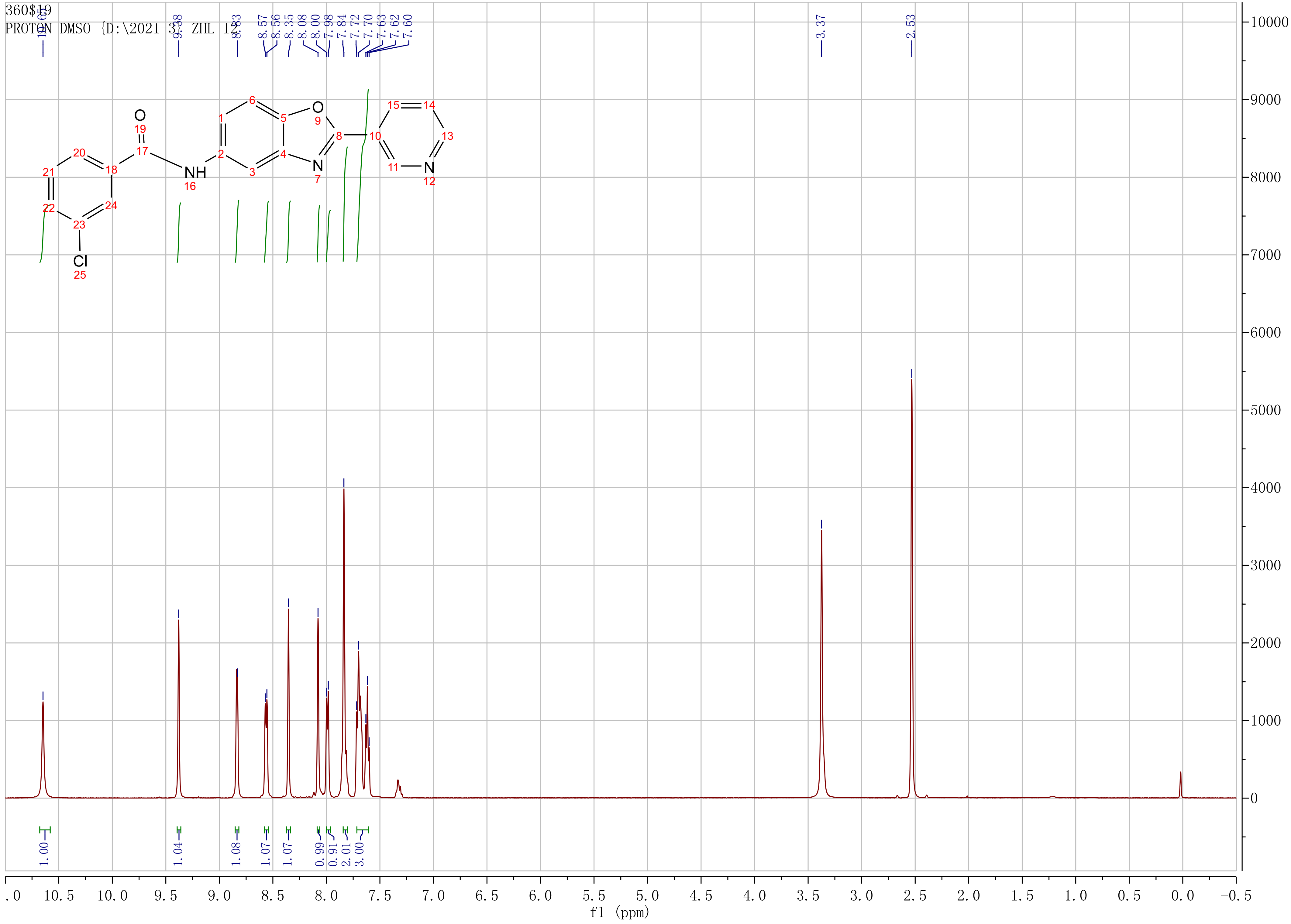

360$19
PROTON DMSO {D:\2021-3\ ZHL 1
9.38
8.83
8.57
8.56
8.35
8.08
8.00
7.98
7.84
7.72
7.70
7.63
7.62
7.60
3.37
2.53
O
19
17
NH
16
18
20
21
22
23
24
Cl
25
1
2
3
4
5
6
O
9
8
N
7
10
11
N
12
13
14
15
1.00
1.04
1.08
1.07
1.07
0.99
0.91
2.01
3.00
f1 (ppm)

360$20
C13CPD DMSO {D:\2021-3} ZHL 12

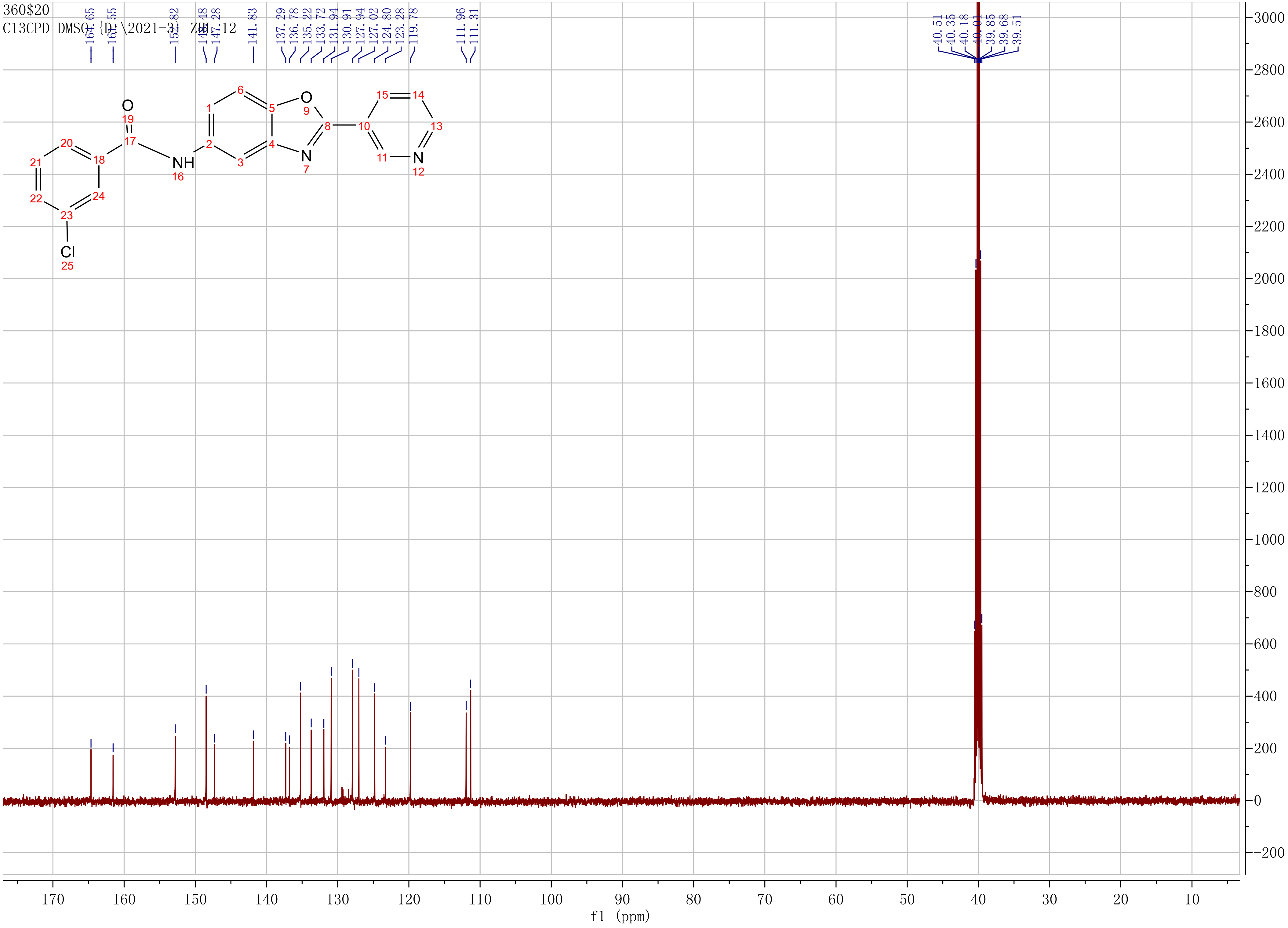

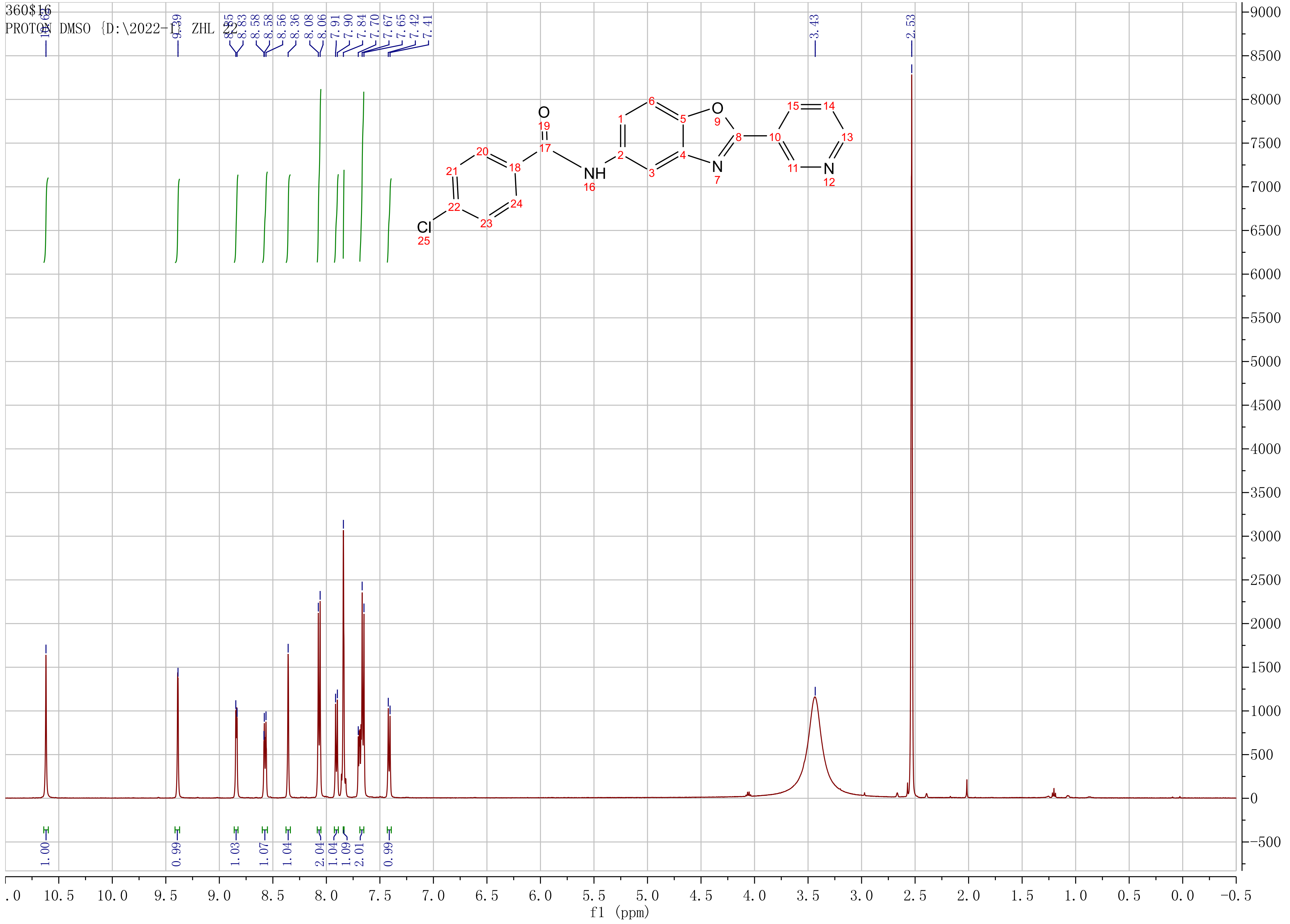
360$16
PROTON DMSO {D:\2022-1
9.39
8.85
8.83
8.58
8.58
8.56
8.36
8.08
8.06
7.91
7.90
7.84
7.70
7.67
7.65
7.42
7.41
3.43
2.53
1.00
0.99
1.03
1.07
1.04
2.04
1.04
1.09
2.01
0.99
f1 (ppm)

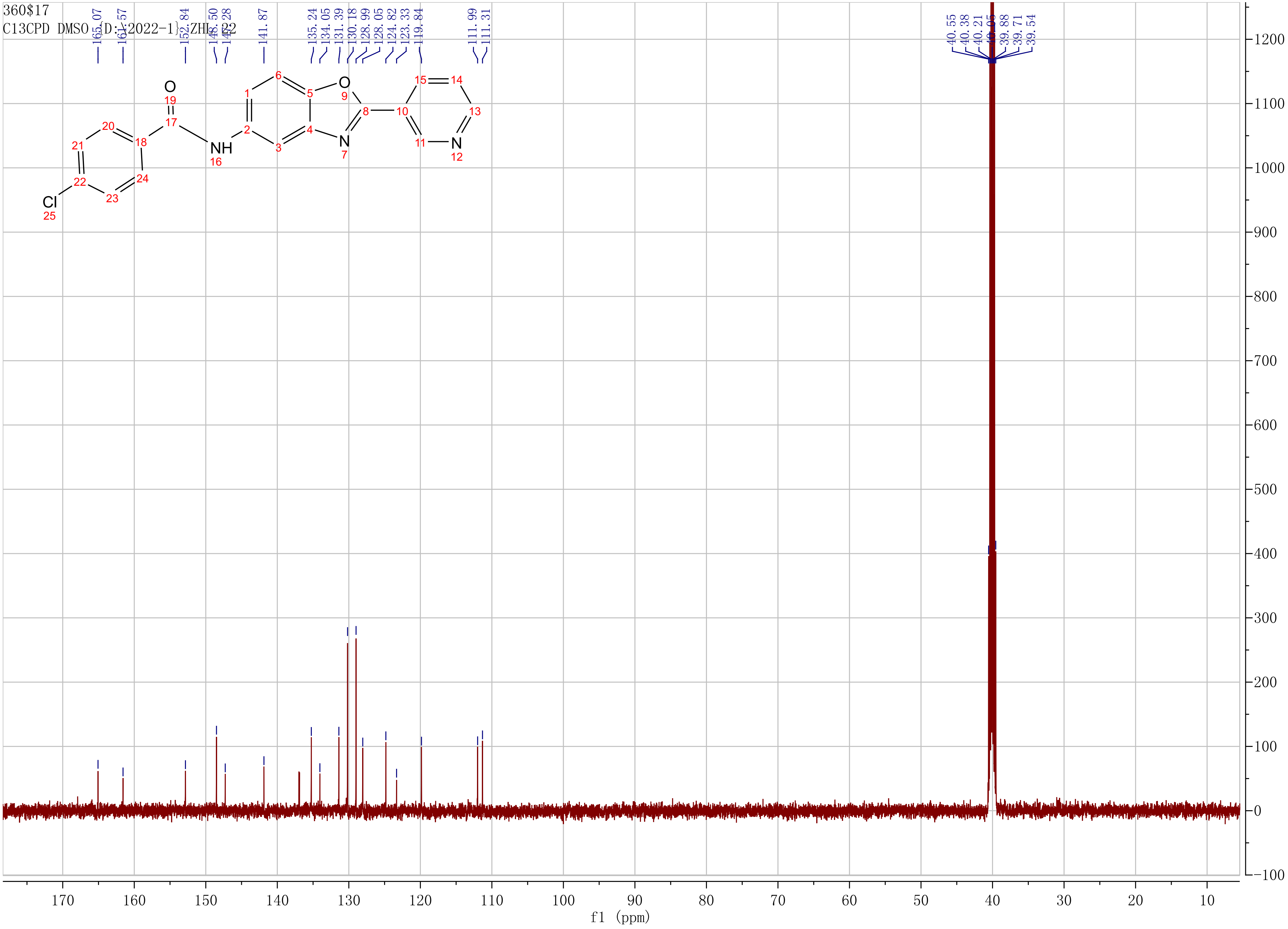
360$17
C13CPD DMSO {D:\2022-1} ZHL 22
165.07
161.57
152.84
148.50
147.28
141.87
135.24
134.05
131.39
130.18
128.99
128.05
124.82
123.33
119.84
111.99
111.31
40.55
40.38
40.21
39.88
39.71
39.54
O
19
17
NH
16
Cl
25
O
9
N
7
N
12
f1 (ppm)

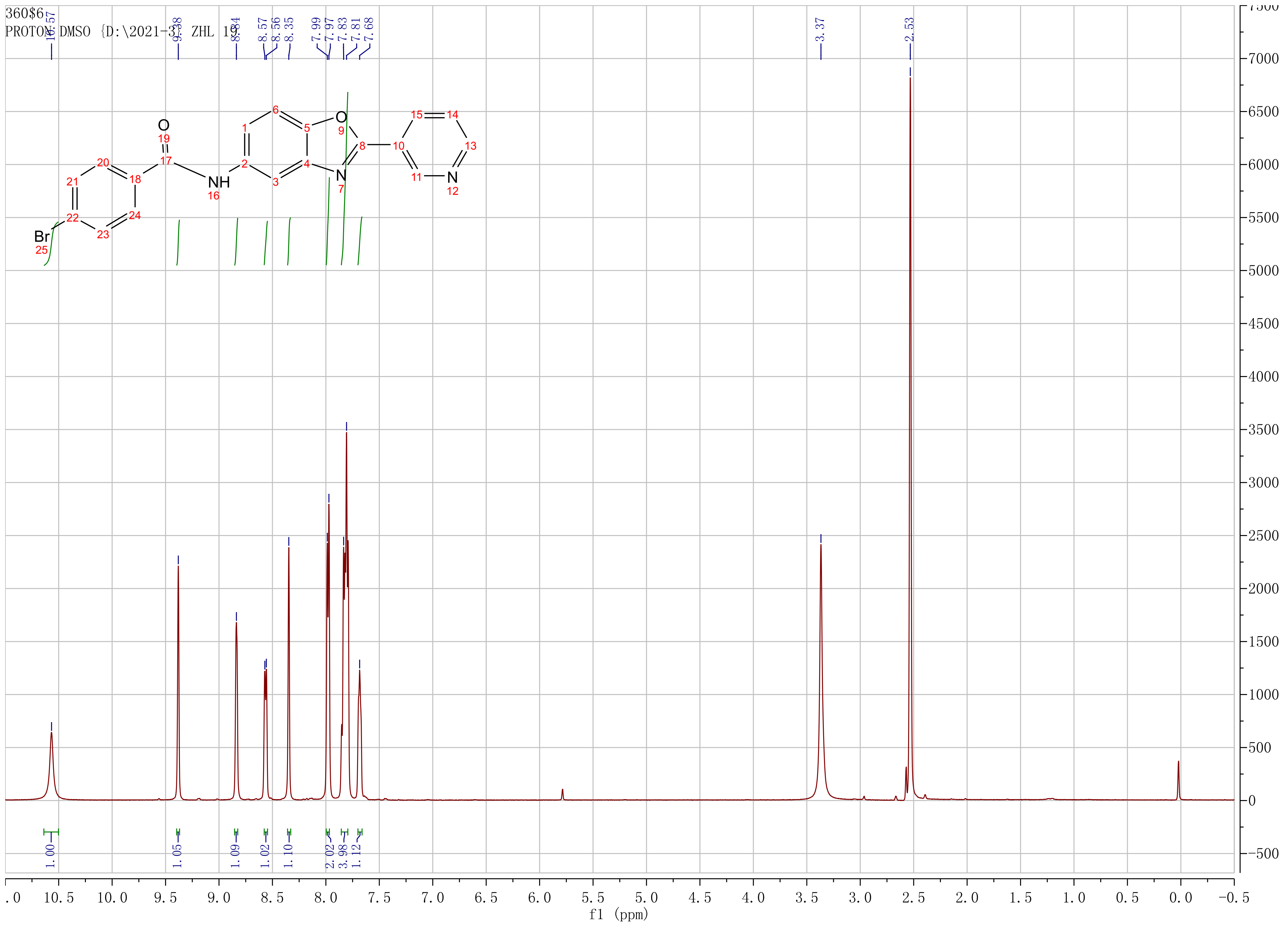

360$6
PROTON DMSO {D:\2021-3} ZHL 19
10.57
9.38
8.84
8.57
8.56
8.35
7.99
7.97
7.83
7.81
7.68
3.37
2.53
1.00
1.05
1.09
1.02
1.10
2.02
3.98
1.12
f1 (ppm)

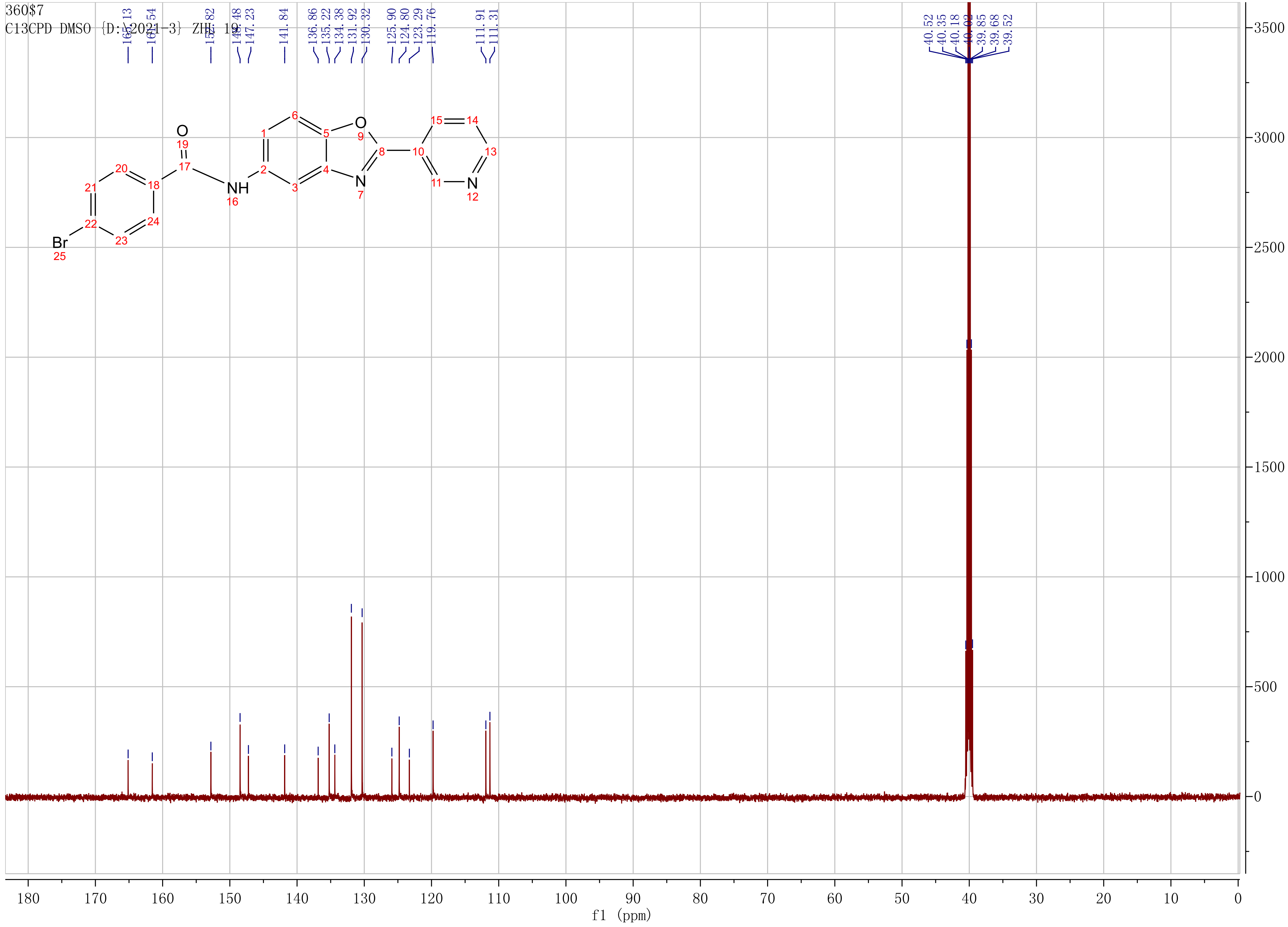
360$7
C13CPD DMSO
147.23
141.84
136.86
135.22
134.38
131.92
130.32
125.90
124.80
123.29
119.76
111.91
111.31
40.52
40.35
40.18
39.85
39.68
39.52
Br
O
NH
N
O
N
N
f1 (ppm)

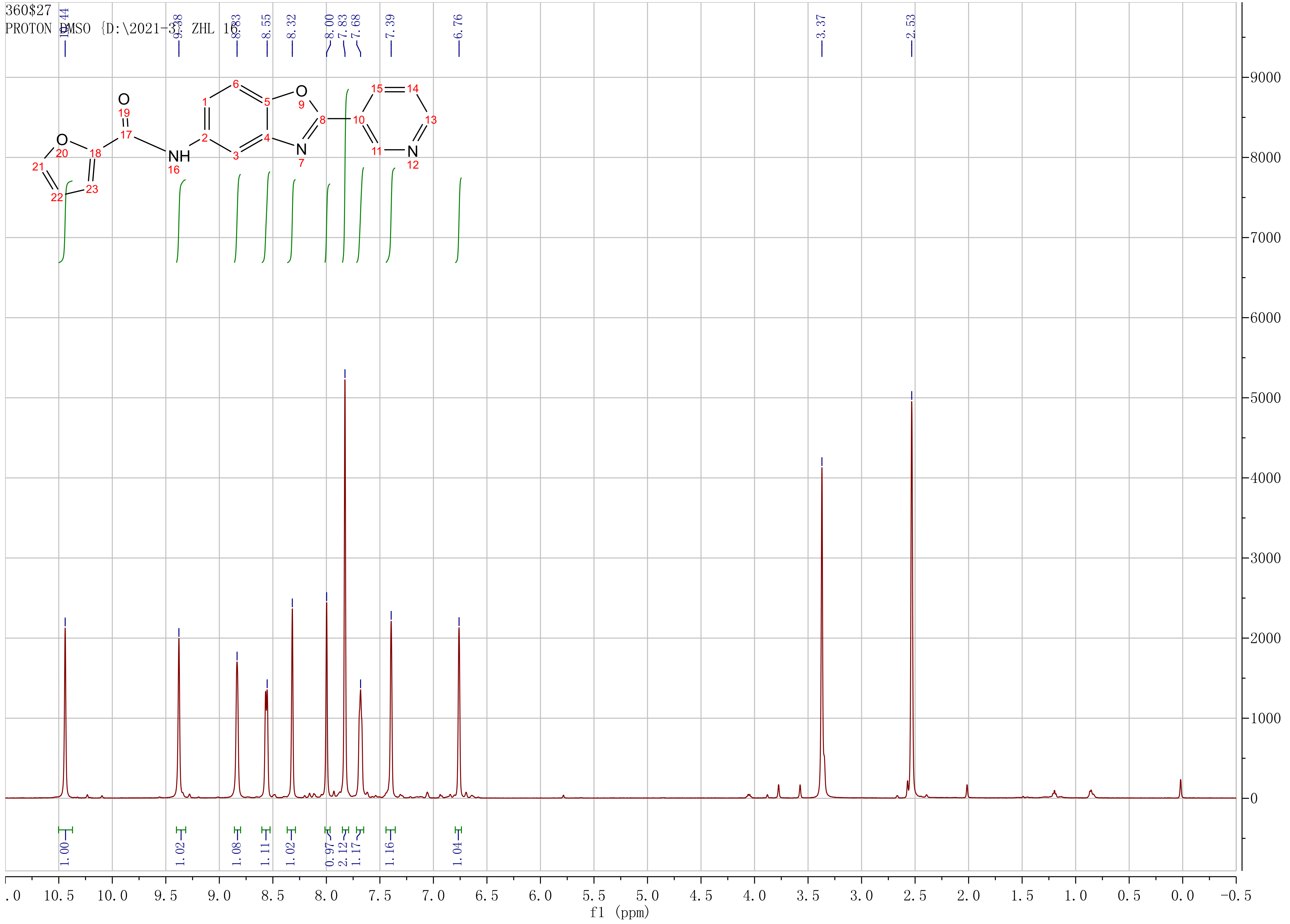
360$27
PROTON DMSO {D:\2021-3} ZHL 16}
10.44
9.38
8.83
8.55
8.32
8.00
7.83
7.68
7.39
6.76
3.37
2.53
f1 (ppm)

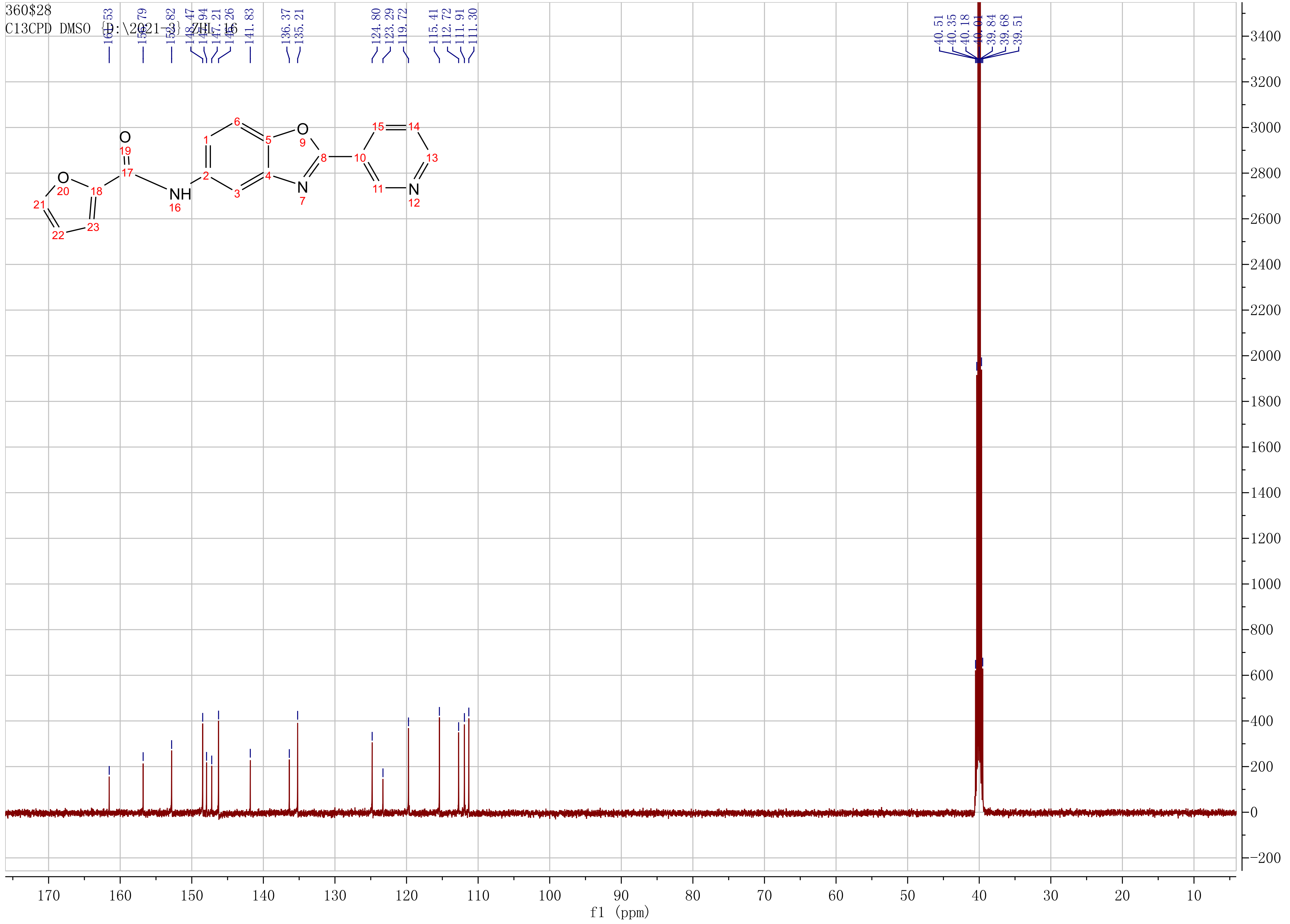

360$28
C13CPD DMSO {D:\2021-3} ZHL 16
161.53
156.79
152.82
148.47
147.94
147.21
146.26
141.83
136.37
135.21
124.80
123.29
119.72
115.41
112.72
111.91
111.30
40.51
40.35
40.18
40.01
39.84
39.68
39.51
f1 (ppm)

360$23
PROTON DMSO {D:\2021-3} ZHL 14

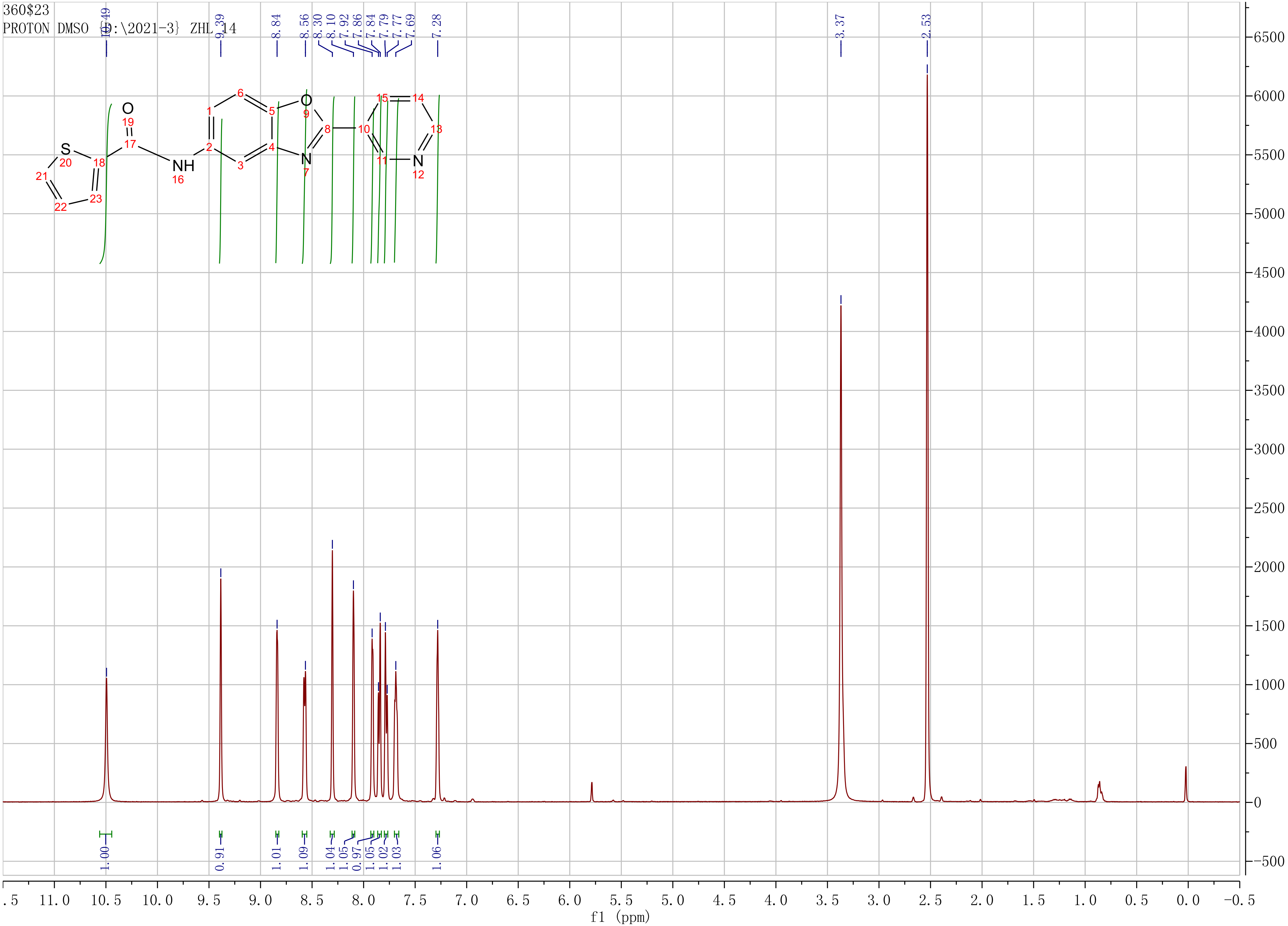

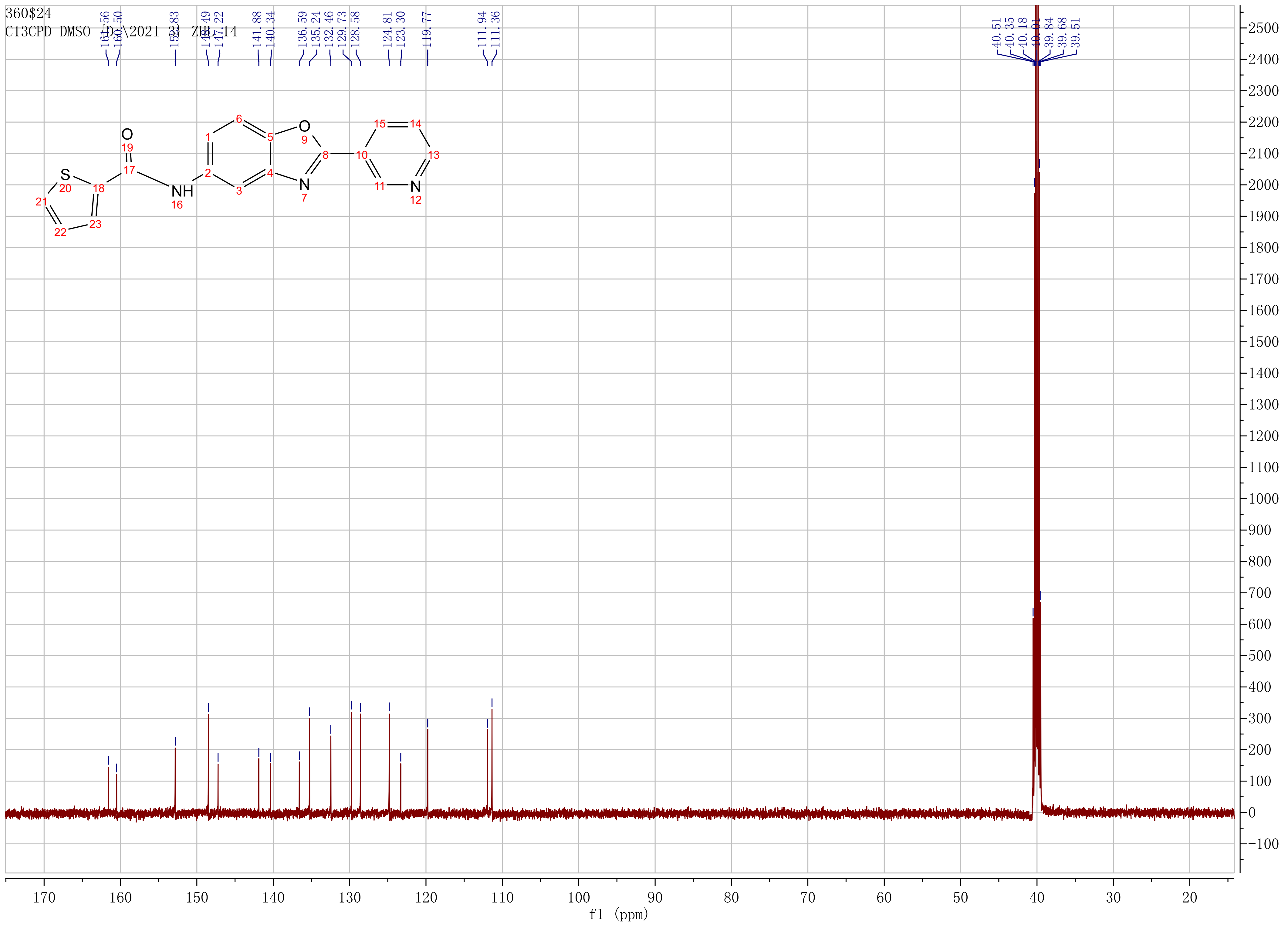
360$24
C13CPD DMSO {D:\2021-3} ZHL 14
161.56
160.50
152.83
148.49
147.22
141.88
140.34
136.59
135.24
132.46
129.73
128.58
124.81
123.30
119.77
111.94
111.36
40.51
40.35
40.18
40.01
39.84
39.68
39.51
f1 (ppm)

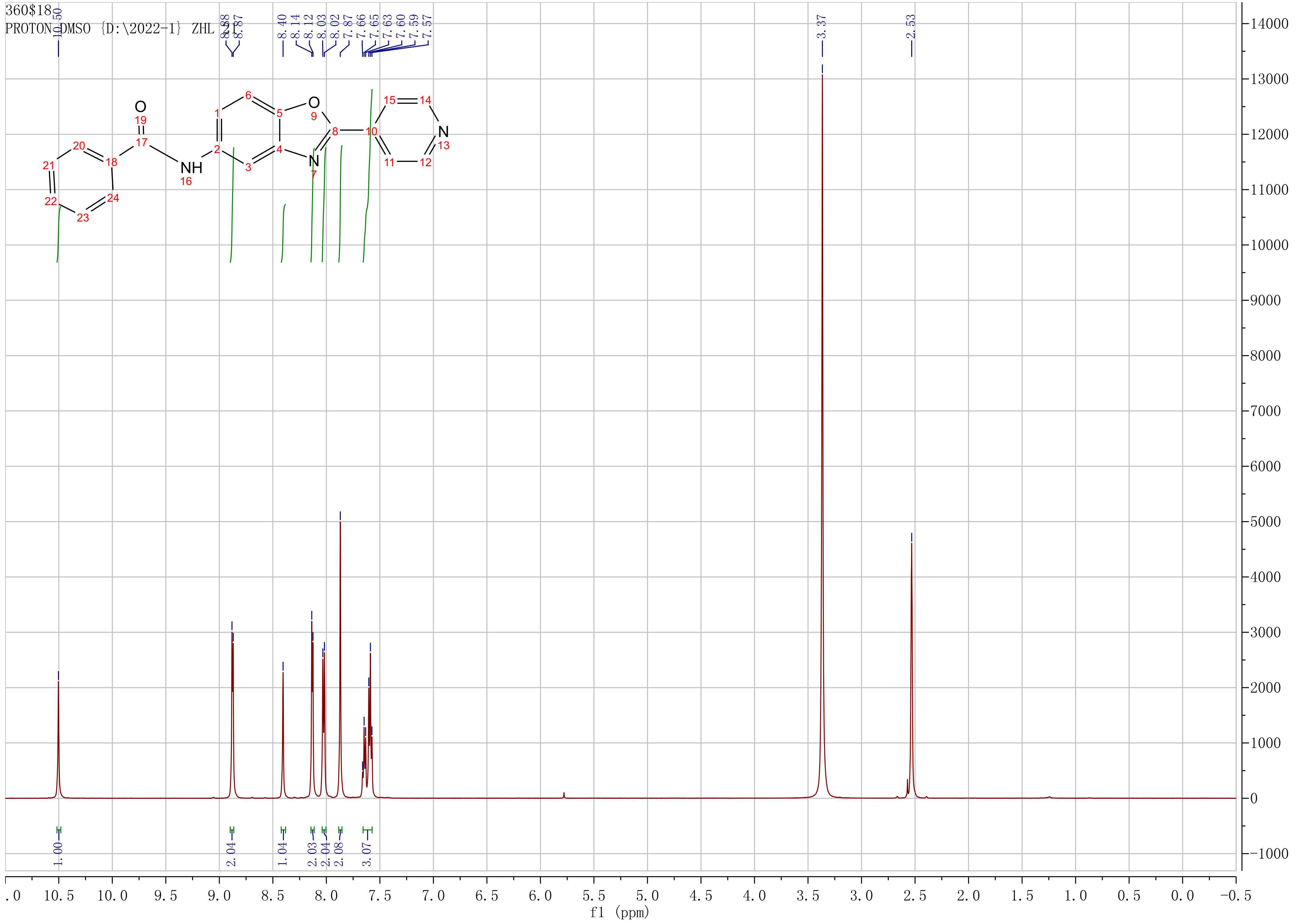
360$18
PROTON-DMSO {D:\2022-1} ZHL
10.50
8.88
8.87
8.40
8.14
8.12
8.03
8.02
7.87
7.66
7.65
7.63
7.60
7.59
7.57
3.37
2.53
1.00
2.04
1.04
2.03
2.04
2.08
3.07
f1 (ppm)

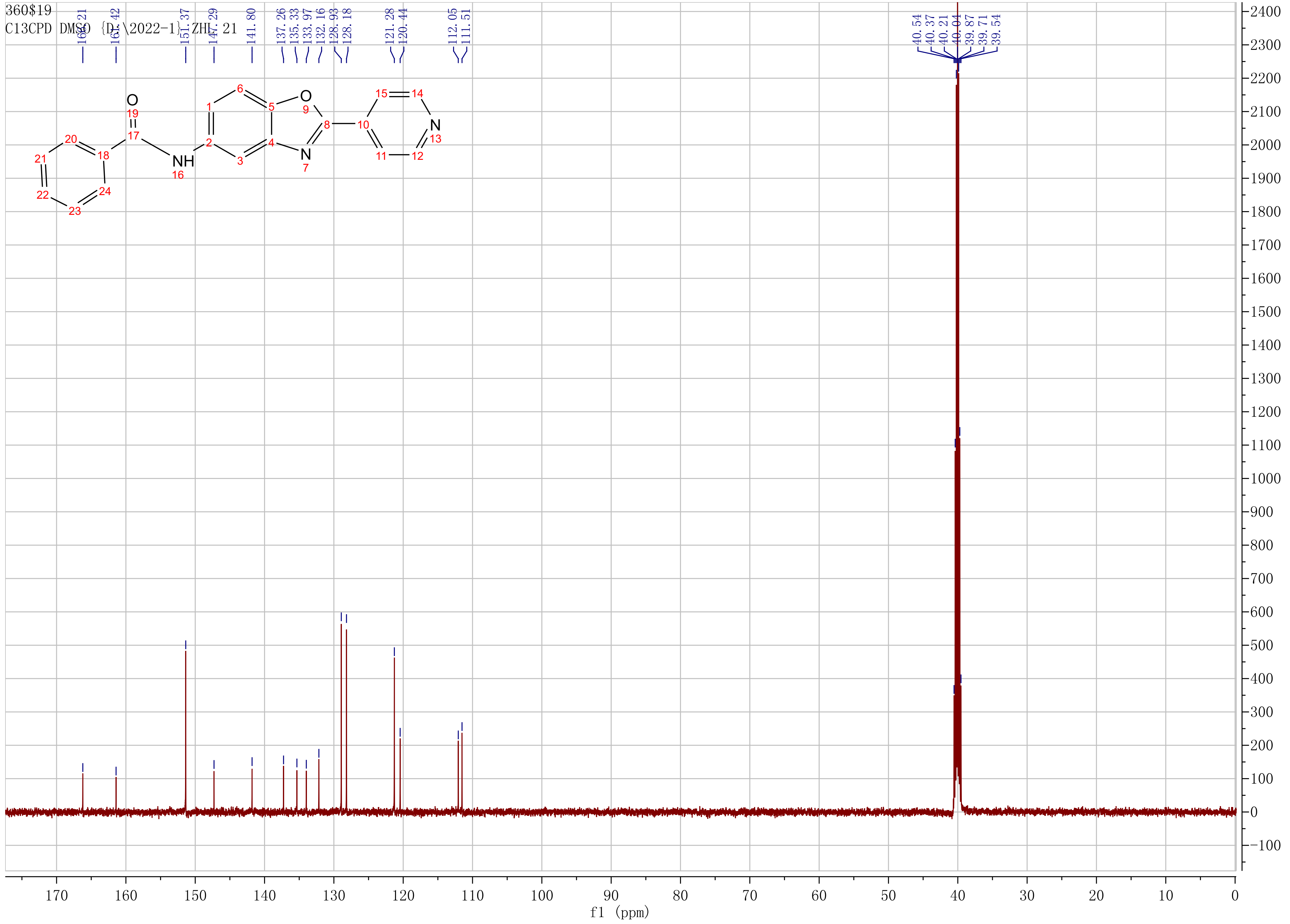

360$19
C13CPD DMSO {D:\2022-1} ZHL 21
166.21
161.42
151.37
147.29
141.80
137.26
135.33
133.97
132.16
128.93
128.18
121.28
120.44
112.05
111.51
40.54
40.37
40.21
40.04
39.87
39.71
39.54
f1 (ppm)

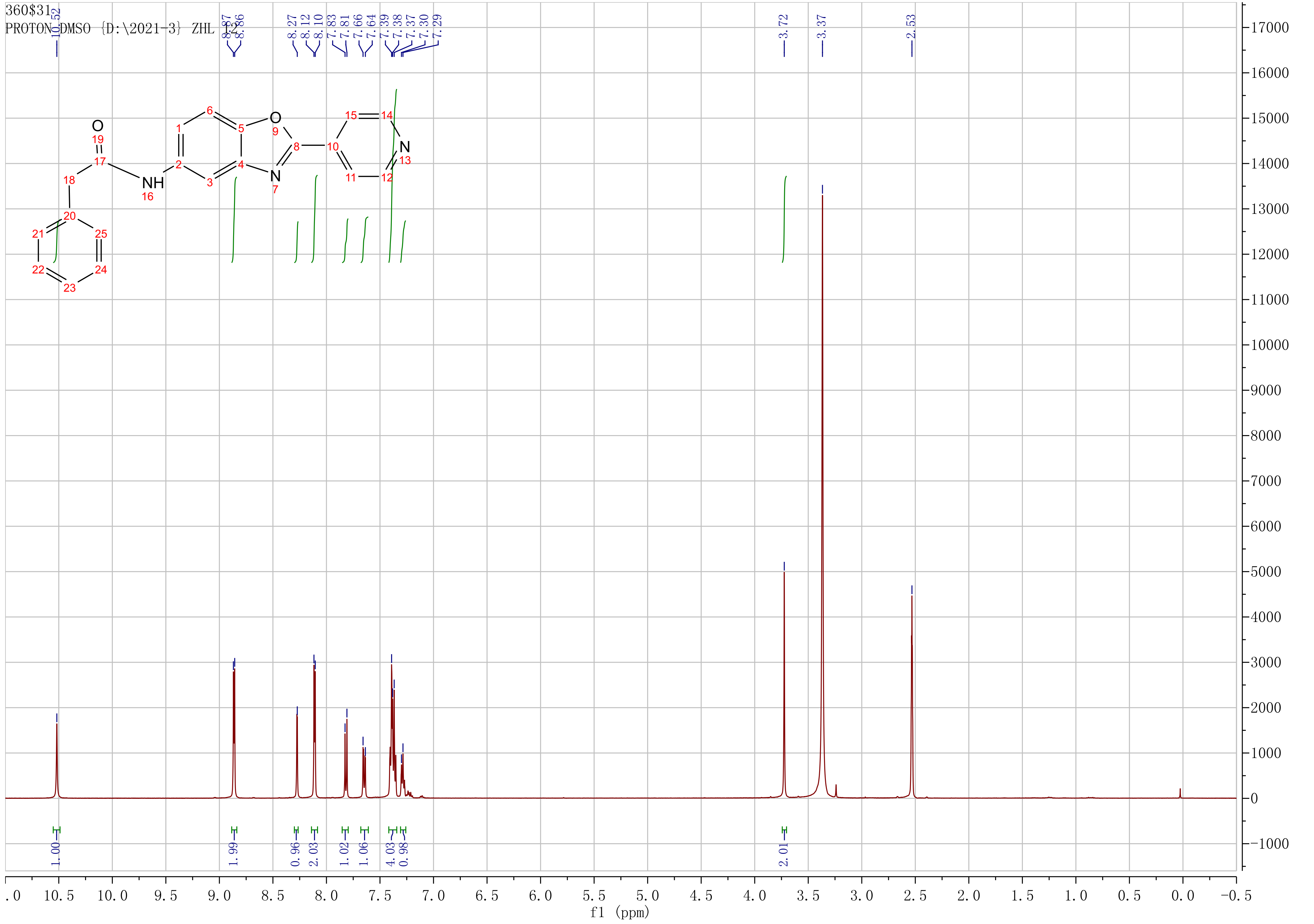
360$31
PROTON_DMSO {D:\2021-3} ZHL 12
10.52
8.87
8.86
8.27
8.12
8.10
7.83
7.81
7.66
7.64
7.39
7.38
7.37
7.30
7.29
3.72
3.37
2.53
1.00
1.99
0.96
2.03
1.02
1.06
4.03
0.98
2.01
f1 (ppm)

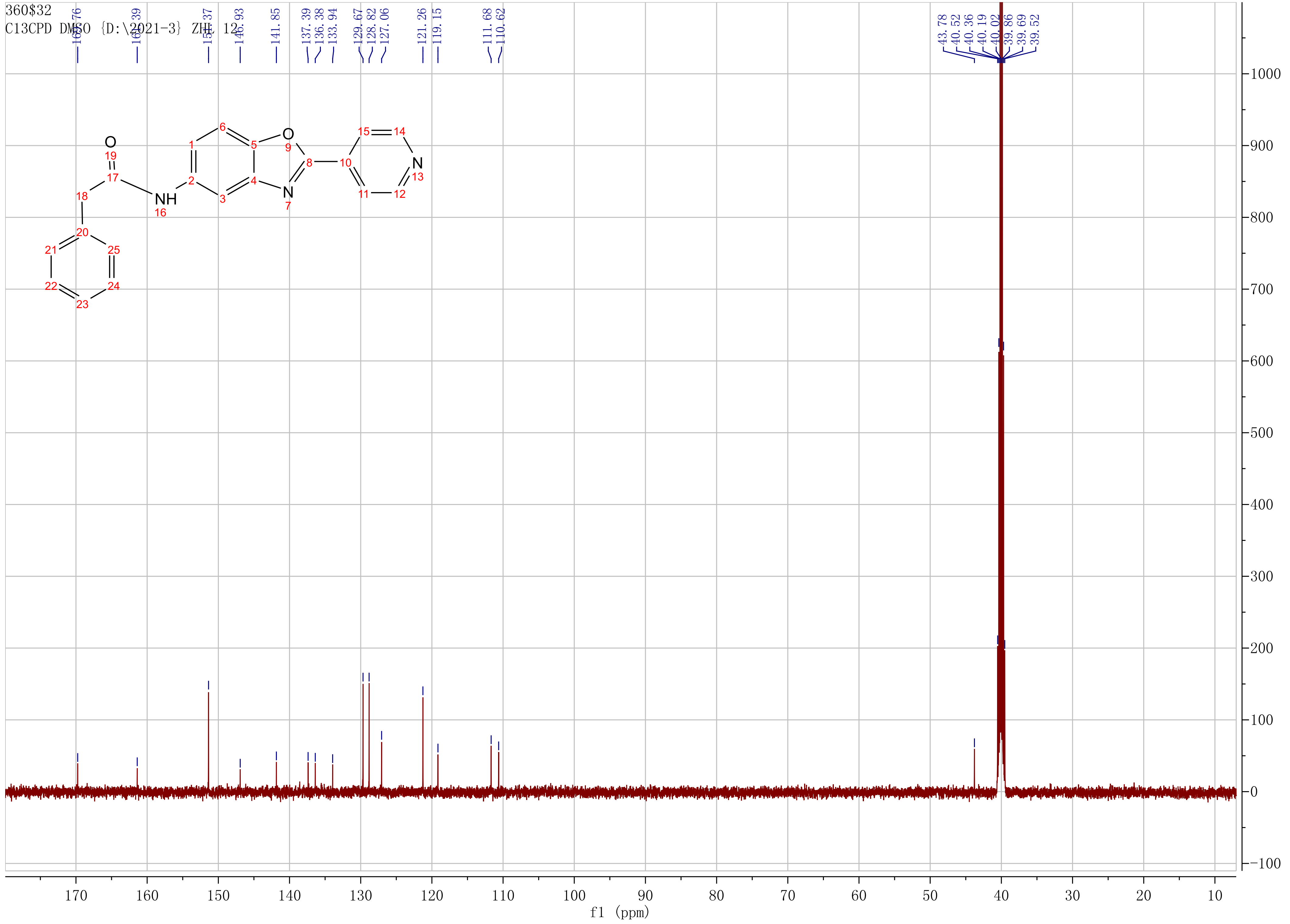
360$32
C13CPD DMSO {D:\2021-3} ZHL 12
141.85
137.39
136.38
133.94
129.67
128.82
127.06
121.26
119.15
111.68
110.62
43.78
40.52
40.36
40.19
40.02
39.86
39.69
39.52
f1 (ppm)

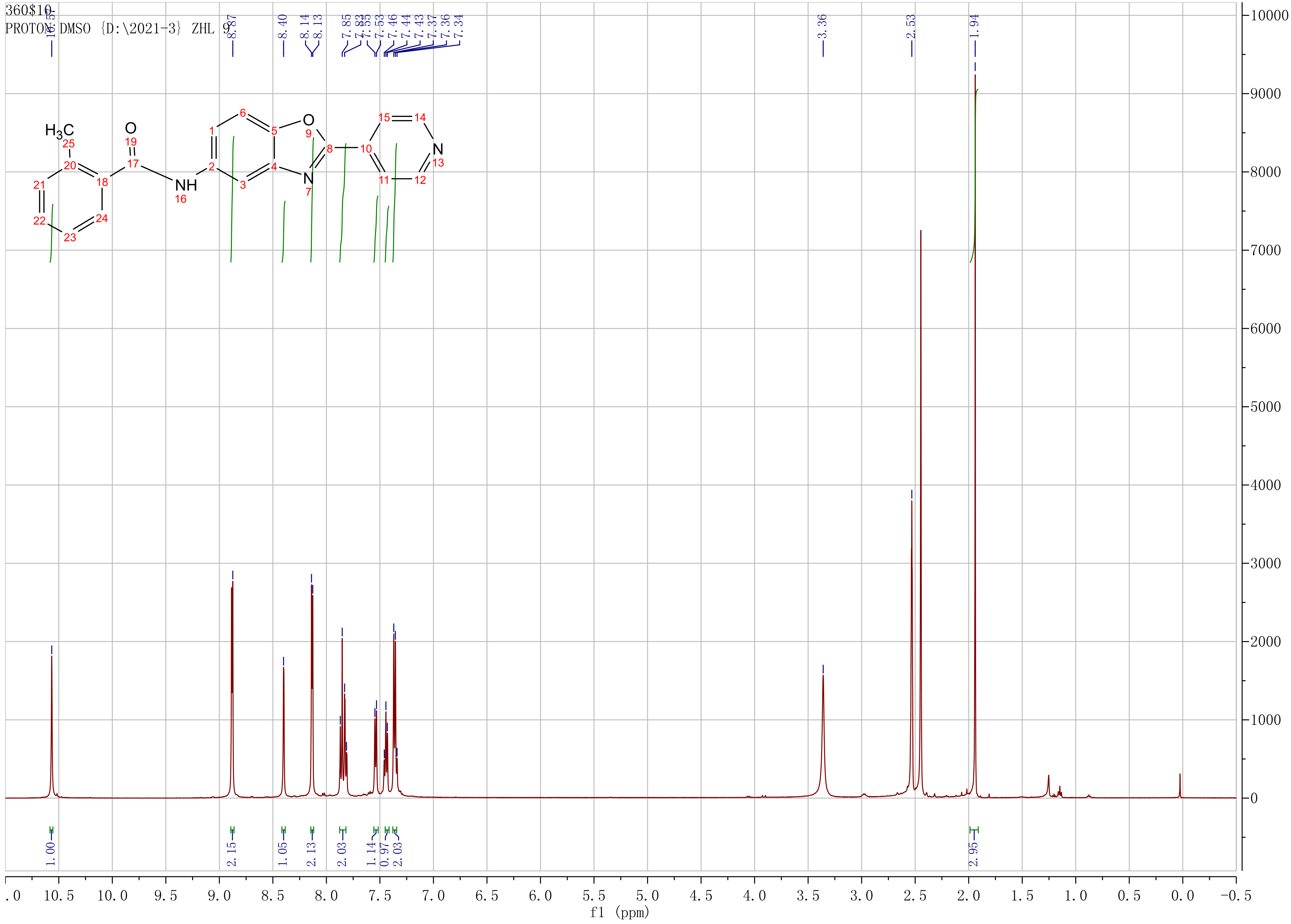
360$10
PROTON DMSO {D:\2021-3} ZHL 9
10.57
8.87
8.40
8.14
8.13
7.85
7.83
7.55
7.53
7.46
7.44
7.43
7.37
7.36
7.34
3.36
2.53
1.94
H_3C
O
NH
O
N
N
1.00
2.15
1.05
2.13
2.03
1.14
0.97
2.03
2.95
f1 (ppm)

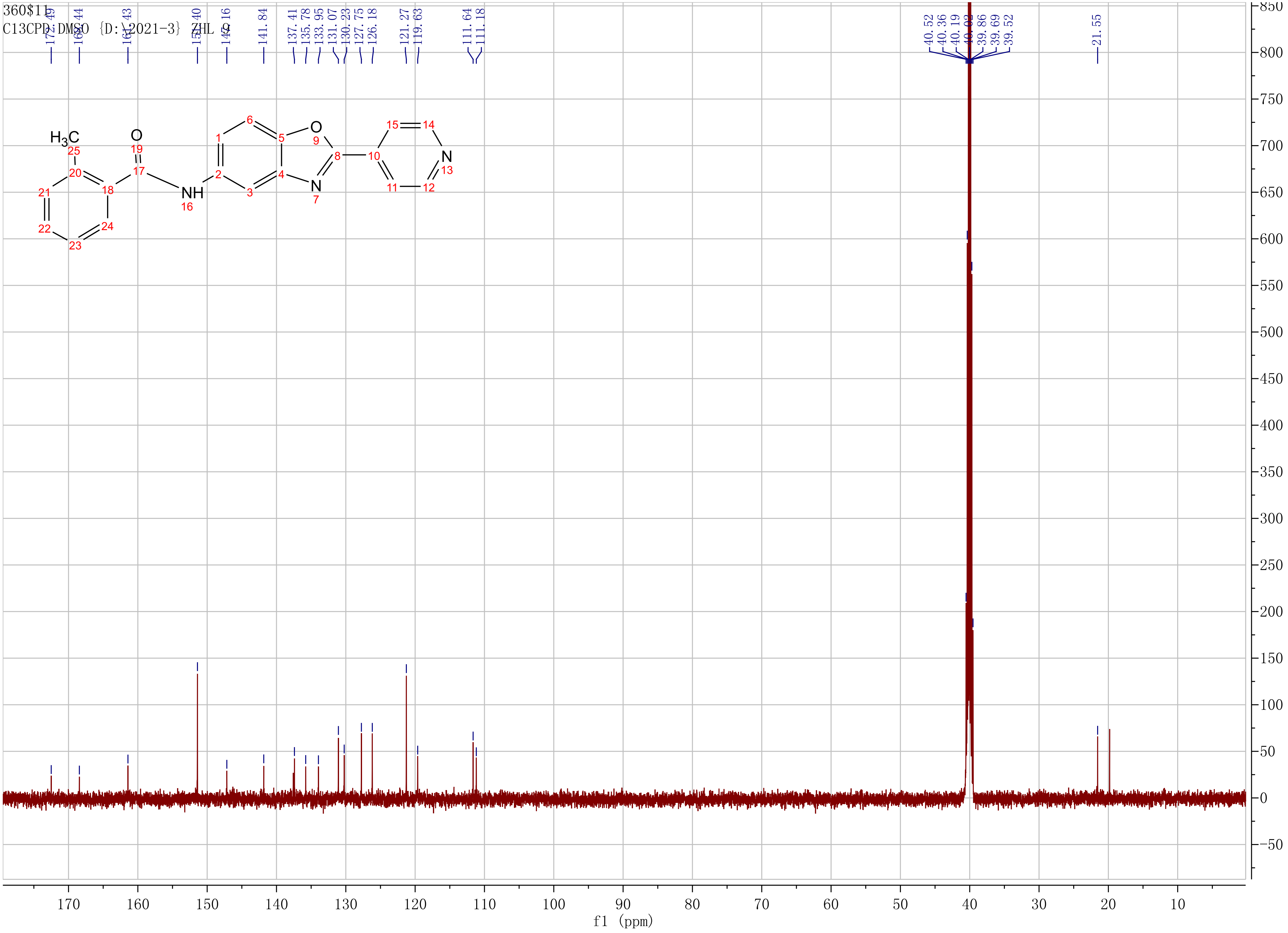360$11
C13CPD:DMSO {D:\2021-3} ZHL
168.44
161.43
141.84
137.41
135.78
133.95
131.07
130.23
127.75
126.18
121.27
119.63
111.64
111.18
40.52
40.36
40.19
39.86
39.69
39.52
21.55
f1 (ppm)

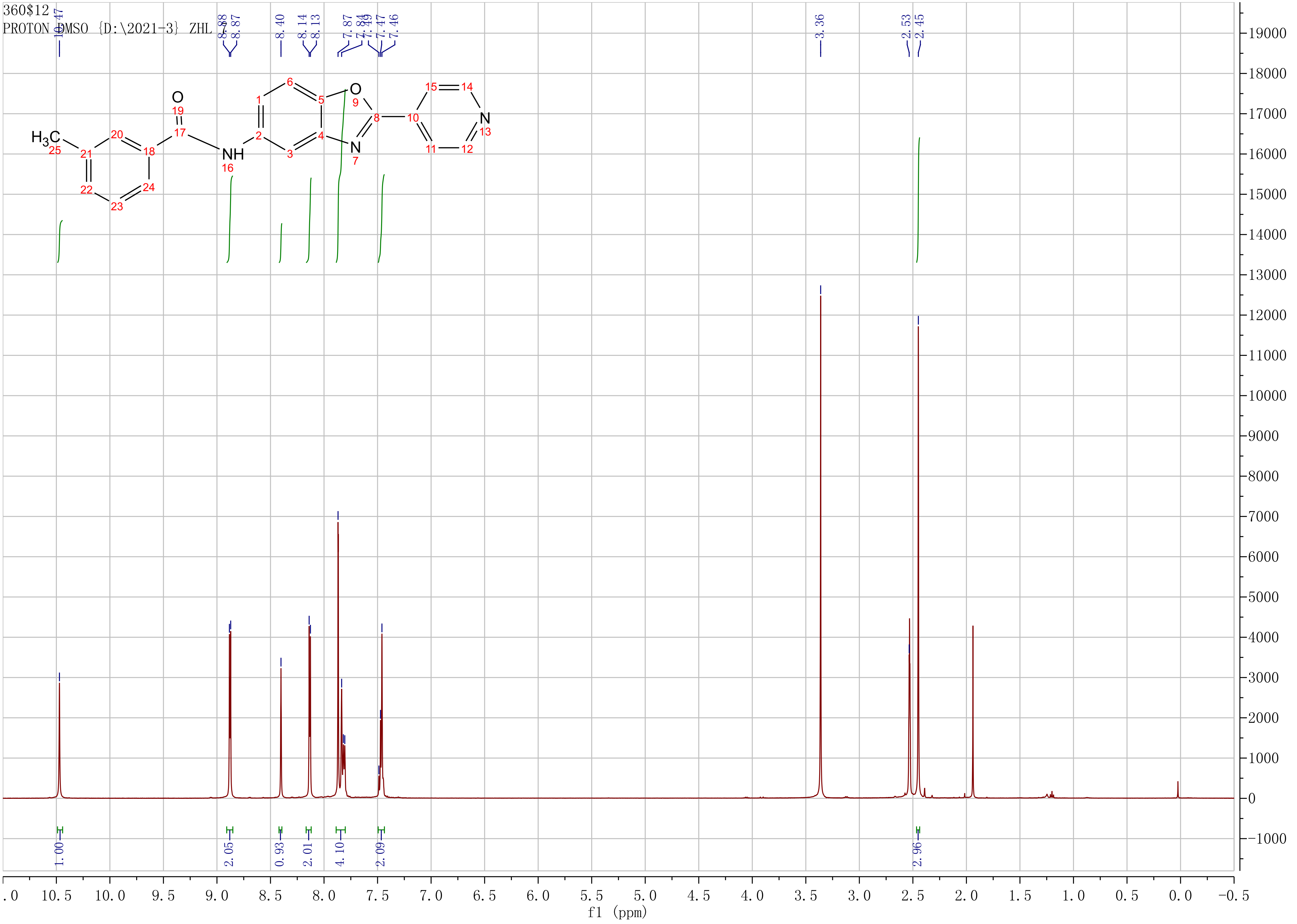
360$12
PROTON DMSO {D:\2021-3} ZHL
10.47
8.88
8.87
8.40
8.14
8.13
7.87
7.84
7.49
7.47
7.46
3.36
2.53
2.45
1.00
2.05
0.93
2.01
4.10
2.09
2.96
f1 (ppm)

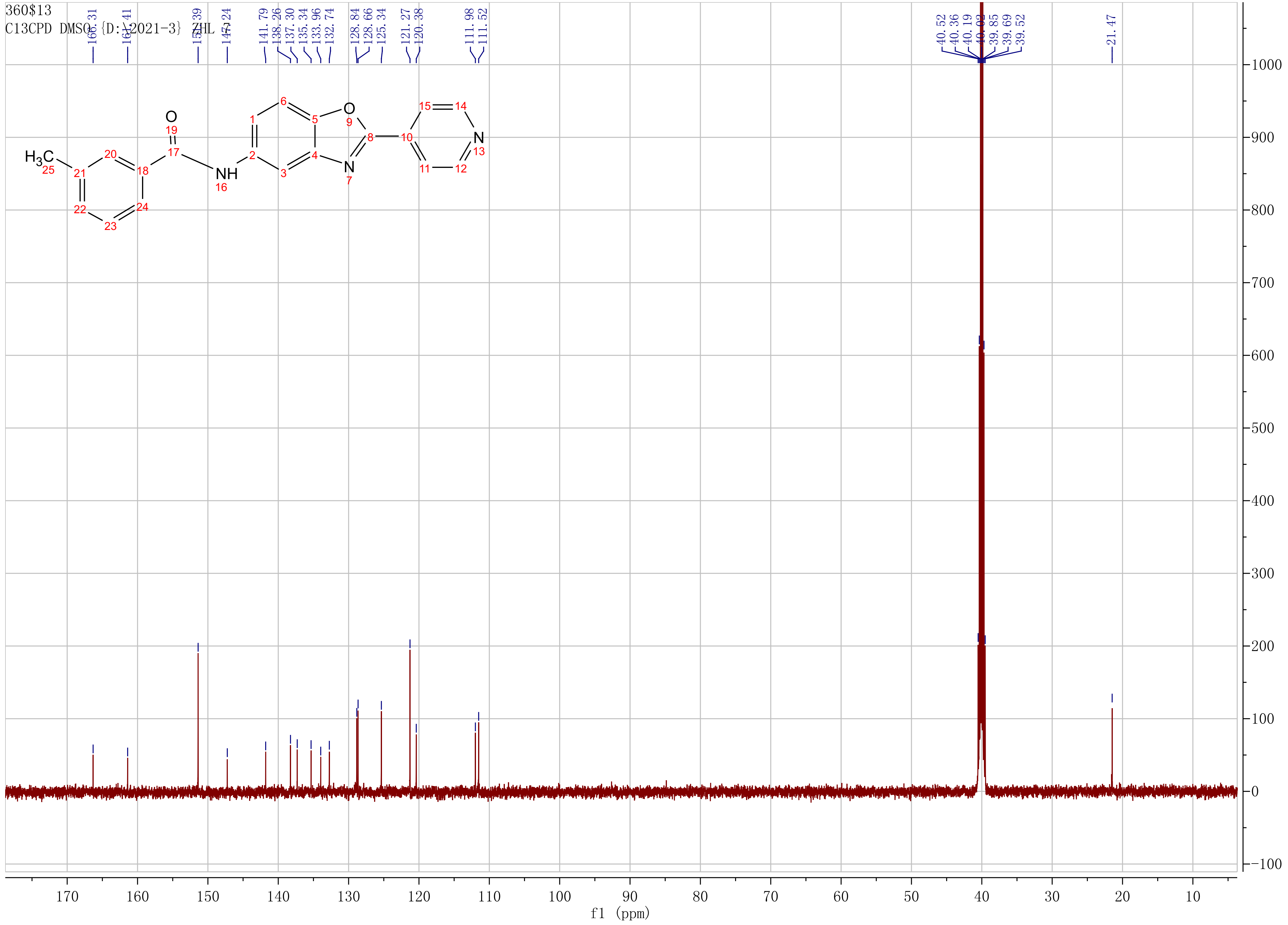
360$13
C13CPD DMSO {D:\2021-3} ZHL 7
166.31
161.41
151.39
147.24
141.79
138.26
137.30
135.34
133.96
132.74
128.84
128.66
125.34
121.27
120.38
111.98
111.52
40.52
40.36
40.19
40.02
39.85
39.69
39.52
21.47
f1 (ppm)

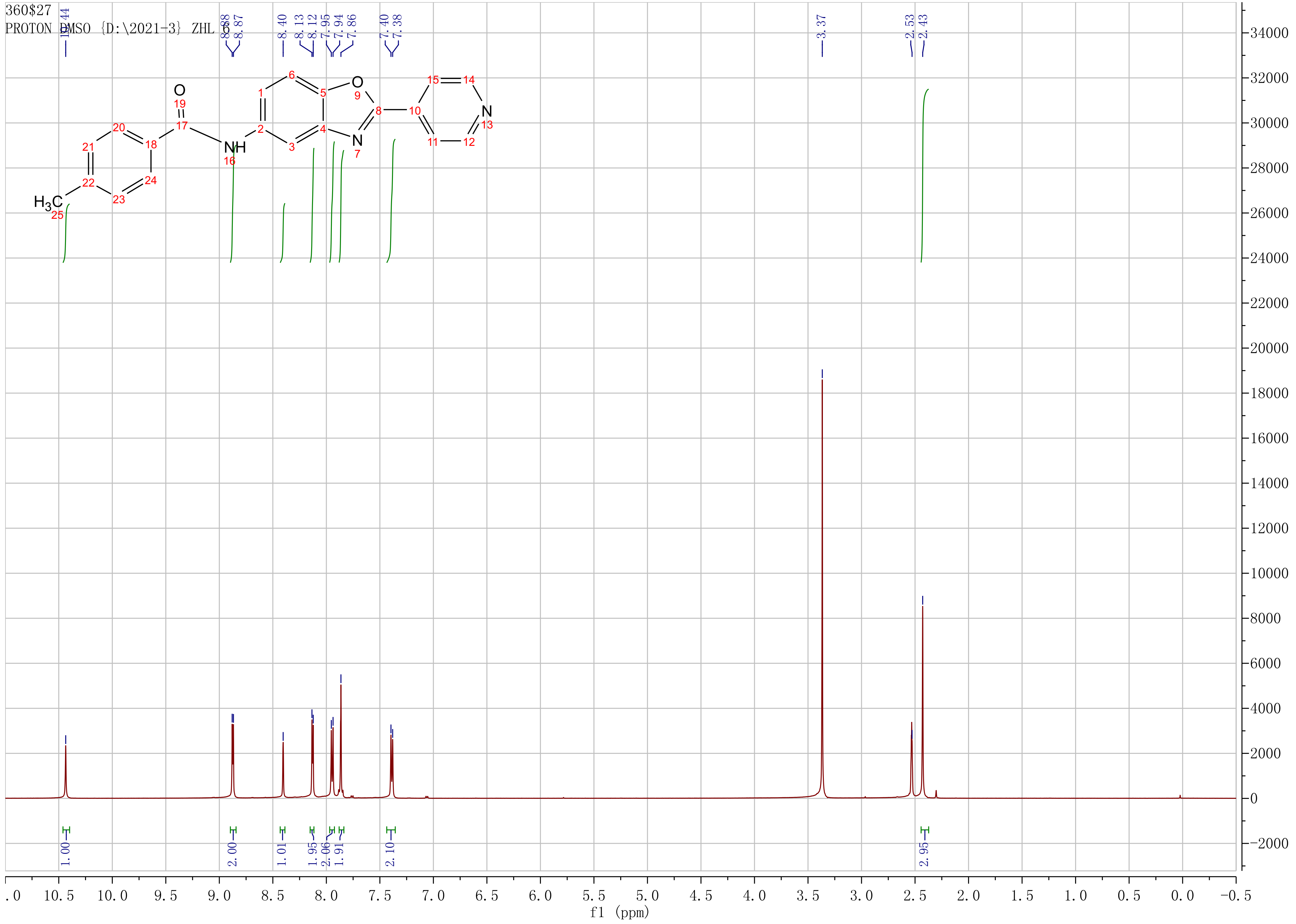

360$27
PROTON DMSO {D:\2021-3} ZHL 6
10.44
8.88
8.87
8.40
8.13
8.12
7.95
7.94
7.86
7.40
7.38
3.37
2.53
2.43
1.00
2.00
1.01
1.95
2.06
1.91
2.10
2.95
f1 (ppm)

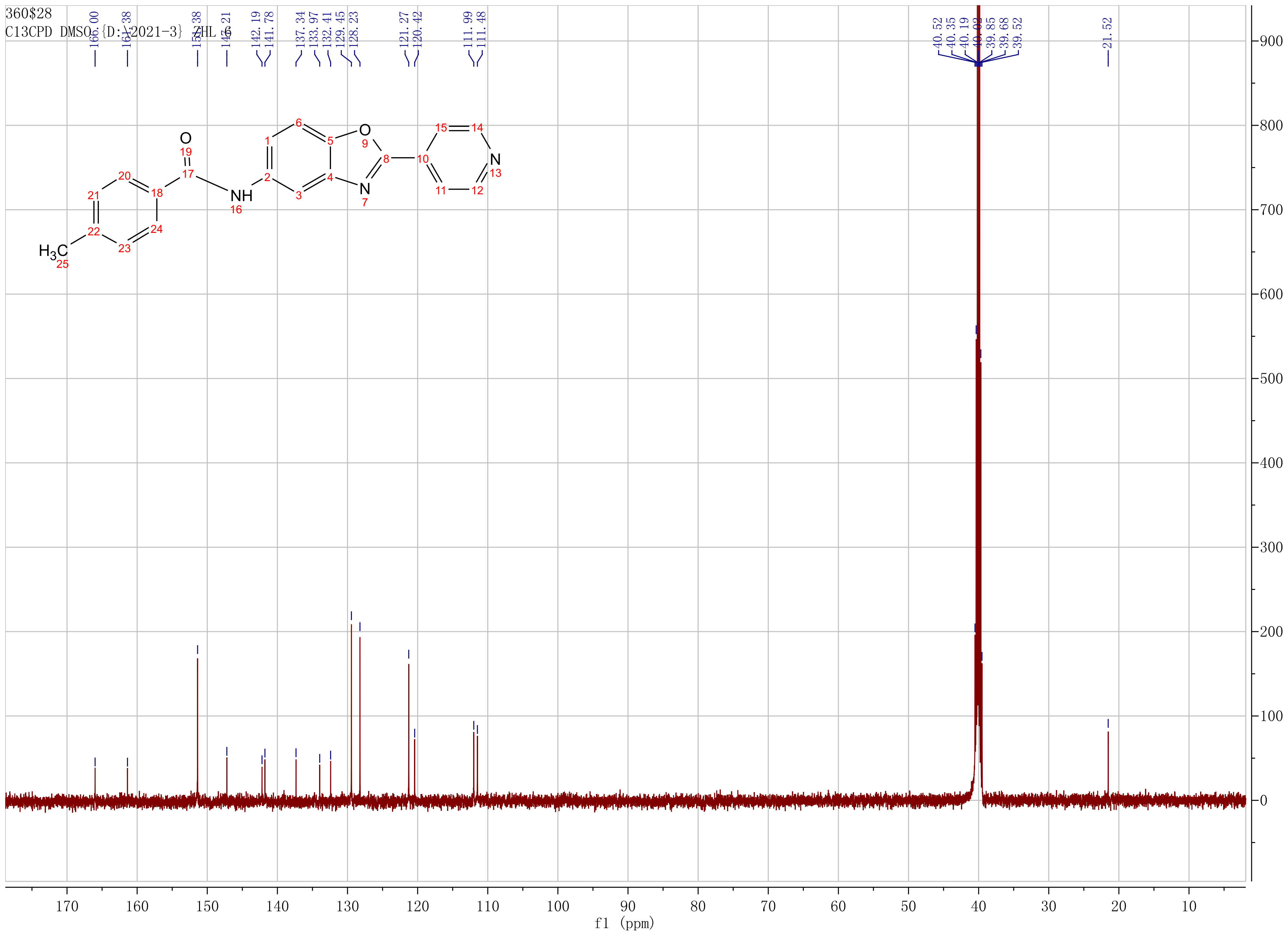
360$28
C13CPD DMSO {D:\2021-3} ZHL-6
166.00
161.38
151.38
147.21
142.19
141.78
137.34
133.97
132.41
129.45
128.23
121.27
120.42
111.99
111.48
40.52
40.35
40.19
40.02
39.85
39.68
39.52
21.52
f1 (ppm)

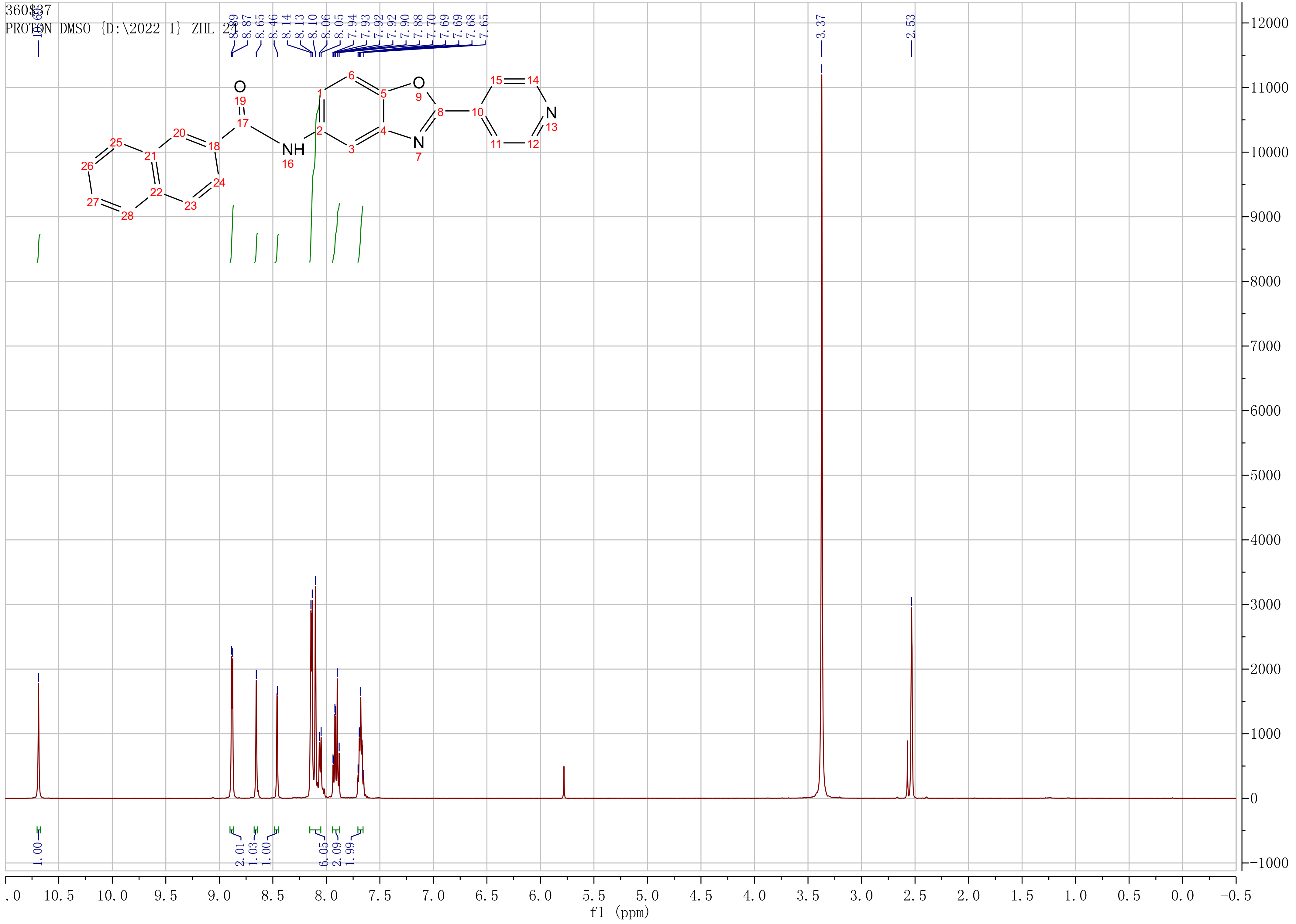
360$37
PROTON DMSO {D:\2022-1} ZHL 24
8.89
8.87
8.65
8.46
8.14
8.13
8.10
8.06
8.05
7.94
7.93
7.92
7.92
7.90
7.88
7.70
7.69
7.69
7.68
7.65
3.37
2.53
1.00
2.01
1.03
1.00
6.05
2.09
1.99
f1 (ppm)

360$38
C13CPD DMSO {D:\2022-1} ZHL 24

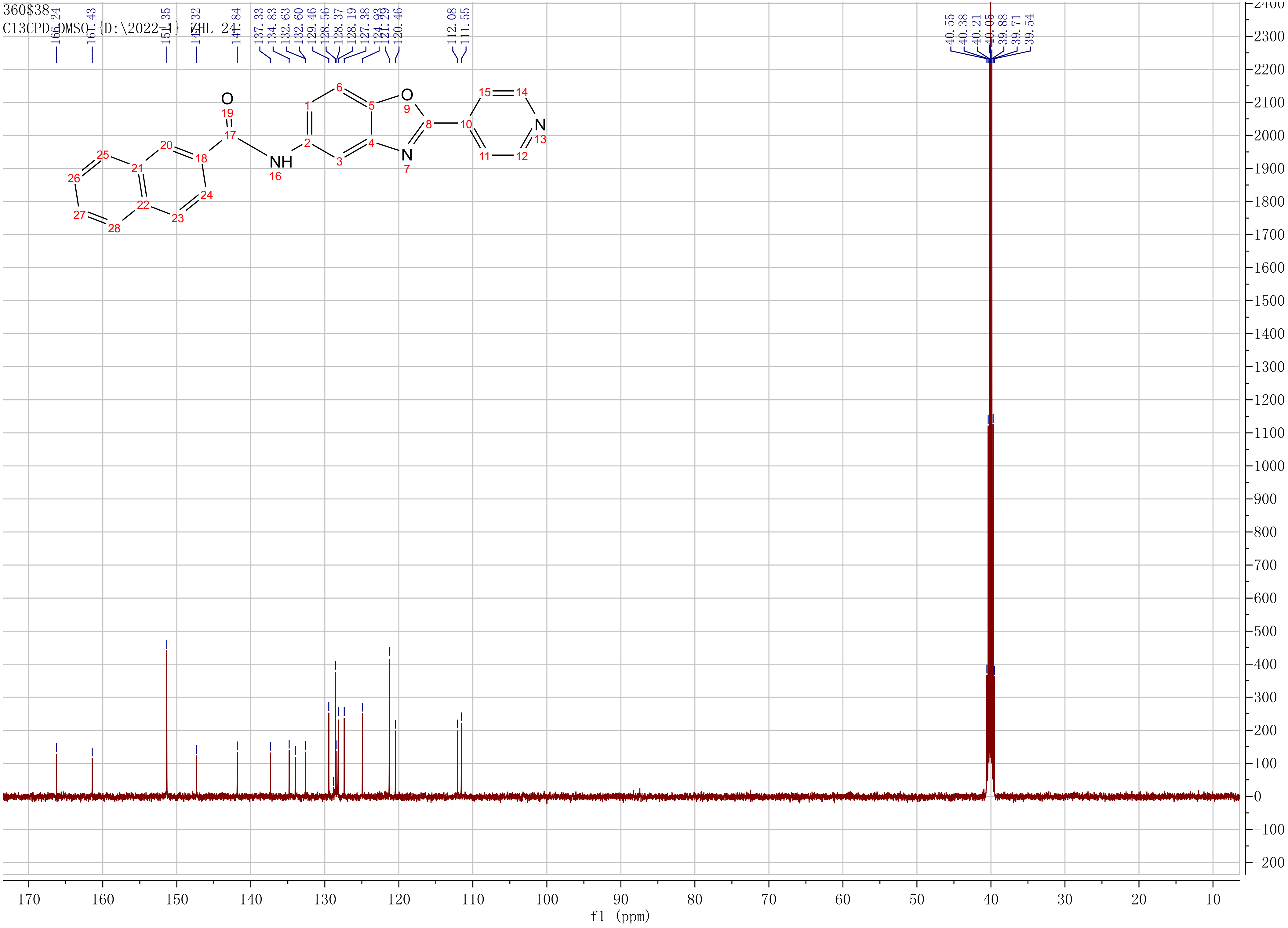

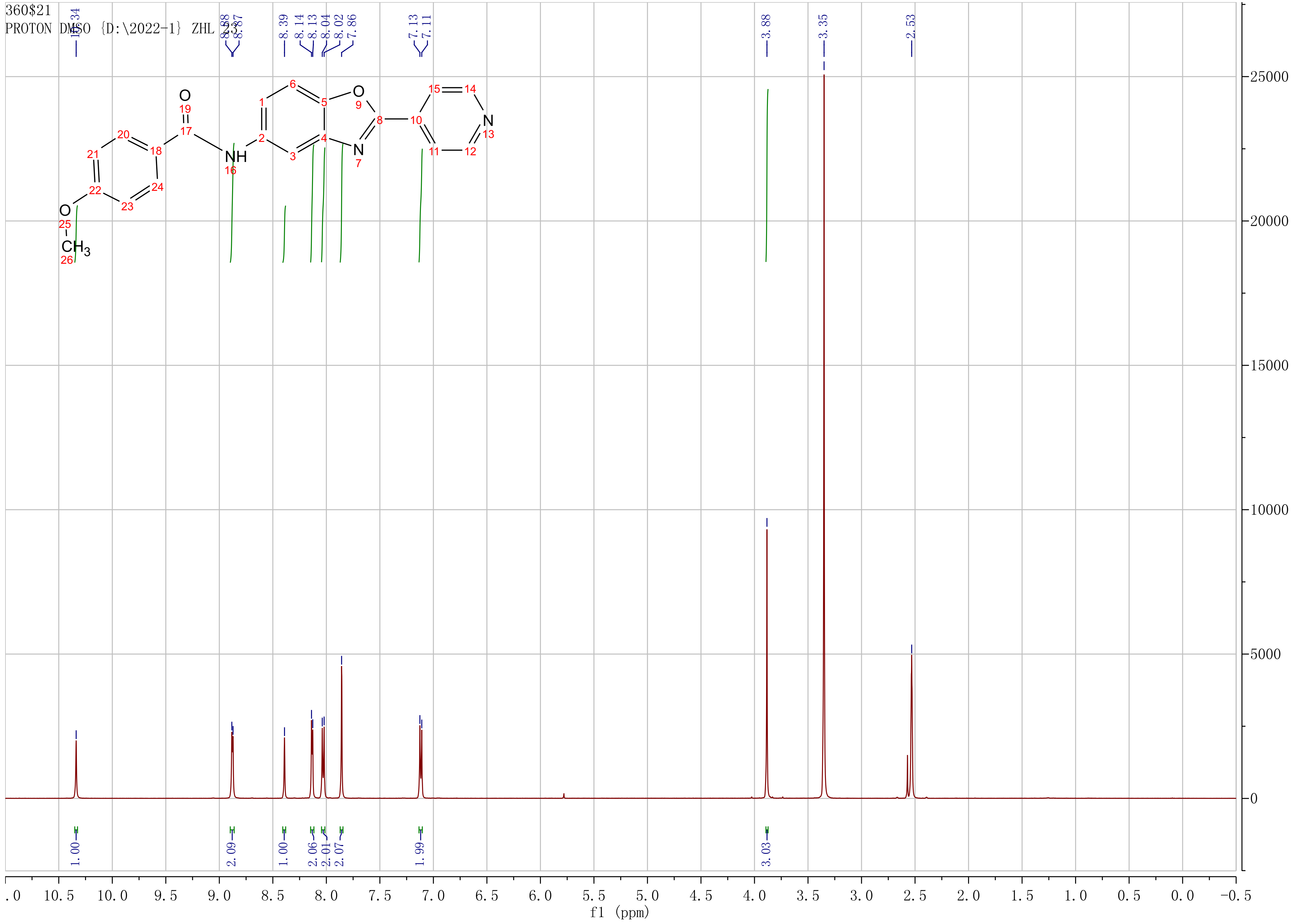

360$21
PROTON DMSO {D:\2022-1} ZHL 23
10.34
8.88
8.87
8.39
8.14
8.13
8.04
8.02
7.86
7.13
7.11
3.88
3.35
2.53
1.00
2.09
1.00
2.06
2.01
2.07
1.99
3.03
f1 (ppm)

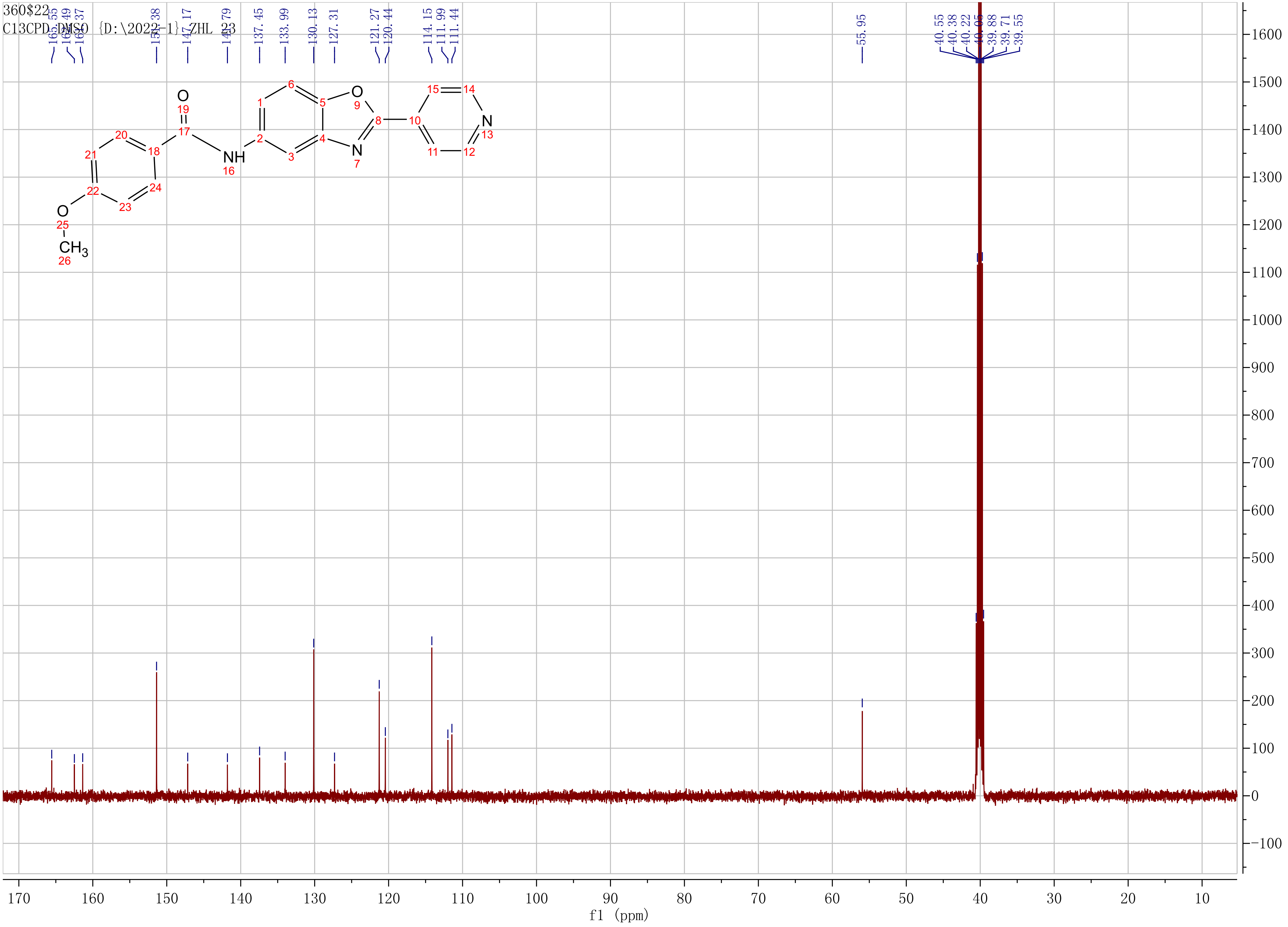
360$22
C13CPD-DMSO {D:\2022-1} ZHL 23
147.17
137.45
133.99
130.13
127.31
121.27
120.44
114.15
111.99
111.44
55.95
40.55
40.38
40.22
39.88
39.71
39.55
NH
CH3
f1 (ppm)

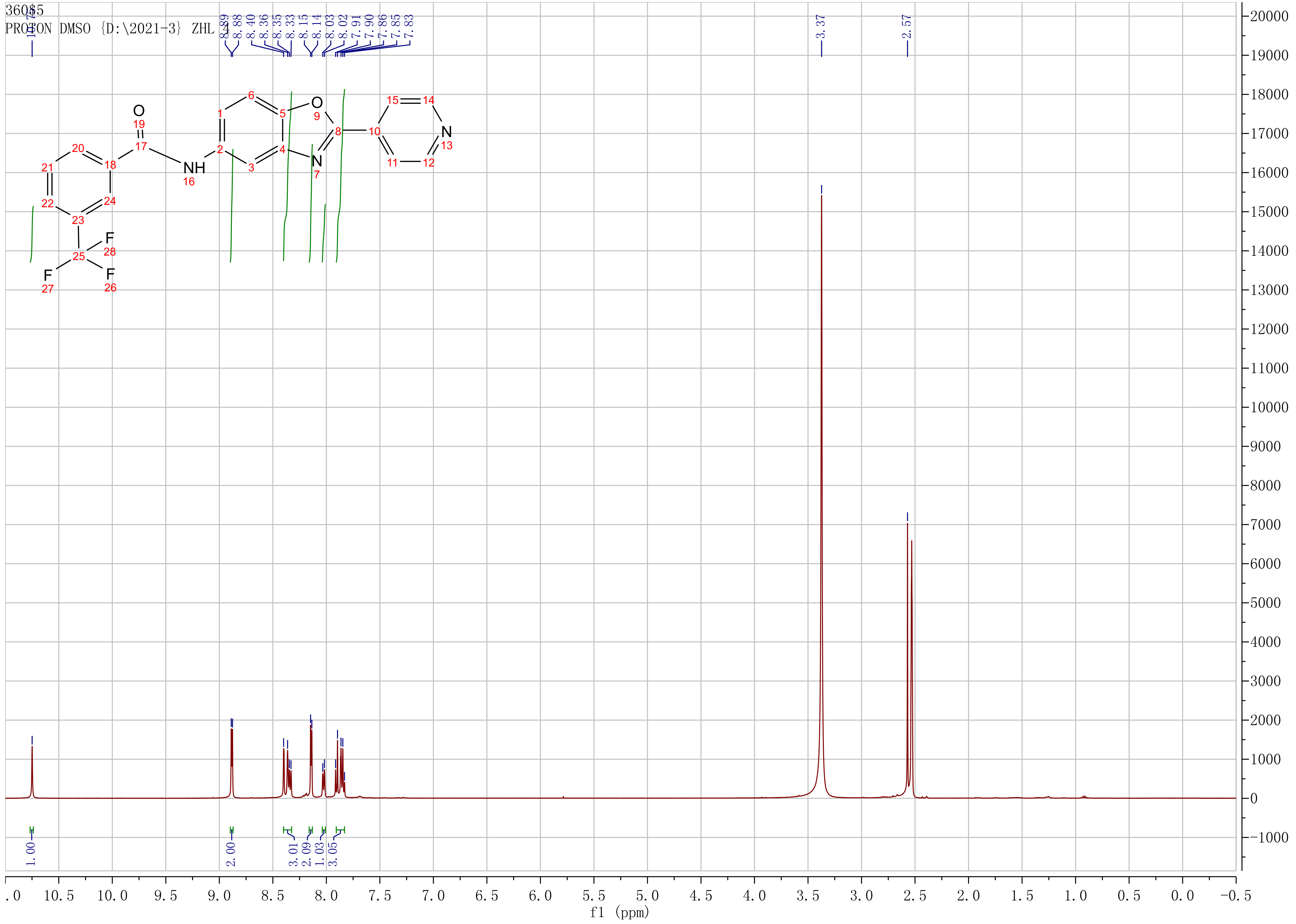

36085
PROTON DMSO {D:\2021-3} ZHL 4
10.75
8.89
8.88
8.40
8.36
8.35
8.33
8.15
8.14
8.03
8.02
7.91
7.90
7.86
7.85
7.83
3.37
2.57
1.00
2.00
3.01
2.09
1.03
3.05
f1 (ppm)

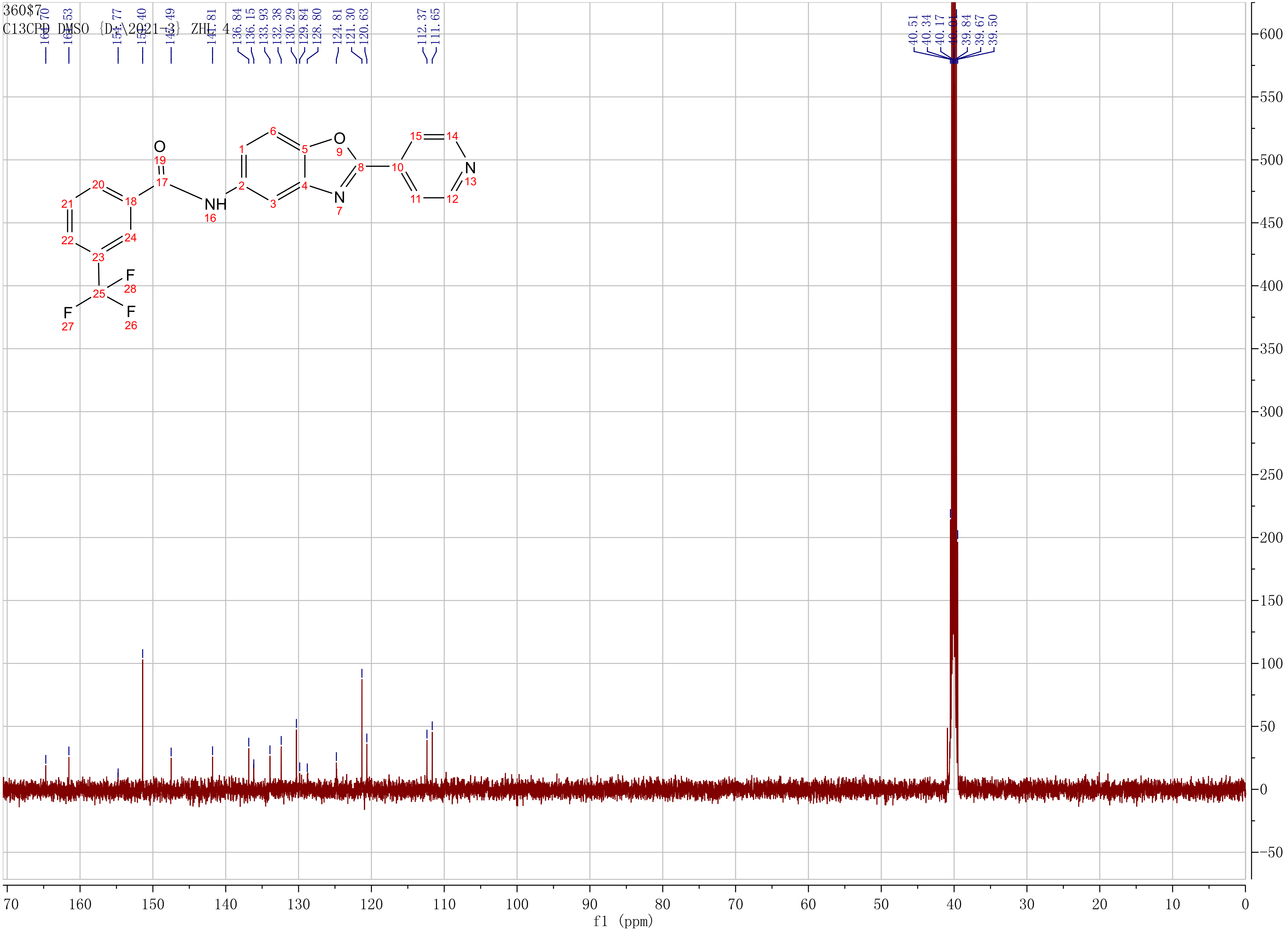

360$7
C13CPD DMSO {D:\2021-3} ZHL 4
136.84
136.15
133.93
132.38
130.29
129.84
128.80
124.81
121.30
120.63
112.37
111.65
40.51
40.34
40.17
39.84
39.67
39.50
f1 (ppm)

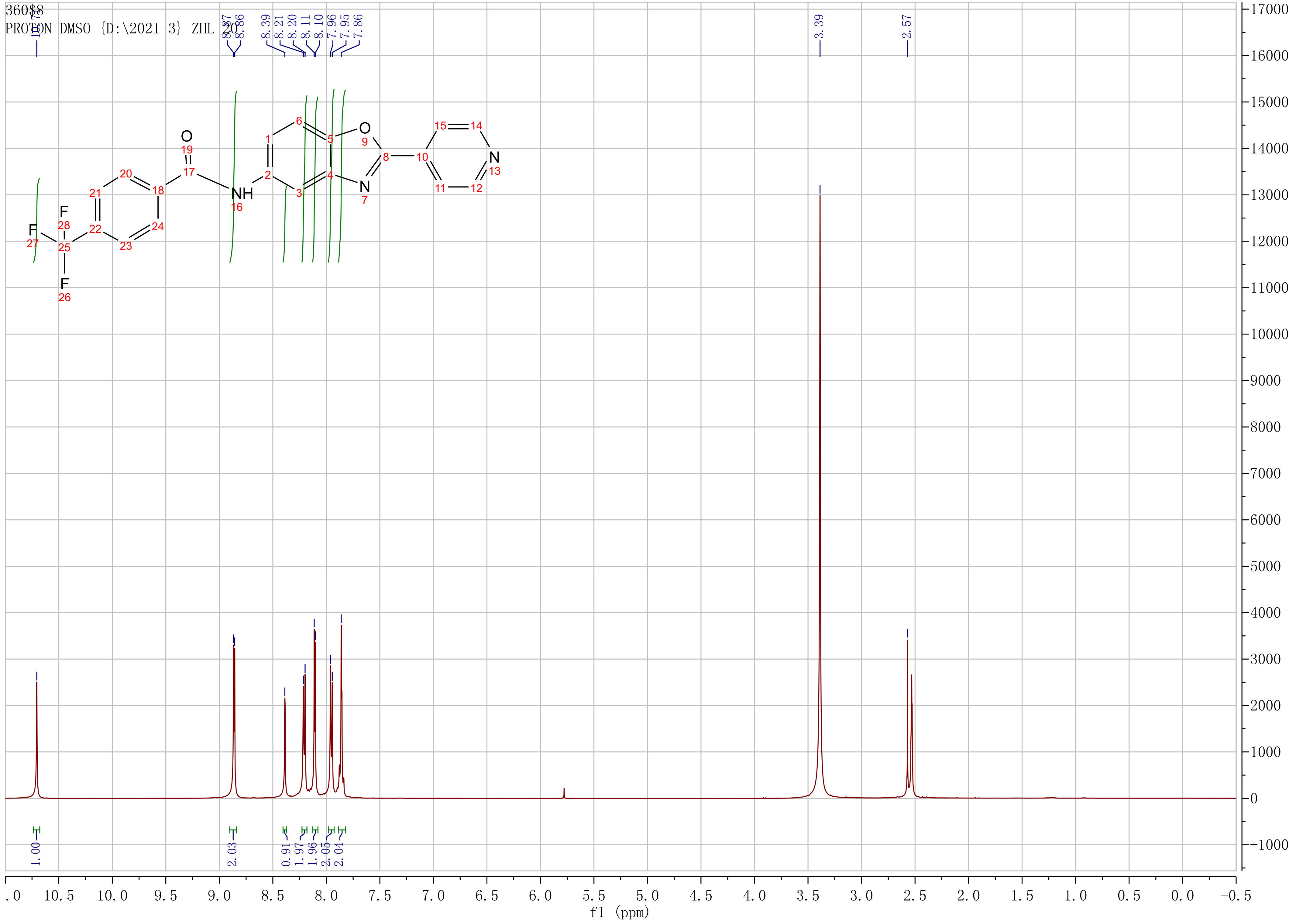
360$$8
PROTON DMSO {D:\2021-3} ZHL 20
8.87
8.86
8.39
8.21
8.20
8.11
8.10
7.96
7.95
7.86
3.39
2.57
1.00
2.03
0.91
1.97
1.96
2.05
2.04
f1 (ppm)

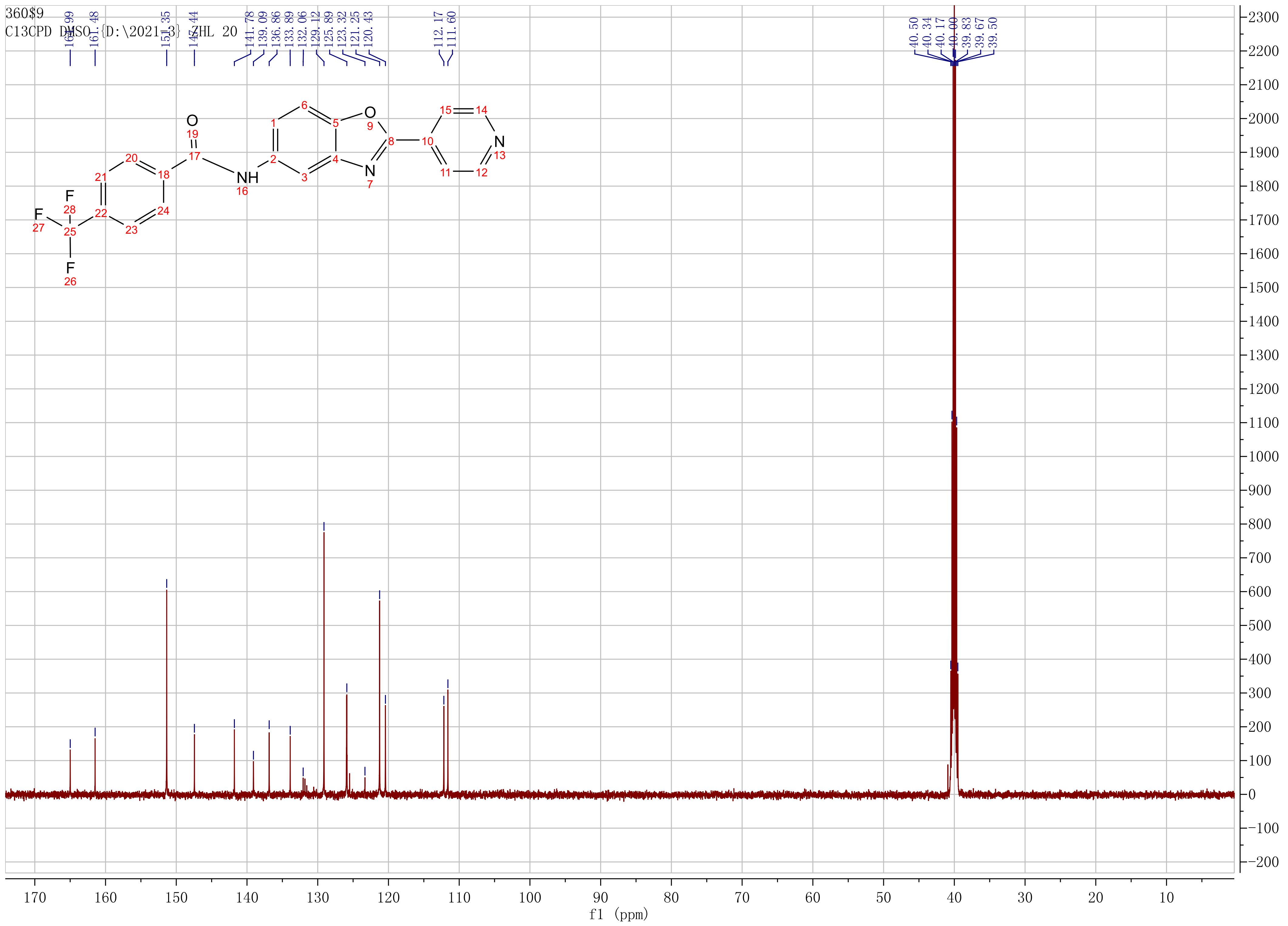

360$9
C13CPD DMSO {D:\2021-3} ZHL 20
164.99
161.48
151.35
147.44
141.78
139.09
136.86
133.89
132.06
129.12
125.89
123.32
121.25
120.43
112.17
111.60
40.50
40.34
40.17
40.00
39.83
39.67
39.50
O
19
17
NH
16
20
21
18
F
28
F
27
25
22
24
23
F
26
1
6
5
O
9
2
3
4
N
7
8
10
15
14
N
13
11
12
f1 (ppm)

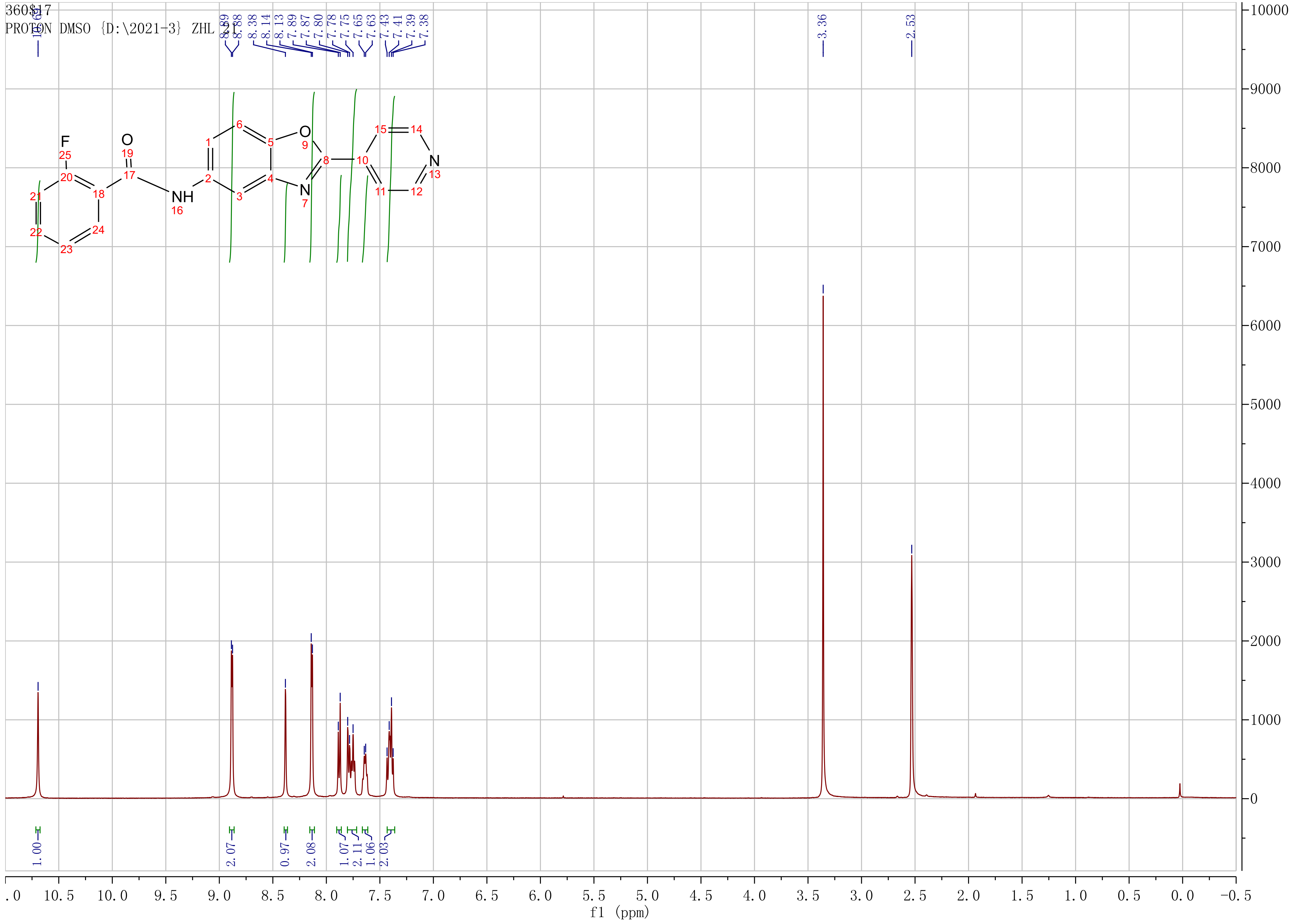
360$17
PROTON DMSO {D:\2021-3} ZHL 21
10.69
8.89
8.88
8.38
8.14
8.13
7.89
7.87
7.80
7.78
7.75
7.65
7.63
7.43
7.41
7.39
7.38
3.36
2.53
F
O
NH
O
N
N
1.00
2.07
0.97
2.08
1.07
2.11
1.06
2.03
f1 (ppm)

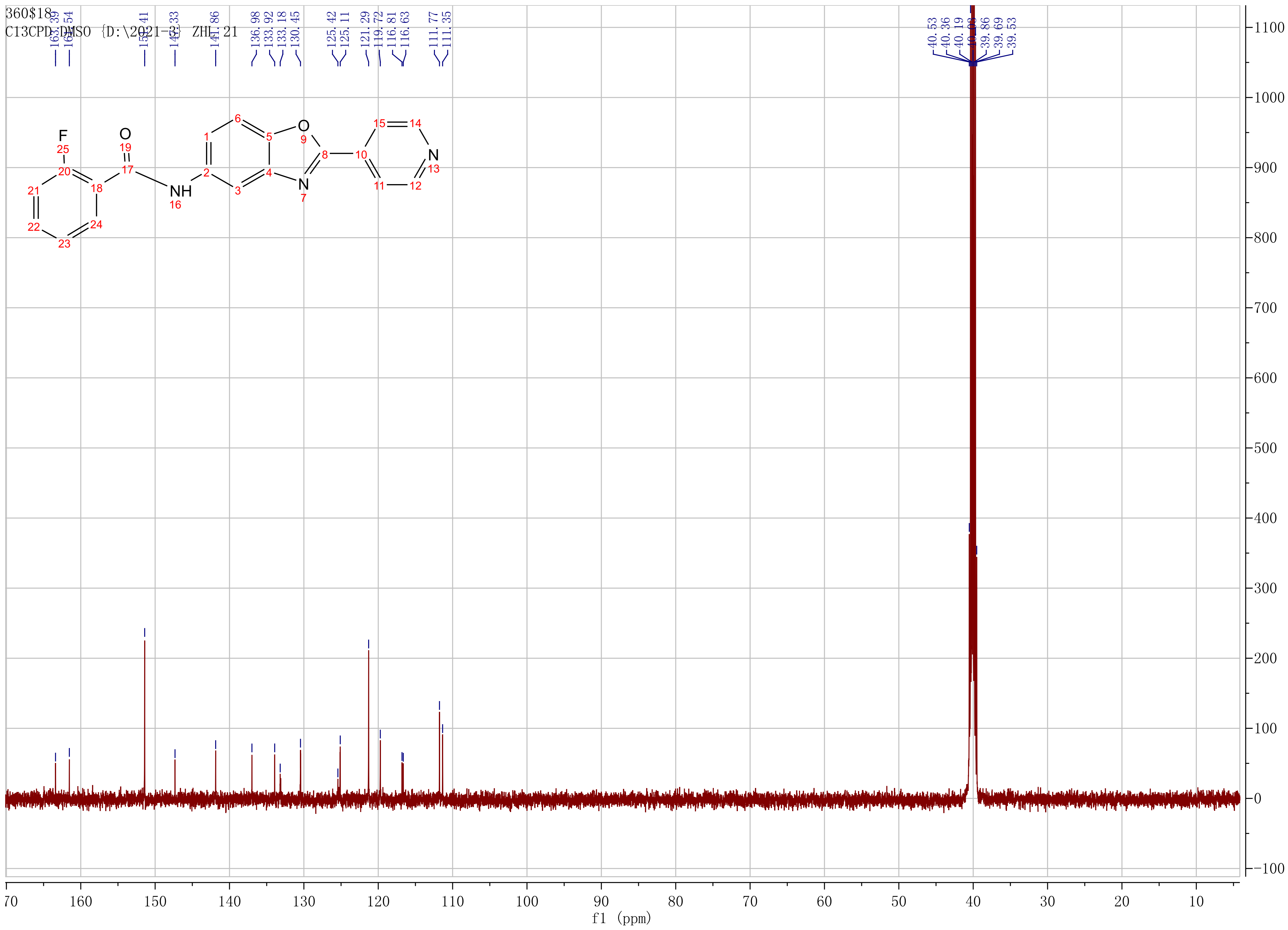

360$18
C13CPD-DMSO {D:\2021-3} ZHL 21
136.98
133.92
133.18
130.45
125.42
125.11
121.29
119.72
116.81
116.63
111.77
111.35
40.53
40.36
40.19
39.86
39.69
39.53
F
O
NH
O
N
N
f1 (ppm)

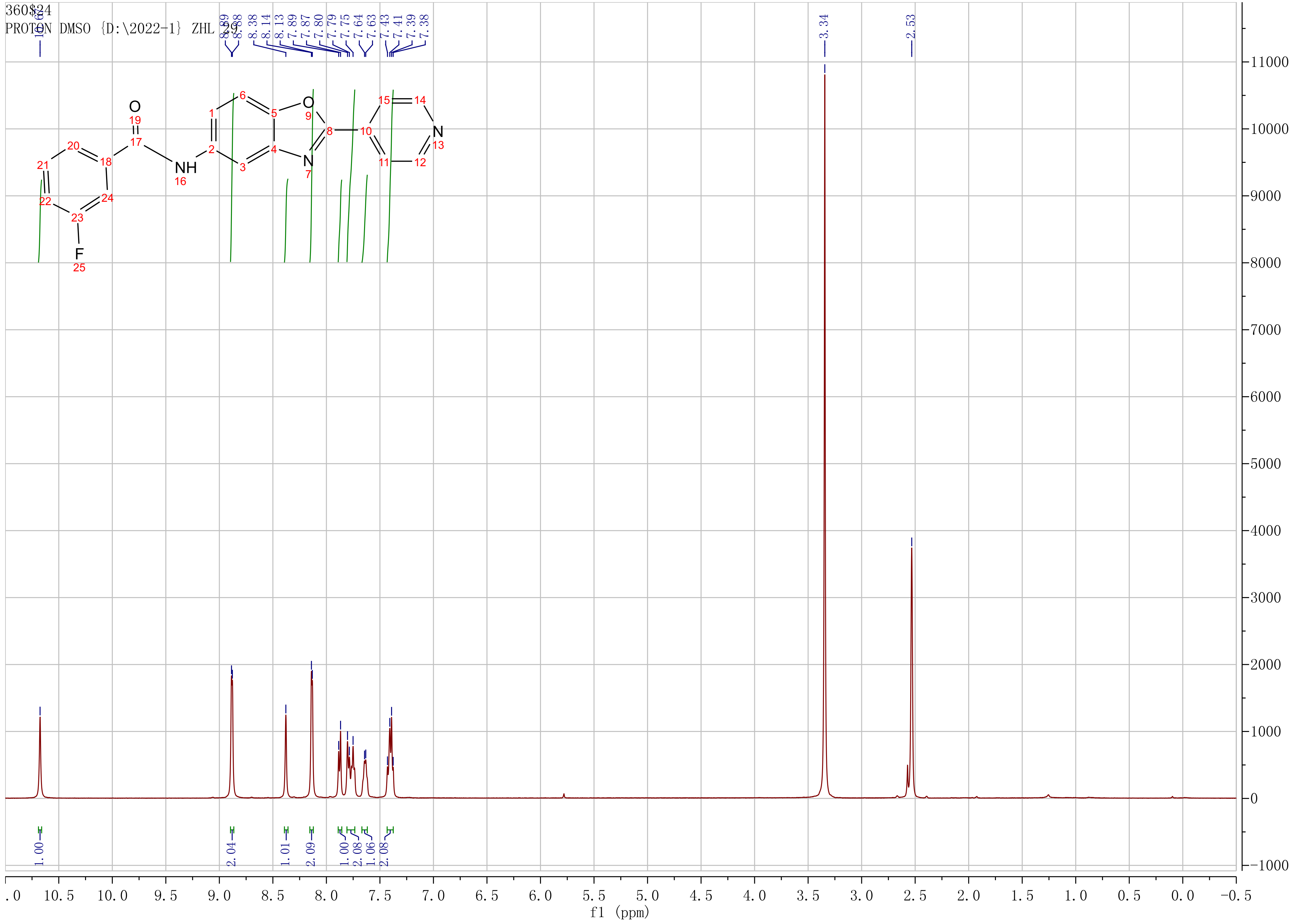

360$24
PROTON DMSO {D:\2022-1} ZHL 29
10.67
8.89
8.88
8.38
8.14
8.13
7.89
7.87
7.80
7.79
7.75
7.64
7.63
7.43
7.41
7.39
7.38
3.34
2.53
1.00
2.04
1.01
2.09
1.00
2.08
1.06
2.08
f1 (ppm)

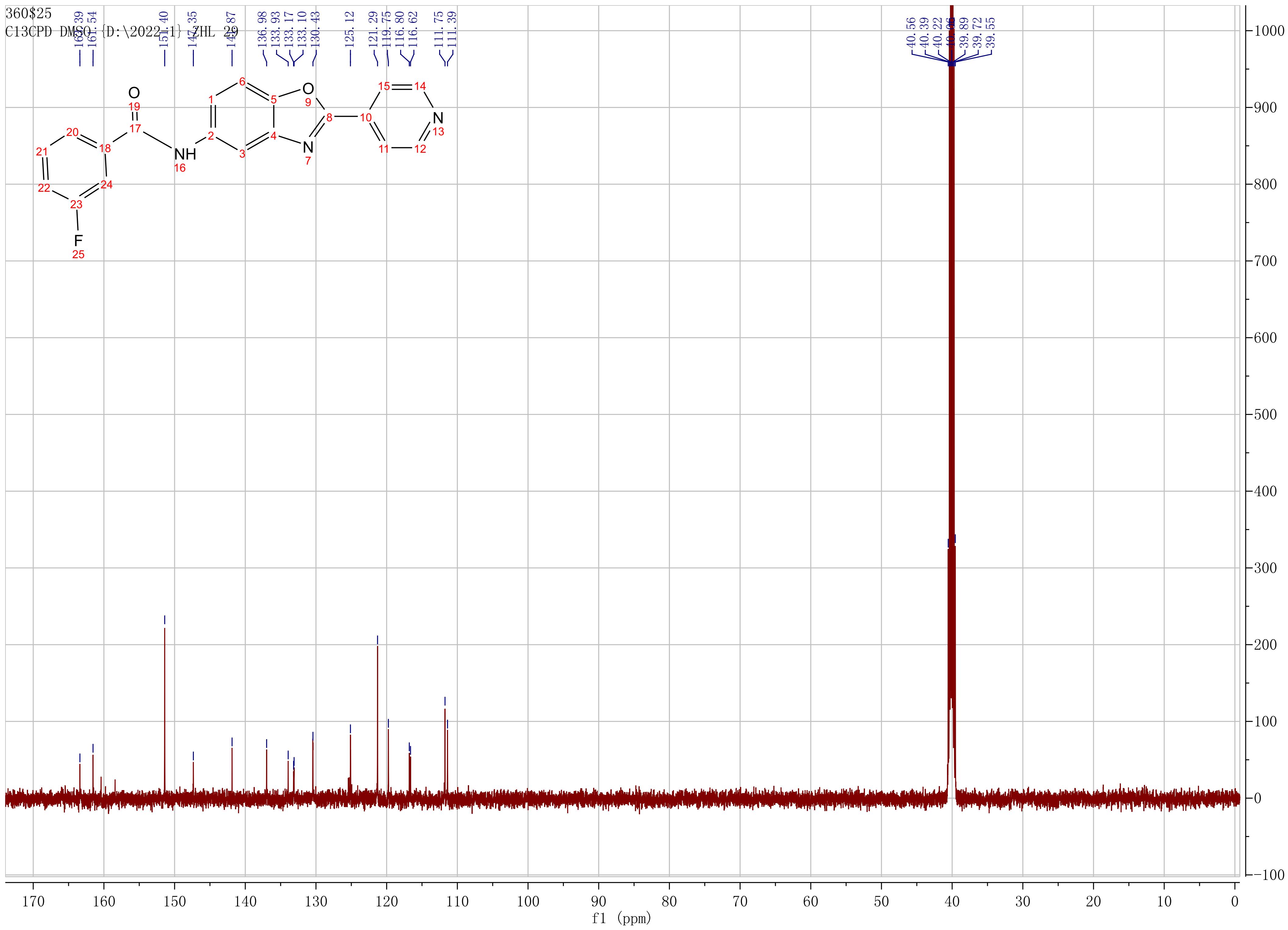

360$25
C13CPD DMSO {D:\2022-1} ZHL 29
161.54
151.40
147.35
136.98
133.93
133.17
133.10
130.43
125.12
121.29
119.75
116.80
116.62
111.75
111.39
40.56
40.39
40.22
39.89
39.72
39.55
f1 (ppm)

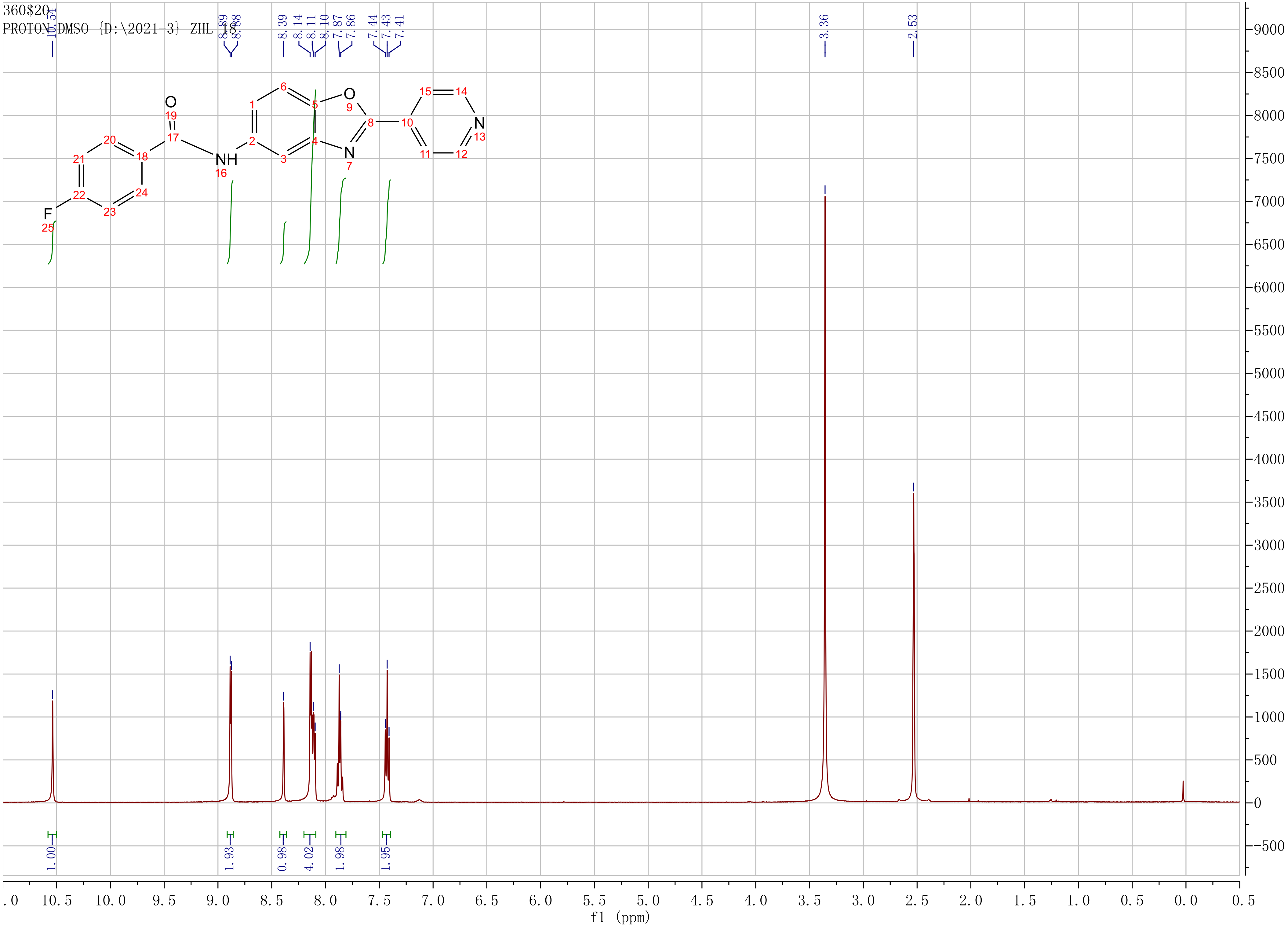

360$20
PROTON-DMSO {D:\2021-3} ZHL 18
10.54
8.89
8.88
8.39
8.14
8.11
8.10
7.87
7.86
7.44
7.43
7.41
3.36
2.53
1.00
1.93
0.98
4.02
1.98
1.95
f1 (ppm)

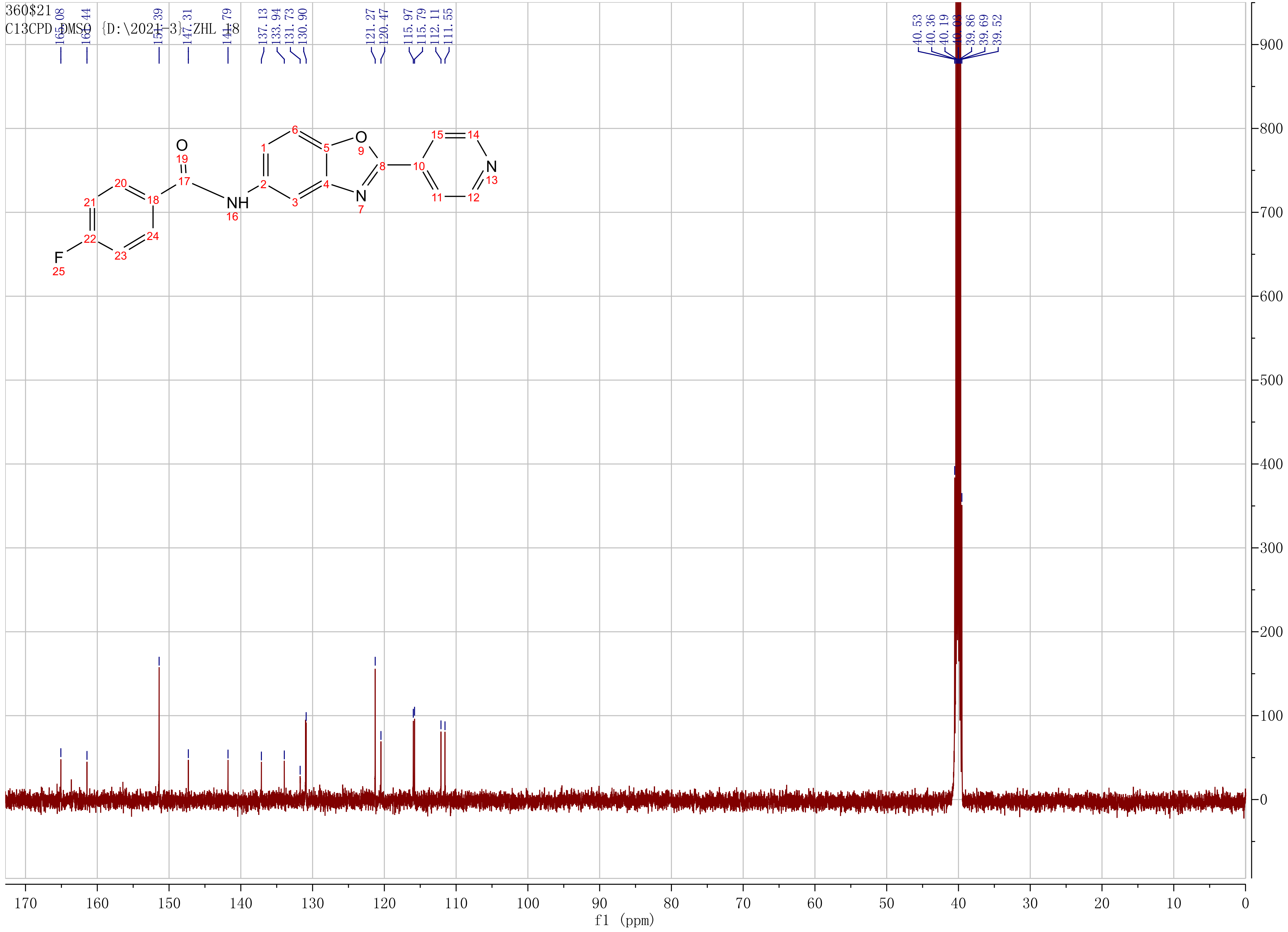
360$21
C13CPD DMSO {D:\2021-3} ZHL 18
165.08
161.44
151.39
147.31
141.79
137.13
133.94
131.73
130.90
121.27
120.47
115.97
115.79
112.11
111.55
40.53
40.36
40.19
40.03
39.86
39.69
39.52
f1 (ppm)

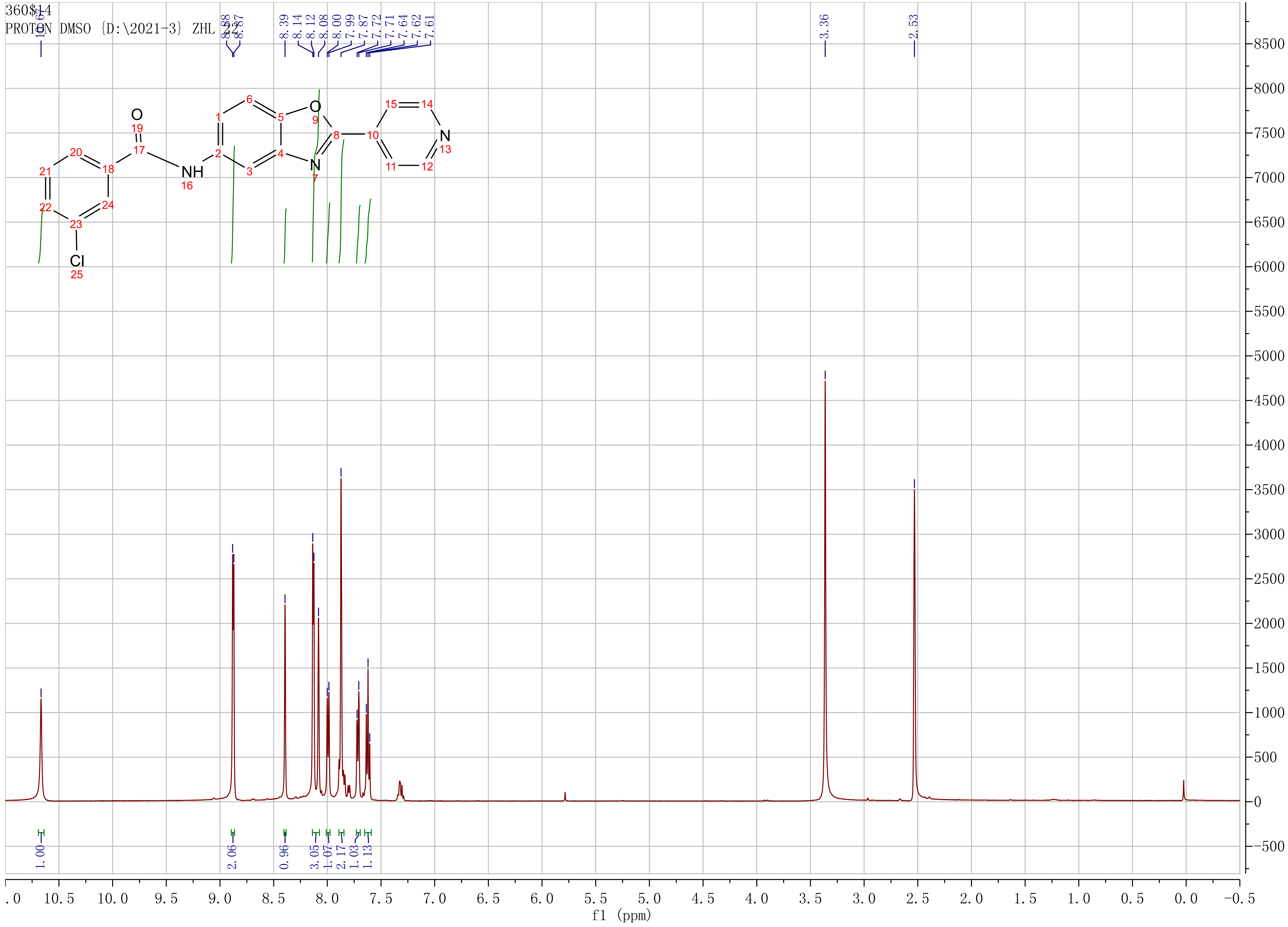

360$14
PROTON DMSO {D:\2021-3} ZHL 22
10.67
8.88
8.87
8.39
8.14
8.12
8.08
8.00
7.99
7.87
7.72
7.71
7.64
7.62
7.61
3.36
2.53
1.00
2.06
0.96
3.05
1.07
2.17
1.03
1.13
f1 (ppm)
Cl
NH
O
N

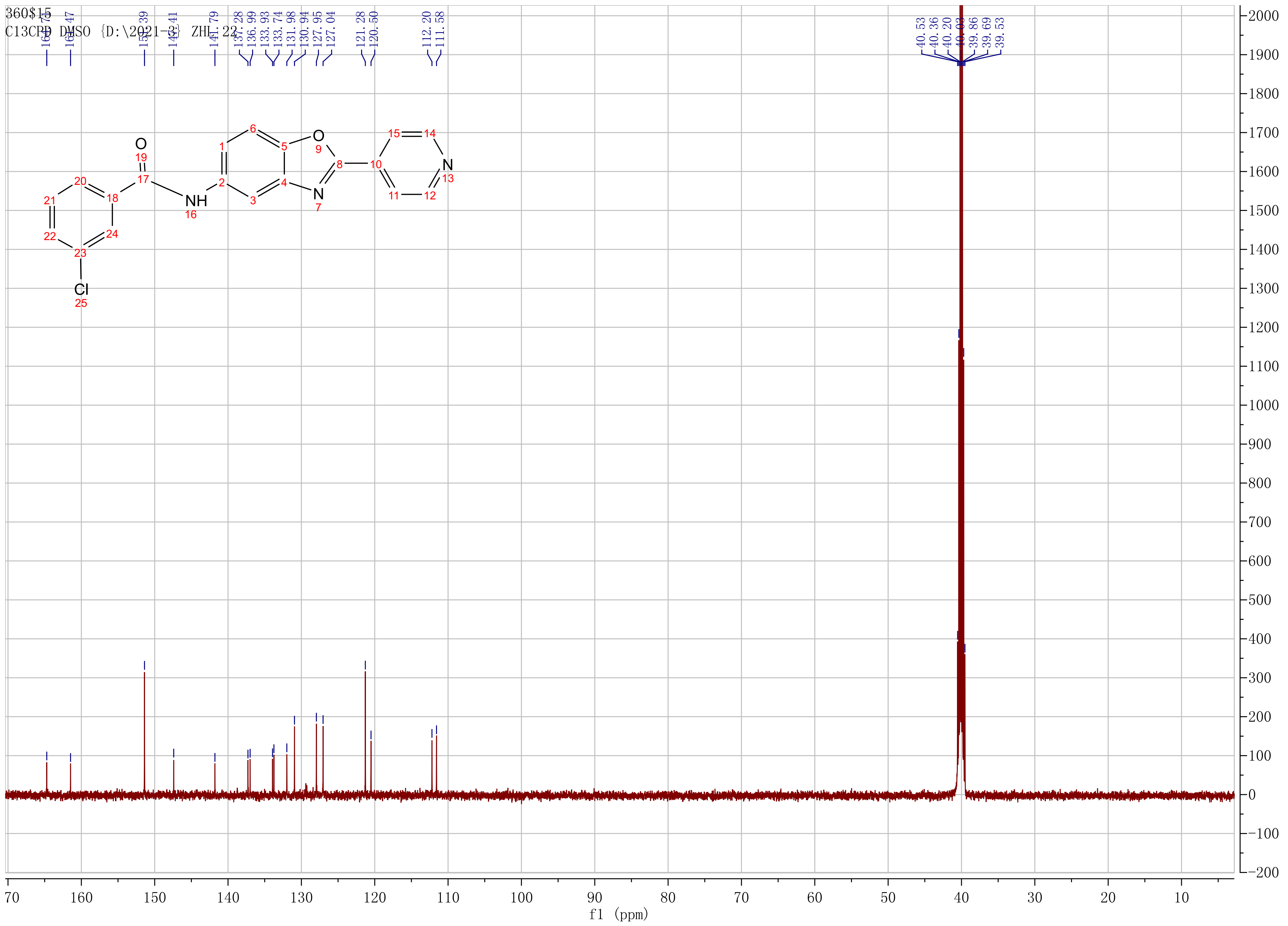

360$15
C13CPD DMSO {D:\2021-3} ZHL 22
151.39
137.28
136.99
133.93
133.74
131.98
130.94
127.95
127.04
121.28
120.50
112.20
111.58
40.53
40.36
40.20
40.03
39.86
39.69
39.53
f1 (ppm)

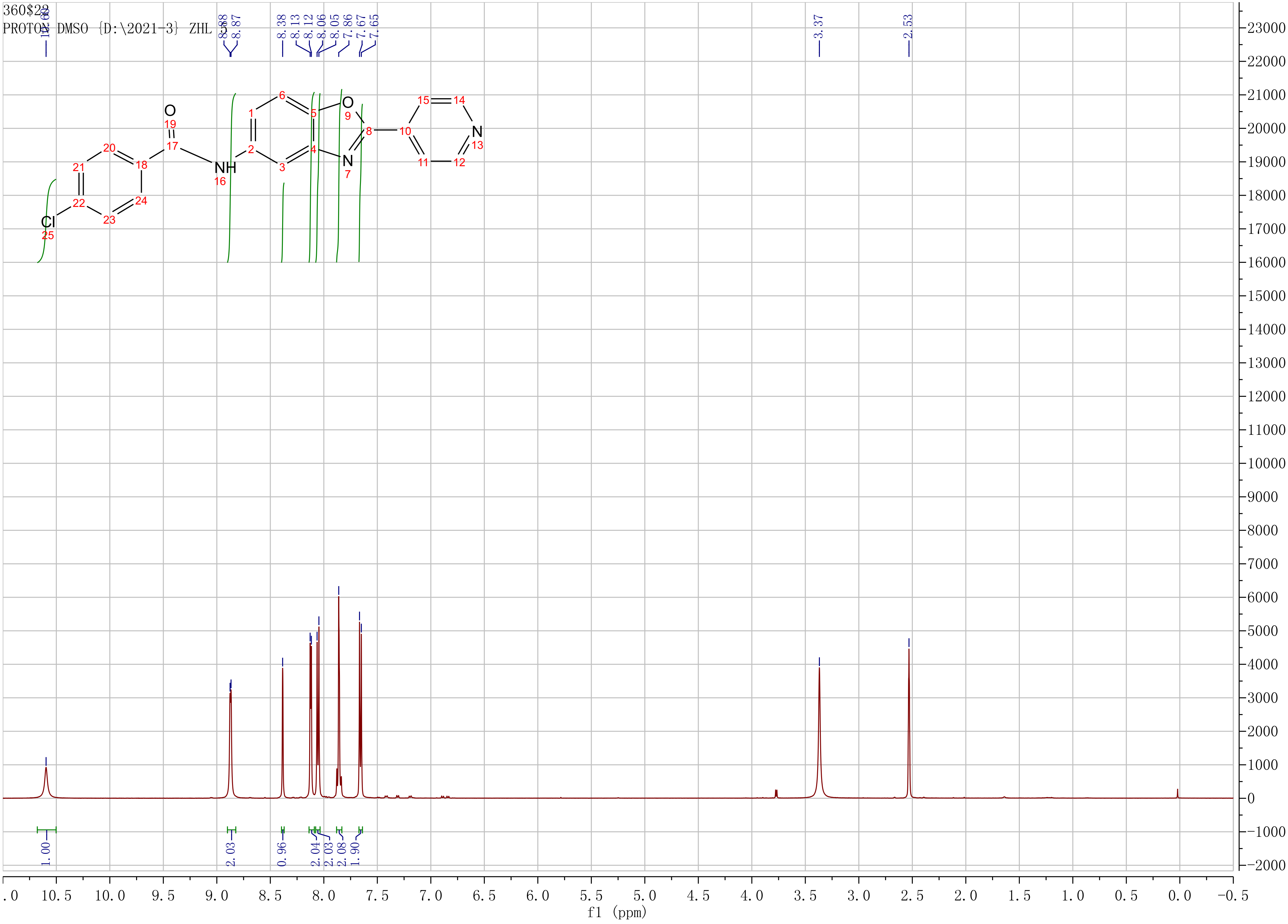

360$22
PROTON DMSO {D:\2021-3} ZHL 5
10.60
8.88
8.87
8.38
8.13
8.12
8.06
8.05
7.86
7.67
7.65
3.37
2.53
1.00
2.03
0.96
2.04
2.03
2.08
1.90
O
19
17
NH
16
Cl
25
N
13
N
7
O
9
f1 (ppm)

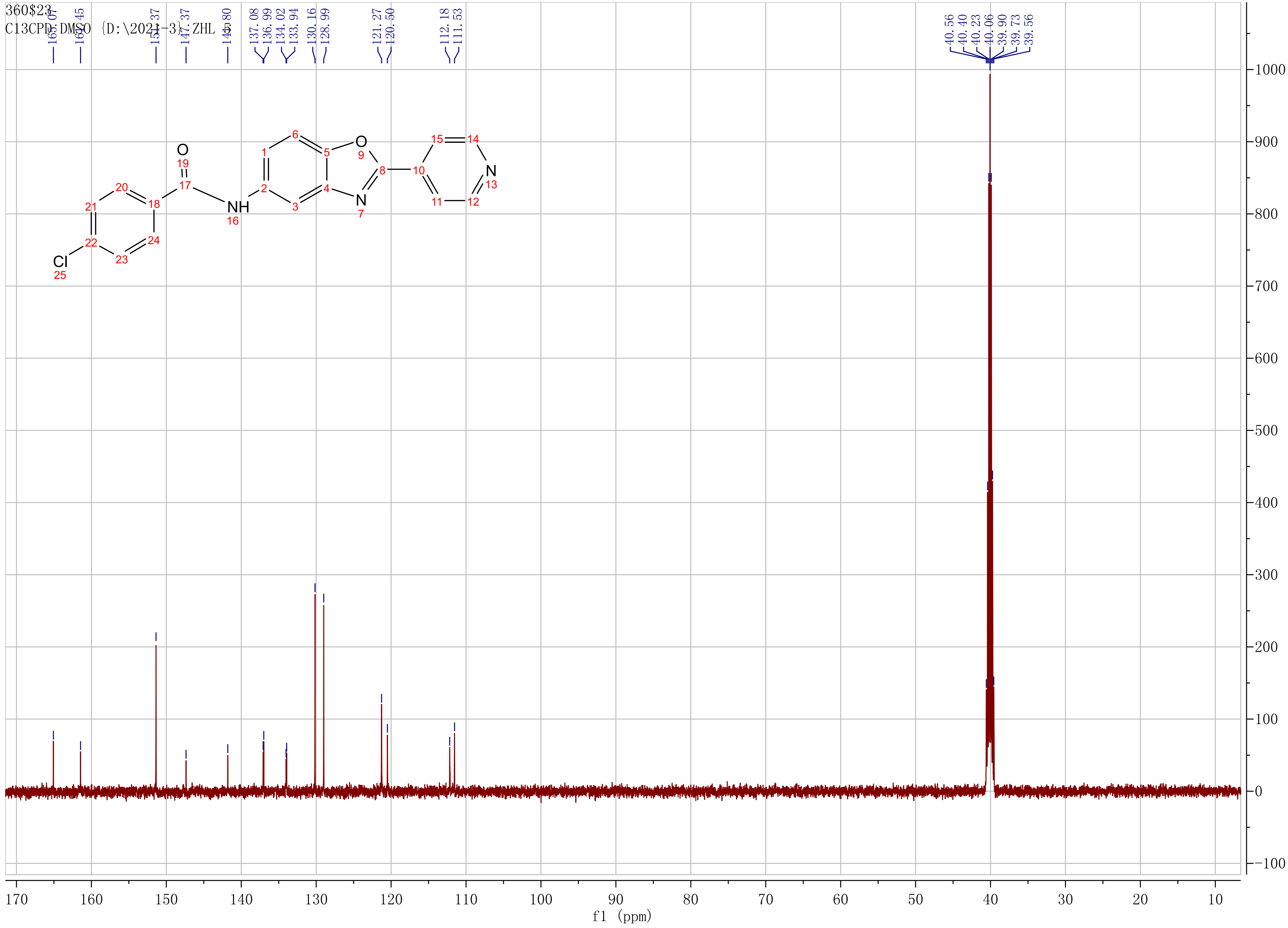

360$23
C13CPD DMSO {D:\2021-3} ZHL
165.07
161.45
151.37
147.37
141.80
137.08
136.99
134.02
133.94
130.16
128.99
121.27
120.50
112.18
111.53
40.56
40.40
40.23
40.06
39.90
39.73
39.56
O
NH
N
Cl
f1 (ppm)

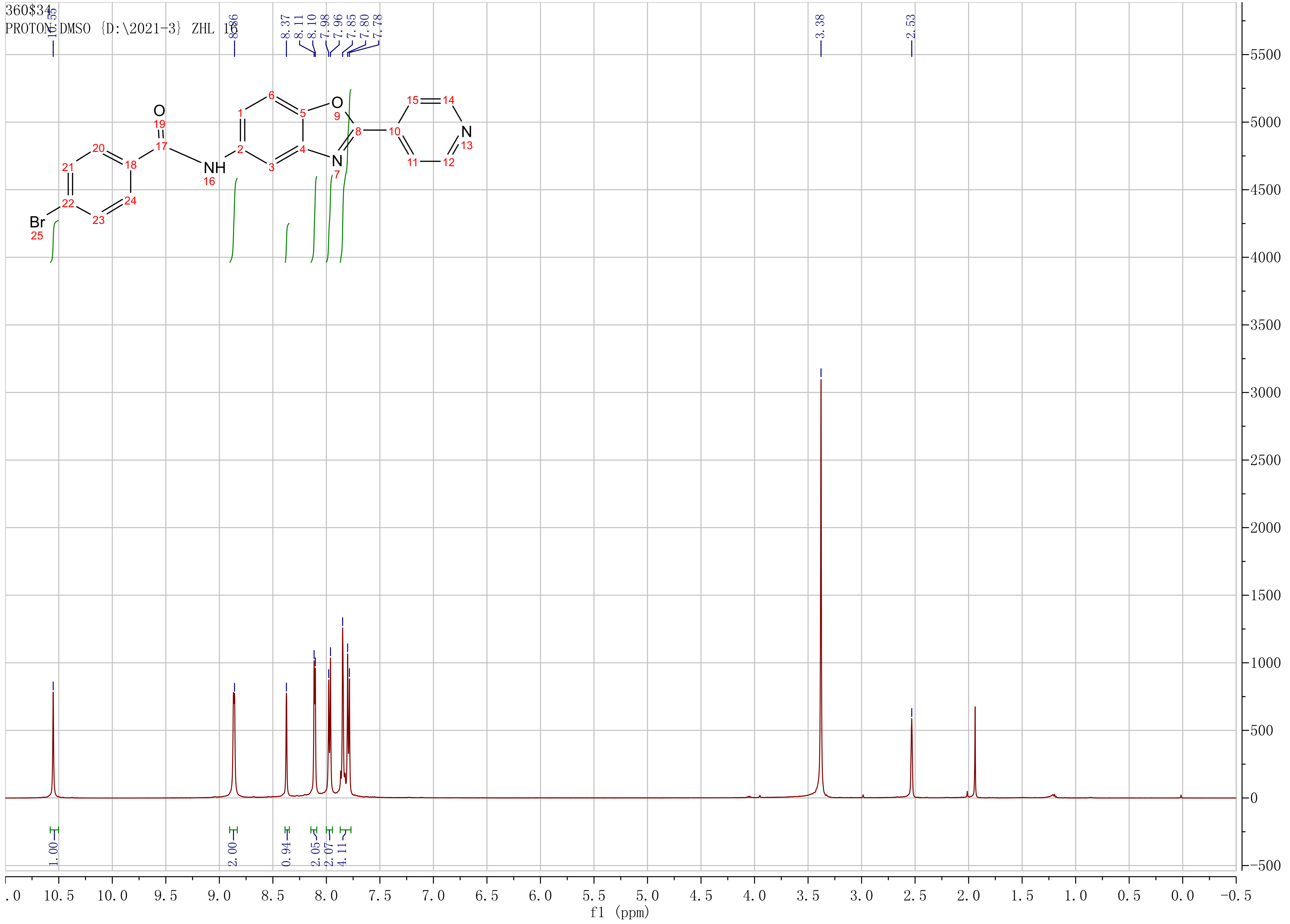

360$34
PROTON DMSO {D:\2021-3} ZHL 16
10.55
8.86
8.37
8.11
8.10
7.98
7.96
7.85
7.80
7.78
3.38
2.53
1.00
2.00
0.94
2.05
2.07
4.11
f1 (ppm)

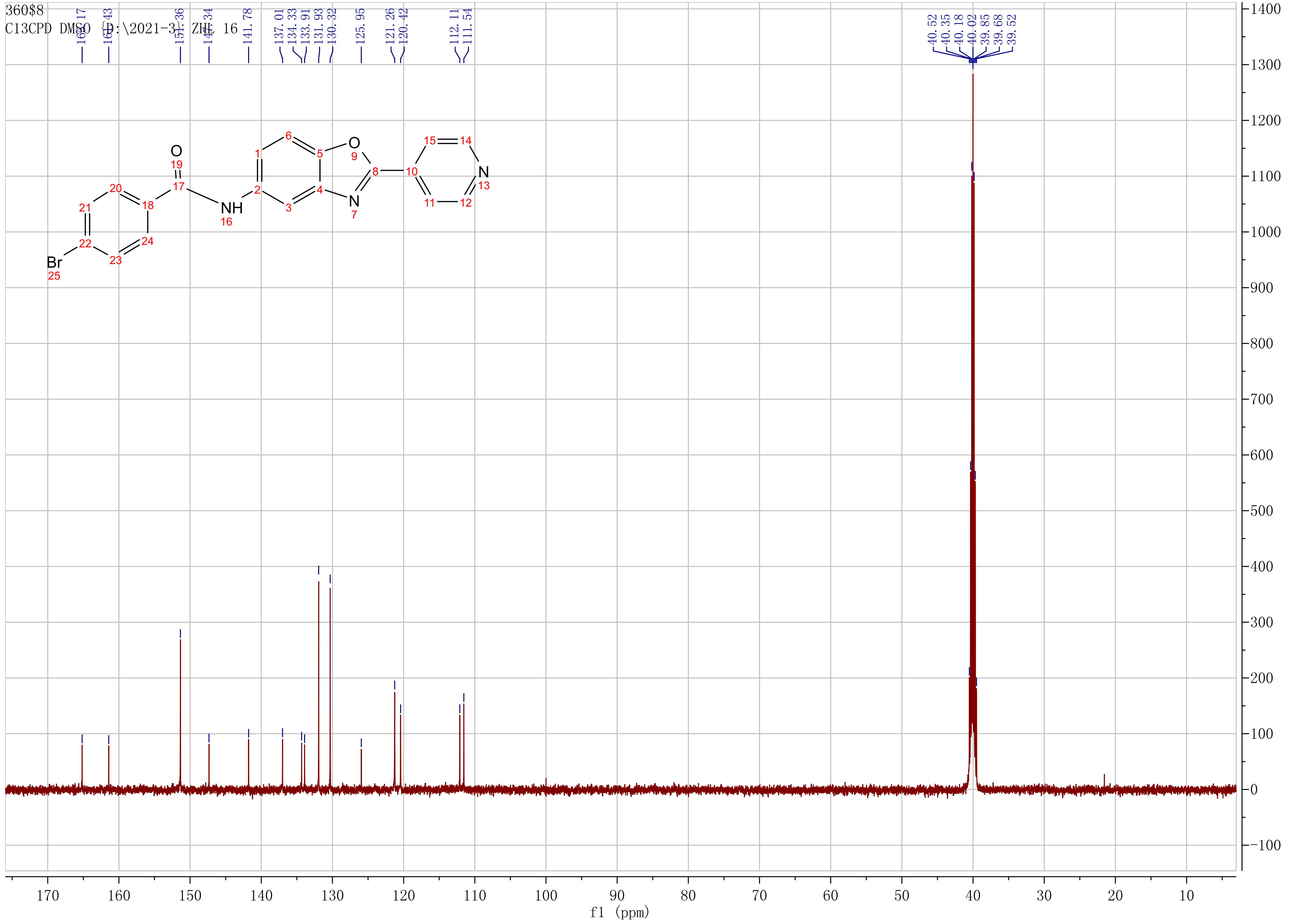

360$8
C13CPD DMSO {D:\2021-3} ZHL 16
165.17
161.43
151.36
147.34
141.78
137.01
134.33
133.91
131.93
130.32
125.95
121.26
120.42
112.11
111.54
40.52
40.35
40.18
40.02
39.85
39.68
39.52
f1 (ppm)

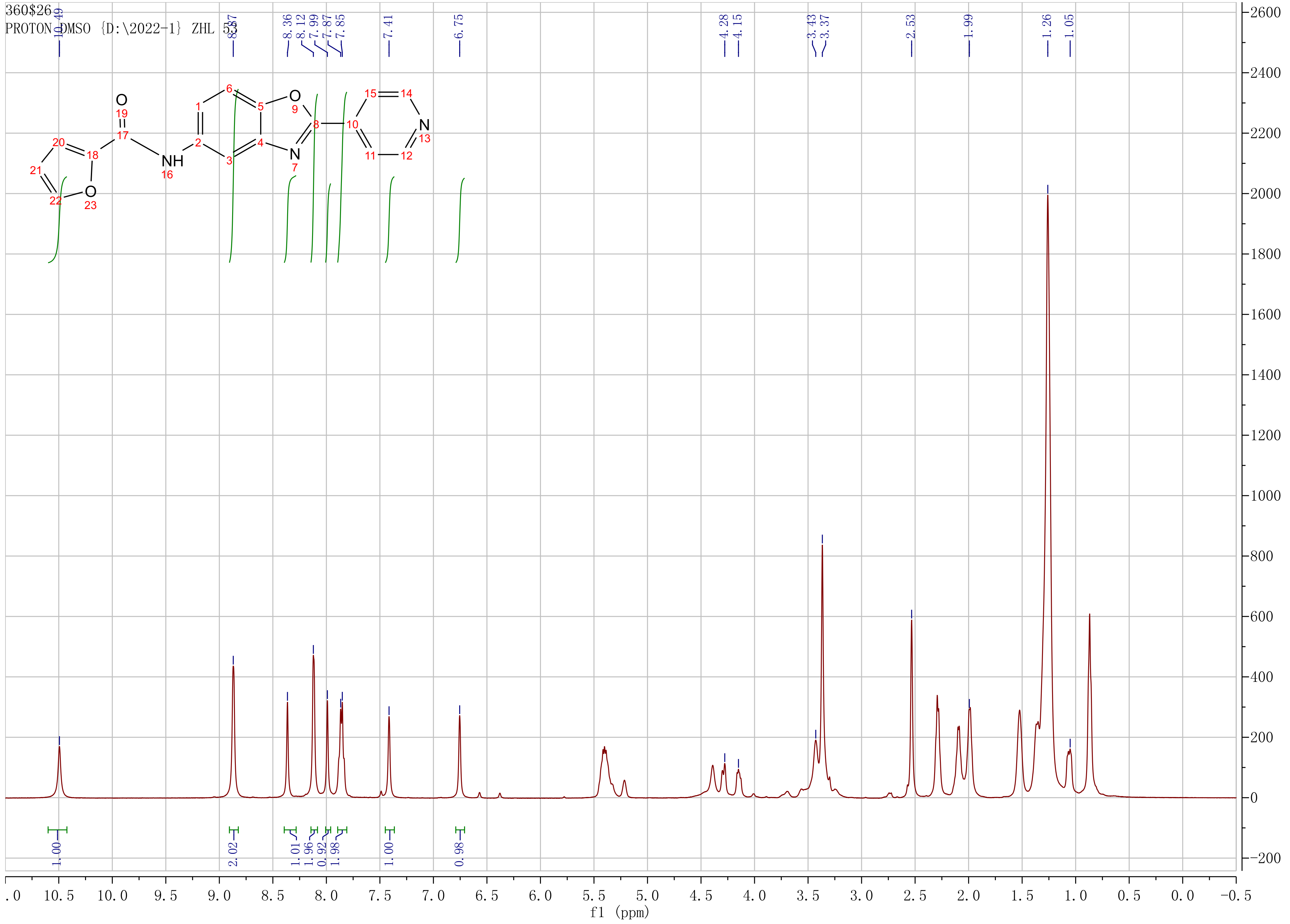

360$26
PROTON_DMSO {D:\2022-1} ZHL 53
10.49
8.87
8.36
8.12
7.99
7.87
7.85
7.41
6.75
4.28
4.15
3.43
3.37
2.53
1.99
1.26
1.05
1.00
2.02
1.01
1.96
0.92
1.98
1.00
0.98
f1 (ppm)

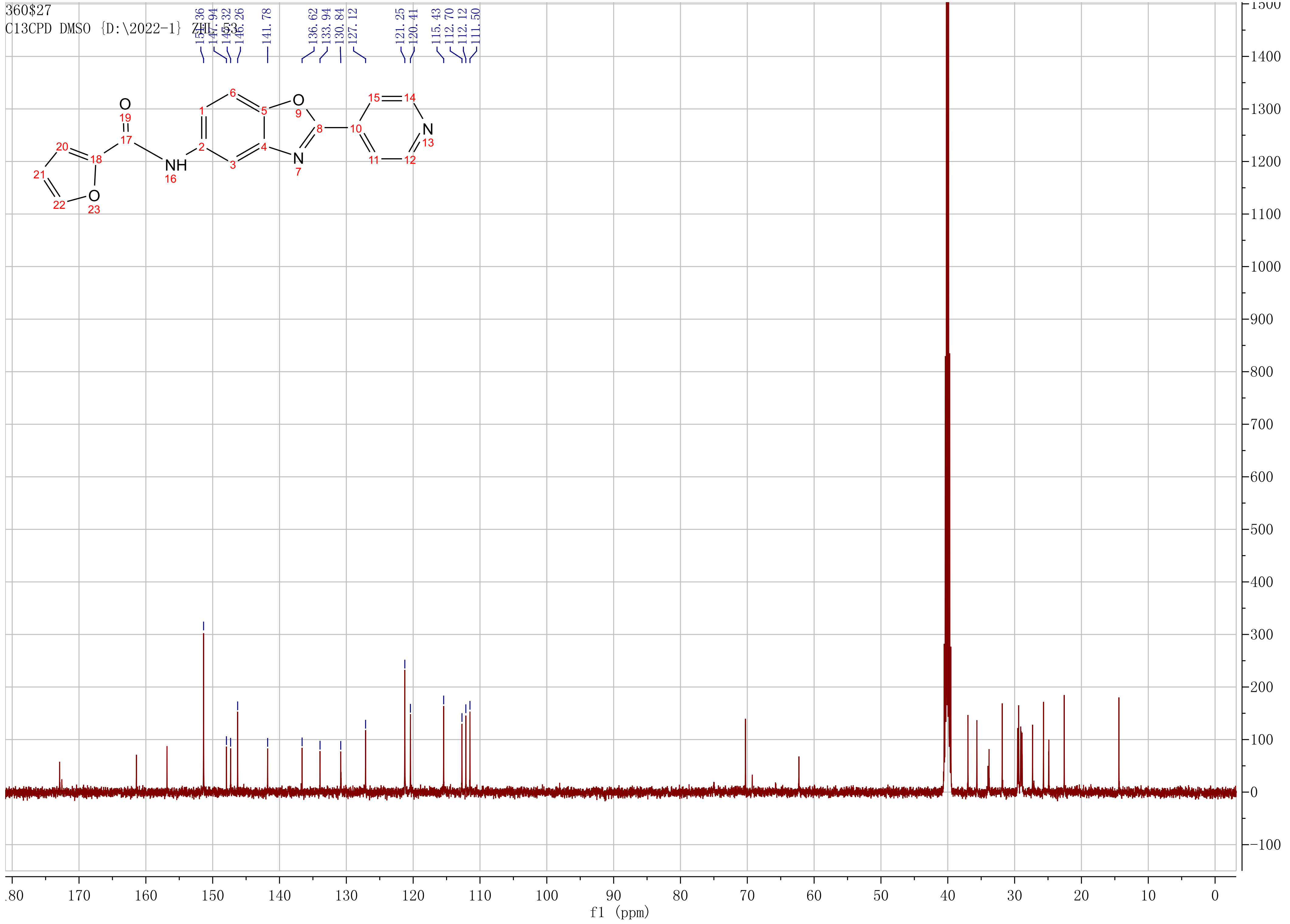
360$27
C13CPD DMSO {D:\2022-1} ZHL-53
146.26
141.78
136.62
133.94
130.84
127.12
121.25
120.41
115.43
112.70
112.12
111.50
f1 (ppm)

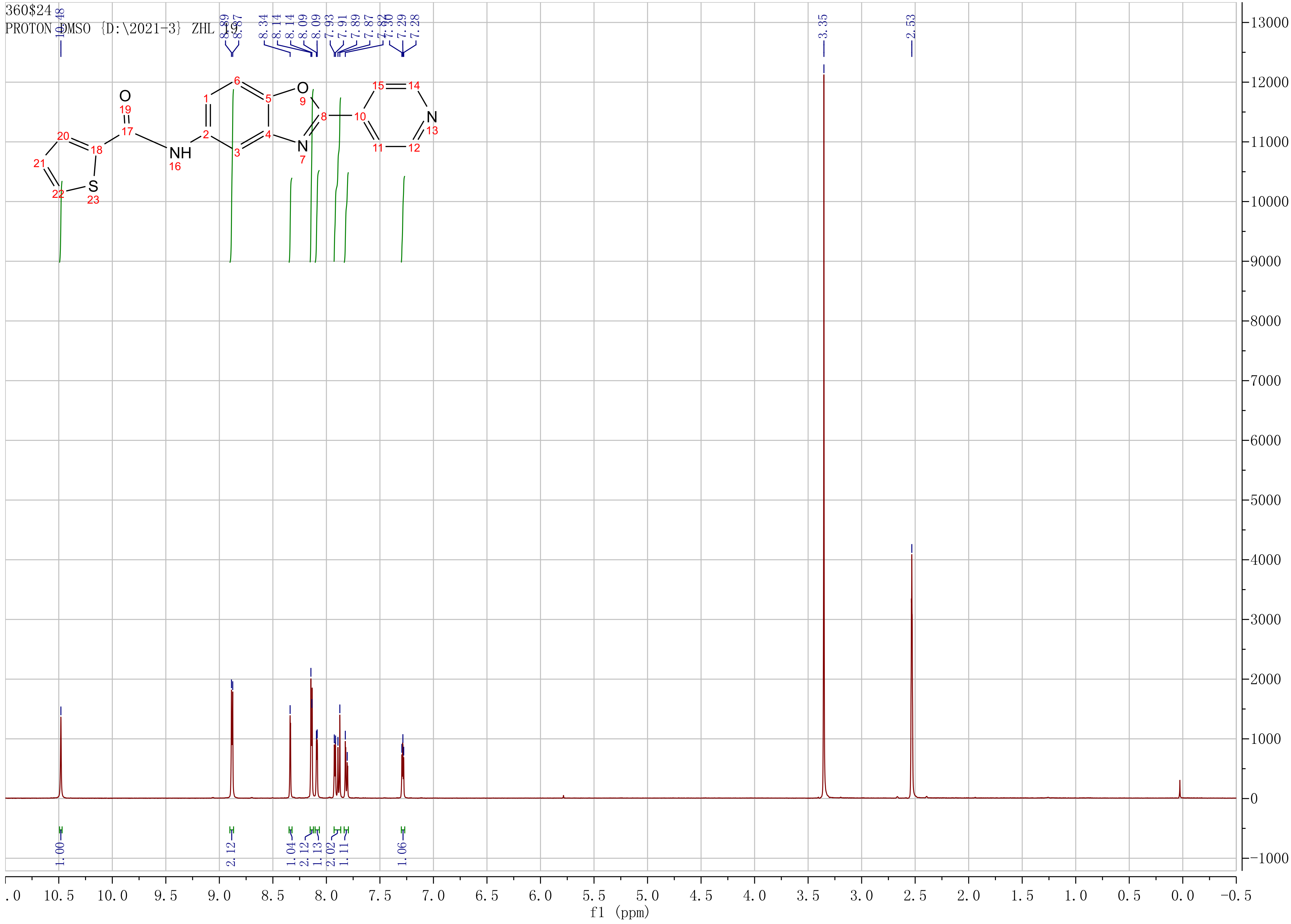

360$24
PROTON DMSO {D:\2021-3} ZHL 19
10.48
8.89
8.87
8.34
8.14
8.14
8.09
8.09
7.93
7.91
7.89
7.87
7.82
7.30
7.29
7.28
3.35
2.53
1.00
2.12
1.04
2.12
1.13
2.02
1.11
1.06
f1 (ppm)

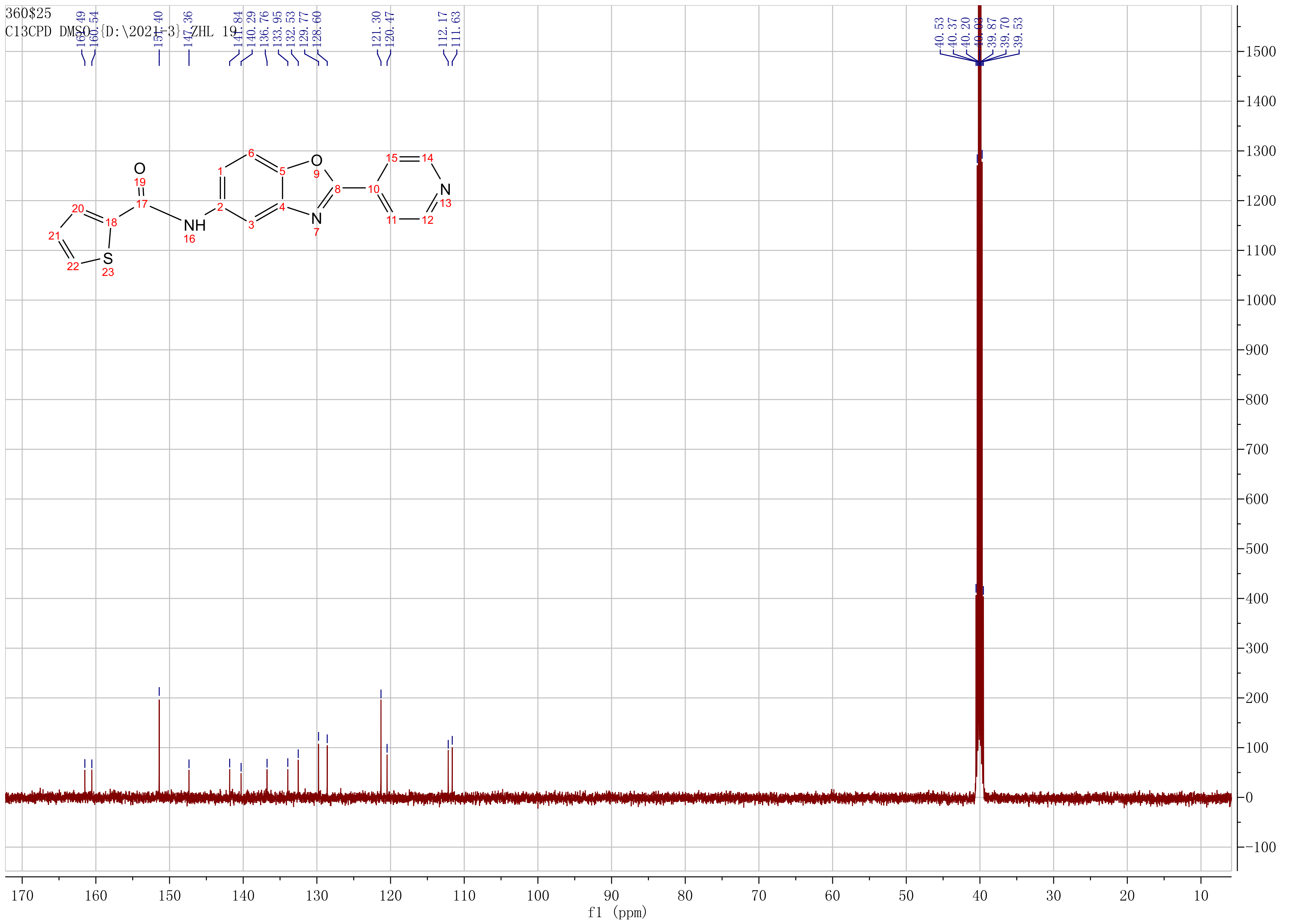

360$25
C13CPD DMSO {D:\2021-3} ZHL 19
160.54
151.40
147.36
141.84
140.29
136.76
133.95
132.53
129.77
128.60
121.30
120.47
112.17
111.63
40.53
40.37
40.20
39.87
39.70
39.53
O
NH
N
S
f1 (ppm)

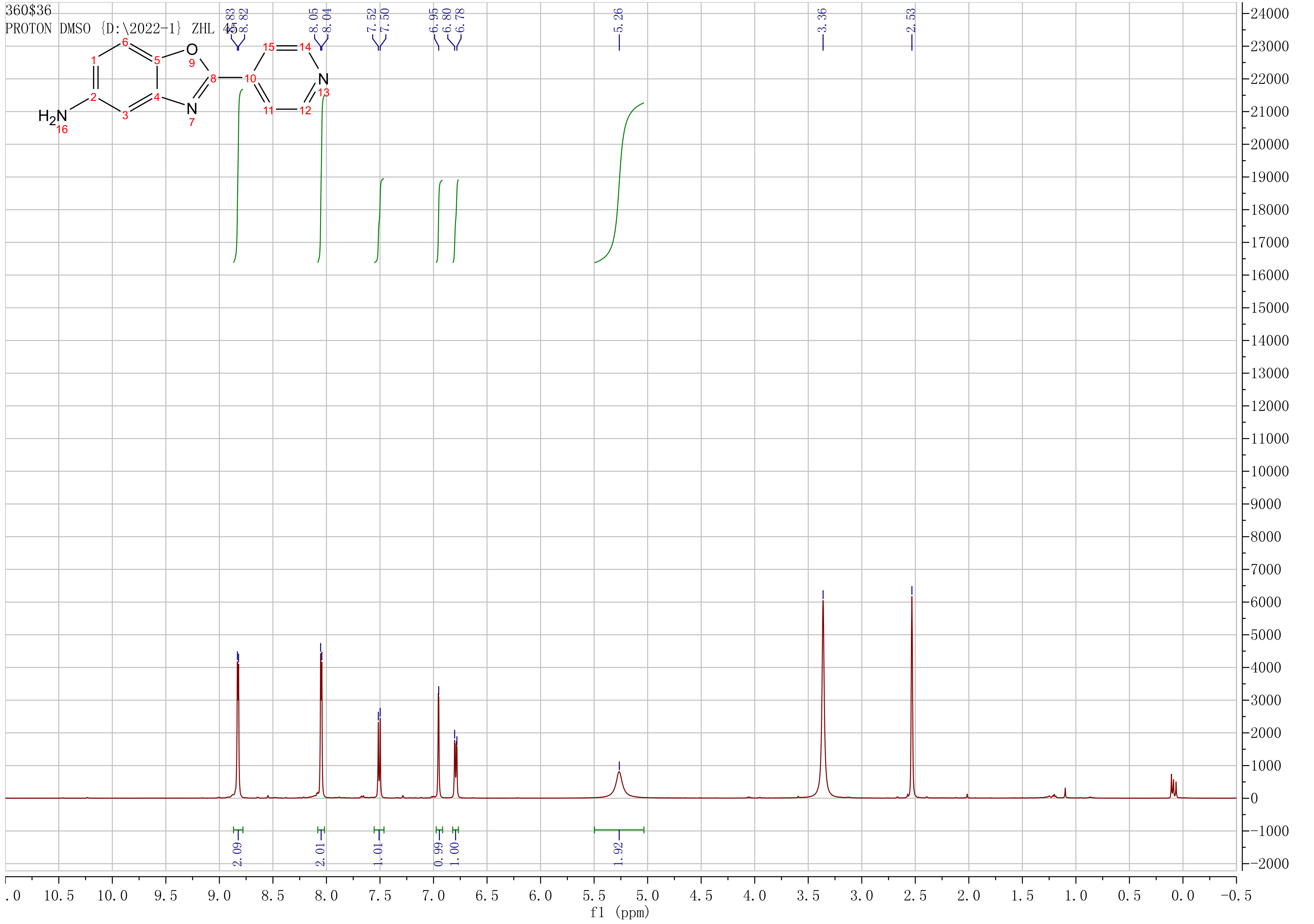

360$36
PROTON DMSO {D:\2022-1} ZHL 45
8.83
8.82
8.05
8.04
7.52
7.50
6.95
6.80
6.78
5.26
3.36
2.53
H2N
O
N
N
2.09
2.01
1.01
0.99
1.00
1.92
f1 (ppm)

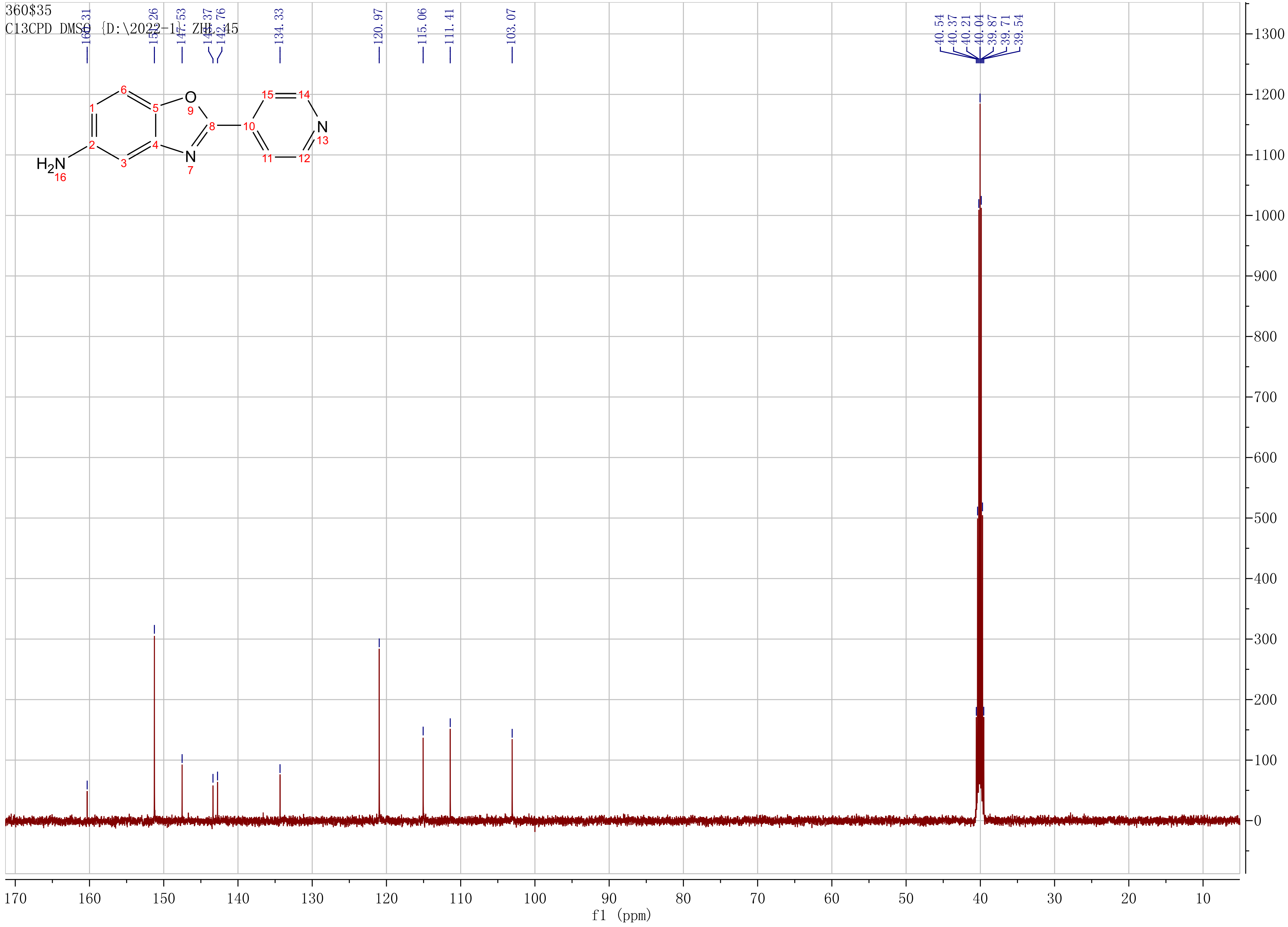
360$35
C13CPD DMSO {D:\2022-1 ZHL 45
160.31
151.26
147.53
143.37
142.76
134.33
120.97
115.06
111.41
103.07
40.54
40.37
40.21
40.04
39.87
39.71
39.54
f1 (ppm)
